# Silylium‐Ion‐Initiated Disulfidation of Internal Alkynes With Disulfides

**DOI:** 10.1002/chem.71187

**Published:** 2026-05-25

**Authors:** Dáiríne M. Morgan, Hendrik F. T. Klare, Martin Oestreich

**Affiliations:** ^1^ Institut für Chemie Technische Universität Berlin Berlin Germany

**Keywords:** alkynes, difunctionalization, silicon, silylium ions, sulfonium ions, sulfur

## Abstract

A two‐component protocol for the *trans*‐selective addition of diaryl disulfides across internal alkynes is reported. The silylium‐ion initiator converts the disulfide reagent into a silylated sulfonium ion and, as such, into a sulfenium‐ion source. A subsequent transfer of that sulfur electrophile to the C≡C triple bond leads to the formation of a thiirenium ion. The ring opening of this intermediate by the disulfide as a sulfur nucleophile yields the disulfidation (bisthiolation) product with release of another sulfenium ion. The resulting 1,2‐bis(arylthio)‐substituted alkene derivatives can be oxidized to the corresponding sulfoxides and sulfones or further engaged in regioselective nickel‐catalyzed cross‐coupling reactions.

## Graphical abstract

Silylium‐ion initiation enables the disulfidation of internal alkynes with diaryl disulfides. The catalytic turnover is maintained by the sulfonium ion ArS(ArSSAr)^+^ as a sulfenium‐ion carrier that reacts with an alkyne to form a thiirenium ion. Its ring opening by disulfide addition yields the 1,2‐bis(arylthio)‐substituted alkene product with *E:Z* > 95:5 in all cases.

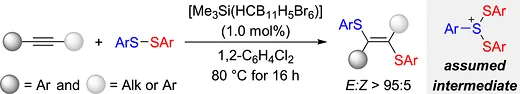

## 1 Introduction

Catalysis with silylium ions is an emerging research field at the intersection of conventional Lewis acid and superelectrophile chemistry [1, 2]. More precisely, silicon cations with silylium‐ion‐like character can be defined catalysts, reagents that are regenerated by proton‐into‐silylium‐ion interconversion [3, 4] as part of a catalytic cycle, or even just initiators [1, 2].

**SCHEME 1:**
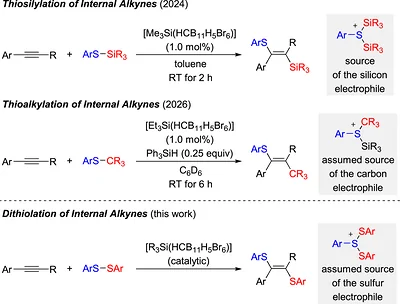
Silylium‐ion‐promoted difunctionalizations of internal alkynes. Blue denotes sulfur nucleophile and red denotes an element electrophile in the sulfide reactant.

Our laboratory recently showed that adduct formation between silylium ions and sulfides can be exploited to promote alkyne difunctionalizations with silylated sulfonium ions as key intermediates (Scheme 1, top). Thiosilylation was achieved with thiosilanes [5], and thioalkylation (mainly *tert*‐butylthiolation) became possible with thioethers [6]. Both were found to proceed in a regio‐ and stereoselective manner. The silylium ion, however, assumes different roles in these similar transformations: reagent in the former but either initiator or catalyst in the latter. We asked ourselves whether this strategy could also be applied to the related disulfidation (bisthiolation) using disulfides as both a sulfur electrophile and a sulfur nucleophile (Scheme 1, bottom). In fact, Oshima and co‐workers had realized this reaction with GaCl_3_ as an initiator or catalyst, also formulating a cationic mechanism [7]. Matsumoto, Yoshida, and co‐workers later used electrochemical oxidation as an entry point where the sulfonium ion ArS(ArSSAr)^+^ is actually generated prior to the addition of the alkyne [8, 9]. This preformation clearly supports a cationic chain mechanism [9]. Starting from this insight, we report here a silylium‐ion‐initiated disulfidation of alkynes [10, 11, 12, 13, 14, 15, 16, 17, 18, 19, 20, 21, 22]. A number of transition‐metal‐free methods for the disulfidation of terminal alkynes have previously been developed [23, 24, 25, 26, 27, 28], but applications involving internal alkynes and those leading to the *trans* product remain limited.

## 2 Results and Discussion

We began our optimization using but‐1‐yn‐1‐ylbenzene (**1a**) and diphenyl disulfide (**2a**) as model compounds (Tables 1 and S1–S4). Using the optimized conditions from the aforementioned thiosilylation reaction [5], i.e., 1.0 mol% of [Me_3_Si(HCB_11_H_5_Br_6_)] at room temperature in toluene, no conversion of the starting material was observed (Table 1, entry 1). However, when the reaction temperature was increased to 80°C the desired product **3aa** was obtained as a single stereoisomer in a 37% yield (entry 2). A survey of solvents revealed that *ortho*‐dichlorobenzene afforded the best yield of 82% (entry 3). Variation of the reaction equivalents led to no further improvement in the reaction yield, with increased equivalents of the alkyne leading to a sharp decline in the product yield (entry 4). Other initiators such as [Et_3_Si(HCB_11_H_5_Br_6_)] and [Ph_3_C][HCB_11_H_5_Br_6_] were also capable of initiating the reaction but slightly reduced yields of 68% and 77% respectively were obtained (entries 5 and 6). The smaller steric demand of the Me_3_Si^+^ ion likely leads to it being a better initiator; the trialkylsilylium ions are also more electrophilic than the trityl cation which may further contribute to the differences observed. Under optimized conditions, product formation could still be detected at reduced temperatures, however, the yield was much lower at 39% (entry 7).

**TABLE 1:** Optimization of the *trans*‐selective alkyne disulfidation.^a^, ^b^

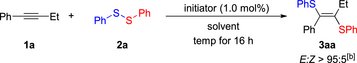						

Entry	Initiator	Solvent	Temp. (°C)	Alkyne 1a (equiv)	Disulfide 2a (equiv)	Yield of 3aa (%)^c^
1	[Me_3_Si(HCB_11_H_5_Br_6_)]	PhMe	RT	1.0	1.2	n.r.
2	[Me_3_Si(HCB_11_H_5_Br_6_)]	PhMe	80	1.0	1.2	37
3	[Me_3_Si(HCB_11_H_5_Br_6_)]	1,2‐C_6_H_4_Cl_2_	80	1.0	1.2	82
4	[Me_3_Si(HCB_11_H_5_Br_6_)]	1,2‐C_6_H_4_Cl_2_	80	1.5	1.0	33
5	[Et_3_Si(HCB_11_H_5_Br_6_)]	1,2‐C_6_H_4_Cl_2_	80	1.0	1.2	68
6	[Ph_3_C][HCB_11_H_5_Br_6_]	1,2‐C_6_H_4_Cl_2_	80	1.0	1.2	77
7	[Me_3_Si(HCB_11_H_5_Br_6_)]	1,2‐C_6_H_4_Cl_2_	RT	1.0	1.2	39

^a^ All reactions were performed on a 0.20 mmol scale in a glovebox under an argon atmosphere in 0.5 mL of the indicated solvent.

^b^ *E:Z* > 95:5 of **3aa** in all cases as verified by ^1^H NMR spectroscopy of the crude reaction mixture.

^c^ Yield determined by ^1^H NMR spectroscopy of the crude reaction mixture using CH_2_Br_2_ as an internal standard. n.r. = no reaction.

**SCHEME 2:**
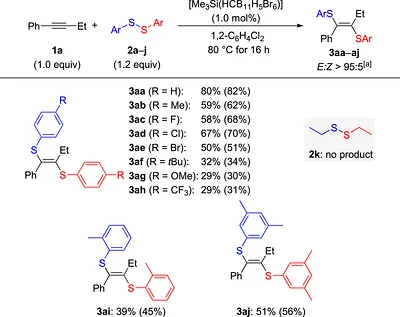
Substrate Scope I: Variation of the diaryl disulfide. All reactions were performed using the alkyne **1a** (0.20 mmol), the indicated disulfide **2** (0.24 mmol, 1.2 equiv), and the initiator [Me_3_Si(HCB_11_H_5_Br_6_)] (2.0 µmol, 1.0 mol%) in a glovebox under an argon atmosphere in 1,2‐C_6_H_4_Cl_2_ (0.5 mL) at 80°C. Isolated yields refer to analytically pure material after flash chromatography on silica gel; yields in parentheses determined by ^1^H NMR spectroscopy of the crude reaction mixture using CH_2_Br_2_ as an internal standard. (a) *E:Z* > 95:5 of **3** in all cases as verified by ^1^H NMR spectroscopy of the crude reaction mixture.

With optimized conditions in hand, we next turned to the substrate scope of the reaction (Scheme 2). We began with the diaryl disulfides **2a**–**j**. A methyl and a halogen substituent were tolerated in the *para* position of the aryl ring with moderate to good yields for **3ab**–**ae** throughout. A *tert*‐butyl group led to a diminished yield for **3af**. Although a low yield was obtained, the electron‐rich methoxy‐containing product **3ag** could be obtained in a 29% yield; the reduced yield may be attributed to Lewis adduct formation between the oxygen atom of the methoxy group and the silylium ion. The electron‐deficient trifluoromethyl‐substituted **3ah** could also be formed in a 29% yield; in this case, the low yield is likely caused by fluoride‐ion abstraction from the trifluoromethyl group by the silylium ion. Products bearing substitution at the *ortho‐* and *meta*‐positions could also be synthesized with **3ai** and **3aj** obtained in 39% and 51% yield, respectively. Dialkyl sulfides such as **2k** were incompatible under the current reaction conditions (gray box); degradation of the dialkyl starting material was observed, and the unreacted alkyne was recovered. It is believed that the formed alkyl‐substituted sulfonium ions are insufficiently stable.

**SCHEME 3:**
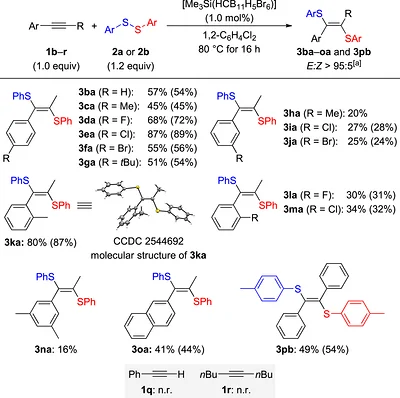
Substrate Scope II: Variation of the internal alkyne. All reactions were performed using the alkyne **1** (0.20 mmol), the indicated disulfide **2a** or **2b** (0.24 mmol, 1.2 equiv), and the initiator [Me_3_Si(HCB_11_H_5_Br_6_)] (2.0 µmol, 1.0 mol%) in a glovebox under an argon atmosphere in 1,2‐C_6_H_4_Cl_2_ (0.5 mL) at 80°C. Isolated yields refer to analytically pure material after flash chromatography on silica gel; yields in parentheses determined by ^1^H NMR spectroscopy of the crude reaction mixture using CH_2_Br_2_ as an internal standard. (a) *E:Z* > 95:5 of **3** in all cases as verified by ^1^H NMR spectroscopy of the crude reaction mixture.

We next focused on varying the alkyne (Scheme 3). Substitution in the *para‐*position of the aryl ring was modified systematically with moderate to good yields for a number of alkyl groups and halide substituents (**3ba**–**ga**; 45%−87% yield). A substantial drop in yield was observed when substitution on the aryl ring was introduced in the *meta* position (**3ha**–**ja**; 20%–27% yield). An improved yield of 80% was obtained when an alkyne bearing an *ortho*‐tolyl ring was used as in **3ka**. However, this trend did not persist when other *ortho*‐substituted rings were used, with low yields of 30% and 34% obtained for **3la** and **3ma**, respectively. Unsurprisingly, a poor yield of 16% was isolated for product bearing a *meta*‐xylyl ring. In turn, the steric bulk present from a β‐naphthyl group did not have a detrimental effect, with a moderate yield of 41% for **3oa**. Diaryl alkynes are also applicable to this reaction, and a moderate yield of 49% was obtained for **3pb**. However, terminal and dialkyl‐substituted alkynes such as **1q** and **1r** were incompatible under these reaction conditions (gray box); in both cases, the unreacted alkyne and disulfide starting materials were recovered. The *E*/*Z* stereochemistry of the reaction products was assigned by reference to the molecular structure of **3ka** (Supporting Information) [29].

**SCHEME 4:**
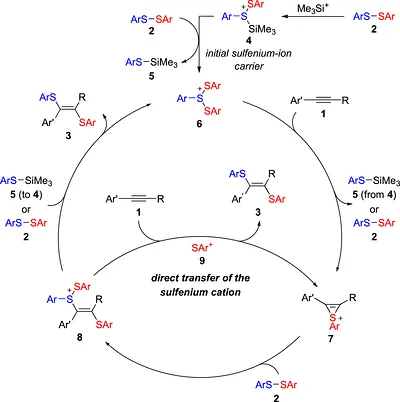
Proposed reaction mechanism. The [HCB_11_H_5_Br_6_]^−^ counterion is omitted for clarity.

While the mechanism bears similarities with that of the previously reported silylium‐ion‐initiated alkyne difunctionalizations (cf. Scheme 1, top) [5, 6], it is also distinctly different with regard to the propagating cationic intermediate. As demonstrated by Matsumoto, Yoshida, and co‐workers, sulfonium ion ArS(ArSSAr)^+^ (= intermediate **6**) can actually maintain the catalytic turnover [7] and is therefore a competent alternative to initially formed Me_3_Si(ArSSAr)^+^ **4** (Scheme 4). The latter is generated from [Me_3_Si(HCB_11_H_5_Br_6_)] and diaryl disulfide **2** in the initiation step. This sulfonium ion formally releases a sulfenium ion to react with the C≡C triple bond in **1**. The resulting thiirenium ion **7** [7] is then opened with another molecule of the disulfide **2**, also setting the *trans* relationship of the two sulfur moieties. The thus‐formed vinyl‐substituted sulfonium ion **8** can either directly transfer the sulfenium ion **9** to alkyne **1** or, in accordance with the existing *cationic chain mechanism*, involve either the disulfide **2** or the minute amount of thiosilane **5** as a sulfenium‐ion shuttle. The sulfonium ion ArS(ArSSAr)^+^ **6** is likely the propagating cation. Either of these steps leads to the formation of 1,2‐bis(arylthio)‐substituted alkene derivative **3**.

**SCHEME 5:**
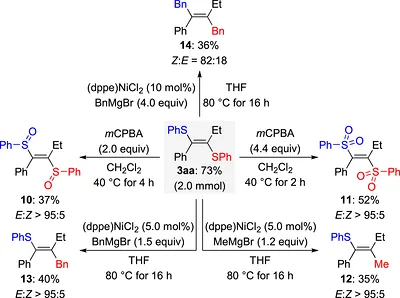
Scale up and further functionalizations. *m*CPBA, *meta*‐chloroperbenzoic acid; dppe,1,2‐bis(diphenylphosphino)ethane.

To explore a few typical follow‐up reactions, the model reaction was performed on a 2.0 mmol scale to give the alkene **3aa** in 73% isolated yield (Scheme 5). Following a modified literature procedure [5], both vinyl sulfide groups could be oxidized to the bis(sulfoxide) **10** in 37% yield. By increasing the equivalents of the peracid oxidant, it was possible to fully oxidize **3aa** to the bis(sulfone) **11** in 52% yield. A typical nickel‐catalyzed cross‐coupling [30] also enabled the regioselective methylation and benzylation of bis(sulfide) **3aa** to furnish **12** and **13** in moderate yields. Again, an increase in the equivalents of the Grignard reagent provided the bisbenzylated product **14** in 36% yield. However, partial isomerization of the desired product occurred to give **14** with a *Z*:*E* ratio of 82:18; the configuration of (*Z*)‐(3‐ethylbut‐2‐ene‐1,2,4‐triyl)tribenzene (**14**) was assigned by a nuclear Overhauser effect (nOe) measurement (Figure S2).

## 3 Conclusion

We have disclosed herein an alternative method for the stereoselective disulfidation of internal alkynes following an ionic mechanism through the intermediacy of a thiirenium ion. The reaction is initiated by the interaction between a silylium ion and a disulfide to generate a sulfonium ion as a sulfenium‐ion carrier. However, the actual sulfenium‐ion shuttle is ArS(ArSSAr)^+^, making this process an unusual example of silylium‐ion initiation without regeneration of the silylium ion. A range of dithiolated alkenes was obtained in moderate to good yields.

## 4 Experimental Section

### 4.1 Representative Dithiolation Procedure

In an argon‐filled glovebox, a 10 mL vial equipped with a stirbar was charged with alkyne **1a** (0.26 g, 2.0 mmol, 1.0 equiv) and disulfide **2a** (0.52 g, 2.4 mmol, 1.2 equiv), followed by the addition of 1,2‐C_6_H_4_Cl_2_ (5.0 mL). [Me_3_Si(HCB_11_H_5_Br_6_)] (14 mg, 0.020 mmol, 1.0 mol%) was subsequently added. The resulting reaction mixture was heated to 80°C and maintained at this temperature for 16 h. Upon completion, the reaction mixture was removed from the glovebox, and all volatiles were removed under reduced pressure. Purification by flash column chromatography on silica gel using *n*‐pentane/CH_2_Cl_2_ (20:1) as the eluent afforded the 1,2‐bis(phenylthio)‐substituted alkene **3aa** in analytically pure form as a yellow oil (0.51 g, 1.47 mmol, 73% yield).

## Conflicts of Interest

The authors declare no conflicts of interest.

## Supporting information




**Supporting File**: Additional supporting information can be found online in the Supporting Information section. Additional references cited within the supporting information [31, 32, 33, 34, 35, 36, 37].

## Data Availability

The data that support the findings of this study are available in the Supporting Information or from the corresponding author upon reasonable request.
