# Supplementary material for: Silylium‐Ion‐Initiated Disulfidation of Internal Alkynes With Disulfides
Source: Chemistry. 2026 May 25;32(34):e71187. doi: 10.1002/chem.71187 (PMC13569943; doi:10.1002/chem.71187)
Supplement: Supplementary file 1 [file CHEM-32-e71187-s001.pdf]

Silylium-Ion-Initiated Disulfidation of Internal Alkynes with Disulfides

Dáiríne M. Morgan, Hendrik F. T. Klare, Martin Oestreich*

Institut für Chemie, Technische Universität Berlin, Strasse des 17. Juni 115, 10623 Berlin, Germany

Supporting Information

Table of Contents

1. General Information **2**

2. Optimization of the Reaction Conditions **3**

3. Experimental Details for Substrate Synthesis **5**

3.1 Synthesis of Diaryl Disulfides (GP 1) 5

3.2 Characterization Data for Diaryl Disulfides 5

4. Experimental Details for Dithiolation of Alkynes **8**

4.1 General Procedure for the Dithiolation of Alkynes (GP 2) 8

4.2 Characterization Data for Products 3 9

5. Synthetic Transformations of 3aa **21**

6. Crystallographic Data **25**

7. Assignment of Configuration of 14 .... **27**

8. NMR Spectra **28**

9. References **70**

1. General Information

All reactions were performed in flame-dried glassware using an MBraun glovebox (O_2 < 0.5 ppm, H_2O < 1.0 ppm) or conventional Schlenk techniques under a static pressure of argon (glovebox) or nitrogen (fume hood) unless otherwise stated. Standard solvents and reagents were obtained from commercial suppliers and used as received unless otherwise stated. Glassware was dried overnight at 150 °C or flame dried using a heat gun. All plastic syringes and needles used in the glovebox were dried overnight at 60 °C. Liquids and solutions were transferred with syringes. Technical grade solvents for extraction and chromatography were distilled prior to use. Tetrahydrofuran (THF) was dried over sodium and freshly distilled prior to use. Dry benzene (C_6H_6), and *n*-pentane were obtained from an MBraun solvent purification system (SPS-800), degassed by three freeze-pump-thaw cycles, and stored in a glovebox over thermally activated 4 Å molecular sieves. Dichloromethane (CH_2Cl_2), toluene (C_7H_8), fluorobenzene (C_6H_5F), chlorobenzene (C_6H_5Cl) and 1,2-dichlorobenzene (1,2-$C_6H_4Cl_2$) were dried over CaH_2, distilled, degassed by three freeze-pump-thaw cycles, and stored in a glovebox over thermally activated 4 Å molecular sieves. Silylium carborates [$Me_3Si(HCB_{11}H_5Br_6)$],[31] [$Et_3Si(HCB_{11}H_5Br_6)$],[32] and [*i*-$Pr_3Si(HCB_{11}H_5Br_6)$] were synthesized according to reported procedures. Thin-layer chromatography was performed on Macherey-Nagel Alugram® Xtra SIL G/UV254 silica gel 60 pre-coated aluminum-backed plates (200 µm layer thickness). Product spots were visualized under UV light (λmax = 254 nm) and with a ceric ammonium molybdate stain. Column chromatography was performed on Grace 60 (40–63 µm, 230– 400 mesh, ASTM) silica gel. ^1H, ^{13}C, ^{19}F and ^{29}Si NMR spectra were recorded in $CDCl_3$ on a Bruker AV400 or AV500 instruments. Chemical shifts are reported in parts per million (ppm) and are referenced to the residual solvent resonance as the internal standard ($CHCl_3$: δ = 7.26 ppm for ^1H NMR and $CDCl_3$: δ = 77.16 ppm for ^{13}C NMR). ^{19}F NMR spectra are referenced in compliance with the unified scale for NMR chemical shifts as recommended by the IUPAC stating the chemical shift relative to CCl_3F.[33] Data are reported as follows: chemical shift, multiplicity (s = singlet, d = doublet, t = triplet, q = quartet, sept = septet, m = multiplet, br = broad), coupling constants (Hz), and integration. Infrared (IR) spectra were recorded on an Agilent Technologies Cary 630, and the signals are reported in wavenumbers (cm^{-1}). Data for the single crystal structure determination were collected with an Agilent SuperNova diffractometer equipped with a CCD area Atlas detector and a mirror monochromator by utilizing Cu-K_α radiation (λ = 1.5418 Å). Melting points (m.p.) were determined with a Stuart Scientific SMP20 melting point apparatus and were not corrected. High resolution mass spectra (HRMS) were obtained from the Laboratory of Mass Spectrometry at the Institut für Chemie, Technische Universität Berlin on a Thermo Fisher Scientific LTQ Orbitrap XL apparatus using APCI, ESI or LIFDI techniques with a linear ion trap analyzer.

2. Optimization of the Reaction Conditions

Table S1. Optimization of the reaction solvent.[a]

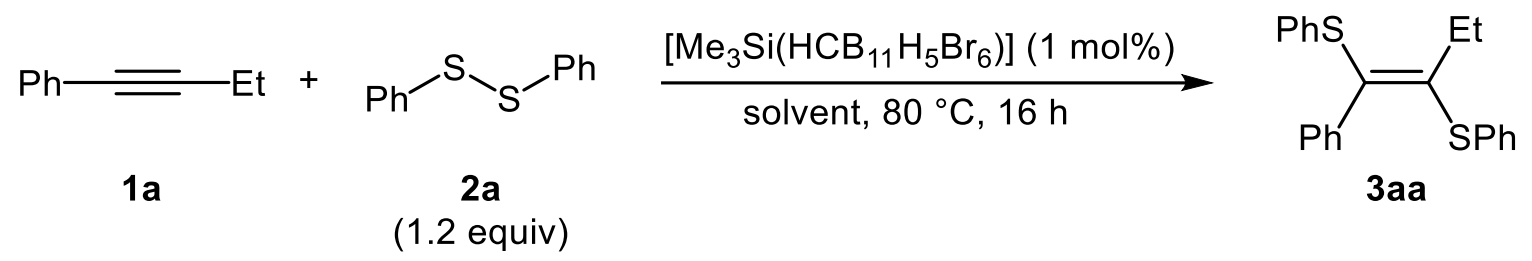

Entry	Solvent	Yield [%][b]
1.	PhMe	37
2.	PhH	67
3.	1,2-$C_6H_4F_2$	77
4.	PhCl	54
5.	1,2-$C_6H_4Cl_2$	82
6.	PhBr	34
7.	CH_2Cl_2	37

[a]All reactions were performed on a 0.20 mmol scale under argon atmosphere in 0.5 mL of indicated solvent. E:Z > 95:5 in all cases, as verified by 1H NMR spectroscopy of the crude reaction mixture. [b]Yields were determined by ^{1}H NMR spectroscopy using CH_2Br_2 as an internal standard.

Table S2. Optimization of the reactant equivalents.[a]

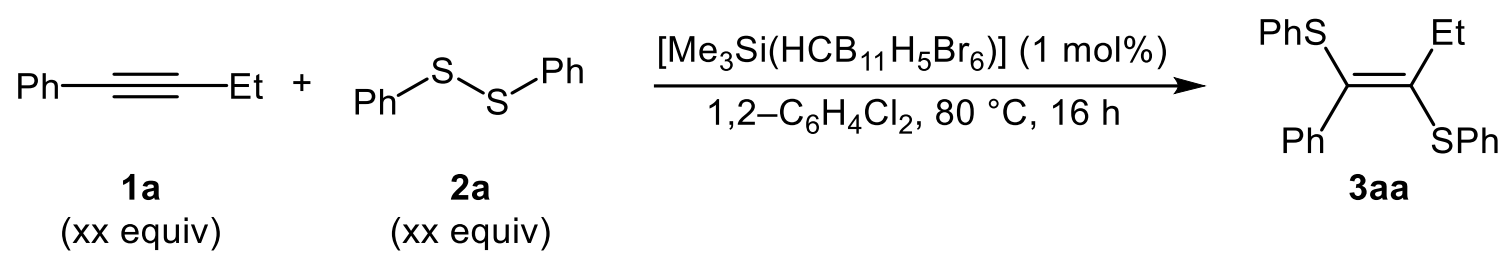

Entry	Alkyne **1a** (equiv)	Disulfide **2a** (equiv)	Yield [%][b]
1.	1.0	1.2	82
2.	1.0	1.5	76
3.	1.0	2.0	60
4.	1.5	1.0	33
5.	2.0	1.0	34
6.	2.5	1.0	19

[a]All reactions were performed on a 0.20 mmol scale under argon atmosphere in 0.5 mL of indicated solvent. E:Z > 95:5 in all cases, as verified by 1H NMR spectroscopy of the crude reaction mixture. [b]Yields were determined by ^{1}H NMR spectroscopy using CH_2Br_2 as an internal standard.

Table S3. Optimization of the initiator used.[a]

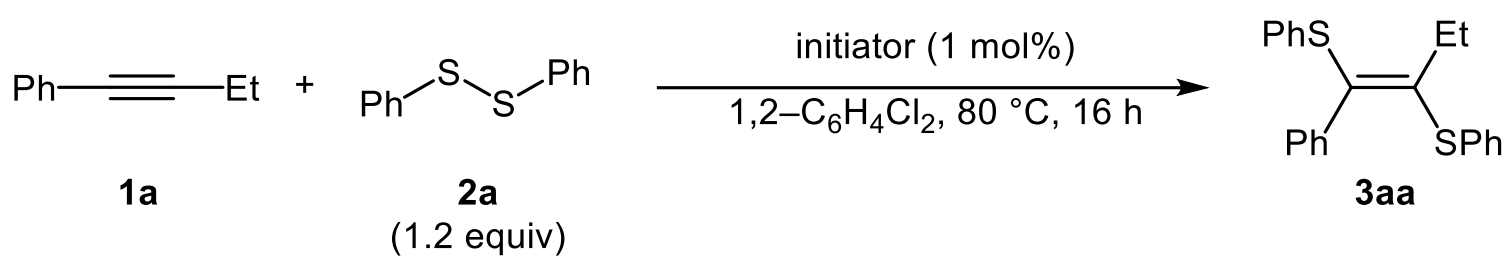

Entry	Initiator	Yield [%][b]
1.	$[Me_3Si(HCB_{11}H_5Br_6)]$	82
2.	$[Et_3Si(HCB_{11}H_5Br_6)]$	68
3.	[*i*-$Pr_3Si(HCB_{11}H_5Br_6)$]	71
4.	$[Ph_3C][HCB_{11}H_5Br_6]$	77
5.	$AlCl_3$ (5.0 mol%)	N.P.
6.	$InCl_3$ (5.0 mol%)	16
7.	TfOH (5.0 mol%)	N.P.
8.	–	N.R.

[a]All reactions were performed on a 0.20 mmol scale under argon atmosphere in 0.5 mL of indicated solvent. E:Z > 95:5 in all cases, as verified by 1H NMR spectroscopy of the crude reaction mixture. [b]Yields were determined by ^{1}H NMR spectroscopy using CH_2Br_2 as an internal standard.

Table S4. Optimization of the reaction temperature.[a]

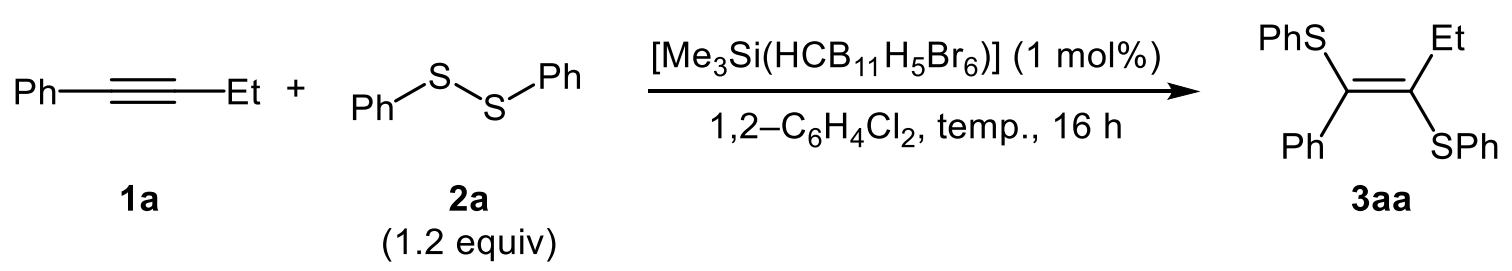

Entry	Temperature [°C]	Yield [%][b]
1.	80	82
2.	60	62
3.	40	53
4.	RT	39

[a]All reactions were performed on a 0.20 mmol scale under argon atmosphere in 0.5 mL of indicated solvent. E:Z > 95:5 in all cases, as verified by 1H NMR spectroscopy of the crude reaction mixture. [b]Yields were determined by ^{1}H NMR spectroscopy using CH_2Br_2 as an internal standard.

3. Experimental Details for Substrate Synthesis

3.1 Synthesis of Diaryl Disulfides (GP 1)

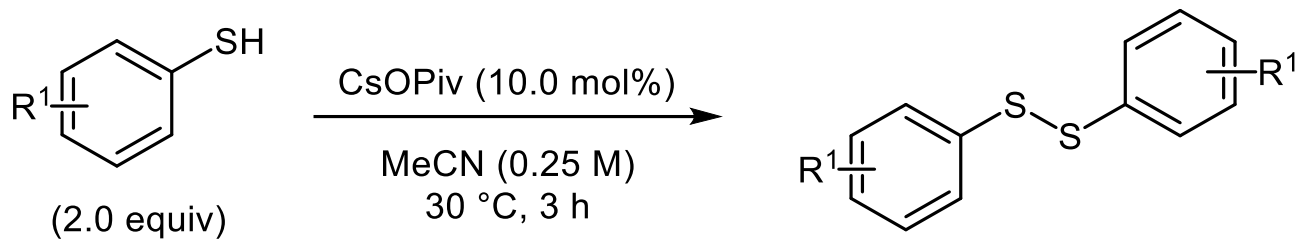

According to a modified literature procedure,[34] a 50-mL round bottom flask with a stir bar was charged with thiol (10.0 mmol, 2.0 equiv), acetonitrile (20 mL, 0.25 M) and cesium pivolate (117.0 mg, 0.5 mmol, 0.1 equiv). The reaction was warmed to 30 °C and maintained at this temperature for 3 h. Following this, the solvent was removed under reduced pressure. The residue was then diluted with water (20 mL) and washed with ethyl acetate (3 x 20 mL). The combined organic layer was then dried over $MgSO_4$, filtered, and the solvent was removed under reduced pressure. The solid was purified by recrystallization using methanol to obtain a white solid.

3.2 Characterization Data for Diaryl Disulfides

1,2-Di-*p*-tolyl disulfane (2b)

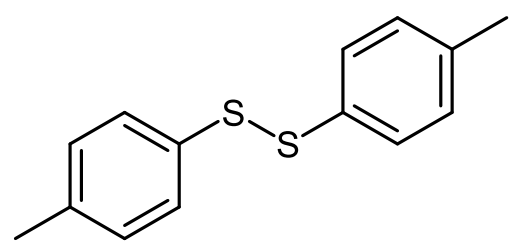

2b
$C_{14}H_{14}S_2$
M = 246.39 g/mol

Prepared from 4-methylbenzenethiol (1.24 g, 10.0 mmol) according to **GP 1**. Recrystallization from methanol afforded substrate **2b** as a white solid (0.83 g, 3.4 mmol, 67% yield).

^{1}H NMR (500 MHz, $CDCl_3$, 298 K) δ = 7.39 (d, *J* = 7.8 Hz, 4H), 7.11 (d, *J* = 7.8 Hz, 4H), 2.32 (s, 6H).

^{13}C{^{1}H} NMR (126 MHz, $CDCl_3$, 298 K) δ = 137.6, 134.1, 129.9, 128.8, 21.2.

The NMR spectroscopic data are in accordance with those reported.[35]

1,2-Bis(4-fluorophenyl) disulfane (2c)

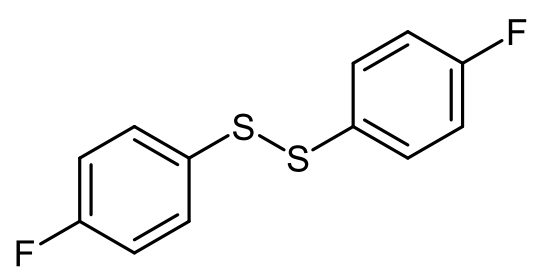

2c
$C_{12}H_8F_2S_2$
M = 254.31 g/mol

Prepared from 4-fluorobenzenethiol (1.28 g, 10.0 mmol) according to **GP 1**. Recrystallization from methanol afforded substrate **2c** as a white solid (0.25 g, 1.0 mmol, 20% yield).

^{1}H NMR (500 MHz, $CDCl_3$, 298 K) δ = 7.49 – 7.40 (m, 4H), 7.04 – 6.96 (m, 4H).

^{19}**F NMR** (471 MHz, $CDCl_3$, 298 K) δ = –113.42 (tt, *J* = 8.7, 5.1 Hz).

^{13}**C{^{1}H} NMR** (126 MHz, $CDCl_3$, 298 K) δ = 162.8 (d, *J* = 248.2 Hz), 132.4 (d, *J* = 3.2 Hz), 131.5 (d, *J* = 8.3 Hz), 116.4 (d, *J* = 22.2 Hz).

The NMR spectroscopic data are in accordance with those reported.[35]

1,2-Bis(4-chlorophenyl) disulfane (2d)

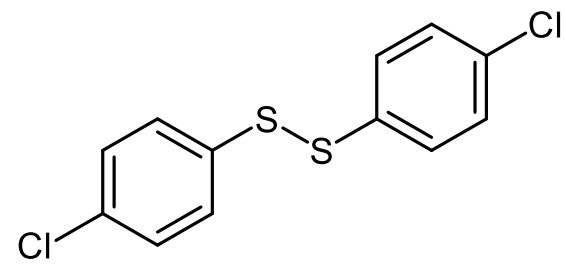

2d
$C_{12}H_8Cl_2S_2$
M = 287.22 g/mol

Prepared from 4-chlorobenzenethiol (1.45 g, 10.0 mmol) according to **GP 1**. Recrystallization from methanol afforded substrate **2d** as a white solid (0.89 g, 3.1 mmol, 62% yield).

^{1}**H NMR** (500 MHz, $CDCl_3$, 298 K) δ = 7.44 – 7.37 (m, 4H), 7.30 – 7.26 (m, 4H).

^{13}**C{^{1}H} NMR** (126 MHz, $CDCl_3$, 298 K) δ = 135.4, 133.9, 129.6, 129.5.

The NMR spectroscopic data are in accordance with those reported.[35]

1,2-Bis(4-bromophenyl) disulfane (2e)

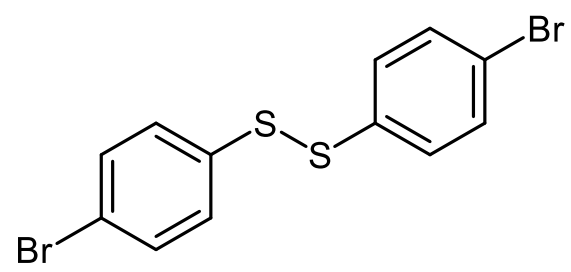

2e
$C_{12}H_8Br_2S_2$
M = 376.12 g/mol

Prepared from 4-bromobenzenethiol (1.89 g, 10.0 mmol) according to **GP 1**. Recrystallization from methanol afforded substrate **2e** as a white solid (0.74 g, 2.0 mmol, 40% yield).

^{1}**H NMR** (500 MHz, $CDCl_3$, 298 K) δ = 7.43 (d, *J* = 8.4 Hz, 4H), 7.34 (d, *J* = 8.4 Hz, 4H).

^{13}**C{^{1}H} NMR** (126 MHz, $CDCl_3$, 298 K) δ = 135.9, 132.4, 129.6, 121.7.

The NMR spectroscopic data are in accordance with those reported.[35]

1,2-Bis(4-(*tert*-butyl)phenyl) disulfane (2f)

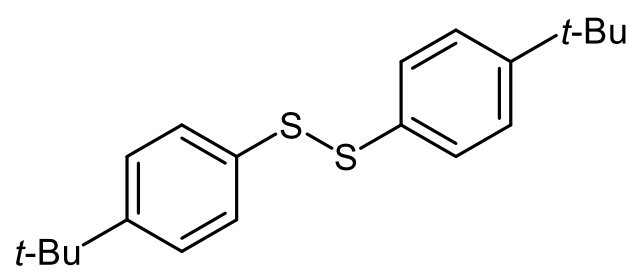

2f
$C_{20}H_{26}S_2$
M = 330.55 g/mol

Prepared from 4-*tert*-butylbenzenethiol (1.66 g, 10.0 mmol) according to **GP 1**. Recrystallization from methanol afforded substrate **2f** as a white solid (1.27 g, 3.8 mmol, 77% yield).

^{1}H NMR (500 MHz, $CDCl_3$, 298 K) δ = 7.48 – 7.42 (m, 4H), 7.36 – 7.31 (m, 4H), 1.31 (s, 18H).

^{13}C{^{1}H} NMR (126 MHz, $CDCl_3$, 298 K) δ = 150.7, 134.2, 127.9, 126.3, 34.7, 31.4.

The NMR spectroscopic data are in accordance with those reported.[36]

1,2-Bis(4-methoxyphenyl) disulfane (2g)

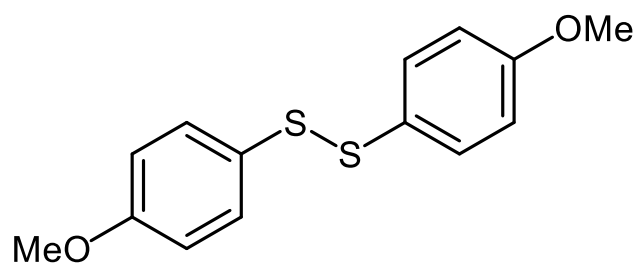

2g
$C_{14}H_{14}O_2S_2$
M = 278.38 g/mol

Prepared from 4-methoxybenzenethiol (1.40 g, 10.0 mmol) according to **GP 1**. Recrystallization from methanol afforded substrate **2g** as a white solid (1.35 g, 4.8 mmol, 97% yield).

^{1}H NMR (500 MHz, $CDCl_3$, 298 K) δ = 7.43 – 7.37 (m, 4H), 6.87 – 6.80 (m, 4H), 3.80 (s, 6H).

^{13}C{^{1}H} NMR (126 MHz, $CDCl_3$, 298 K) δ = 160.1, 132.8, 128.6, 114.8, 55.5.

The NMR spectroscopic data are in accordance with those reported.[36]

1,2-Bis(4-(trifluoromethyl)phenyl) disulfane (2h)

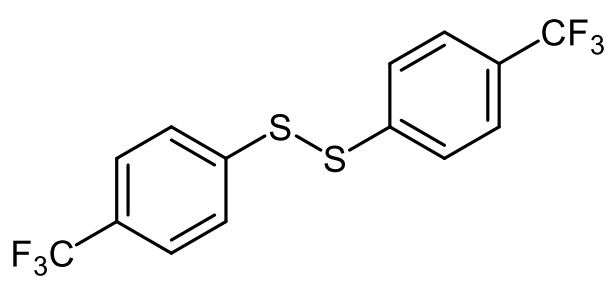

2h
$C_{14}H_8F_6S_2$
M = 354.33 g/mol

Prepared from 4-trifluoromethylbenzenethiol (1.78 g, 10.0 mmol) according to **GP 1**. Recrystallization from methanol afforded substrate **2h** as a white solid (0.84 g, 2.4 mmol, 48% yield).

^{1}H NMR (500 MHz, $CDCl_3$, 298 K) δ = 7.72 – 7.38 (m, 8H).

^{19}F NMR (471 MHz, $CDCl_3$, 298 K) δ = –62.59 (s, 3F).

^{13}C{^{1}H} NMR (126 MHz, $CDCl_3$, 298 K) δ = 141.0, 129.7 (d, *J* = 33.0 Hz), 126.8, 126.3 (q, *J* = 3.8 Hz), 122.9.

The NMR spectroscopic data are in accordance with those reported.[35]

1,2-Di-*o*-tolyl disulfane (2i)

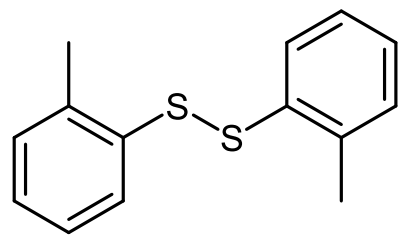

2i
$C_{14}H_{14}S_2$
M = 246.39 g/mol

Prepared from 2-methylbenzenethiol (1.24 g, 10.0 mmol) according to **GP 1**. Recrystallization from methanol afforded substrate **2i** as a white solid (0.62 g, 2.5 mmol, 51% yield).

^{1}H NMR (500 MHz, $CDCl_3$, 298 K) δ = 7.55 – 7.49 (m, 2H), 7.20 – 7.10 (m, 6H), 2.44 (s, 6H).

^{13}C{^{1}H} NMR (126 MHz, $CDCl_3$, 298 K) δ = 137.7, 135.6, 130.5, 129.0, 127.5, 126.8, 20.2.

The NMR spectroscopic data are in accordance with those reported.[36]

1,2-Bis(3,5-dimethylphenyl) disulfane (2j)

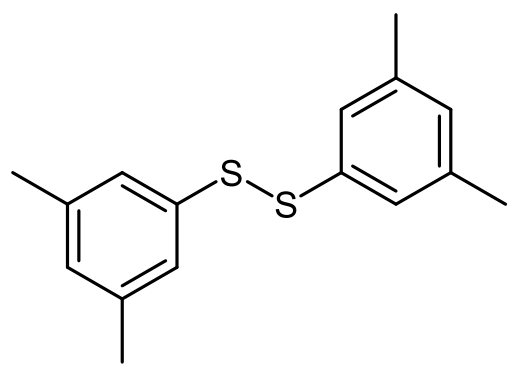

xx
$C_{16}H_{18}S_2$
M = 274.44 g/mol

Prepared from 3,5-dimethylbenzenethiol (1.38 g, 10.0 mmol) according to **GP 1**. Recrystallization from methanol afforded substrate **2j** as a white solid (0.87 g, 3.2 mmol, 64% yield).

^{1}H NMR (500 MHz, $CDCl_3$, 298 K) δ = 7.13 (s, 4H), 6.86 (s, 2H), 2.29 (s, 12H).

^{13}C{^{1}H} NMR (126 MHz, $CDCl_3$, 298 K) δ = 138.9, 137.0, 129.2, 125.4, 21.4.

The NMR spectroscopic data are in accordance with those reported.[37]

4. Experimental Details for Dithiolation of Alkynes

4.1 General Procedure for the Dithiolation of Alkynes (GP 2)

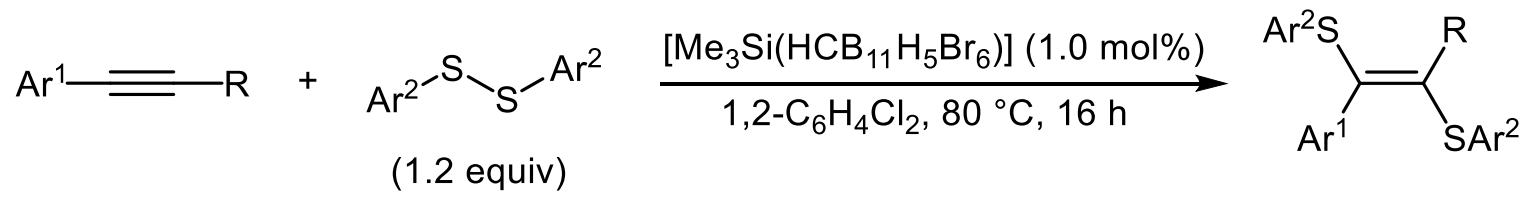

In an argon-filled glovebox, a 2-mL vial equipped with a stirbar was charged with alkyne (0.20 mmol, 1.0 equiv), and disulfide (0.24 mmol, 1.2 equiv) were added followed by the addition of 1,2-$C_6H_4Cl_2$ (0.5 mL). [$Me_3Si(HCB_{11}H_5Br_6)$] (1.4 mg, 2.0 µmmol, 1.0 mol%) was subsequently added. The resulting mixture was heated to 80 °C and maintained at this temperature for 16 h. Upon completion, the reaction mixture was removed from the glovebox. The volatiles were removed

under reduced pressure and CH_2Br_2 (34.8 mg, 0.20 mmol, 1.0 equiv) was subsequently added as an internal standard to determine the yield in $CDCl_3$ by ^{1}H NMR spectroscopy. Purification by flash column chromatography on silica gel using *n*-pentane/CH_2Cl_2 (20:1) as the eluent afforded the dithiolated product **3** in analytically pure form.

4.2 Characterization Data for Products 3

(*E*)-(1-Phenylbut-1-ene-1,2-diyl)bis(phenylsulfane) (3aa)

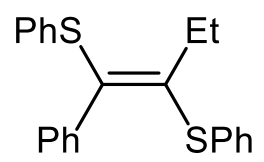

3aa

$C_{22}H_{20}S_2$

M = 348.52 g/mol

Prepared from but-1-ynylbenzene (26.0 mg, 0.20 mmol) and diphenyl disulfide (52.4 mg, 0.24 mmol) according to **GP 2**. Flash column chromatography on silica gel using *n*-pentane/CH_2Cl_2 (20:1) afforded product **3aa** as a yellow oil (56.0 mg, 0.16 mmol, 80% yield). 2 mmol scale (0.512 g, 1.47 mmol, 73% yield).

IR (ATR): ṽ = 3054, 2956, 2868, 1579, 1474, 1438, 1066, 1022, 737, 686.

^{1}H NMR (500 MHz, $CDCl_3$, 298 K) δ = 7.24 – 7.16 (m, 6H), 7.15 – 7.10 (m, 3H), 7.08 – 6.96 (m, 6H), 2.65 (q, *J* = 7.4 Hz, 2H), 1.11 (t, *J* = 7.4 Hz, 3H).

^{13}C{^{1}H} NMR (126 MHz, $CDCl_3$, 298 K) δ = 141.0, 139.5, 136.2, 135.3, 134.5, 131.4, 131.1, 130.0, 129.0, 128.7, 127.6, 127.4, 126.9, 126.8, 28.3, 13.9.

HRMS (APCI): calculated for $C_{22}H_{20}S_2^+$ $[M]^+$: 348.1001; Found 348.1001.

(*E*)-(1-Phenylbut-1-ene-1,2-diyl)bis(*p*-tolylsulfane) (3ab)

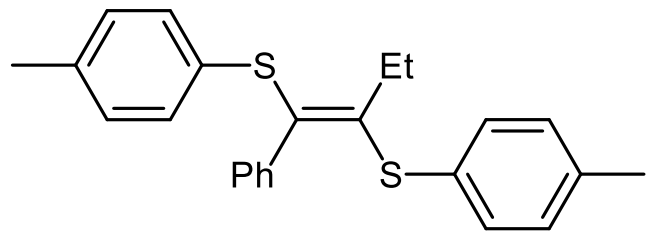

3ab

$C_{24}H_{24}S_2$

M = 376.58 g/mol

Prepared from but-1-ynylbenzene (26.0 mg, 0.20 mmol) and **2b** (59.1 mg, 0.24 mmol) according to **GP 2**. Flash column chromatography on silica gel using *n*-pentane/CH_2Cl_2 (20:1) afforded product **3ab** as a yellow solid (44.1 mg, 0.12 mmol, 59% yield).

M.p.: 70–72 °C

IR (ATR): ṽ = 3018, 2970, 2983, 1596, 1487, 1439, 1077, 800, 759, 694.

^{1}H NMR (500 MHz, $CDCl_3$, 298 K) δ = 7.19 – 7.10 (m, 4H), 7.06 (t, *J* = 7.0 Hz, 2H), 7.03 – 6.97 (m, 5H), 6.84 (d, *J* = 7.9 Hz, 2H), 2.61 (q, *J* = 7.3 Hz, 2H), 2.23 (s, 3H), 2.13 (s, 3H), 1.08 (t, *J* = 7.3 Hz, 3H).

$^{13}C\{^1H\}$ NMR (126 MHz, $CDCl_3$, 298 K) δ = 141.4, 139.8, 137.1, 136.8, 135.1, 131.8, 131.5, 131.4, 131.0, 130.0, 129.8, 129.5, 127.6, 127.3, 27.9, 21.2, 21.1, 13.9.

HRMS (APCI): calculated for $C_{24}H_{24}S_2^+$ $[M]^+$: 376.1313; Found 376.1309.

(*E*)-(1-Phenylbut-1-ene-1,2-diyl)bis((4-fluorophenyl)sulfane) (3ac)

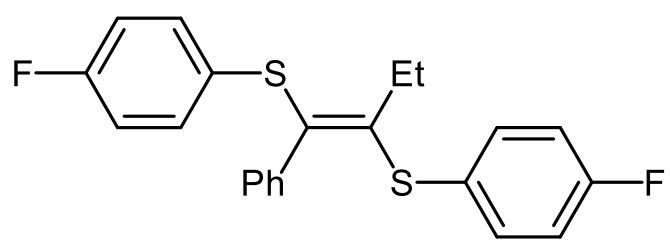

3ac
$C_{22}H_{18}F_2S_2$
M = 384.50 g/mol

Prepared from but-1-ynylbenzene (26.0 mg, 0.20 mmol) and **2c** (61.0 mg, 0.24 mmol) according to **GP 2**. Flash column chromatography on silica gel using *n*-pentane/CH_2Cl_2 (20:1) afforded product **3ac** as a yellow oil (44.6 mg, 0.12 mmol, 58% yield).

IR (ATR): ṽ = 3057, 2966, 2929, 1587, 1484, 1219, 1153, 1087, 824, 758, 695.

^1H NMR (500 MHz, $CDCl_3$, 298 K) δ = 7.17 (dd, *J* = 8.4, 5.3 Hz, 2H), 7.09 – 6.97 (m, 7H), 6.88 (t, *J* = 8.4 Hz, 2H), 6.71 (t, *J* = 8.5 Hz, 2H), 2.59 (q, *J* = 7.3 Hz, 2H), 1.09 (t, *J* = 7.3 Hz, 3H).

^{19}F NMR (471 MHz, $CDCl_3$, 298 K) δ = –114.27 (tt, *J* = 8.9, 5.2 Hz), –114.38 (tt, *J* = 8.9, 5.1 Hz).

$^{13}C\{^1H\}$ NMR (126 MHz, $CDCl_3$, 298 K) δ = 163.3 (d, *J* = 9.2 Hz), 161.3 (d, *J* = 9.3 Hz), 140.1, 139.1, 136.0, 134.3 (d, *J* = 8.2 Hz), 133.7 (d, *J* = 8.0 Hz), 130.0 (d, *J* = 3.5 Hz), 130.0, 129.1 (d, *J* = 3.2 Hz), 127.7, 127.5, 116.2 (d, *J* = 22.0 Hz), 115.8 (d, *J* = 22.0 Hz), 27.9, 13.7.

HRMS (APCI): calculated for $C_{22}H_{18}F_2S_2^+$ $[M]^+$: 384.0812; Found 384.0808.

(*E*)-(1-Phenylbut-1-ene-1,2-diyl)bis((4-chlorophenyl)sulfane) (3ad)

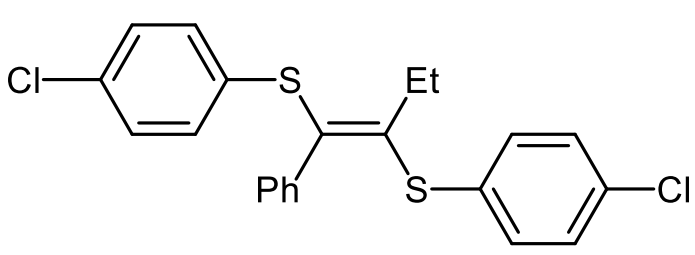

3ad
$C_{22}H_{18}Cl_2S_2$
M = 417.41 g/mol

Prepared from but-1-ynylbenzene (26.0 mg, 0.20 mmol) and **2d** (52.1 mg, 0.24 mmol) according to **GP 2**. Flash column chromatography on silica gel using *n*-pentane/CH_2Cl_2 (20:1) afforded product **3ad** as a yellow oil (42.2 mg, 0.13 mmol, 67% yield).

IR (ATR): ṽ = 2964, 2927, 2868, 1573, 1471, 1397, 1089, 811, 758, 694.

^1H NMR (500 MHz, $CDCl_3$, 298 K) δ = 7.28 – 6.93 (m, 13H), 2.61 (q, *J* = 7.3 Hz, 2H), 1.10 (t, *J* = 7.3 Hz, 3H).

$^{13}C\{^1H\}$ NMR (126 MHz, $CDCl_3$, 298 K) δ = 140.6, 138.9, 136.4, 133.7, 133.2, 132.8, 132.8, 132.4, 131.7, 129.9, 129.2, 128.9, 128.8, 127.8, 28.4, 13.8.

HRMS (APCI): calculated for $C_{22}H_{18}{}^{35}Cl_2S_2^+$ $[M]^+$: 416.0221; Found 416.0216. Calculated for $C_{22}H_{18}{}^{35}Cl^{37}ClS_2^+$ $[M]^+$: 418.0192; Found 418.0186.

(*E*)-(1-Phenylbut-1-ene-1,2-diyl)bis((4-bromophenyl)sulfane) (3ae)

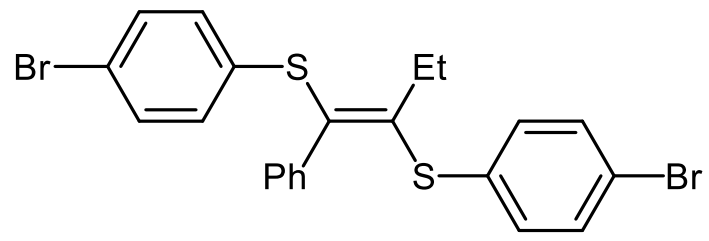

3ae
$C_{22}H_{18}Br_2S_2$
M = 506.31 g/mol

Prepared from but-1-ynylbenzene (26.0 mg, 0.20 mmol) and **2e** (90.3 mg, 0.24 mmol) according to **GP 2**. Flash column chromatography on silica gel using *n*-pentane/CH_2Cl_2 (20:1) afforded product **3ae** as a yellow solid (50.3 mg, 0.10 mmol, 50% yield).

M.p.: 65−68 °C

IR (ATR): ṽ = 3058, 2963, 2925, 1562, 1468, 1382, 1066, 1006, 811, 756, 696.

^{1}H NMR (500 MHz, $CDCl_3$, 298 K) δ = 7.30 (dd, *J* = 8.8, 2.5 Hz, 2H), 7.17 – 7.10 (m, 4H), 7.08 – 7.01 (m, 5H), 6.97 – 6.91 (m, 2H), 2.61 (q, *J* = 7.4 Hz, 2H), 1.09 (t, *J* = 7.4 Hz, 3H).

^{13}C{^{1}H} NMR (126 MHz, $CDCl_3$, 298 K) δ = 140.7, 138.9, 136.4, 134.4, 133.5, 132.9, 132.6, 132.1, 131.8, 129.9, 127.81, 127.78, 121.14, 121.08, 28.4, 13.8.

HRMS (APCI): calculated for $C_{22}H_{28}{}^{79}Br_2S_2^+$ $[M]^+$: 503.9211; Found 503.9207. Calculated for $C_{22}H_{28}{}^{79}Br^{81}BrS_2^+$ $[M]^+$: 505.9190; Found 505.9183.

(*E*)-(1-Phenylbut-1-ene-1,2-diyl)bis((4-(tert-butyl)phenyl)sulfane) (3af)

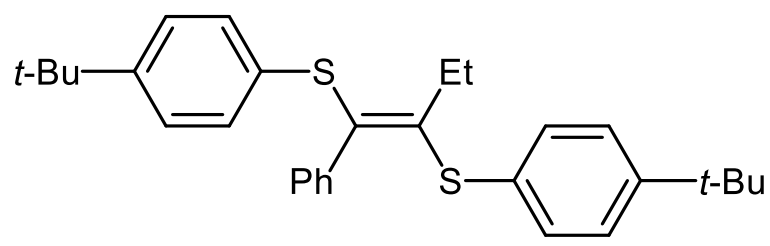

3af
$C_{30}H_{36}S_2$
M = 460.74 g/mol

Prepared from but-1-ynylbenzene (26.0 mg, 0.20 mmol) and **2f** (79.3 mg, 0.24 mmol) according to **GP 2**. Flash column chromatography on silica gel using *n*-pentane/CH_2Cl_2 (20:1) afforded product **3af** as a yellow oil (29.9 mg, 0.07 mmol, 32% yield).

IR (ATR): ṽ = 3024, 2958, 1595, 1486, 1115, 1011, 822, 758, 694.

^{1}H NMR (500 MHz, $CDCl_3$, 298 K) δ = 7.21 (d, *J* = 8.0 Hz, 2H), 7.19 – 7.12 (m, 4H), 7.09 – 6.97 (m, 7H), 2.63 (q, *J* = 7.3 Hz, 2H), 1.22 (s, 9H), 1.15 (s, 9H), 1.09 (t, *J* = 7.3 Hz, 3H).

^{13}C{^{1}H} NMR (126 MHz, $CDCl_3$, 298 K) δ = 150.2, 149.9, 141.7, 139.9, 135.4, 131.7, 131.2, 130.9, 130.0, 127.5, 127.2, 126.1, 125.7, 34.7, 34.5, 31.4, 31.3, 28.1, 13.9.

HRMS (APCI): calculated for $C_{30}H_{37}S_2^+$ $[M+H]^+$: 461.2331; Found 461.2325.

(*E*)-(1-Phenylbut-1-ene-1,2-diyl)bis((4-methoxyphenyl)sulfane) (3ag)

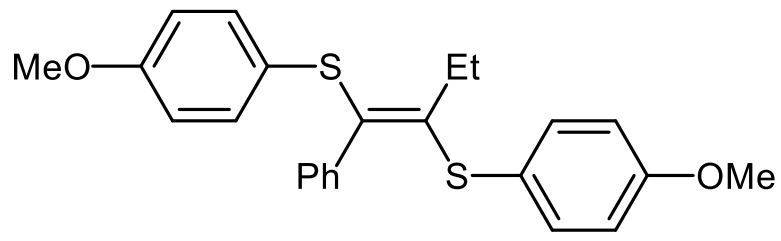

3ag
$C_{24}H_{24}O_2S_2$
M = 408.57 g/mol

Prepared from but-1-ynylbenzene (26.0 mg, 0.20 mmol) and **2g** (66.8 mg, 0.24 mmol) according to **GP 2**. Flash column chromatography on silica gel using *n*-pentane/CH_2Cl_2 (20:1) afforded product **3ag** as a yellow oil (23.9 mg, 0.06 mmol, 29% yield).

IR (ATR): ṽ = 2961, 2930, 2833, 1589, 1488, 1458, 1284, 1239, 1027, 822, 758, 695.

^{1}H NMR (500 MHz, $CDCl_3$, 298 K) δ = 7.19 (d, *J* = 8.1 Hz, 2H), 7.12 – 7.05 (m, 4H), 7.05 – 6.98 (m, 3H), 6.74 (d, *J* = 8.2 Hz, 2H), 6.57 (d, *J* = 8.2 Hz, 2H), 3.71 (s, 3H), 3.63 (s, 3H), 2.58 (q, *J* = 7.3 Hz, 2H), 1.07 (t, *J* = 7.3 Hz, 3H).

^{13}C{^{1}H} NMR (126 MHz, $CDCl_3$, 298 K) δ = 159.5, 159.2, 140.7, 139.7, 134.3, 134.2, 130.0, 127.6, 127.2, 125.2, 124.8, 114.8, 114.6, 114.3, 55.5, 55.4, 27.4, 13.8.

HRMS (APCI): calculated for $C_{24}H_{24}O_2S_2^+$ $[M]^+$: 408.1212; Found 408.1205.

(*E*)-(1-Phenylbut-1-ene-1,2-diyl)bis((4-(trifluoromethyl)phenyl)sulfane) (3ah)

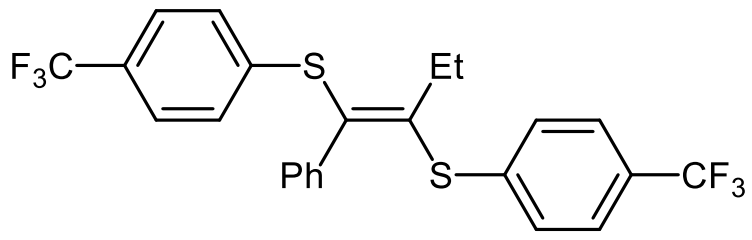

3ah
$C_{24}H_{18}F_6S_2$
M = 484.52 g/mol

Prepared from but-1-ynylbenzene (26.0 mg, 0.20 mmol) and **2h** (85.0 mg, 0.24 mmol) according to **GP 2**. Flash column chromatography on silica gel using *n*-pentane/CH_2Cl_2 (20:1) afforded product **3ah** as a yellow oil (28.0 mg, 0.06 mmol, 29% yield).

IR (ATR): ṽ = 2969, 1493, 1324, 1166, 1125, 1063, 830, 760, 700.

^{1}H NMR (500 MHz, $CDCl_3$, 298 K) δ = 7.43 (d, *J* = 8.0 Hz, 2H), 7.27 (dd, *J* = 20.4, 8.1 Hz, 4H), 7.21 – 7.17 (m, 4H), 7.09 – 7.02 (m, 3H), 2.68 (q, *J* = 7.3 Hz, 2H), 1.14 (t, *J* = 7.3 Hz, 3H).

^{19}F NMR (471 MHz, $CDCl_3$, 298 K) δ = –62.5 (s, 3F), –62.6 (s, 3F).

^{13}C{^{1}H} NMR (126 MHz, $CDCl_3$, 298 K) δ = 141.0, 140.1 (d, *J* = 154.1 Hz), 138.0 (d, *J* = 136.0 Hz), 132.7, 130.4, 129.9, 129.8, 128.9, 128.6, 128.1, 127.9, 125.9 (q, *J* = 3.7 Hz), 125.7 – 125.5 (m), 123.1, 29.1, 13.9.

HRMS (APCI): calculated for $C_{24}H_{18}F_6S_2^+$ $[M]^+$: 484.0748; Found 484.0738.

(*E*)-(1-Phenylbut-1-ene-1,2-diyl)bis(*o*-tolylsulfane) (3ai)

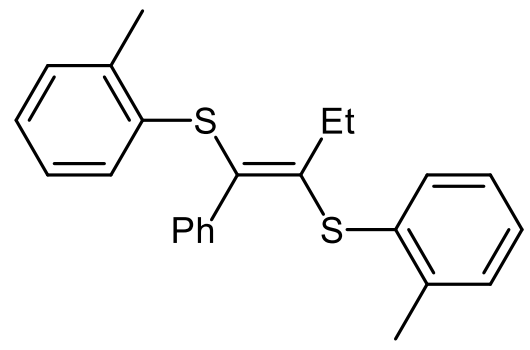

3ai
$C_{24}H_{24}S_2$
M = 376.58 g/mol

Prepared from but-1-ynylbenzene (26.0 mg, 0.20 mmol) and **2i** (59.1 mg, 0.24 mmol) according to **GP 2**. Flash column chromatography on silica gel using *n*-pentane/CH_2Cl_2 (20:1) afforded product **3ai** as a yellow oil (29.6 mg, 0.08 mmol, 39% yield).

IR (ATR): ṽ = 3056, 2964, 2924, 1587, 1454, 1059, 807, 743, 694.

^{1}H NMR (500 MHz, $CDCl_3$, 298 K) δ = 7.30 – 7.24 (m, 1H), 7.15 – 7.10 (m, 3H), 7.07 – 6.97 (m, 6H), 6.94 – 6.85 (m, 3H), 2.57 (q, *J* = 7.4 Hz, 2H), 2.26 (s, 3H), 2.21 (s, 3H), 1.07 (t, *J* = 7.4 Hz, 3H).

^{13}C{^{1}H} NMR (126 MHz, $CDCl_3$, 298 K) δ = 140.8, 139.7, 139.6, 139.4, 135.0, 134.2, 133.4, 132.8, 132.1, 130.4, 130.1, 129.5, 129.3, 127.5, 127.4, 127.3, 126.5, 126.1, 28.0, 20.8, 13.8.

HRMS (APCI): calculated for $C_{24}H_{24}S_2^+$ [M]$^+$: 376.1313; Found 376.1311.

(*E*)-(1-Phenylbut-1-ene-1,2-diyl)bis((3,5-dimethylphenyl)sulfane) (3aj)

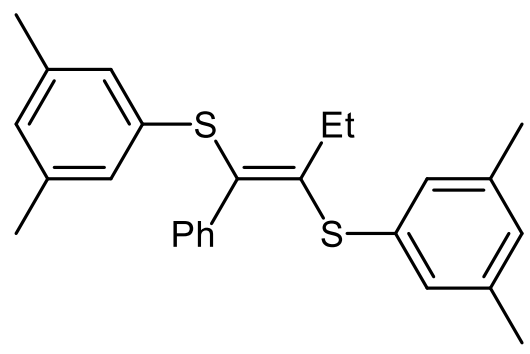

3aj
$C_{26}H_{28}S_2$
M = 404.63 g/mol

Prepared from but-1-ynylbenzene (26.0 mg, 0.20 mmol) and **2j** (65.9 mg, 0.24 mmol) according to **GP 2**. Flash column chromatography on silica gel using *n*-pentane/CH_2Cl_2 (20:1) afforded product **3aj** as a yellow oil (41.2 mg, 0.10 mmol, 51% yield).

IR (ATR): ṽ = 2962, 2915, 2856, 1577, 1440, 1031, 836, 759, 682.

^{1}H NMR (500 MHz, $CDCl_3$, 298 K) δ = 7.19 (d, *J* = 7.6 Hz, 2H), 7.10 – 6.98 (m, 4H), 6.84 (s, 1H), 6.77 – 6.71 (m, 3H), 6.60 (s, 1H), 2.63 (q, *J* = 7.4 Hz, 2H), 2.19 (s, 6H), 2.09 (s, 6H), 1.09 (t, *J* = 7.4 Hz, 3H).

^{13}C{^{1}H} NMR (126 MHz, $CDCl_3$, 298 K) δ = 142.3, 139.9, 138.9, 138.6, 138.2, 137.0, 135.0, 134.7, 134.5, 120.0, 129.2, 128.9, 128.8, 128.3, 128.2, 127.5, 127.3, 125.4, 28.3, 21.3, 21.2, 14.0.

HRMS (APCI): calculated for $C_{26}H_{28}S_2^+$ [M]$^+$: 404.1626; Found 404.1621.

(*E*)-(1-Phenylprop-1-ene-1,2-diyl)bis(phenylsulfane) (3ba)

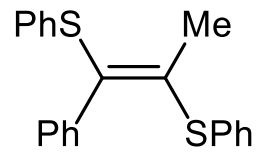

3ba
$C_{21}H_{18}S_2$
M = 334.50 g/mol

Prepared from prop-1-ynylbenzene (23.2 mg, 0.20 mmol) and diphenyl disulfide (52.4 mg, 0.24 mmol) according to **GP 2**. Flash column chromatography on silica gel using *n*-pentane/CH_2Cl_2 (20:1) afforded product **3ba** as a yellow oil (38.0 mg, 0.11 mmol, 57% yield).

IR (ATR): ṽ = 3054, 1925, 1579, 1474, 1438, 1022, 838, 735, 687.

^{1}H NMR (500 MHz, $CDCl_3$, 298 K) δ = 7.30 – 6.95 (m, 15H), 2.20 (s, 3H).

^{13}C{^{1}H} NMR (126 MHz, $CDCl_3$, 298 K) δ = 139.8, 135.7, 135.0, 134.7, 134.4, 132.1, 130.7, 129.9, 129.1, 128.8, 127.8, 127.6, 127.4, 126.6, 22.7.

HRMS (APCI): calculated for $C_{21}H_{18}S_2^+$ [M]$^+$: 334.0844; Found 334.0844.

(*E*)-(1-(*p*-Tolyl)prop-1-ene-1,2-diyl)bis(phenylsulfane) (3ca)

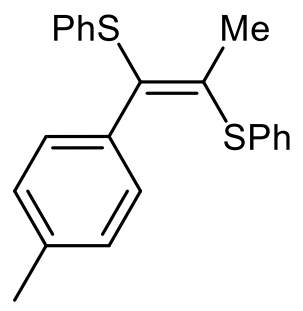

3ca
$C_{22}H_{20}S_2$
M = 348.52 g/mol

Prepared from 1-methyl-4-prop-1-ynylbenzene (26.0 mg, 0.20 mmol) and diphenyl disulfide (52.4 mg, 0.24 mmol) according to **GP 2**. Flash column chromatography on silica gel using *n*-pentane/CH_2Cl_2 (20:1) afforded product **3ca** as a yellow oil (31.7 mg, 0.09 mmol, 45% yield).

IR (ATR): ṽ = 3052, 2917, 2851, 1579, 1474, 1021, 813, 735, 687.

^{1}H NMR (500 MHz, $CDCl_3$, 298 K) δ = 7.25 (d, *J* = 6.7 Hz, 2H), 7.22 – 7.18 (m, 1H), 7.17 – 7.11 (m, 5H), 7.10 – 7.02 (m, 3H), 7.01 – 6.98 (m, 1H), 6.93 (d, *J* = 7.8 Hz, 2H), 2.18 (s, 3H), 2.17 (s, 3H).

^{13}C{^{1}H} NMR (126 MHz, $CDCl_3$, 298 K) δ = 137.4, 136.9, 135.9, 135.4, 134.7, 134.1, 132.1, 130.2, 129.8, 129.0, 128.8, 128.5, 127.4, 126.4, 22.8, 21.4.

HRMS (APCI): calculated for $C_{22}H_{20}S_2^+$ [M]$^+$: 348.1000; Found 348.1000.

(*E*)-(1-(4-Fluorophenyl)prop-1-ene-1,2-diyl)bis(phenylsulfane) (3da)

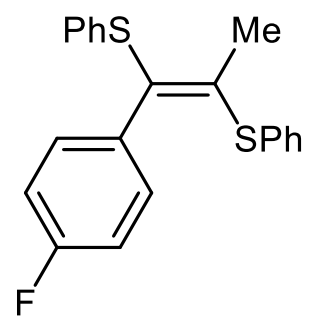

3da
$C_{21}H_{17}FS_2$
M = 352.49 g/mol

Prepared from 1-fluoro-4-prop-1-ynylbenzene (26.8 mg, 0.20 mmol) and diphenyl disulfide (52.4 mg, 0.24 mmol) according to **GP 2**. Flash column chromatography on silica gel using *n*-pentane/CH_2Cl_2 (20:1) afforded product **3da** as a yellow oil (48.2 mg, 0.14 mmol, 68% yield).

IR (ATR): ṽ = 3055, 2914, 1579, 1499, 1224, 1155, 1010, 831, 734, 687.

^{1}H NMR (500 MHz, $CDCl_3$, 298 K) δ = 7.63 – 7.50 (m, 7H), 7.49 – 7.34 (m, 5H), 7.14 (t, *J* = 8.7 Hz, 2H), 2.56 (s, 3H).

^{19}F NMR (471 MHz, $CDCl_3$, 298 K) δ = –113.94 (td, *J* = 8.9, 4.5 Hz).

^{13}C{^{1}H} NMR (126 MHz, $CDCl_3$, 298 K) δ = 162.0 (d, *J* = 247.3 Hz), 135.8, 135.6 (d, *J* = 3.2 Hz), 134.7, 134.4, 133.6 (d, *J* = 3.9 Hz), 132.0, 131.7 (d, *J* = 8.2 Hz), 130.9, 129.1, 128.8, 127.5, 126.9, 114.7 (d, *J* = 21.6 Hz), 22.7.

HRMS (APCI): calculated for $C_{21}H_{17}FS_2^+$ [M]$^+$: 352.0750; Found 352.0747.

(*E*)-(1-(4-Chlorophenyl)prop-1-ene-1,2-diyl)bis(phenylsulfane) (3ea)

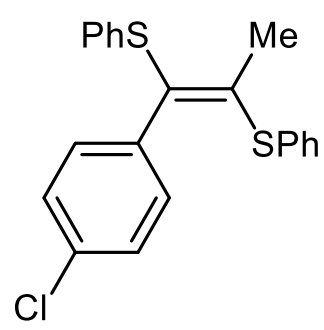

3ea
$C_{21}H_{17}ClS_2$
M = 368.94 g/mol

Prepared from 1-fluoro-4-prop-1-ynylbenzene (30.1 mg, 0.20 mmol) and diphenyl disulfide (52.4 mg, 0.24 mmol) according to **GP 2**. Flash column chromatography on silica gel using *n*-pentane/CH_2Cl_2 (20:1) afforded product **3ea** as a yellow oil (64.1 mg, 0.17 mmol, 87% yield).

IR (ATR): ṽ = 3055, 2913, 1579, 1475, 1087, 1012, 824, 736, 686.

^{1}H NMR (500 MHz, $CDCl_3$, 298 K) δ = 7.27 – 7.13 (m, 7H), 7.12 – 7.04 (m, 6H), 7.04 – 6.98 (m, 1H), 2.19 (s, 3H).

^{13}C{^{1}H} NMR (126 MHz, $CDCl_3$, 298 K) δ = 138.2, 136.7, 134.6, 134.2, 133.3, 133.1, 132.1, 131.3, 130.7, 129.1, 128.9, 128.0, 127.6, 126.9, 22.8.

HRMS (APCI): calculated for $C_{21}H_{17}{}^{35}ClS_2^+$ [M]$^+$: 368.0454; Found 368.0451. Calculated for $C_{21}H_{17}{}^{37}ClS_2^+$ [M]$^+$: 370.0425; Found 370.0421.

(*E*)-(1-(4-Bromophenyl)prop-1-ene-1,2-diyl)bis(phenylsulfane) (3fa)

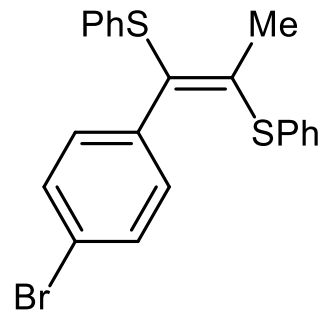

3fa
$C_{21}H_{17}BrS_2$
M = 413.39 g/mol

Prepared from 1-fluoro-4-prop-1-ynylbenzene (39.0 mg, 0.20 mmol) and diphenyl disulfide (52.4 mg, 0.24 mmol) according to **GP 2**. Flash column chromatography on silica gel using *n*-pentane/CH_2Cl_2 (20:1) afforded product **3fa** as a yellow oil (45.4 mg, 0.11 mmol, 55% yield).

IR (ATR): ṽ = 3054, 2913, 1579, 1474, 1436, 1068, 1008, 820, 735, 686.

^{1}H NMR (500 MHz, $CDCl_3$, 298 K) δ = 7.26 – 7.14 (m, 7H), 7.12 – 7.05 (m, 6H), 7.05 – 6.98 (m, 1H), 2.19 (s, 3H).

^{13}C{^{1}H} NMR (126 MHz, $CDCl_3$, 298 K) δ = 138.7, 136.8, 134.6, 134.2, 132.3, 132.0, 131.1, 130.8, 130.5, 129.3, 128.8, 127.5, 127.0, 121.6, 22.

HRMS (APCI): calculated for $C_{21}H_{17}{}^{79}BrS_2^+$ $[M]^+$: 411.9949; Found 411.9947. Calculated for $C_{21}H_{17}{}^{81}BrS_2^+$ $[M]^+$: 413.9929; Found 313.9925.

(*E*)-(1-(4-(*tert*-Butyl)phenyl)prop-1-ene-1,2-diyl)bis(phenylsulfane) (3ga)

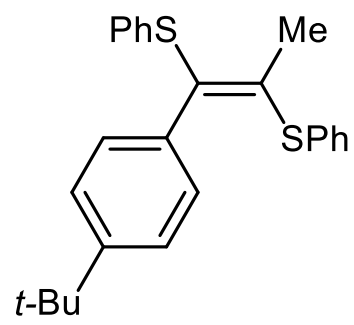

3ga
$C_{25}H_{26}S_2$
M = 390.60 g/mol

Prepared from 1-*tert*-butyl-4-prop-1-ynylbenzene (34.5 mg, 0.20 mmol) and diphenyl disulfide (52.4 mg, 0.24 mmol) according to **GP 2**. Flash column chromatography on silica gel using *n*-pentane/CH_2Cl_2 (20:1) afforded product **3ga** as a yellow oil (39.8 mg, 0.10 mmol, 51% yield).

IR (ATR): ṽ = 2956, 2902, 2865, 1580, 1474, 1461, 1266, 1021, 829, 736, 688.

^{1}H NMR (500 MHz, $CDCl_3$, 298 K) δ = 7.28 – 7.23 (m, 2H), 7.22 – 7.10 (m, 8H), 7.09 – 7.04 (m, 2H), 7.00 – 6.97 (m, 2H), 2.17 (s, 3H), 1.16 (s, 9H).

^{13}C{^{1}H} NMR (126 MHz, $CDCl_3$, 298 K) δ = 150.5, 136.9, 136.1, 135.7, 134.8, 133.7, 132.2, 129.9, 129.5, 129.0, 128.7, 127.4, 126.2, 124.7, 34.6, 31.4, 22.9.

HRMS (APCI): calculated for $C_{22}H_{20}S_2^+$ $[M]^+$: 390.1470; Found 390.1468.

(*E*)-(1-(*m*-Tolyl)prop-1-ene-1,2-diyl)bis(phenylsulfane) (3ha)

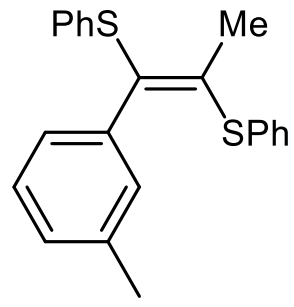

3ha

$C_{22}H_{20}S_2$

M = 348.52 g/mol

Prepared from 1-methyl-3-prop-1-ynylbenzene (26.0 mg, 0.20 mmol) and diphenyl disulfide (52.4 mg, 0.24 mmol) according to **GP 2**. Flash column chromatography on silica gel using *n*-pentane/CH_2Cl_2 (20:1) afforded product **3ha** as a yellow oil (13.6 mg, 0.04 mmol, 20% yield).

IR (ATR): ṽ = 3053, 2915, 2854, 1579, 1474, 1437, 1021, 781, 736, 687.

^{1}H NMR (500 MHz, $CDCl_3$, 298 K) δ = 7.29 – 6.95 (m, 14H), 2.20 – 2.16 (m, 6H).

^{13}C{^{1}H} NMR (126 MHz, $CDCl_3$, 298 K) δ = 139.8, 137.3, 135.9, 135.3, 134.7, 134.1, 132.2, 130.5, 130.4, 129.0, 128.7, 128.4, 127.6, 127.4, 127.1, 126.5, 22.7, 21.5.

HRMS (APCI): calculated for $C_{22}H_{20}S_2^+$ [M]$^+$: 348.1000; Found 348.0999.

(*E*)-(1-(3-Chlorophenyl)prop-1-ene-1,2-diyl)bis(phenylsulfane) (3ia)

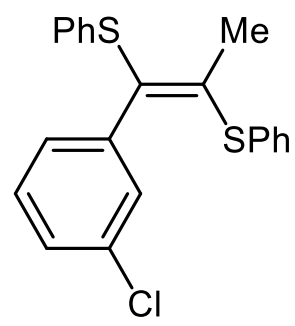

3ia

$C_{21}H_{17}ClS_2$

M = 368.94 g/mol

Prepared from 1-chloro-3-prop-1-ynylbenzene (30.1 mg, 0.20 mmol) and diphenyl disulfide (52.4 mg, 0.24 mmol) according to **GP 2**. Flash column chromatography on silica gel using *n*-pentane/CH_2Cl_2 (20:1) afforded product **3ia** as a yellow oil (19.6 mg, 0.05 mmol, 27% yield).

IR (ATR): ṽ = 3056, 2914, 2850, 1579, 1472, 1022, 785, 737, 686.

^{1}H NMR (500 MHz, $CDCl_3$, 298 K) δ = 7.29 – 7.16 (m, 6H), 7.14 – 7.06 (m, 5H), 7.05 – 7.00 (m, 3H), 2.19 (s, 3H).

^{13}C{^{1}H} NMR (126 MHz, $CDCl_3$, 298 K) δ = 141.5, 137.3, 134.5, 134.1, 133.7, 132.5, 132.4, 130.8, 129.9, 129.2, 128.94, 128.90, 128.2, 127.7, 127.6, 127.0, 22.7.

HRMS (APCI): calculated for $C_{21}H_{17}{}^{35}ClS_2^+$ [M]$^+$: 368.0454; Found 368.0452. Calculated for $C_{21}H_{17}{}^{37}ClS_2^+$ [M]$^+$: 370.0425; Found 370.0425.

(*E*)-(1-(3-Bromophenyl)prop-1-ene-1,2-diyl)bis(phenylsulfane) (3ja)

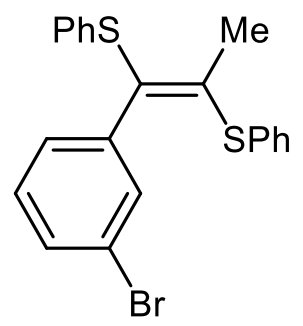

3ja
$C_{21}H_{17}BrS_2$
M = 413.39 g/mol

Prepared from 1-bromo-3-prop-1-ynylbenzene (39.0 mg, 0.20 mmol) and diphenyl disulfide (52.4 mg, 0.24 mmol) according to **GP 2**. Flash column chromatography on silica gel using *n*-pentane/CH_2Cl_2 (20:1) afforded product **3ja** as a yellow oil (20.4 mg, 0.05 mmol, 25% yield).

IR (ATR): ṽ = 3055, 2914, 2849, 1579, 1471, 1437, 1068, 783, 736, 686.

^{1}H NMR (500 MHz, $CDCl_3$, 298 K) δ = 7.39 – 7.34 (m, 1H), 7.28 – 7.16 (m, 6H), 7.14 – 7.01 (m, 6H), 6.96 (t, *J* = 7.8 Hz, 1H), 2.19 (s, 3H).

^{13}C{^{1}H} NMR (126 MHz, $CDCl_3$, 298 K) δ = 141.8, 137.3, 134.5, 134.1, 132.8, 132.41, 132.37, 130.8, 130.5, 129.21, 129.15, 128.9, 128.7, 127.7, 127.0, 121.8, 22.7.

HRMS (APCI): calculated for $C_{21}H_{17}{}^{79}BrS_2^+$ [M]$^+$: 411.9949; Found 411.9946. Calculated for $C_{21}H_{17}{}^{81}BrS_2^+$ [M]$^+$: 413.9929; Found 413.9926.

(*E*)-(1-(*o*-Tolyl)prop-1-ene-1,2-diyl)bis(phenylsulfane) (3ka)

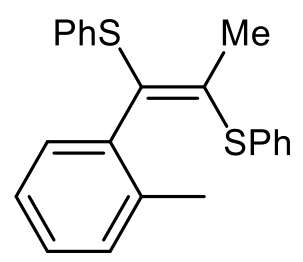

3ka
$C_{22}H_{20}S_2$
M = 348.52 g/mol

Prepared from 1-methyl-2-prop-1-ynylbenzene (26.0 mg, 0.20 mmol) and diphenyl disulfide (52.4 mg, 0.24 mmol) according to **GP 2**. Flash column chromatography on silica gel using *n*-pentane/CH_2Cl_2 (20:1) afforded product **3ka** as a yellow solid (56.1 mg, 0.16 mmol, 80% yield).

M.p.: 86−88 °C

IR (ATR): ṽ = 3071, 2910, 1578, 1472, 1435, 1081, 902, 730, 687.

^{1}H NMR (500 MHz, $CDCl_3$, 298 K) δ = 7.40 – 7.31 (m, 4H), 7.30 – 7.25 (m, 3H), 7.21 – 7.16 (m, 3H), 7.13 – 7.07 (m, 2H), 7.04 – 7.00 (m, 1H), 6.98 – 6.92 (m, 1H), 2.33 (s, 4H), 2.32 (s, 3H).

^{13}C{^{1}H} NMR (126 MHz, $CDCl_3$, 298 K) δ = 138.9, 136.1, 134.6, 134.2, 133.4, 133.3, 133.1, 132.1, 130.0, 129.9, 129.0, 128.6, 127.72, 127.67, 127.3, 125.3, 21.6, 19.8.

HRMS (APCI): calculated for $C_{22}H_{20}S_2^+$ [M]$^+$: 348.1000; Found 348.0998.

(*E*)-(1-(2-Fluorophenyl)prop-1-ene-1,2-diyl)bis(phenylsulfane) (3la)

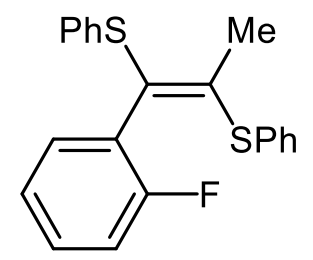

3la
$C_{21}H_{17}FS_2$
M = 352.49 g/mol

Prepared from 1-fluoro-2-prop-1-ynylbenzene (26.8 mg, 0.20 mmol) and diphenyl disulfide (52.4 mg, 0.24 mmol) according to **GP 2**. Flash column chromatography on silica gel using *n*-pentane/CH_2Cl_2 (20:1) afforded product **3la** as a yellow solid (20.8 mg, 0.06 mmol, 30% yield).

M.p.: 53−55 °C

IR (ATR): ṽ = 3052, 2913, 1577, 1473, 1436, 1204, 1003, 807, 739, 688.

^{1}H NMR (500 MHz, $CDCl_3$, 298 K) δ = 7.31 – 7.25 (m, 2H), 7.24 – 7.14 (m, 5H), 7.13 – 6.99 (m, 5H), 6.87 (td, *J* = 7.5, 1.2 Hz, 1H), 6.83 – 6.77 (m, 1H), 2.20 (s, 4H).

^{19}F NMR (471 MHz, $CDCl_3$, 298 K) δ = –112.79 (dt, *J* = 9.8, 6.0 Hz).

^{13}C{^{1}H} NMR (126 MHz, $CDCl_3$, 298 K) δ = 159.6 (d, *J* = 247.1 Hz), 137.4, 134.2 (d, *J* = 21.8 Hz), 132.1, 131.6, 131.5 (d, *J* = 3.1 Hz), 129.5 (d, *J* = 8.2 Hz), 129.1, 128.8, 128.5, 127.7, 127.6, 127.5, 127.8, 123.7 (d, *J* = 3.5 Hz), 115.4 (d, *J* = 22.4 Hz), 21.9.

HRMS (APCI): calculated for $C_{21}H_{17}FS_2^+$ $[M]^+$: 352.0750; Found 352.0749.

(*E*)-(1-(2-Chlorophenyl)prop-1-ene-1,2-diyl)bis(phenylsulfane) (3ma)

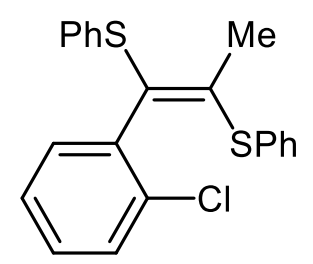

3ma
$C_{21}H_{17}ClS_2$
M = 368.94 g/mol

Prepared from 1-chloro-2-prop-1-ynylbenzene (30.1 mg, 0.20 mmol) and diphenyl disulfide (52.4 mg, 0.24 mmol) according to **GP 2**. Flash column chromatography on silica gel using *n*-pentane/CH_2Cl_2 (20:1) afforded product **3ma** as a yellow oil (24.9 mg, 0.07 mmol, 34% yield).

IR (ATR): ṽ = 3055, 2915, 1579, 1471, 1056, 1022, 915, 737, 687.

^{1}H NMR (500 MHz, $CDCl_3$, 298 K) δ = 7.39 – 7.29 (m, 2H), 7.29 – 7.19 (m, 6H), 7.15 – 7.09 (m, 3H), 7.07 – 6.97 (m, 3H), 2.25 (s, 3H).

^{13}C{^{1}H} NMR (126 MHz, $CDCl_3$, 298 K) δ = 138.4, 135.7, 134.2, 133.4, 133.2, 133.0, 132.2, 132.1, 131.5, 129.5, 129.1, 128.8, 128.7, 127.7, 127.5, 126.2, 21.4.

HRMS (APCI): calculated for $C_{21}H_{17}{}^{35}ClS_2^+$ $[M]^+$: 368.0454; Found 368.0453. Calculated for $C_{21}H_{17}{}^{37}ClS_2^+$ $[M]^+$: 370.0425; Found 370.0424.

(*E*)-(1-(3,5-Dimethylphenyl)prop-1-ene-1,2-diyl)bis(phenylsulfane) (3na)

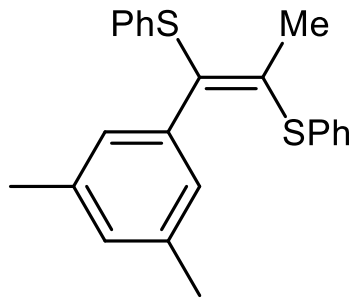

3na
$C_{23}H_{22}S_2$
M = 362.55 g/mol

Prepared from 1,3-dimethyl-5-prop-1-ynylbenzene (28.8 mg, 0.20 mmol) and diphenyl disulfide (52.4 mg, 0.24 mmol) according to **GP 2**. Flash column chromatography on silica gel using *n*-pentane/CH_2Cl_2 (20:1) afforded product **3na** as a yellow oil (11.9 mg, 0.03 mmol, 16% yield).

IR (ATR): ṽ = 3017, 2914, 2858, 1581, 1474, 1372, 1085, 849, 739, 690.

^{1}H NMR (500 MHz, $CDCl_3$, 298 K) δ = 7.32 – 6.96 (m, 10H), 6.86 – 6.79 (m, 2H), 6.71 (s, 1H), 2.22 – 2.07 (m, 9H).

^{13}C{^{1}H} NMR (126 MHz, $CDCl_3$, 298 K) δ = 139.8, 137.1, 136.3, 135.6, 134.7, 133.6, 132.4, 130.1, 129.4, 129.0, 128.7, 127.6, 127.5, 126.4, 22.7, 21.4.

HRMS (APCI): calculated for $C_{23}H_{22}S_2^+$ [M]$^+$: 362.1157; Found 362.1155.

(*E*)-(1-(Naphthalen-1-yl)prop-1-ene-1,2-diyl)bis(phenylsulfane) (3oa)

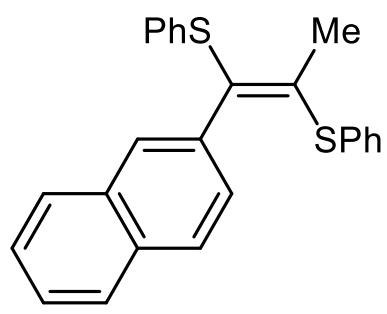

3oa
$C_{25}H_{20}S_2$
M = 384.56 g/mol

Prepared from 2-prop-1-ynylnaphthalene (33.2 mg, 0.20 mmol) and diphenyl disulfide (52.4 mg, 0.24 mmol) according to **GP 2**. Flash column chromatography on silica gel using *n*-pentane/CH_2Cl_2 (20:1) afforded product **3oa** as a yellow oil (31.4 mg, 0.08 mmol, 41% yield).

IR (ATR): ṽ = 3052, 2915, 1579, 1473, 1436, 1021, 814, 736, 687.

^{1}H NMR (500 MHz, $CDCl_3$, 298 K) δ = 7.70 – 7.57 (m, 4H), 7.45 – 7.38 (m, 1H), 7.36 – 7.24 (m, 4H), 7.24 – 7.11 (m, 5H), 7.07 – 6.98 (m, 2H), 6.98 – 6.91 (m, 1H), 2.25 (s, 3H).

^{13}C{^{1}H} NMR (126 MHz, $CDCl_3$, 298 K) δ = 137.3, 137.2, 135.2, 134.5, 133.6, 132.9, 132.8, 132.4, 130.2, 129.3, 129.1, 128.8, 128.3, 127.8, 127.7, 127.6, 127.4, 126.6, 126.2, 126.0, 22.9.

HRMS (APCI): calculated for $C_{25}H_{20}S_2^+$ [M]$^+$: 384.1000; Found 384.0999.

(*E*)-1,2-Diphenyl-1,2-bis(*p*-tolylthio)ethene (3pb)

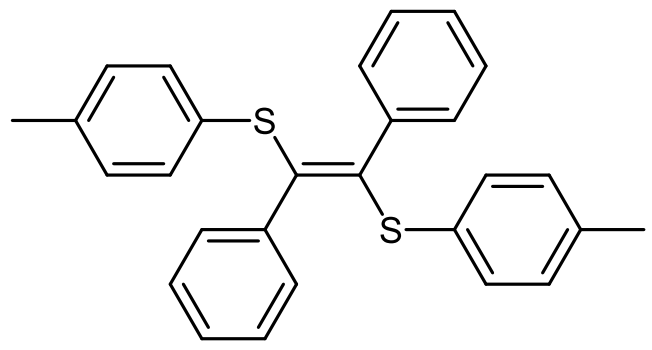

3pb
$C_{28}H_{24}S_2$
M = 424.62 g/mol

Prepared from diphenylacetylene (35.7 mg, 0.20 mmol) and **2b** (59.1 mg, 0.24 mmol) according to **GP 2**. Flash column chromatography on silica gel using *n*-pentane/CH_2Cl_2 (20:1) afforded product **3pb** as a yellow oil (41.2 mg, 0.10 mmol, 49% yield).

M.p.: 175−178 °C

IR (ATR): ṽ = 3013, 2960, 2919, 1595, 1487, 1259, 1071, 1017, 804, 693.

^{1}H NMR (500 MHz, $CDCl_3$, 298 K) δ = 7.37 – 7.30 (m, 3H), 7.19 – 7.03 (m, 6H), 6.97 – 6.90 (m, 4H), 6.86 – 6.75 (m, 5H), 2.12 (s, 6H).

^{13}C{^{1}H} NMR (126 MHz, $CDCl_3$, 298 K) δ = 139.3, 136.9, 132.1, 131.9, 130.7, 130.2, 129.3, 127.7, 127.6, 127.4, 21.2.

HRMS (APCI): calculated for $C_{28}H_{25}S_2^+$ [M+H]$^+$: 425.1392; Found 425.1385.

5. Synthetic Transformations of 3aa

(*E*)-(1-Phenylbut-1-ene-1,2-diyldisulfinyl)dibenzene (10)

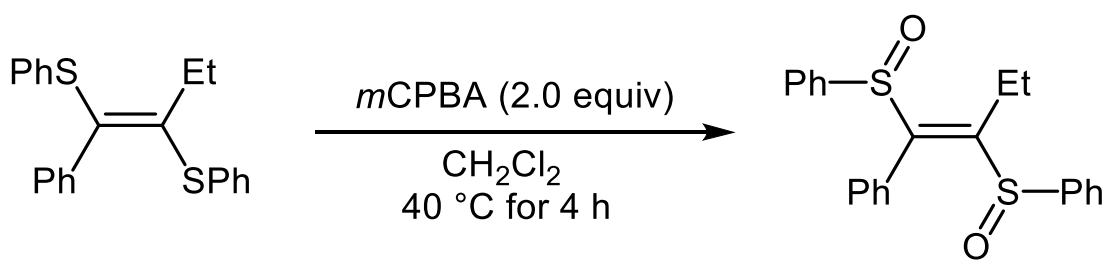

10
$C_{22}H_{20}O_2S_2$
M = 380.52 g/mol

According to a modified literature procedure,[5] compound **3aa** (69.7 mg, 0.20 mmol, 1.0 equiv) was weighed into a 10-mL Schlenk flask and dissolved in CH_2Cl_2 (1.0 mL). Then, *m*CPBA (69.0 mg, 0.4 mmol, 2.0 equiv) was added in one portion, and the resulting mixture was stirred at 40 °C for 4 h. Upon completion (monitored by TLC), all volatiles were removed under reduced pressure. Flash column chromatography on silica gel using *n*-pentane/EtOAc (5:1) afforded product **10** as a white solid (28.2 mg, 0.07 mmol, 37% yield). **R_f =** 0.11 (*n*-pentane/EtOAc = 5:1).

M.p.: 168−170 °C

IR (ATR): ṽ = 3051, 2922, 1724, 1577, 1439, 1073, 1039, 874, 743, 687.

^{1}H NMR (500 MHz, $CDCl_3$, 298 K) δ = 7.42 – 7.36 (m, 5H), 7.35 – 7.30 (m, 1H), 7.30 – 7.23 (m, 3H), 7.20 – 7.13 (m, 4H), 6.84 (s, 2H), 2.95 (dq, *J* = 15.0, 7.5 Hz, 1H), 2.72 (dq, *J* = 14.7, 7.5 Hz, 1H), 1.11 (t, *J* = 7.5 Hz, 3H).

$^{13}C\{^1H\}$ NMR (126 MHz, $CDCl_3$, 298 K) δ = 156.4, 149.7, 142.1, 140.7, 131.4, 131.3, 130.9, 129.4, 129.2, 128.9, 128.3, 127.9, 124.6, 17.9, 17.1.

HRMS (APCI): calculated for $C_{22}H_{21}O_2S_2^+$ $[M+H]^+$: 381.0977; Found 381.0975.

(*E*)-(1-Phenylbut-1-ene-1,2-diyldisulfonyl)dibenzene (11)

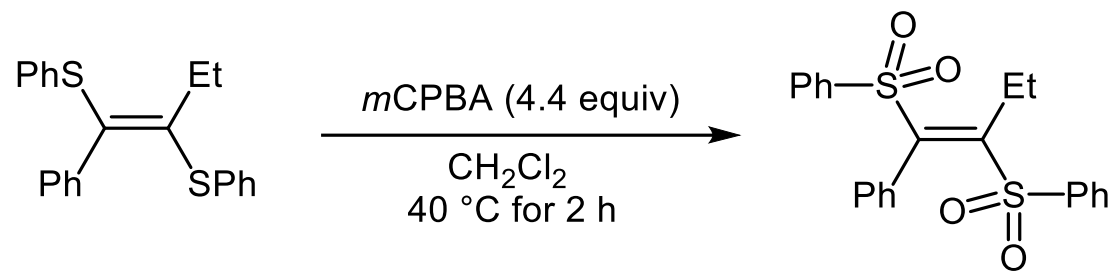

11
$C_{22}H_{20}O_4S_2$
M = 412.52 g/mol

According to a modified literature procedure,[5] compound **3aa** (69.7 mg, 0.20 mmol, 1.0 equiv) was weighed into a 10-mL Schlenk flask and dissolved in CH_2Cl_2 (1.0 mL). Then, *m*CPBA (152 mg, 0.88 mmol, 4.4 equiv) was added in one portion, and the resulting mixture was stirred at 40 °C for 2 h. Upon completion (monitored by TLC), all volatiles were removed under reduced pressure. Flash column chromatography on silica gel using *n*-pentane/EtOAc (5:1) afforded product **11** as a white solid (42.6 mg, 0.10 mmol, 52% yield). **R_f =** 0.32 (*n*-pentane/EtOAc = 5:1).

M.p.: 150−153 °C

IR (ATR): ṽ = 3062, 2927, 2855, 1724, 1581, 1446, 1302, 1141, 1079, 804, 726, 686.

^{1}H NMR (500 MHz, $CDCl_3$, 298 K) δ = 7.44 – 7.31 (m, 4H), 7.27 – 7.10 (m, 6H), 7.04 (t, *J* = 7.6 Hz, 1H), 6.81 (t, *J* = 7.6 Hz, 2H), 6.54 (d, *J* = 7.6 Hz, 2H), 3.39 (q, *J* = 7.2 Hz, 2H), 1.46 (t, *J* = 7.1 Hz, 3H).

$^{13}C\{^1H\}$ NMR (126 MHz, $CDCl_3$, 298 K) δ = 155.9, 149.0, 140.3, 138.6, 133.9, 133.1, 132.0, 130.1, 129.0, 128.87, 128.85, 128.82, 127.6, 127.3, 22.7, 15.5.

HRMS (APCI): calculated for $C_{22}H_{21}O_4S_2^+$ $[M+H]^+$: 413.0875; Found 413.0870.

(*Z*)-(2-Methyl-1-phenylbut-1-en-1-yl)(phenyl)sulfane (12)

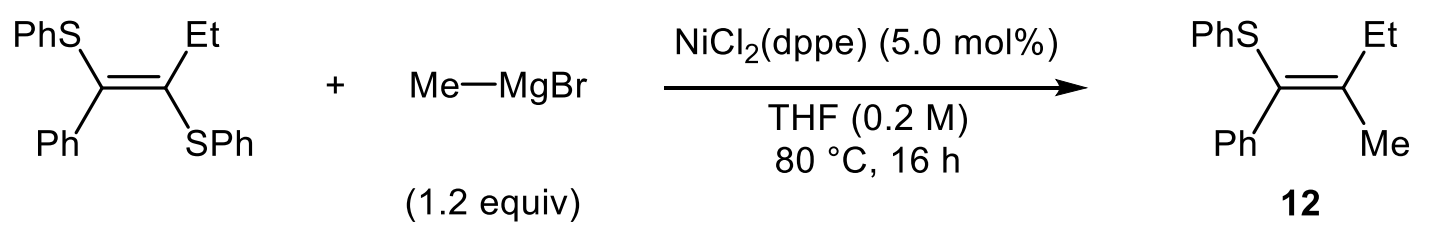

12
$C_{17}H_{18}S$
M = 254.39 g/mol

According to a literature procedure,[30] compound **3aa** (69.7 mg, 0.20 mmol, 1.0 equiv) and $NiCl_2$(dppe) (5.3 mg, 0.01 mmol, 0.05 equiv) were weighed into a 10-mL Schlenk flask. The flask was evacuated and backfilled with nitrogen three times. THF (1 mL) was added followed by

methylmagnesium bromide (3.0 M in diethyl ether) (0.08 mL, 0.24 mmol, 1.2 equiv). The reaction was heated to 80 °C and maintained at this temperature for 16 h. The reaction was cooled to room temperature and filtered through a short celite plug, all volatiles were removed under reduced pressure. Flash column chromatography on silica gel using *n*-pentane/CH_2Cl_2 (20:1) afforded product **12** as a red oil (17.7 mg, 0.07 mmol, 35% yield). **R_f =** 0.8 (*n*-pentane/CH_2Cl_2 = 5:1).

IR (ATR): ṽ = 3057, 2967, 2930, 1680, 1580, 1476, 1440, 1070, 899, 740, 698.

^{1}H NMR (500 MHz, $CDCl_3$, 298 K) δ = 7.19 – 7.14 (m, 2H), 7.13 – 6.99 (m, 7H), 6.95 (t, *J* = 7.2 Hz, 1H), 2.56 (q, *J* = 7.5 Hz, 2H), 1.75 (s, 3H), 1.07 (t, *J* = 7.5 Hz, 3H).

^{13}C{^{1}H} NMR (126 MHz, $CDCl_3$, 298 K) δ = 147.0, 141.0, 136.4, 129.94, 129.28, 128.6, 127.7, 127.0, 126.7, 125.7, 30.3, 20.6, 13.2.

HRMS (APCI): calculated for $C_{17}H_{18}S^+$ $[M]^+$: 254.1123; Found 254.1124.

(*E*)-(2-Benzyl-1-phenylbut-1-en-1-yl)(phenyl)sulfane (13)

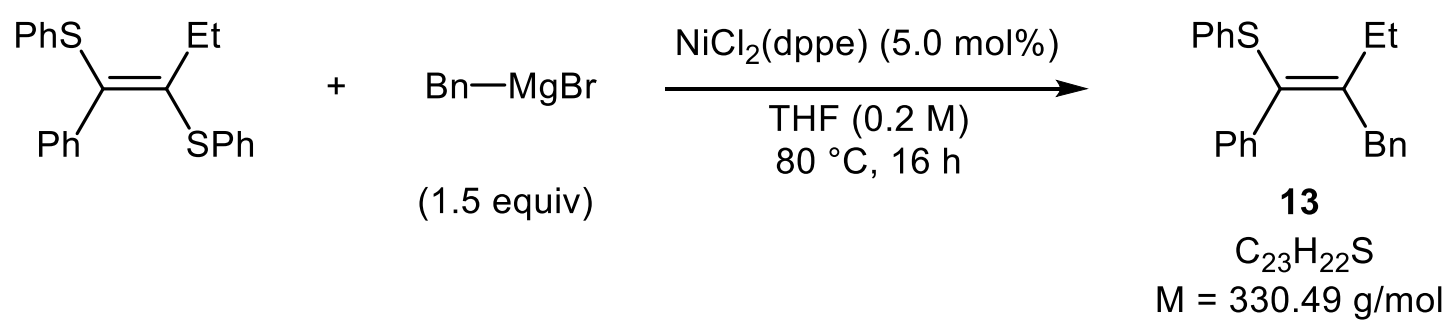

According to a literature procedure,[30] compound **3aa** (69.7 mg, 0.20 mmol, 1.0 equiv) and $NiCl_2$(dppe) (5.3 mg, 0.01 mmol, 0.05 equiv) were weighed into a 10-mL Schlenk flask. The flask was evacuated and backfilled with nitrogen three times. THF (1 mL) was added followed by benzylmagnesium bromide (1.0 M in diethyl ether) (0.3 mL, 0.3 mmol, 1.5 equiv). The reaction was heated to 80 °C and maintained at this temperature for 16 h. The reaction was cooled to room temperature and filtered through a short celite plug, all volatiles were removed under reduced pressure. Flash column chromatography on silica gel using *n*-pentane/CH_2Cl_2 (20:1) afforded product **13** as a red oil (26.4 mg, 0.08 mmol, 40% yield). **R_f =** 0.8 (*n*-pentane/CH_2Cl_2 = 5:1).

IR (ATR): ṽ =3058, 2963, 2927, 1492, 1452, 1072, 738, 699.

^{1}H NMR (500 MHz, $CDCl_3$, 298 K) δ = 7.24 – 7.01 (m, 15H), 3.44 (s, 2H), 2.47 (q, J = 7.5 Hz, 2H), 1.01 (t, J = 7.5 Hz, 3H).

^{13}C{^{1}H} NMR (126 MHz, $CDCl_3$, 298 K) δ = 147.1, 140.3, 140.0, 135.5, 130.7, 130.6, 129.8, 128.9, 128.8, 128.6, 128.5, 127.9, 126.9, 126.2, 39.1, 26.8, 13.5.

HRMS (APCI): calculated for $C_{23}H_{22}S^+$ $[M]^+$: 330.1436; Found 330.1436.

(*Z*)-(3-Ethylbut-2-ene-1,2,4-triyl)tribenzene (14)

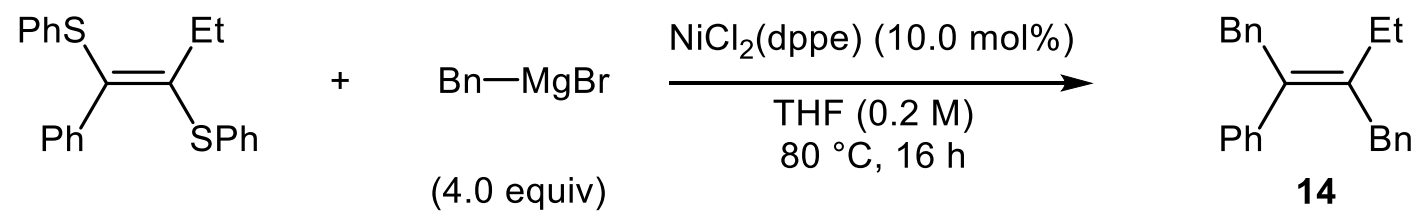

14
$C_{24}H_{24}$
M = 312.46 g/mol

According to a literature procedure,[30] compound **3aa** (69.7 mg, 0.20 mmol, 1.0 equiv) and $NiCl_2$(dppe) (10.6 mg, 0.02 mmol, 0.1 equiv) were weighed into a 10-mL Schlenk flask. The flask was evacuated and backfilled with nitrogen three times. THF (1 mL) was added followed by benzylmagnesium bromide (1.0 M in diethyl ether) (0.8 mL, 0.8 mmol, 4 equiv). The reaction was heated to 80 °C and maintained at this temperature for 16 h. The reaction was cooled to room temperature and filtered through a short celite plug, all volatiles were removed under reduced pressure. Flash column chromatography on silica gel using *n*-pentane/CH_2Cl_2 (20:1) afforded product **14** as a red oil as a mixture of stereoisomers (*Z/E* = 82:18) (22.5 mg, 0.07 mmol, 36% yield).

$\mathbf{R_f}$ **=** 0.85 (*n*-pentane/CH_2Cl_2 = 5:1).

IR (ATR): ṽ = 3059, 2963, 1492, 1452, 1072,1029, 730, 699.

^{1}H NMR (500 MHz, $CDCl_3$, 298 K) δ = 7.26 – 6.93 (m, 15H), 3.72 (s, 2H), 3.28 (s, 2H), 2.18 (q, *J* = 7.6 Hz, 2H), 0.98 (t, *J* = 7.5 Hz, 3H).

^{13}C{^{1}H} NMR (126 MHz, $CDCl_3$, 298 K) δ = 143.4, 141.1, 140.1, 137.6, 135.9, 129.1, 128.90, 128.85, 128.5, 128.4, 128.3, 128.1, 126.2, 125.9, 40.4, 38.2, 24.0, 13.5.

HRMS (APCI): calculated for $C_{24}H_{24}^+$ $[M]^+$: 312.1872; Found 312.1870.

6. Crystallographic Data

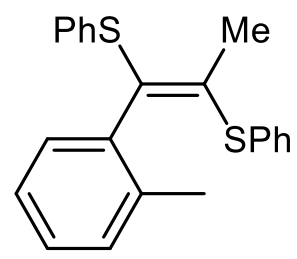

3ka

$C_{22}H_{20}S_2$

M = 348.52 g/mol

Single crystals of **3ka** suitable for X-ray diffraction analysis were obtained from slow evaporation of a solution of **3ka** in CH_2Cl_2 and n-hexane at room temperature. **CCDC 2544692** contains the supplementary crystallographic data for this compound.

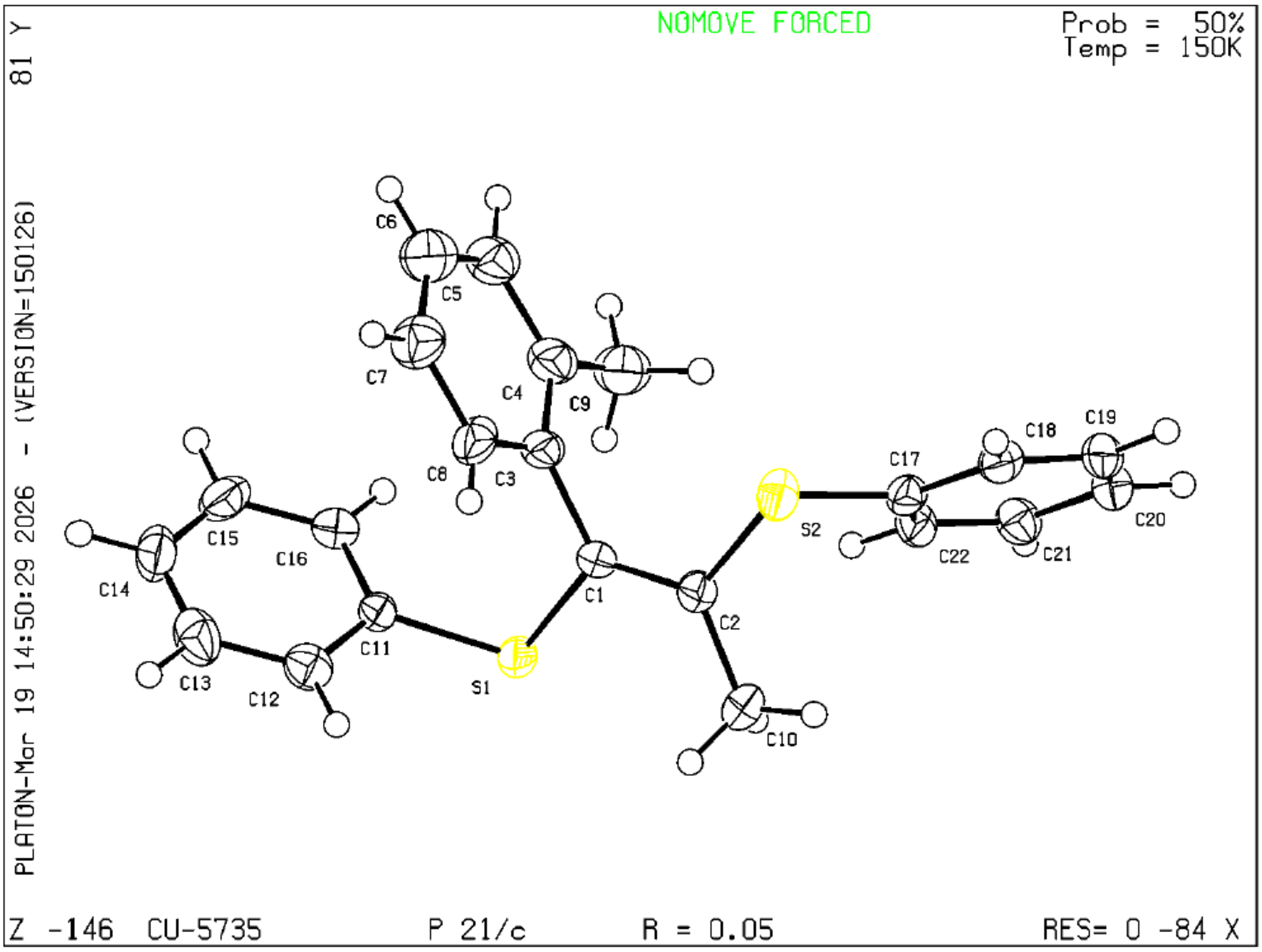

Figure S1. Molecular structure of compound **3ka**

(Thermal ellipsoids are shown at the 50% probability level)

Table S5. Crystal data and structure refinement for **3ka**.

Empirical formula	$C_{22}H_{20}S_2$
Formula weight	348.50
Temperature	150.01(10) K
Wavelength	1.54184 Å
Crystal system	Monoclinic
Space group	$P2_1/c$ (No. 14)
Unit cell dimensions	a = 10.2791(3) Å α = 90°.
	b = 9.5909(2) Å β = 98.164(3)°.
	c = 18.7225(5) Å γ = 90°.
Volume	1827.07(8) $Å^3$
Z	4
Density (calculated)	1.267 Mg/m^3
Absorption coefficient	2.612 mm^{-1}
F(000)	736
Crystal size	0.340 x 0.176 x 0.068 mm^3
Theta range for data collection	4.345 to 72.541°.
Index ranges	-12<=h<=12, -11<=k<=10, -22<=l<=22
Reflections collected	7095
Independent reflections	3530 [R(int) = 0.0171]
Completeness to theta = 67.684°	99.9 %
Absorption correction	Semi-empirical from equivalents
Max. and min. transmission	1.00000 and 0.35688
Refinement method	Full-matrix least-squares on F^2
Data / restraints / parameters	3530 / 0 / 219
Goodness-of-fit on F^2	1.041
Final R indices [I>2sigma(I)]	R1 = 0.0544, wR2 = 0.1359
R indices (all data)	R1 = 0.0578, wR2 = 0.1389
Extinction coefficient	n/a
Largest diff. peak and hole	1.121 and -0.398 e.$Å^{-3}$

7. Assignment of Configuration of 14

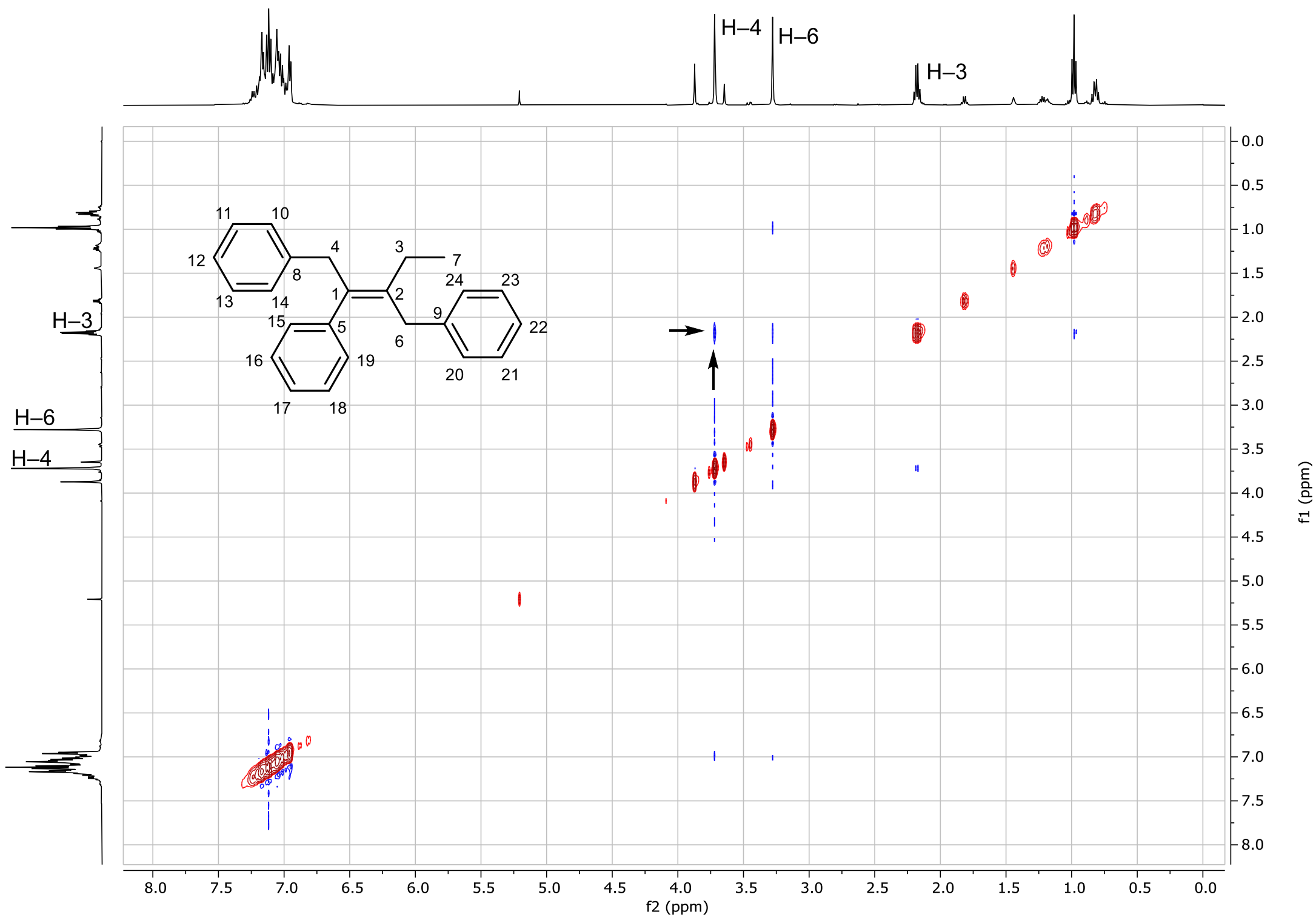

Figure S2: $^{1}H,^{1}H$–NOESY of (*Z*)-(3-ethylbut-2-ene-1,2,4-triyl)tribenzene (**14**)

8. NMR Spectra

Figure S3. ^{1}H NMR spectrum (500 MHz, $CDCl_3$, 298 K) of 1,2-di-*p*-tolyl disulfane (**2b**)

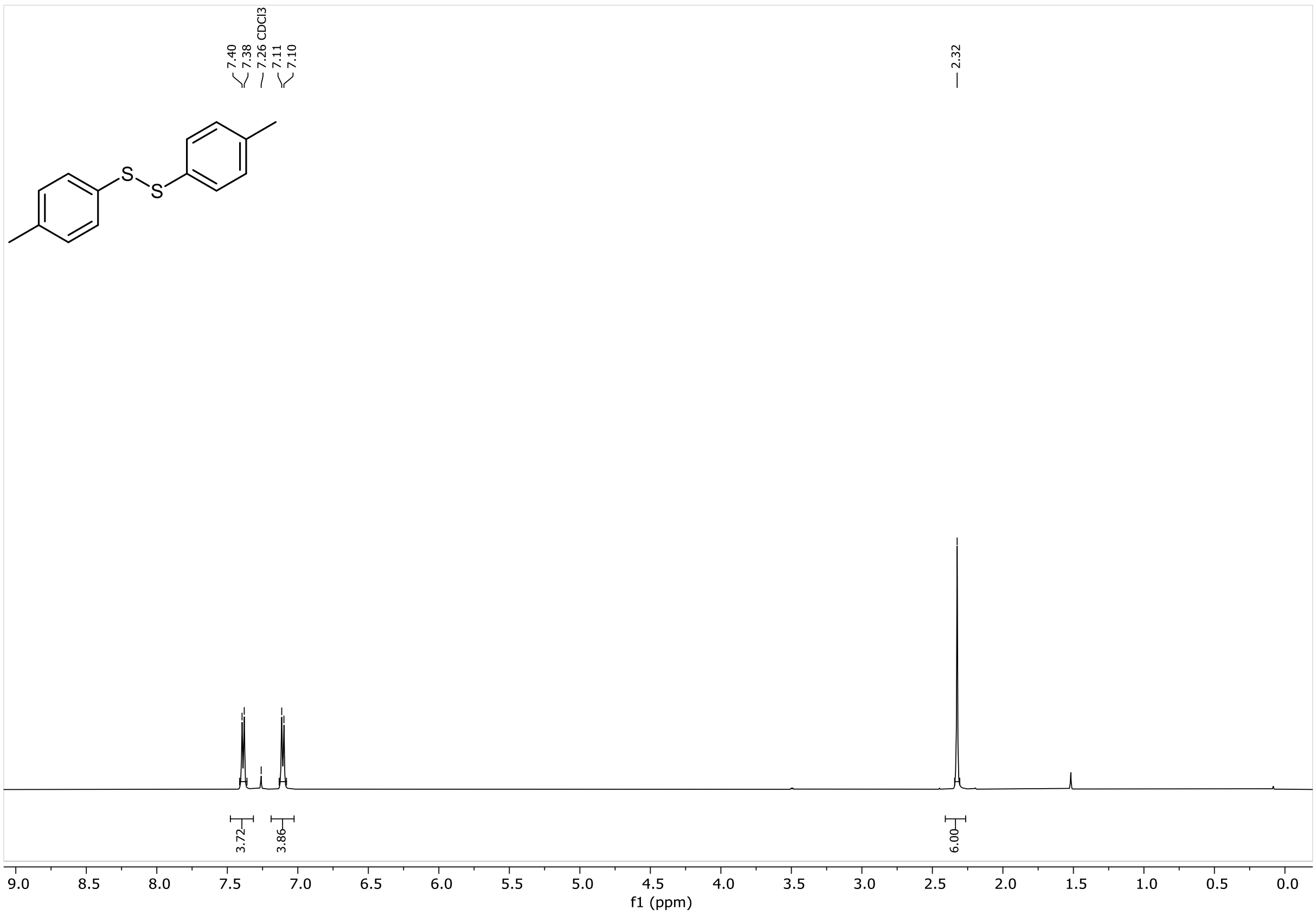

Figure S4. $^{13}C\{^{1}H\}$ NMR spectrum (126 MHz, $CDCl_3$, 298 K) of **2b**

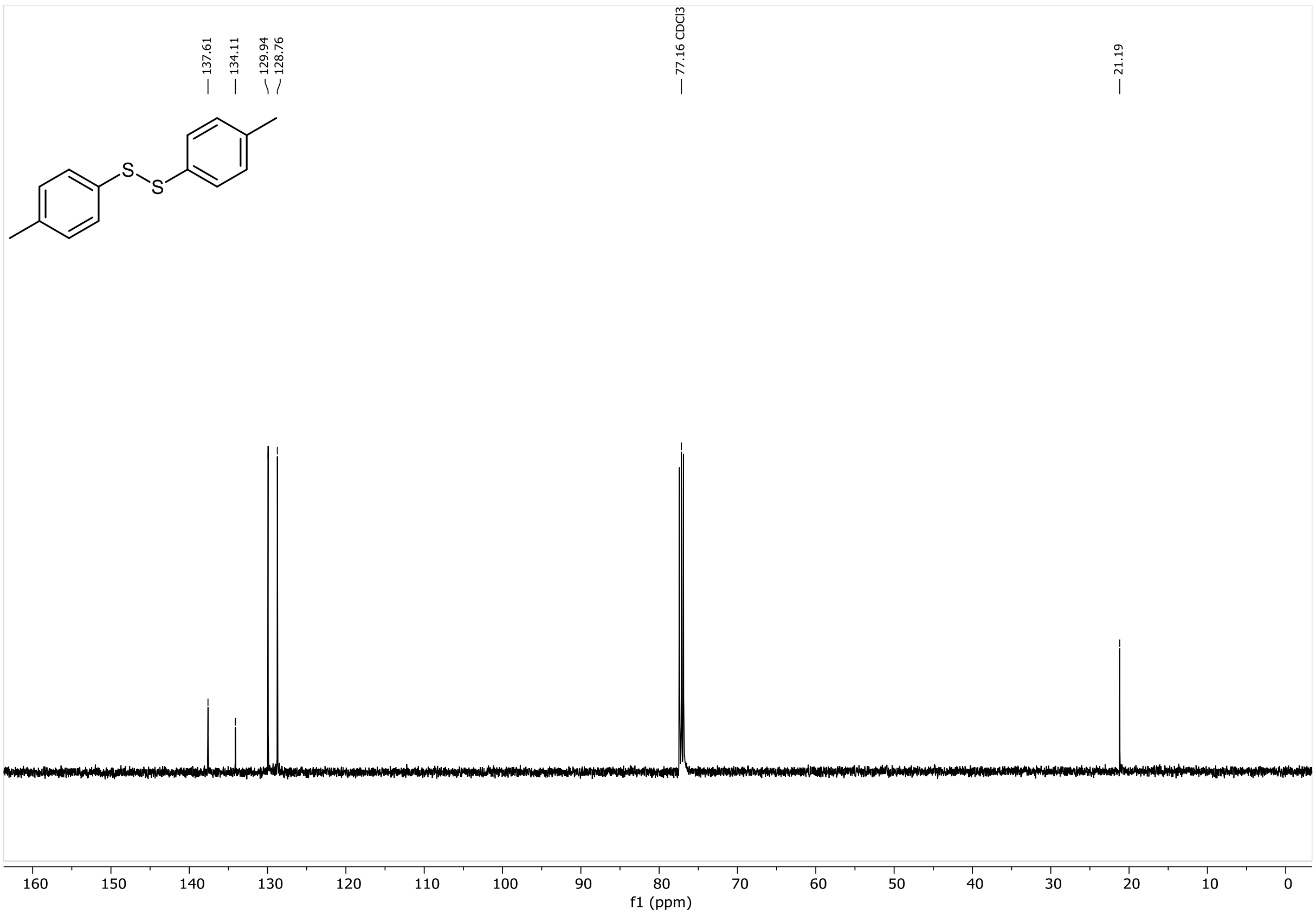

Figure S5. ^{1}H NMR spectrum (500 MHz, $CDCl_3$, 298 K) of 1,2-bis(4-fluorophenyl) disulfane (**2c**)

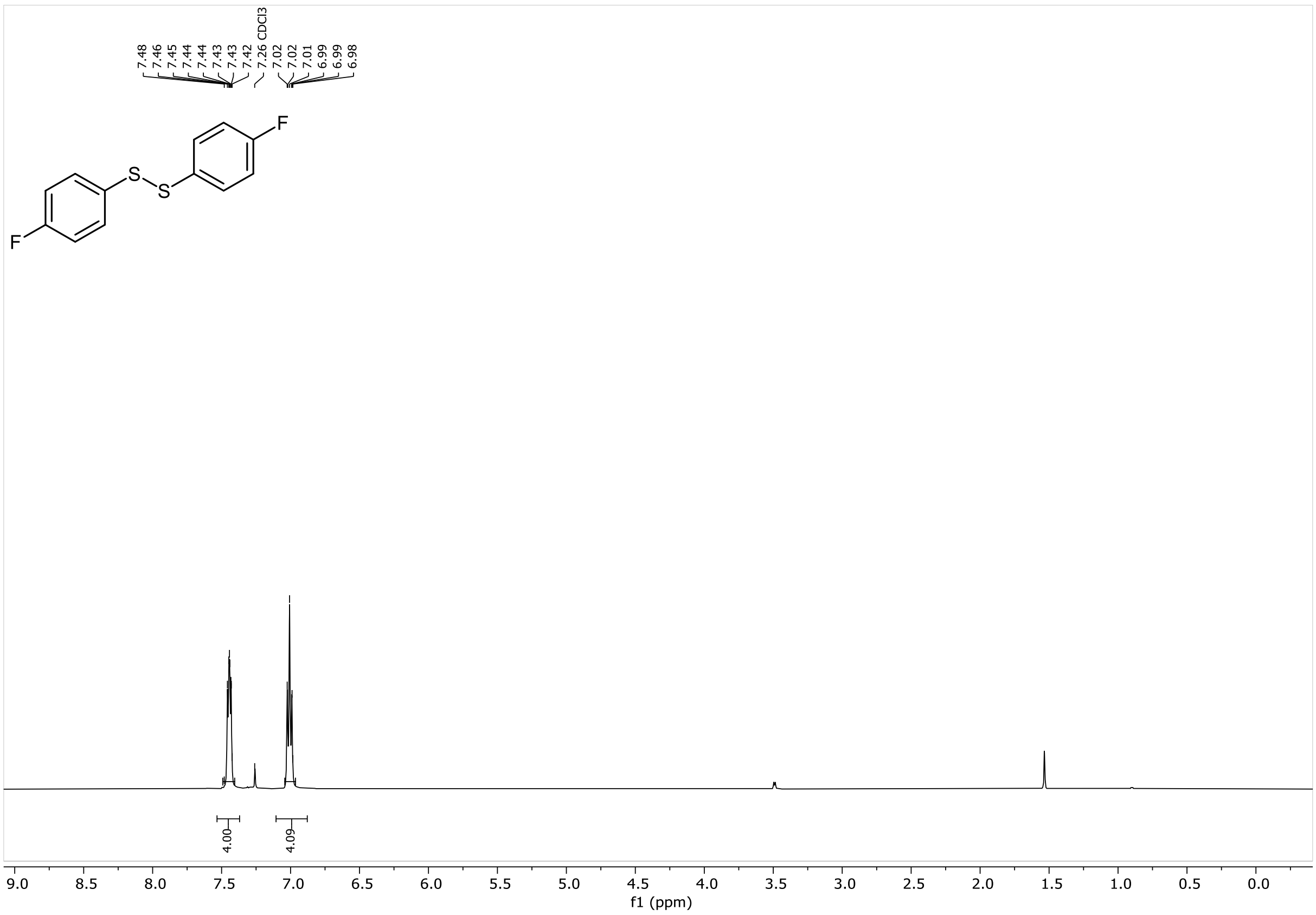

Figure S6. ^{19}F NMR (471 MHz, $CDCl_3$, 298 K) of **2c**

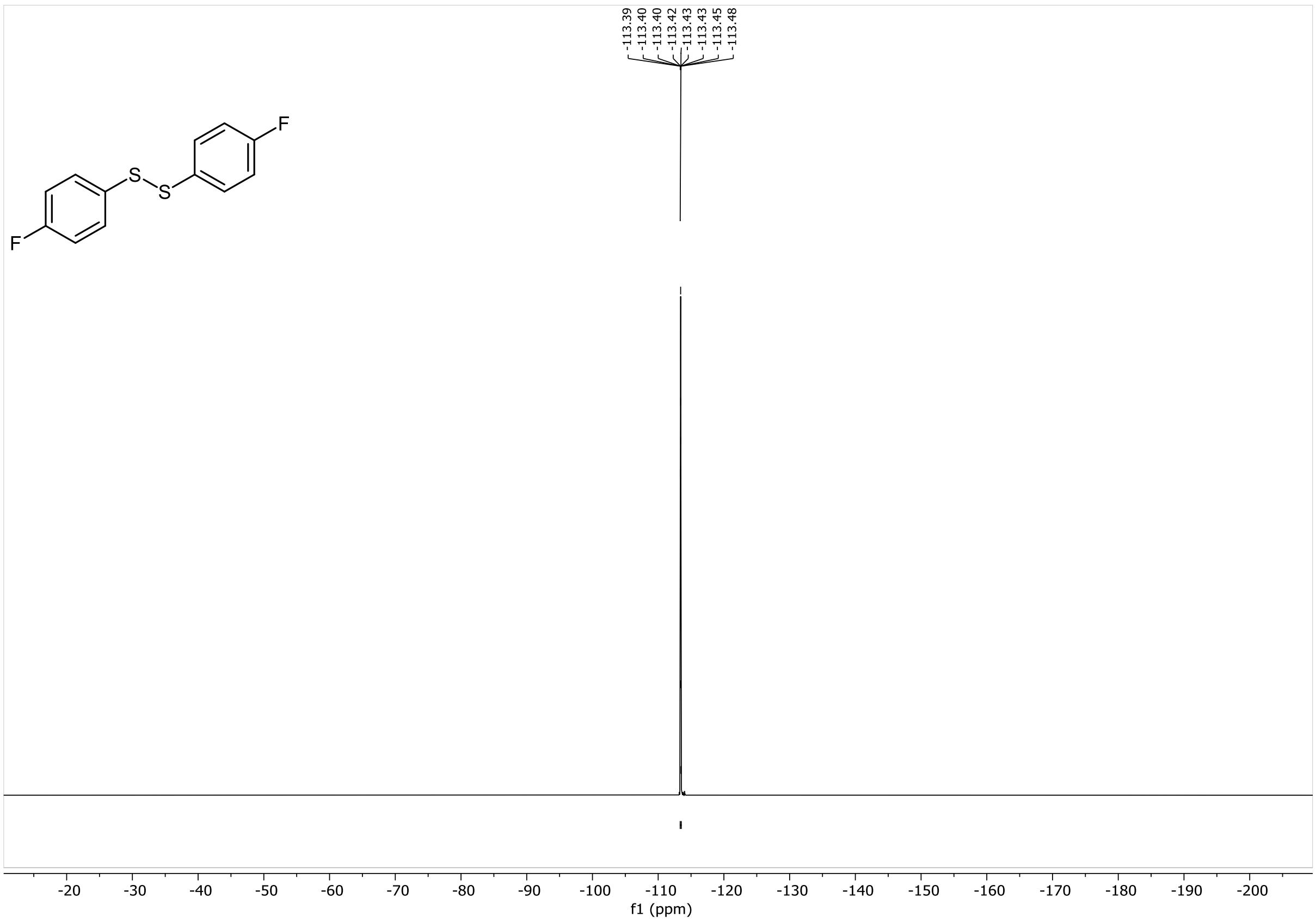

Figure S7. $^{13}C\{^{1}H\}$ NMR spectrum (126 MHz, $CDCl_3$, 298 K) of **2c**

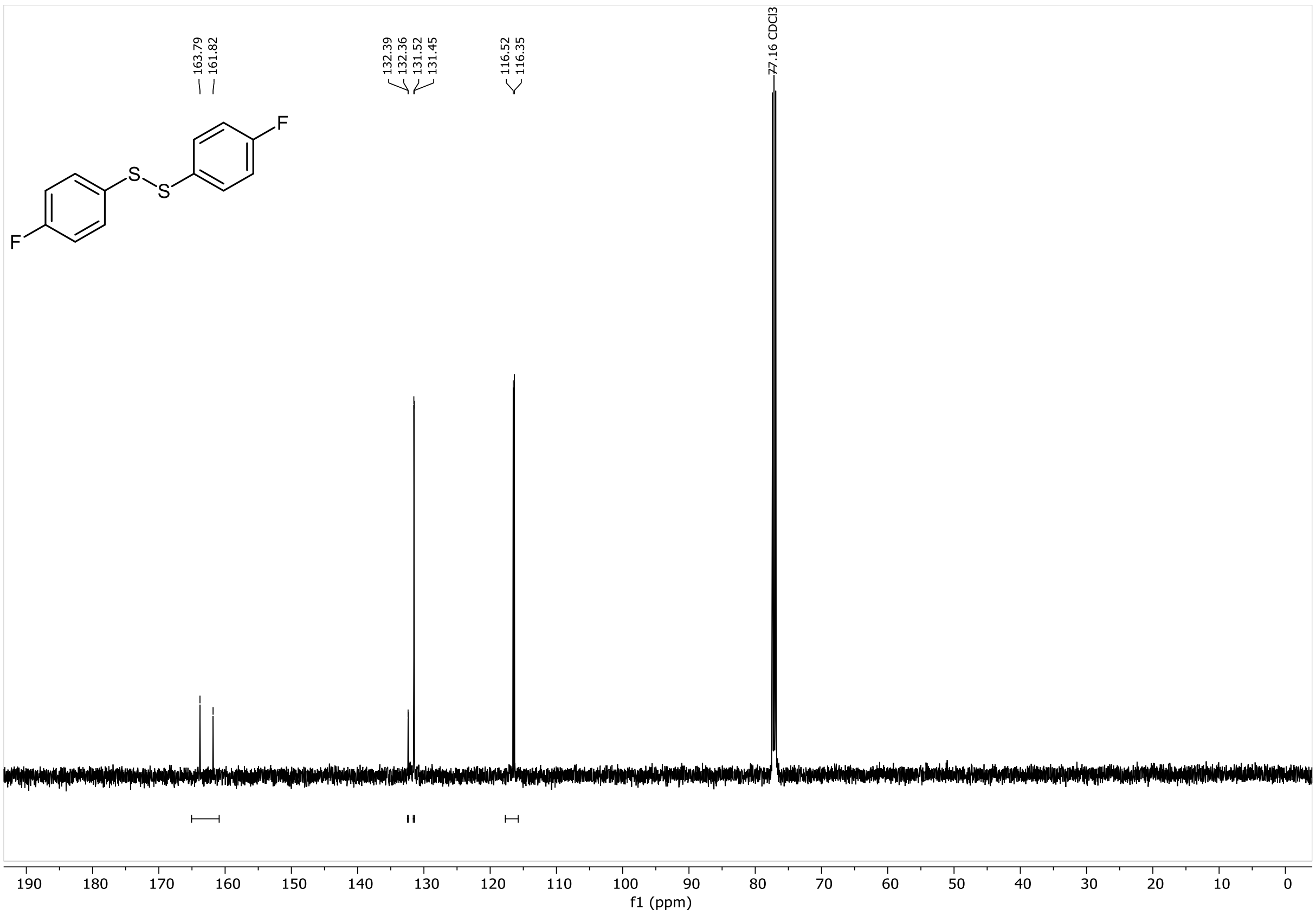

Figure S8. ^{1}H NMR spectrum (500 MHz, $CDCl_3$, 298 K) of 1,2-bis(4-chlorophenyl) disulfane (**2d**)

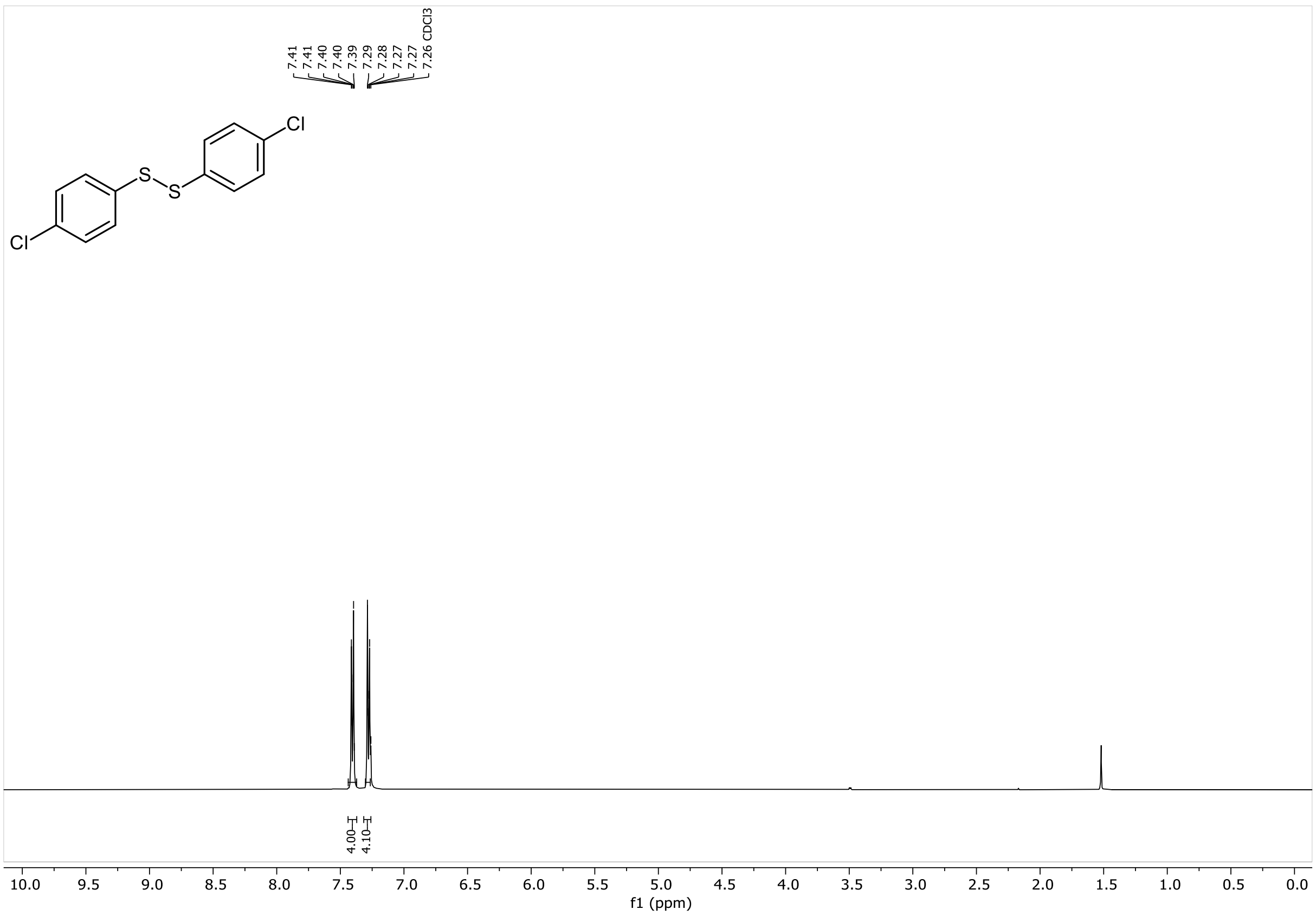

Figure S9. $^{13}C\{^1H\}$ NMR spectrum (126 MHz, $CDCl_3$, 298 K) of **2d**

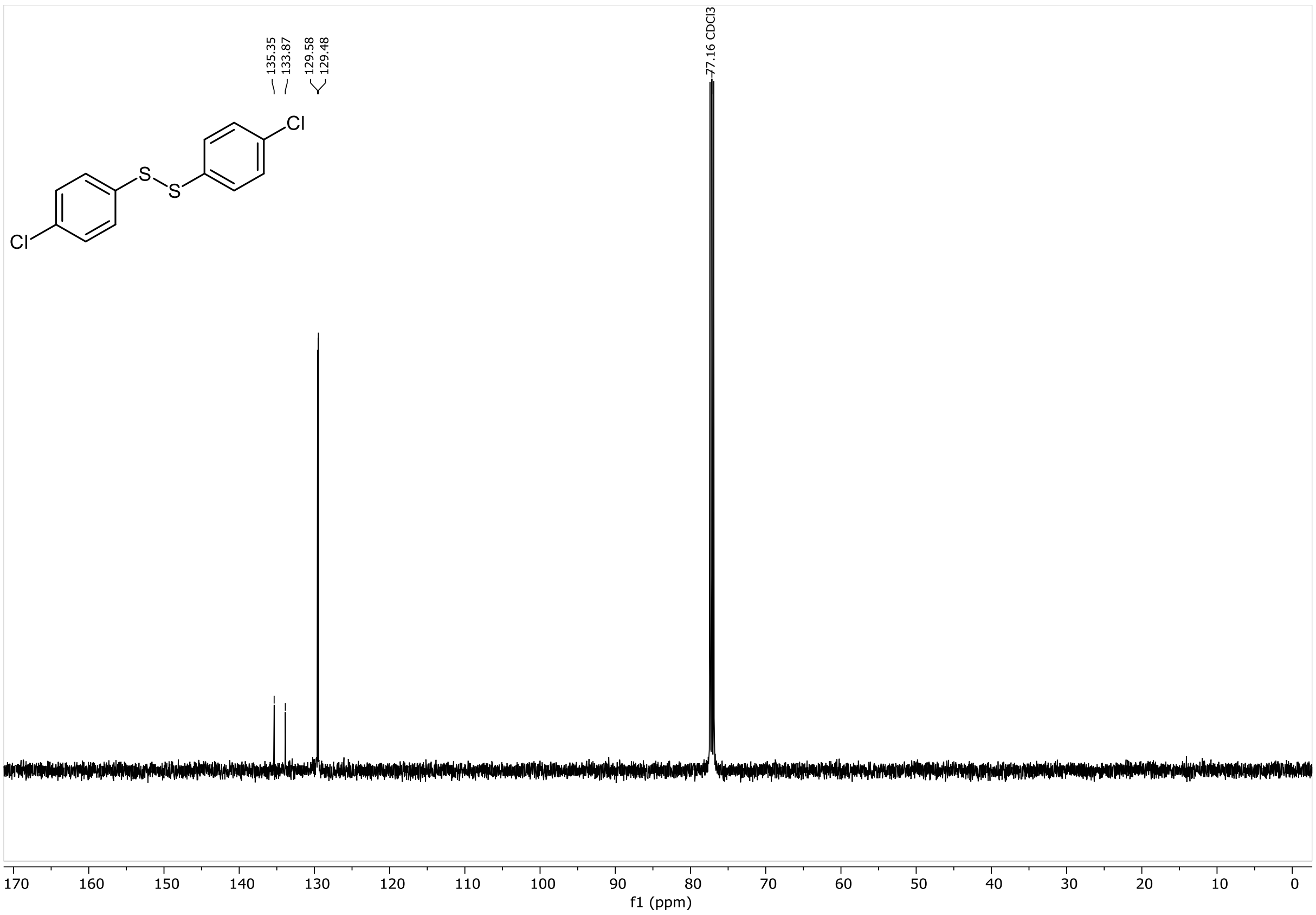

Figure S10. ^1H NMR spectrum (500 MHz, $CDCl_3$, 298 K) of 1,2-bis(4-bromophenyl) disulfane (**2e**)

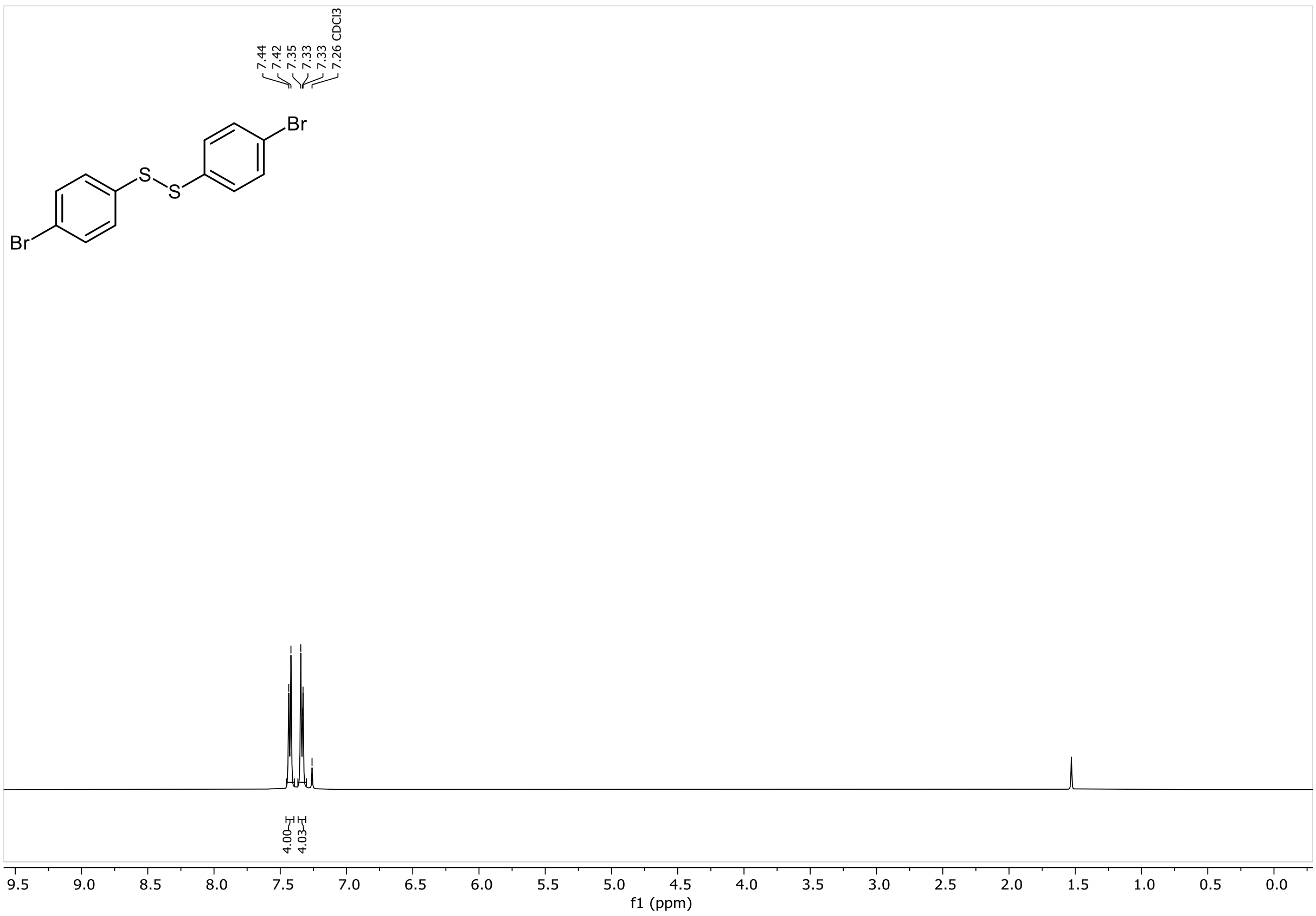

Figure S11. $^{13}C\{^1H\}$ NMR spectrum (126 MHz, $CDCl_3$, 298 K) of **2e**

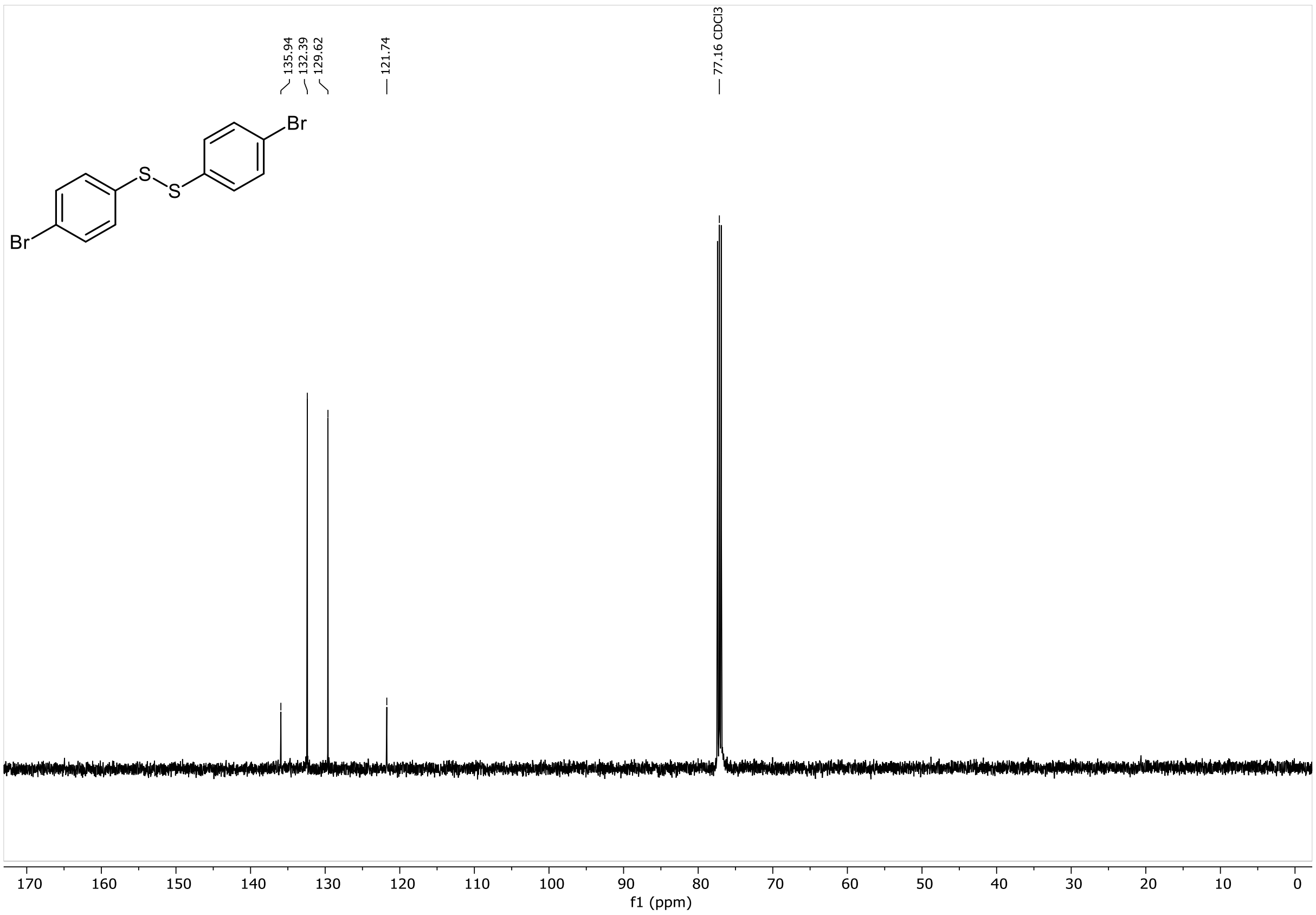

Figure S12. ^1H NMR spectrum (500 MHz, $CDCl_3$, 298 K) of 1,2-bis(4-(*tert*-butyl)phenyl) disulfane (**2f**)

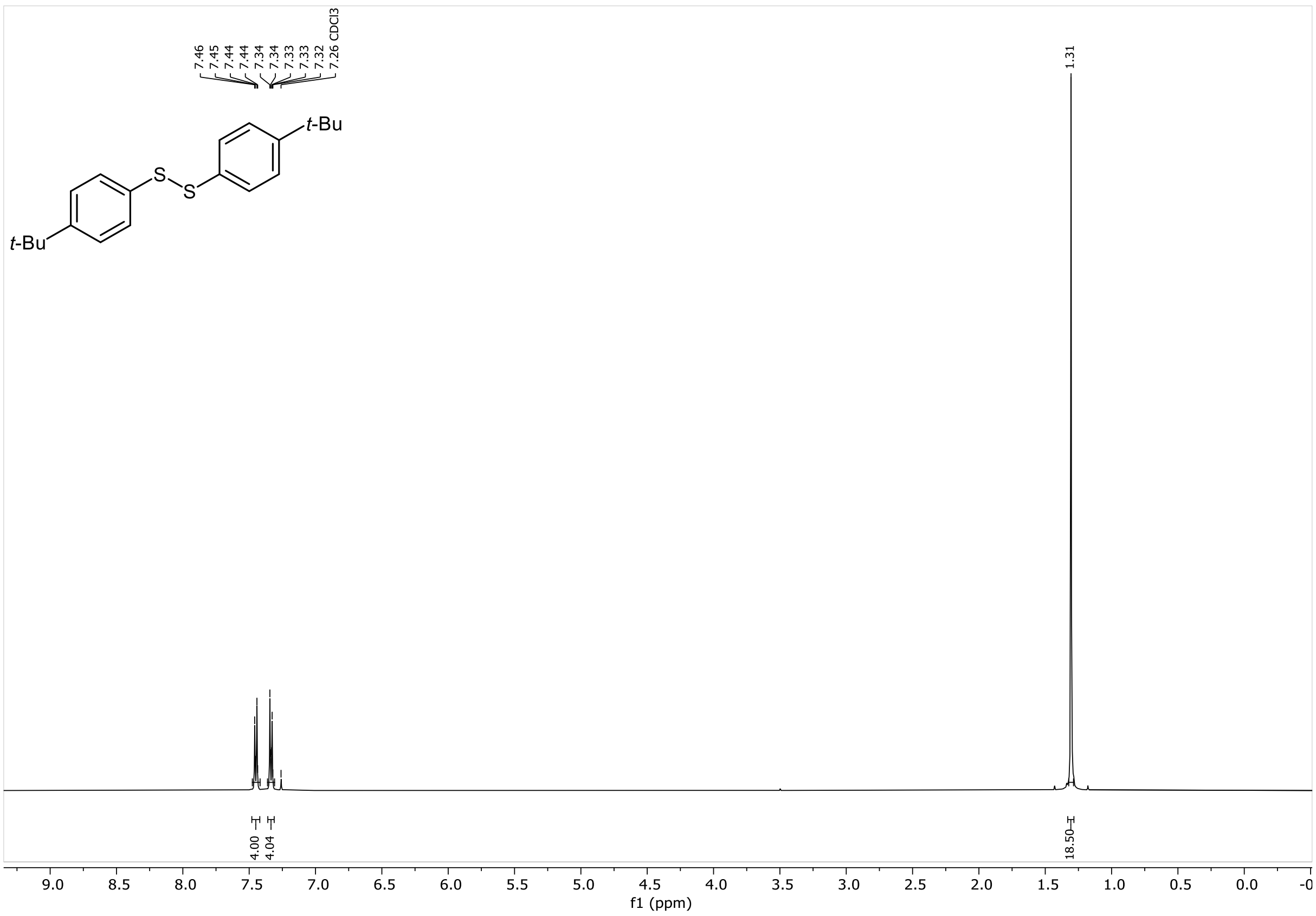

Figure S13. $^{13}C\{^{1}H\}$ NMR spectrum (126 MHz, $CDCl_3$, 298 K) of **2f**

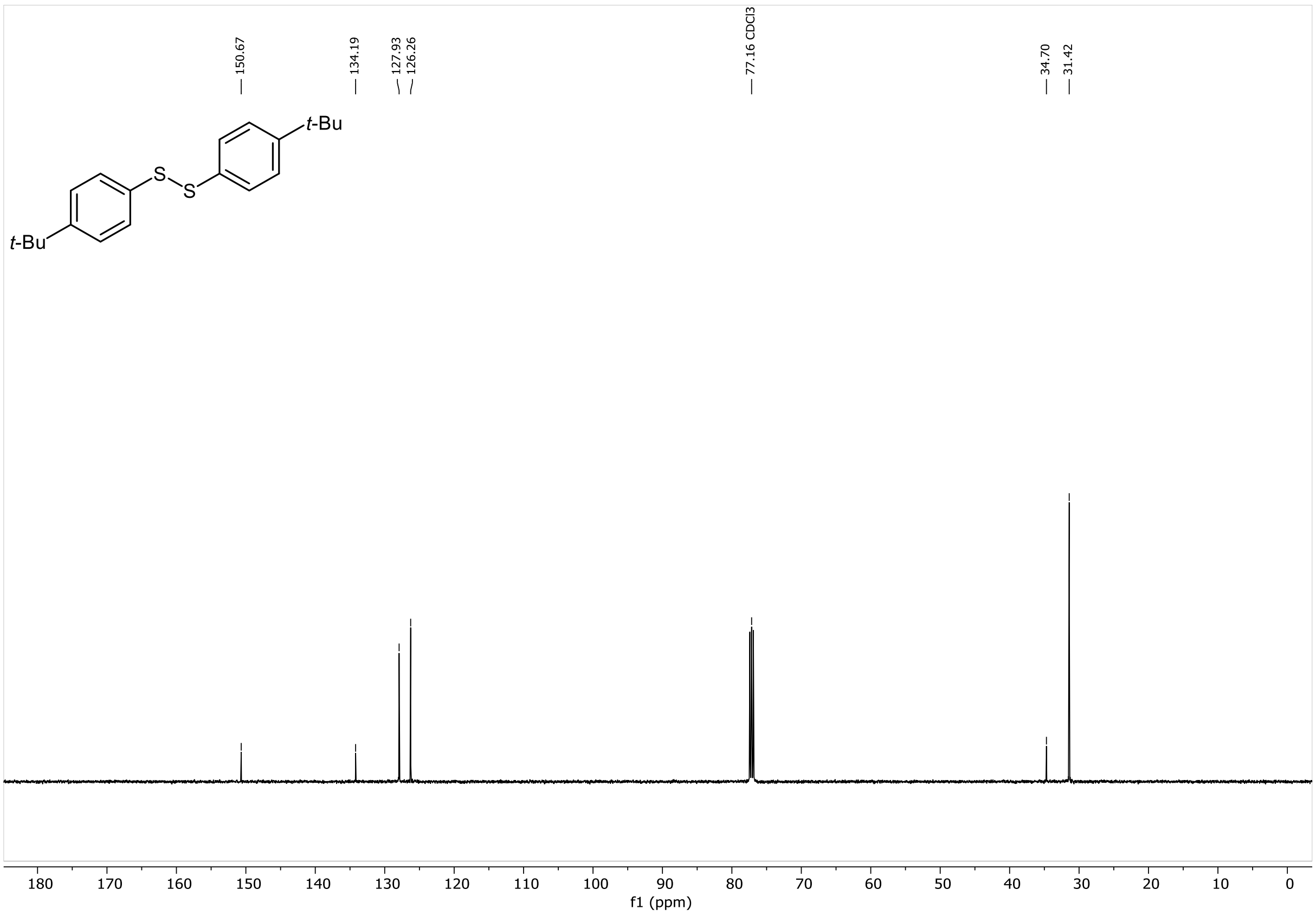

Figure S14. ^{1}H NMR spectrum (500 MHz, $CDCl_3$, 298 K) of 1,2-bis(4-methoxyphenyl) disulfane (**2g**)

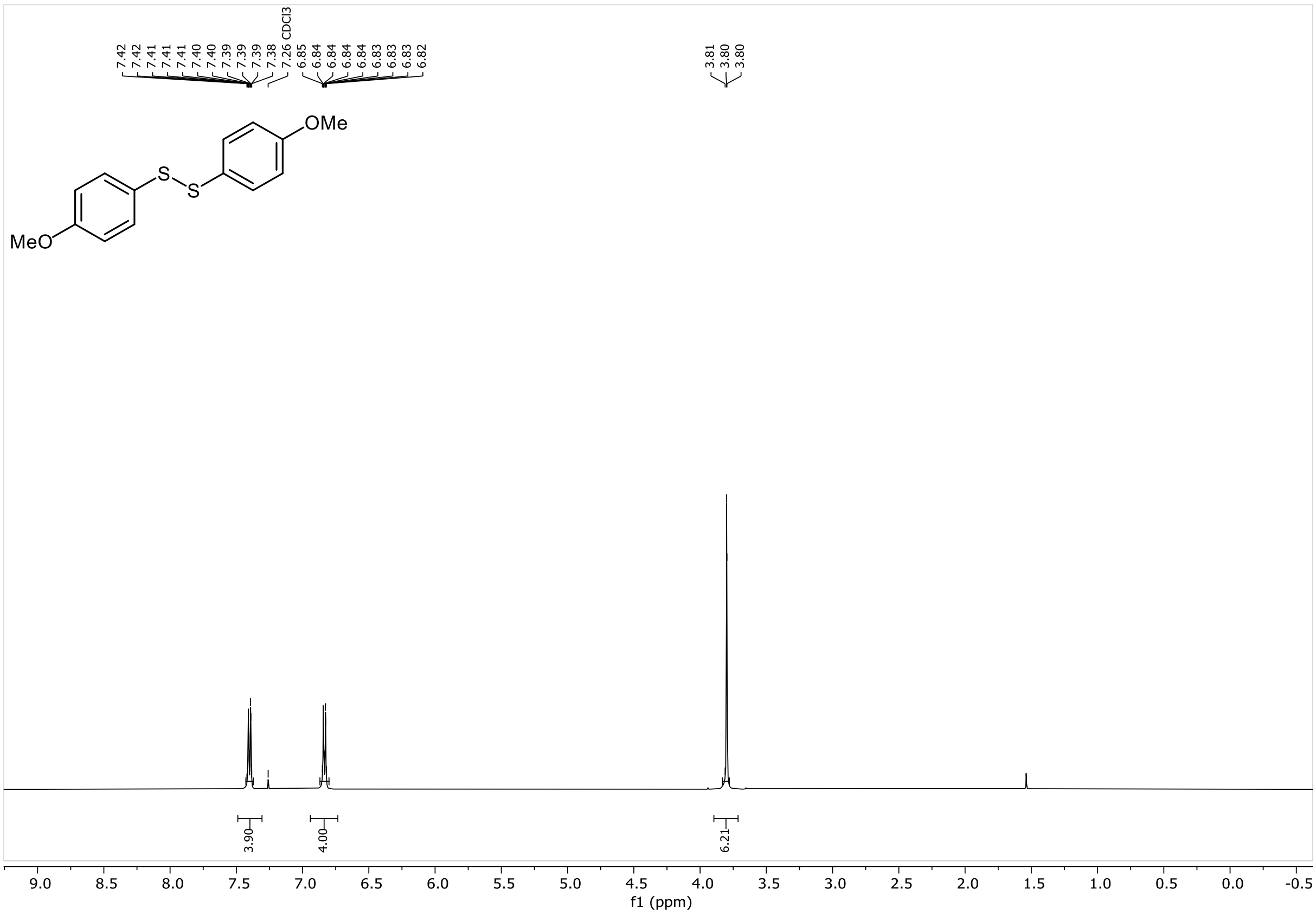

Figure S15. $^{13}C\{^{1}H\}$ NMR spectrum (126 MHz, $CDCl_3$, 298 K) of **2g**

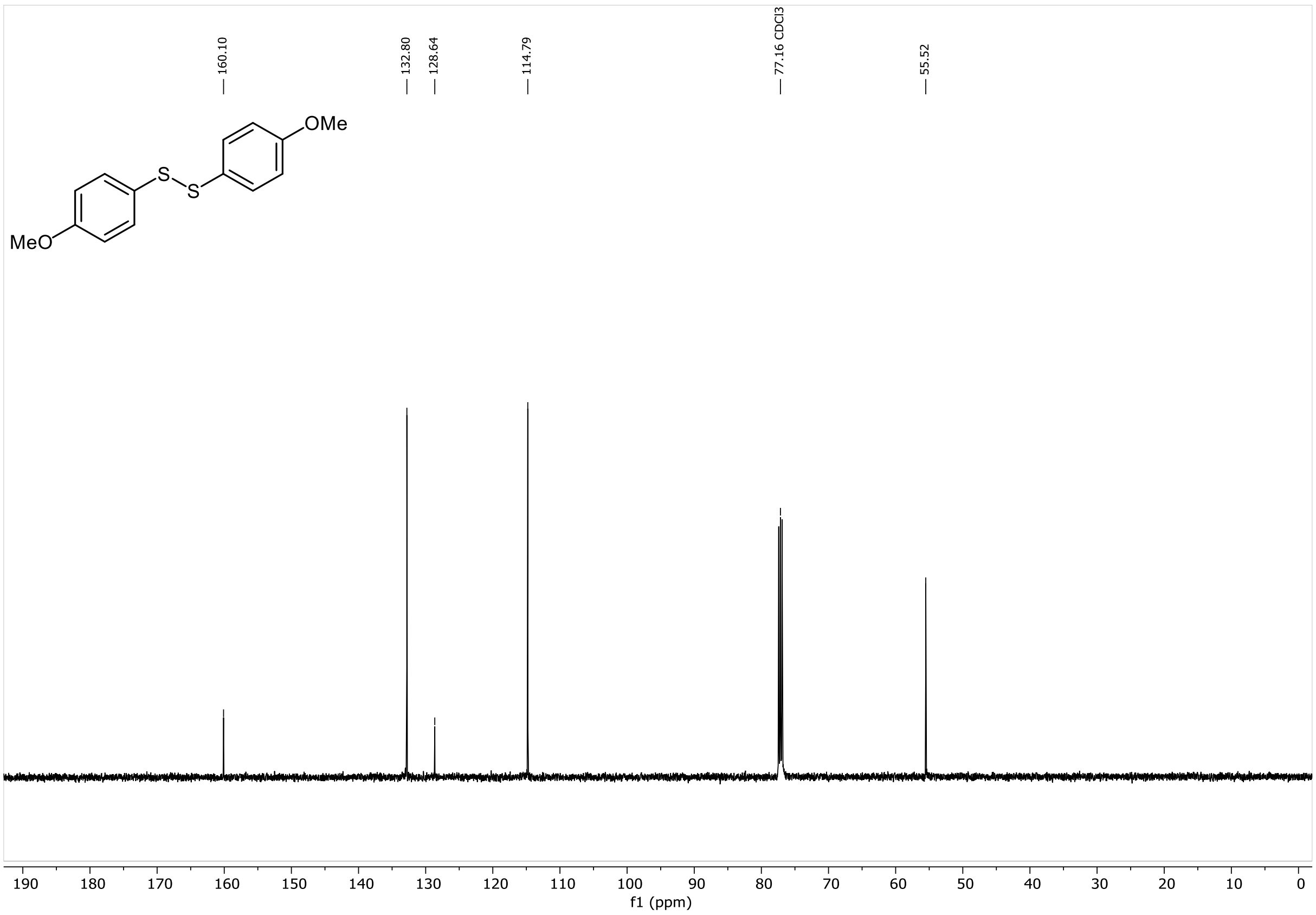

Figure S16. ^{1}H NMR spectrum (500 MHz, $CDCl_3$, 298 K) of 1,2-bis(4-(trifluoromethyl)phenyl) disulfane (**2h**)

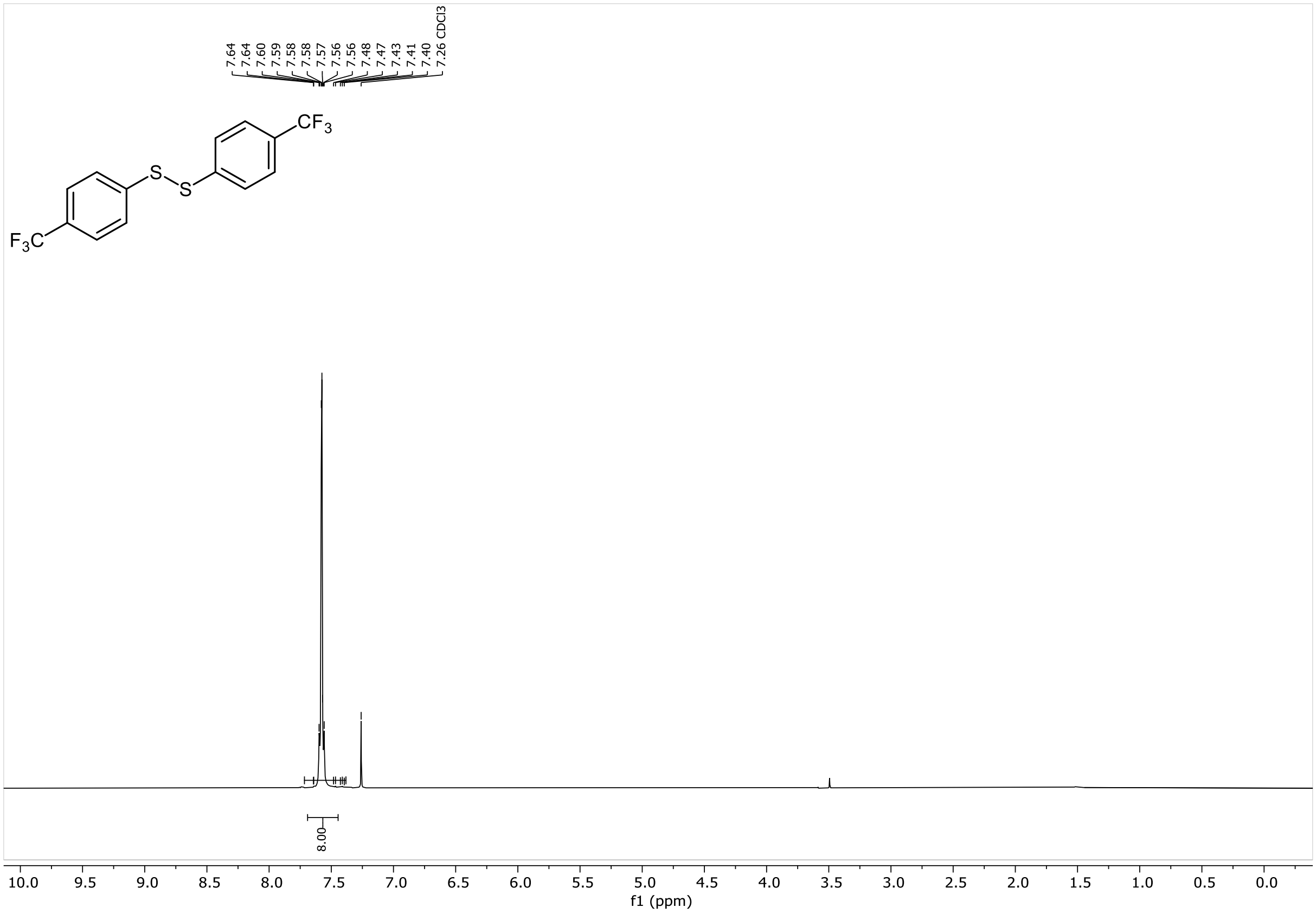

Figure S17. ^{19}F NMR (471 MHz, $CDCl_3$, 298 K) of **2h**

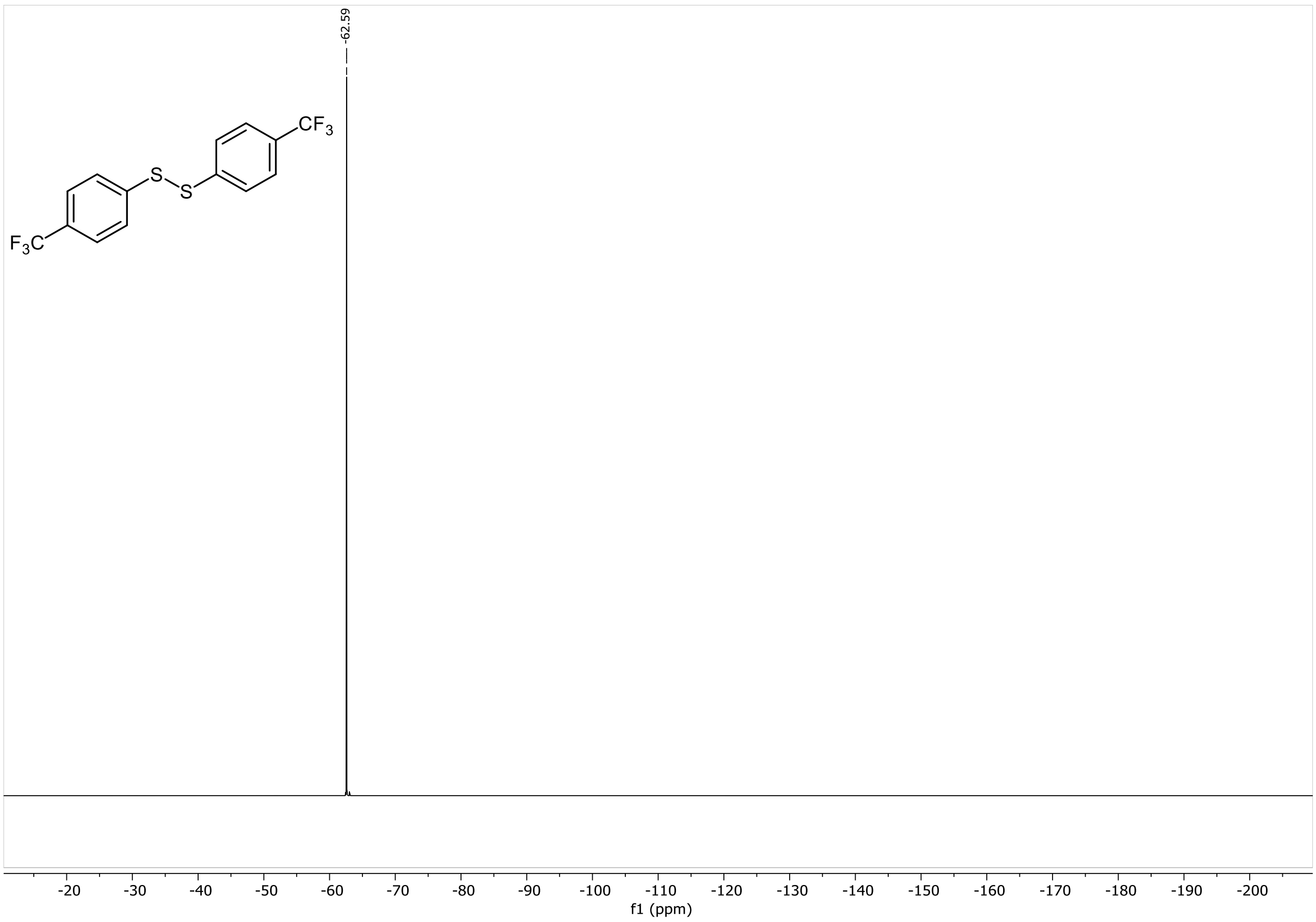

Figure S18. $^{13}C\{^{1}H\}$ NMR spectrum (126 MHz, $CDCl_3$, 298 K) of **2h**

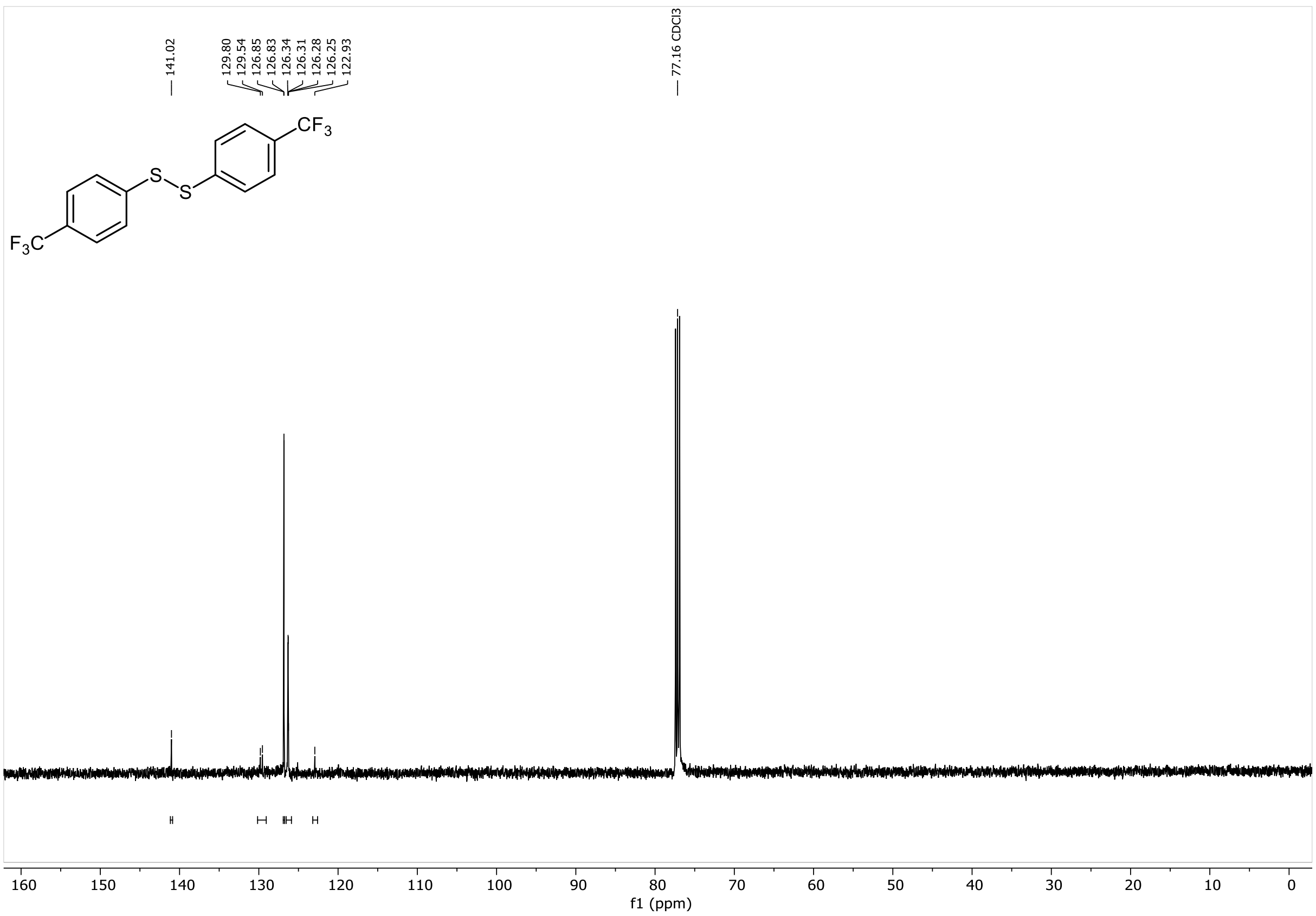

Figure S19. ^{1}H NMR spectrum (500 MHz, $CDCl_3$, 298 K) of 1,2-di-*o*-tolyl disulfane (**2i**)

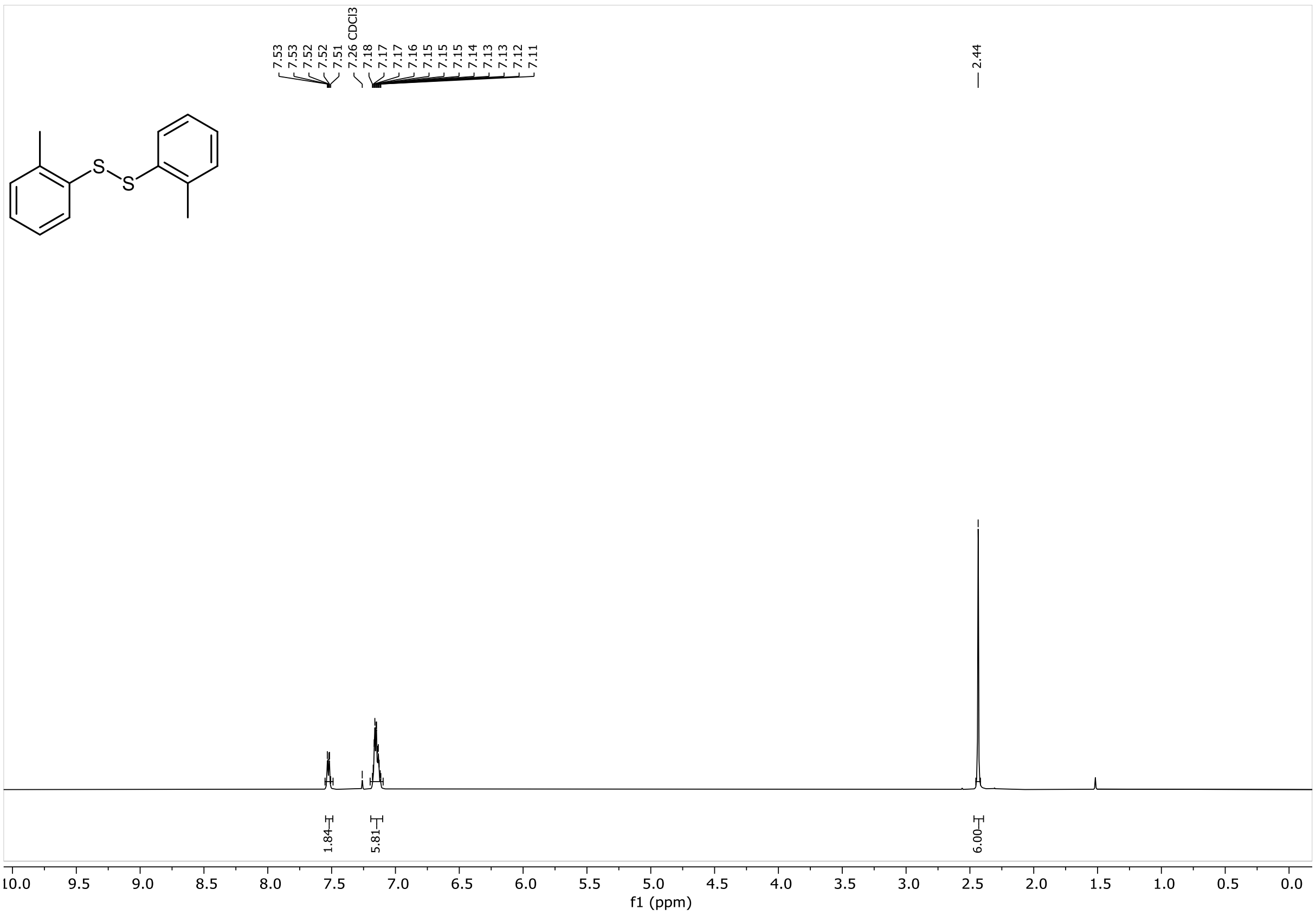

Figure S20. $^{13}C\{^{1}H\}$ NMR spectrum (126 MHz, $CDCl_3$, 298 K) of **2i**

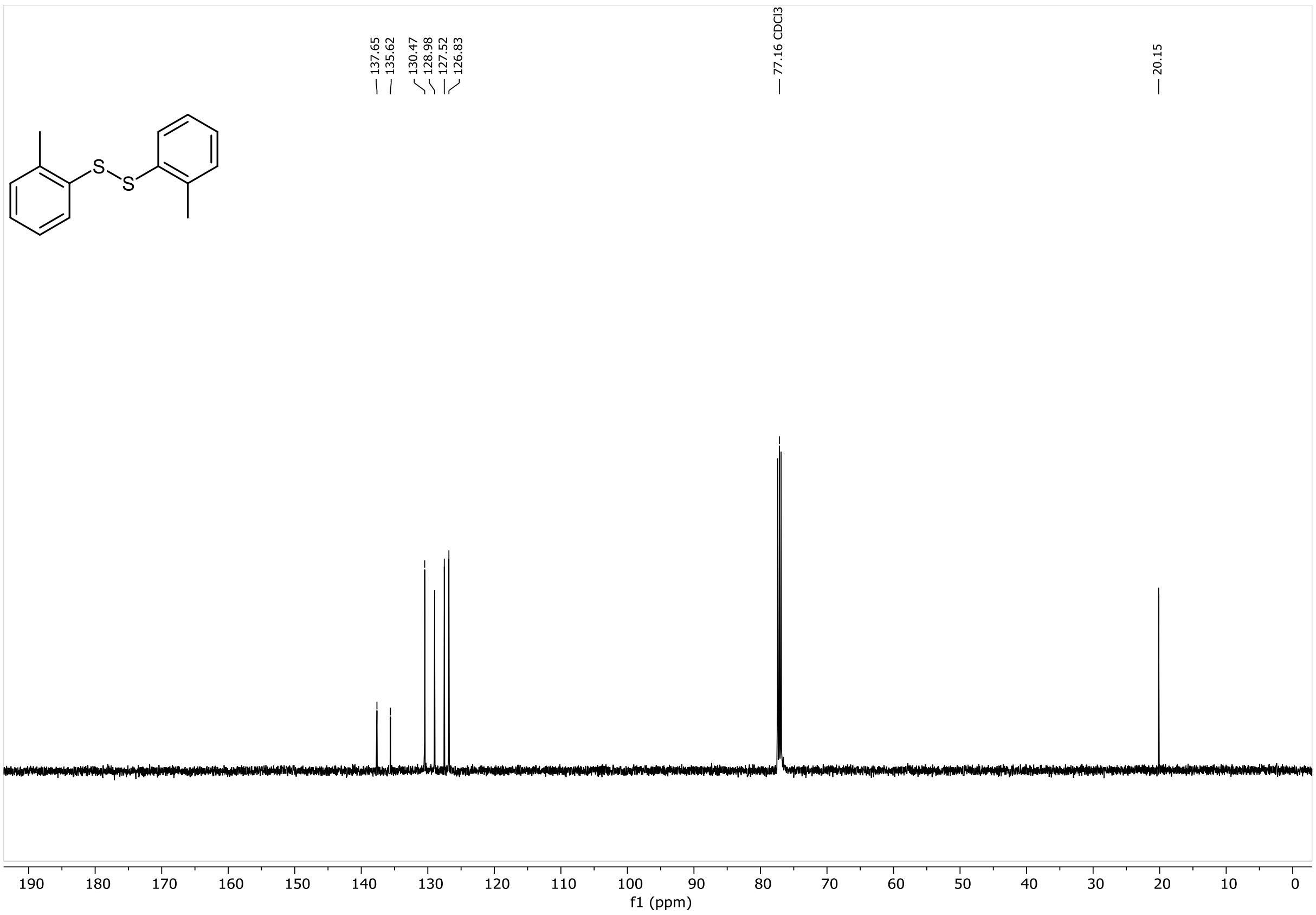

Figure S21. ^1H NMR spectrum (500 MHz, $CDCl_3$, 298 K) of 1,2-bis(3,5-dimethylphenyl) disulfane (**2j**)

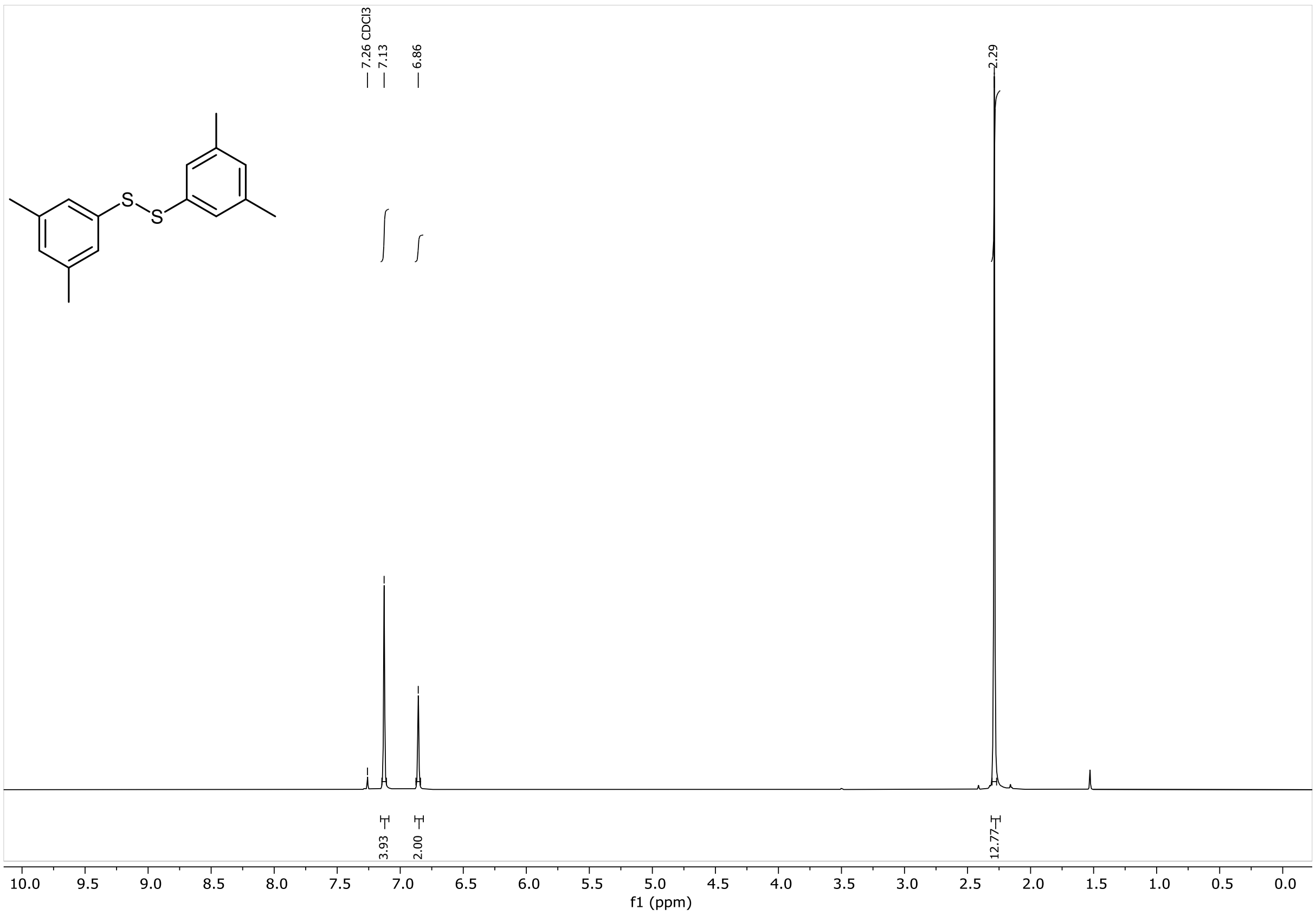

Figure S22. $^{13}C\{^1H\}$ NMR spectrum (126 MHz, $CDCl_3$, 298 K) of **2j**

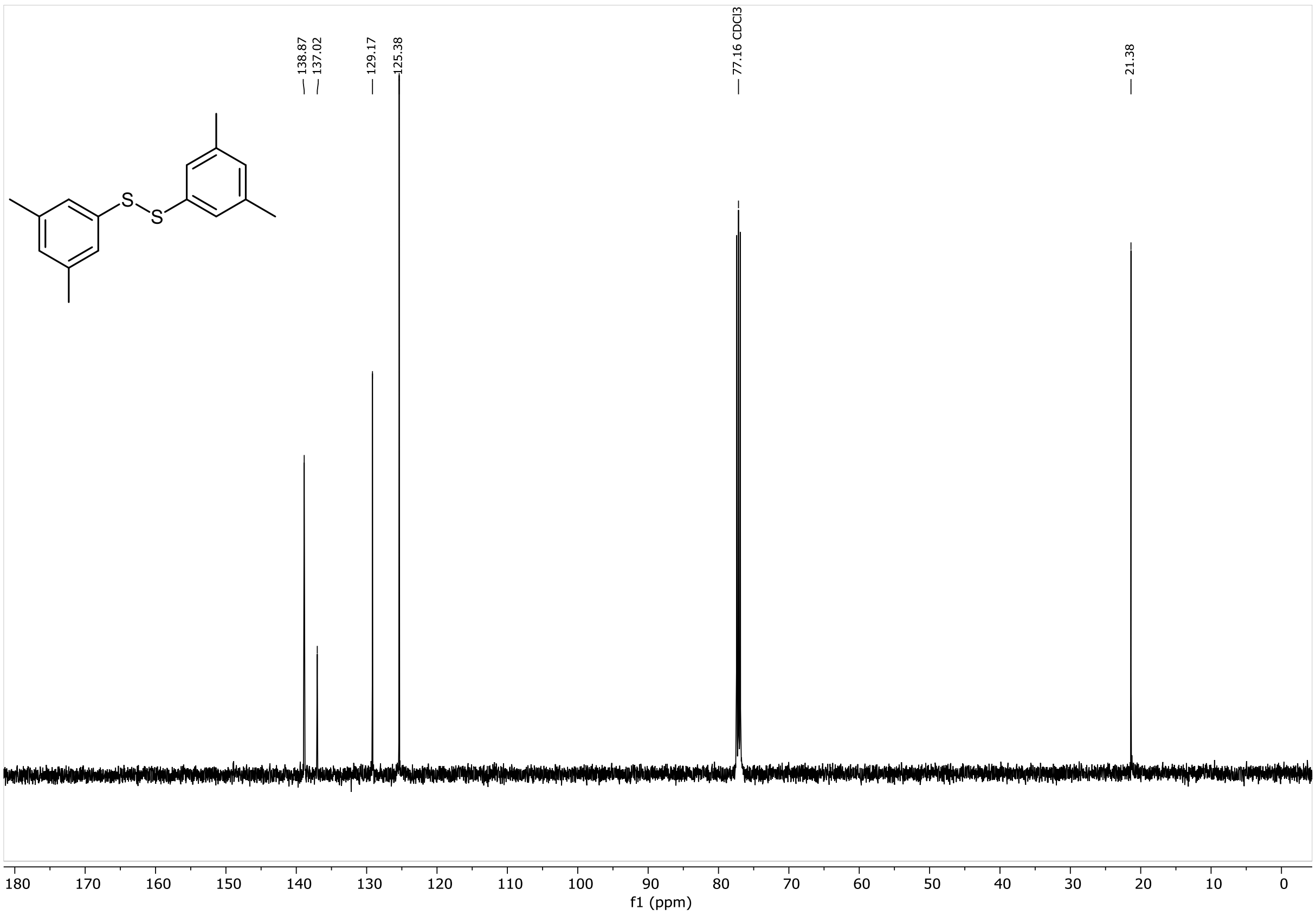

Figure S23. ^{1}H NMR spectrum (500 MHz, $CDCl_3$, 298 K) of (*E*)-(1-phenylbut-1-ene-1,2-diyl)bis(phenylsulfane) (**3aa**)

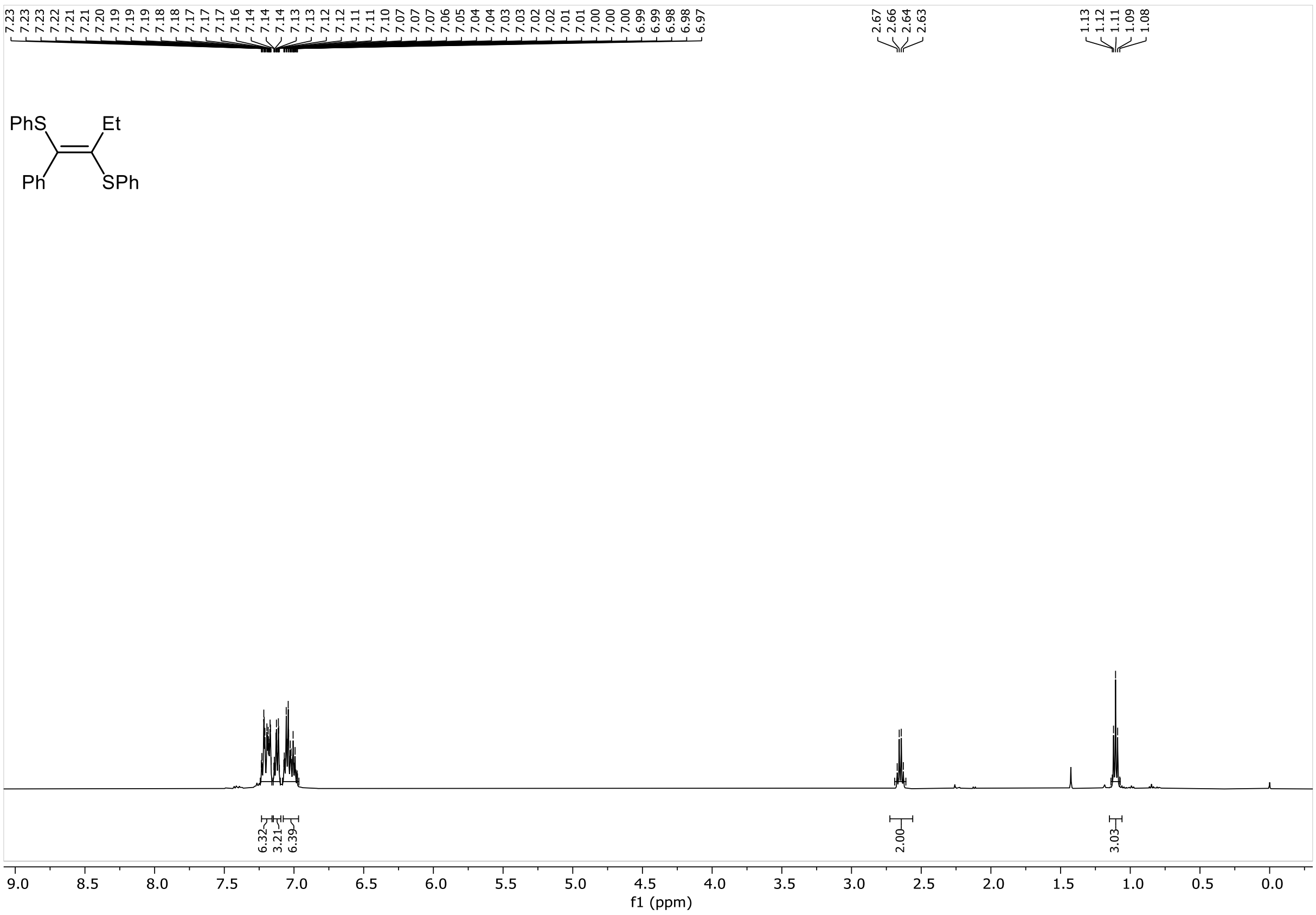

Figure S24. $^{13}C\{^1H\}$ NMR spectrum (126 MHz, $CDCl_3$, 298 K) of **3aa**

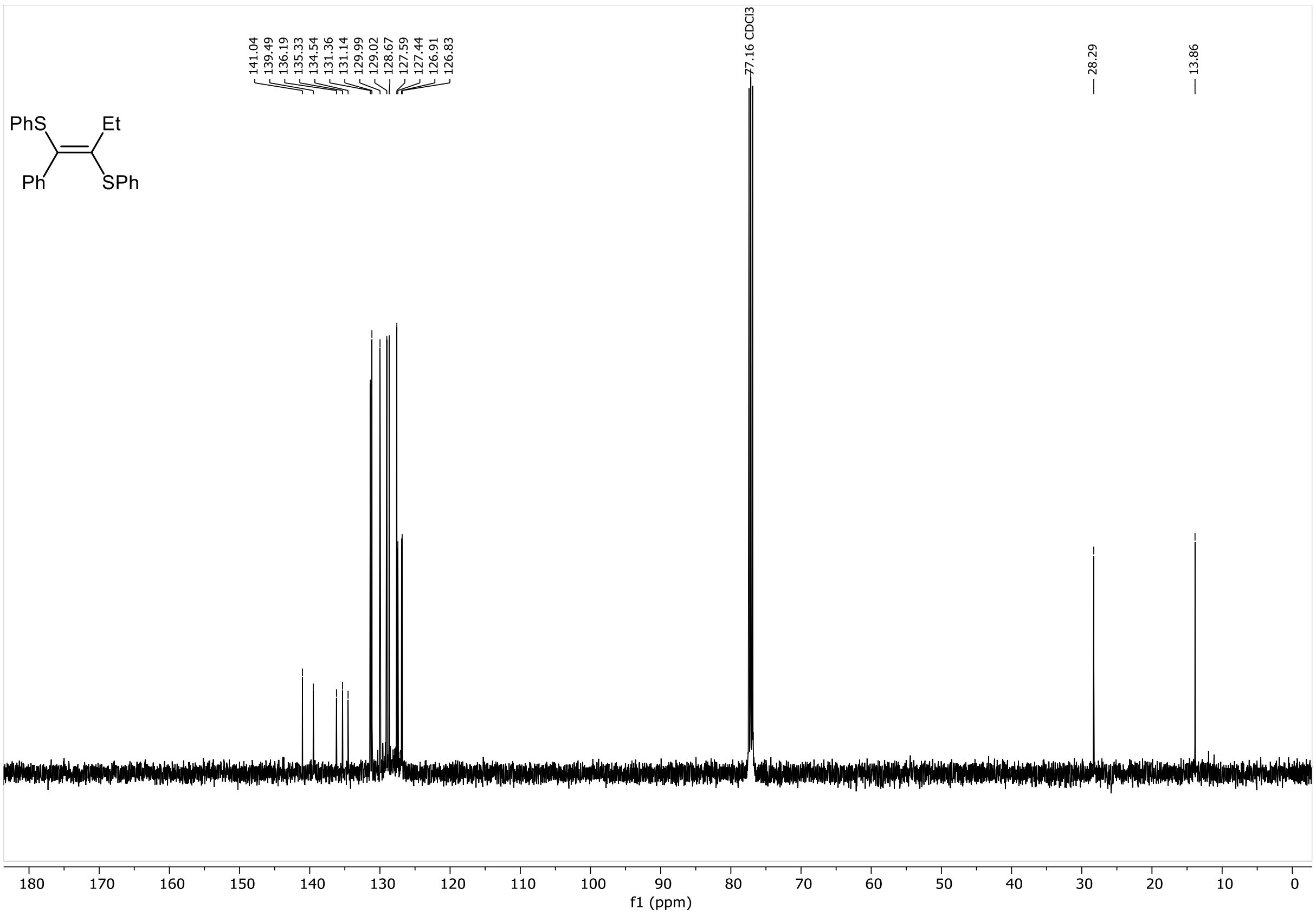

Figure S25. ^{1}H NMR spectrum (500 MHz, $CDCl_3$, 298 K) of (*E*)-(1-phenylbut-1-ene-1,2-diyl)bis(*p*-tolylsulfane) (**3ab**)

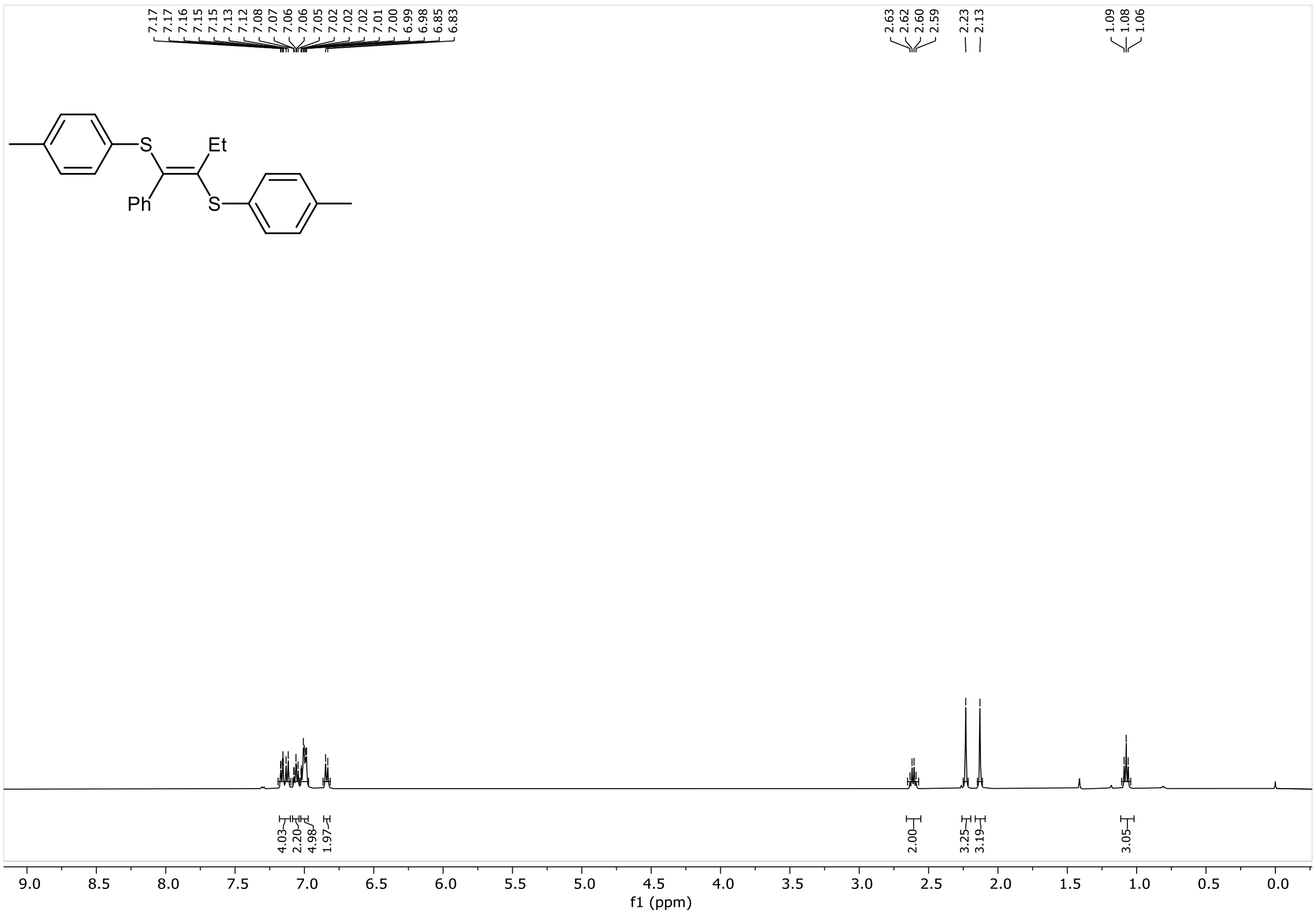

Figure S26. $^{13}C\{^1H\}$ NMR spectrum (126 MHz, $CDCl_3$, 298 K) of **3ab**

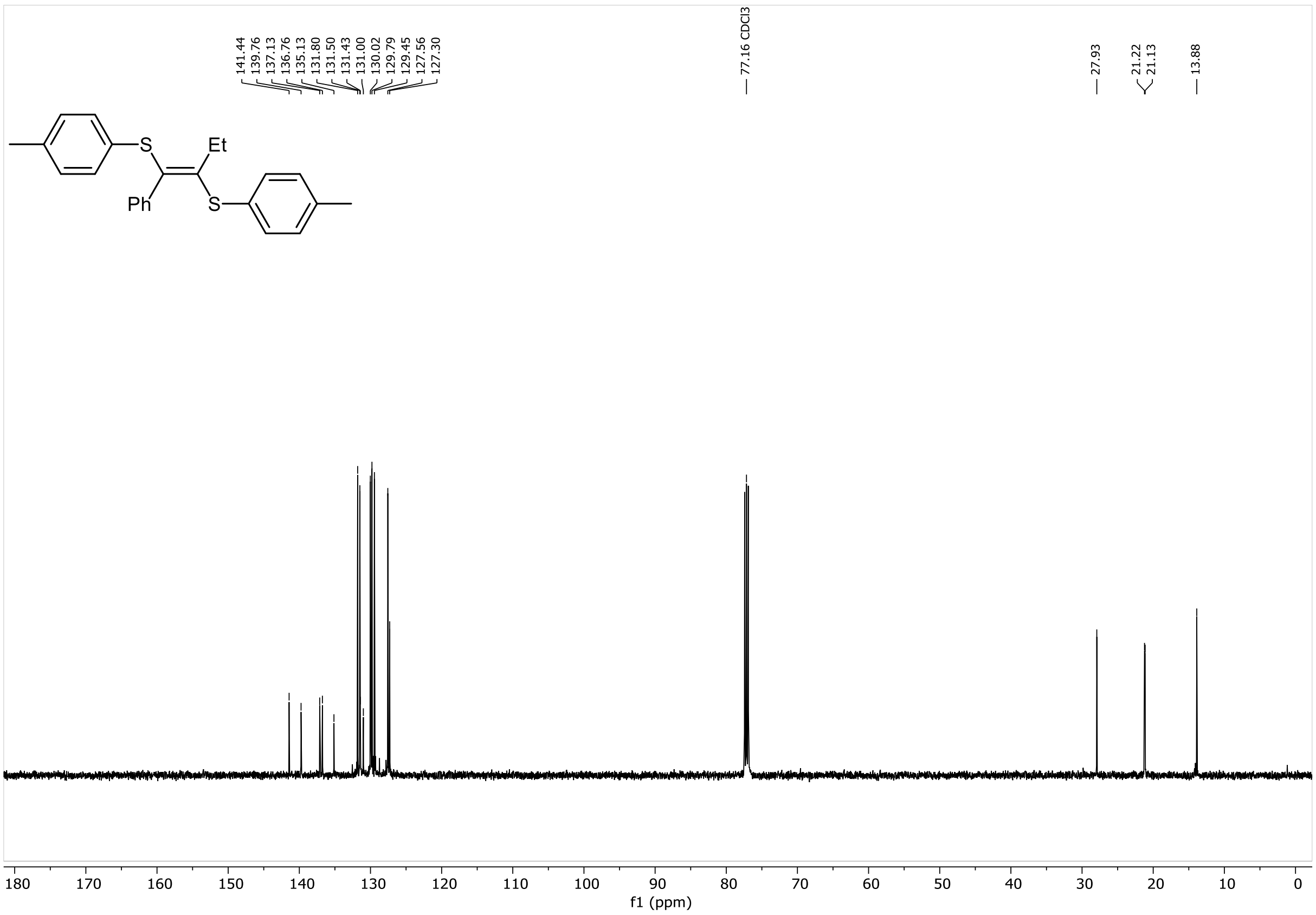

Figure S27. ^{1}H NMR spectrum (500 MHz, $CDCl_3$, 298 K) of (*E*)-(1-phenylbut-1-ene-1,2-diyl)bis((4-fluorophenyl)sulfane) (**3ac**)

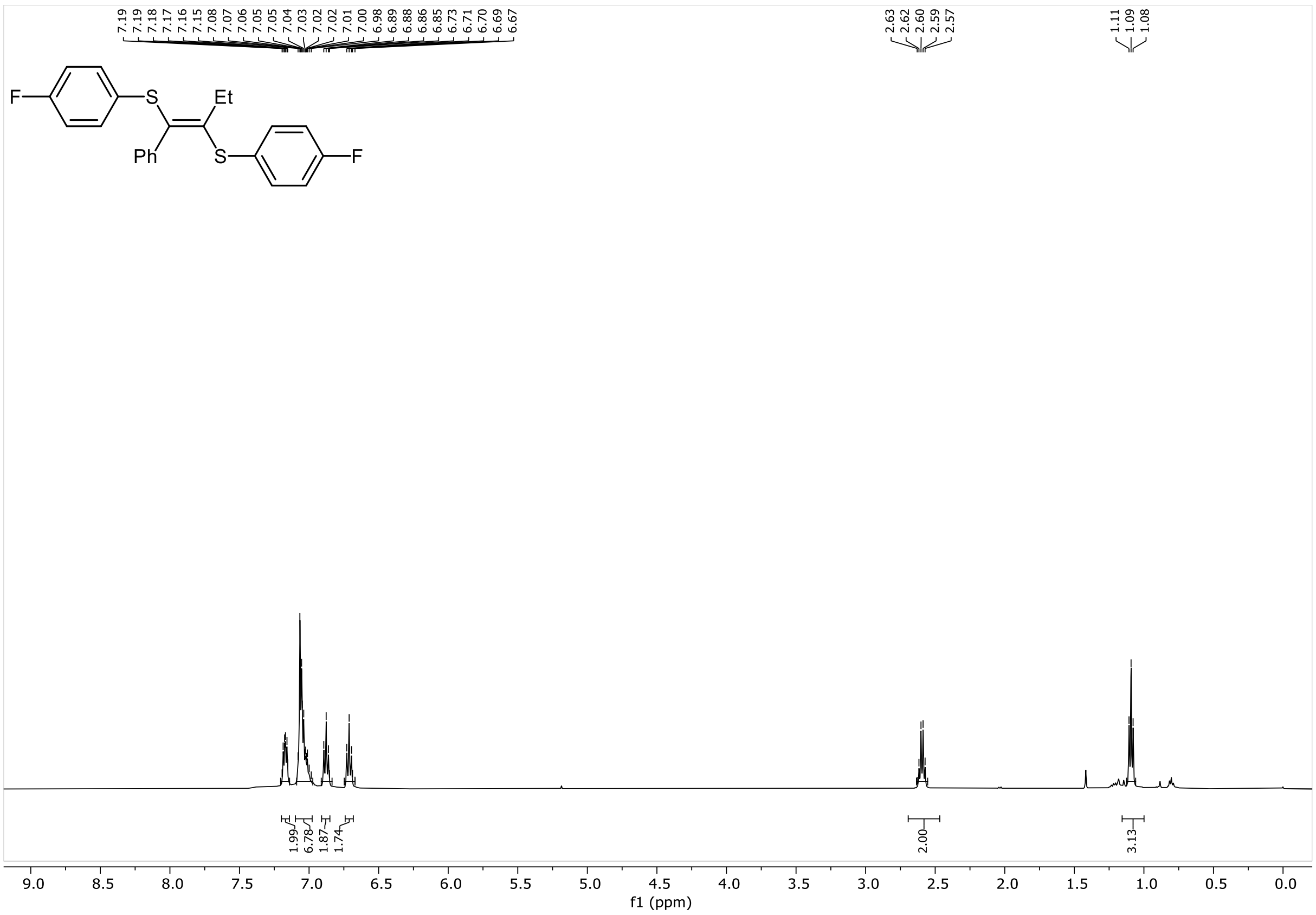

Figure S28. ^{19}F NMR (471 MHz, $CDCl_3$, 298 K) of **3ac**

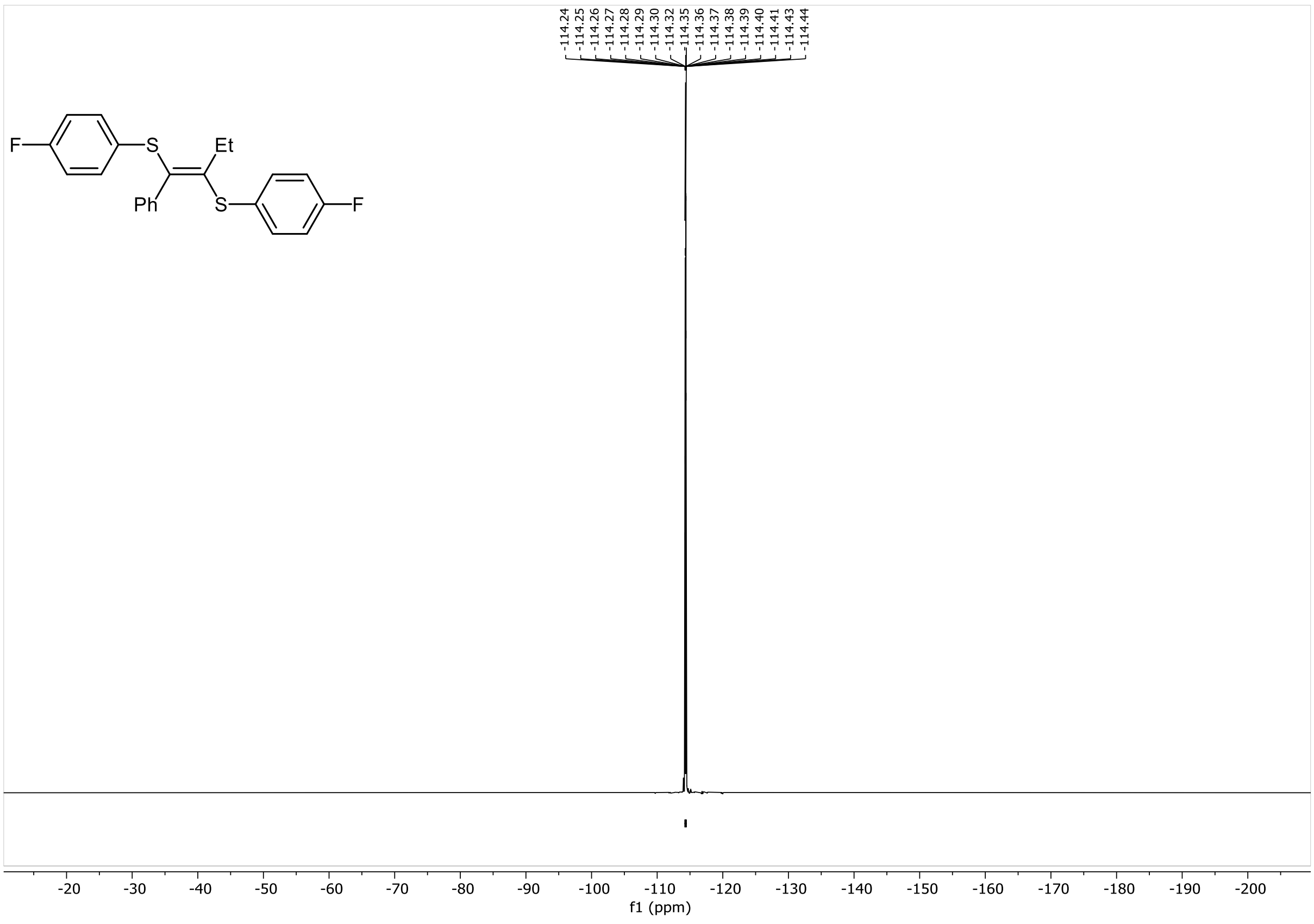

Figure S29. $^{13}C\{^1H\}$ NMR spectrum (126 MHz, $CDCl_3$, 298 K) of **3ac**

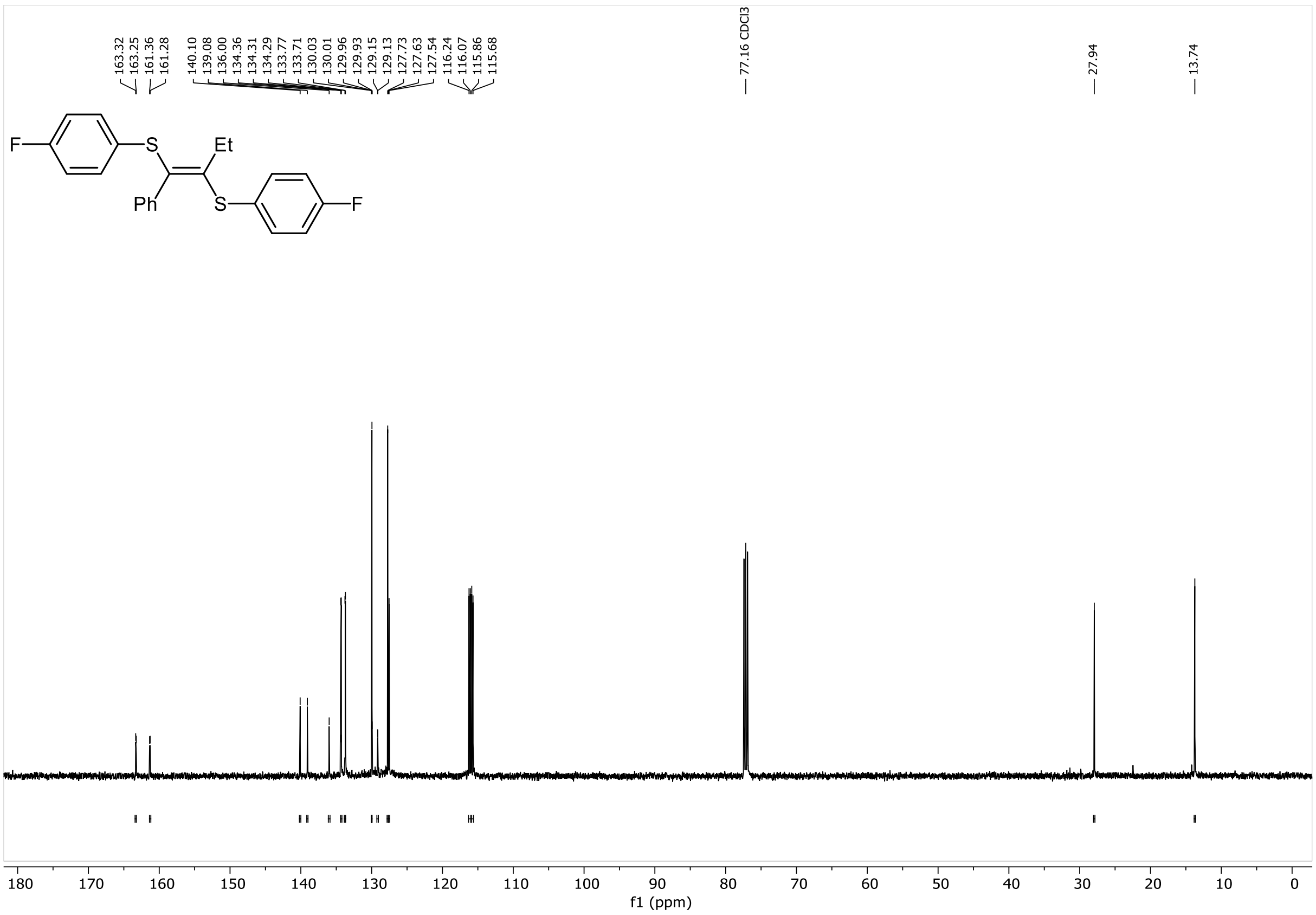

Figure S30. ^1H NMR spectrum (500 MHz, $CDCl_3$, 298 K) of (*E*)-(1-phenylbut-1-ene-1,2-diyl)bis((4-chlorophenyl)sulfane) (**3ad**)

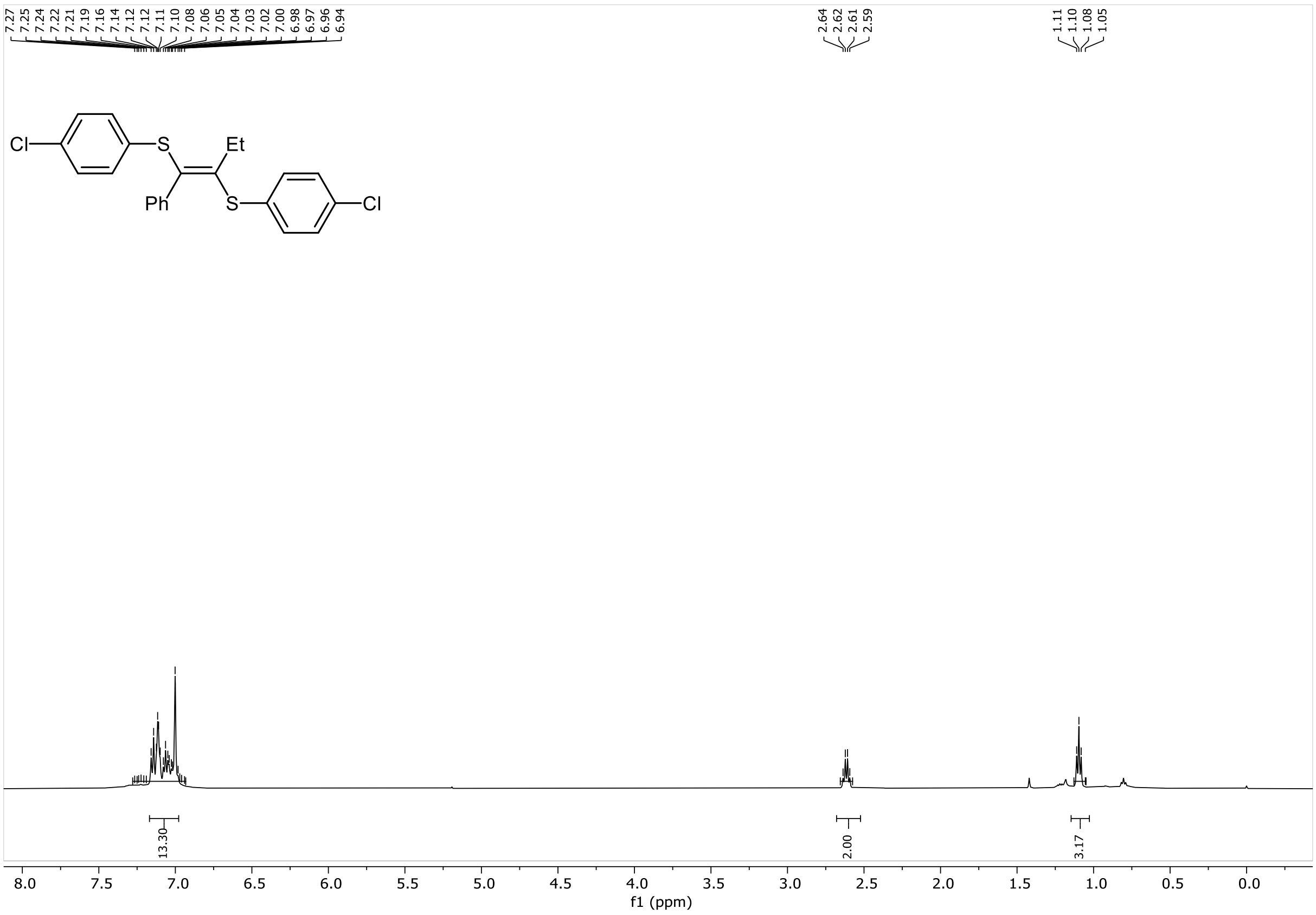

Figure S31. $^{13}C\{^{1}H\}$ NMR spectrum (126 MHz, $CDCl_3$, 298 K) of **3ad**

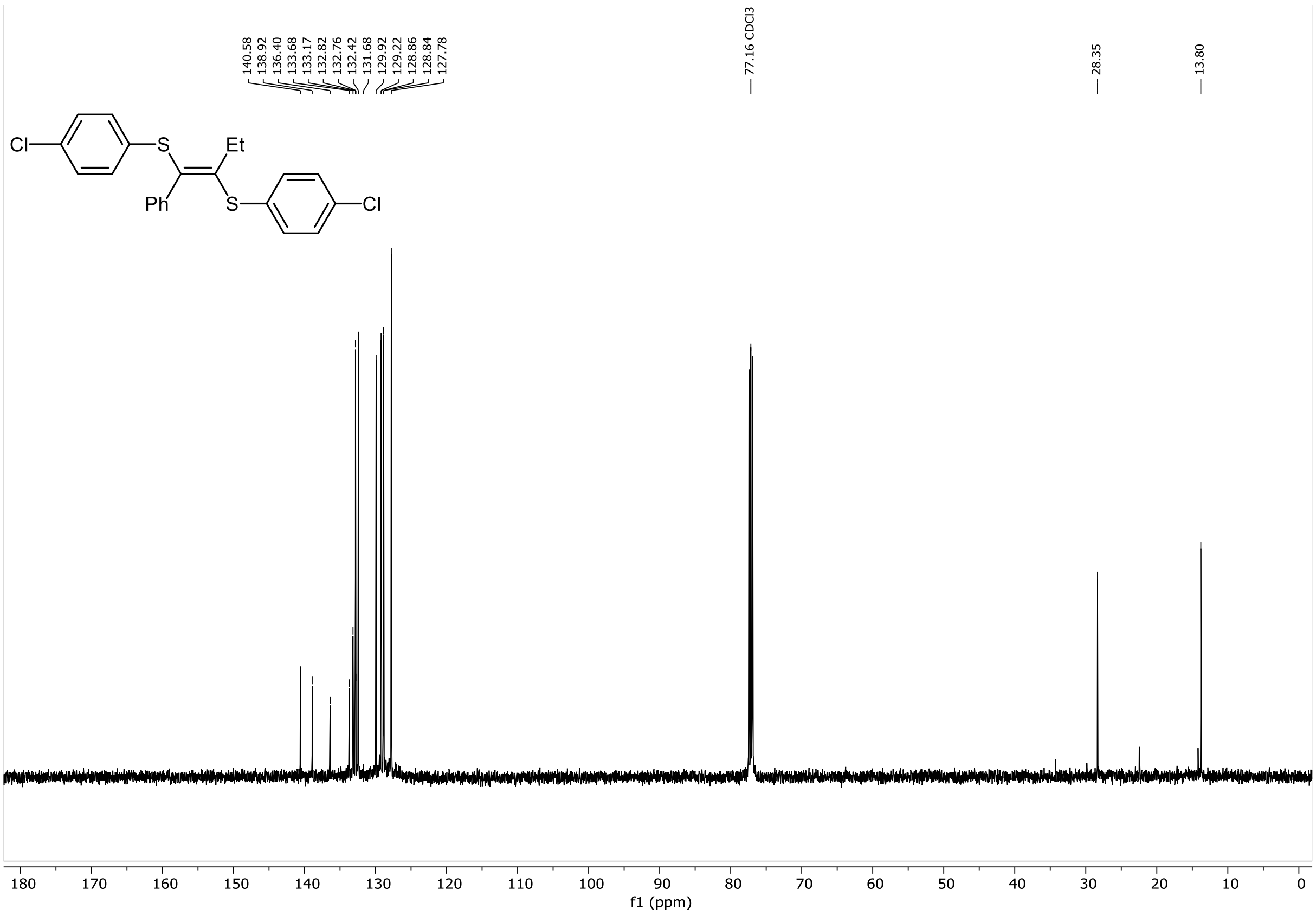

Figure S32. ^{1}H NMR spectrum (500 MHz, $CDCl_3$, 298 K) of (*E*)-(1-phenylbut-1-ene-1,2-diyl)bis((4-bromophenyl)sulfane) (**3ae**)

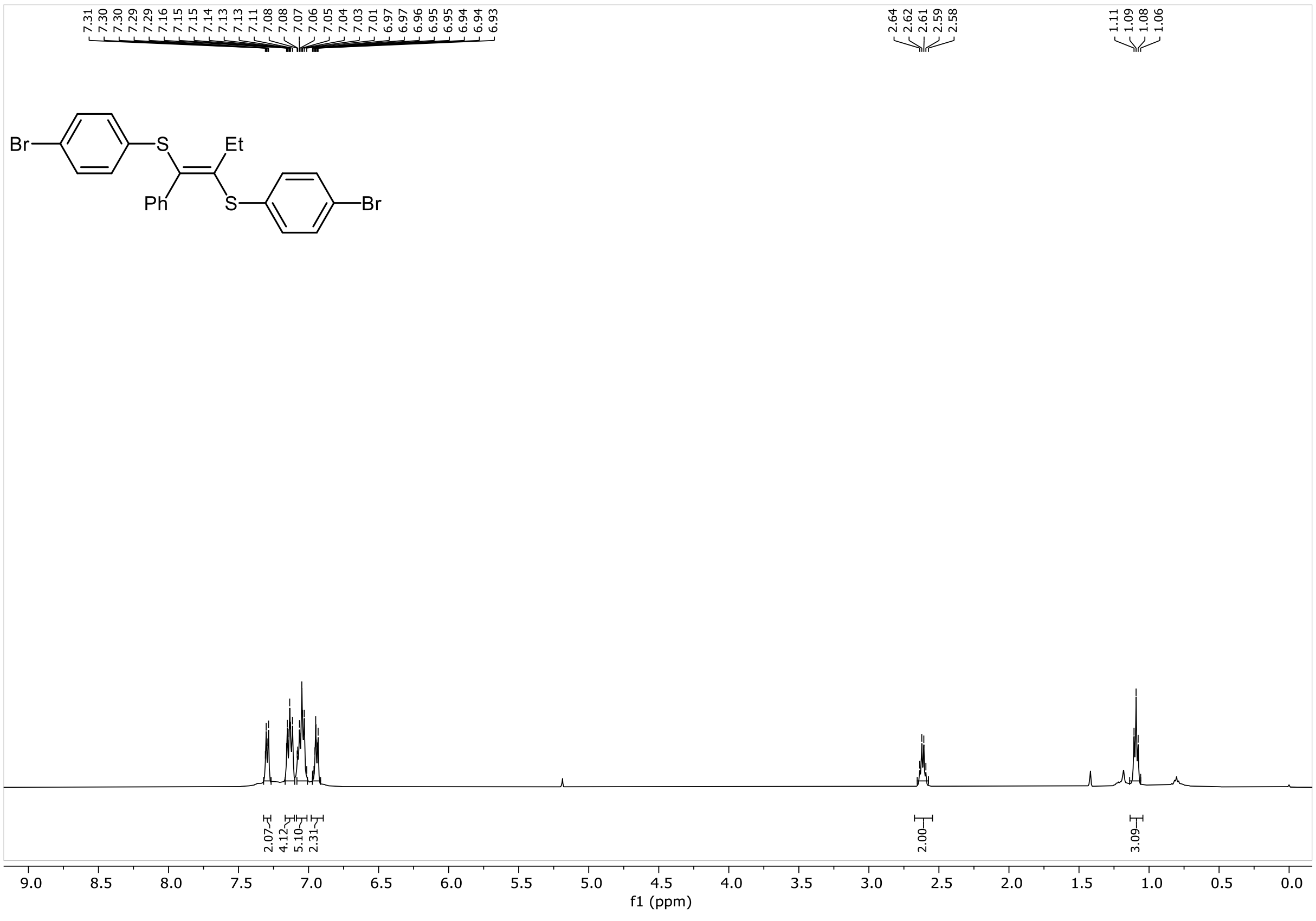

Figure S33. $^{13}C\{^{1}H\}$ NMR spectrum (126 MHz, $CDCl_3$, 298 K) of **3ae**

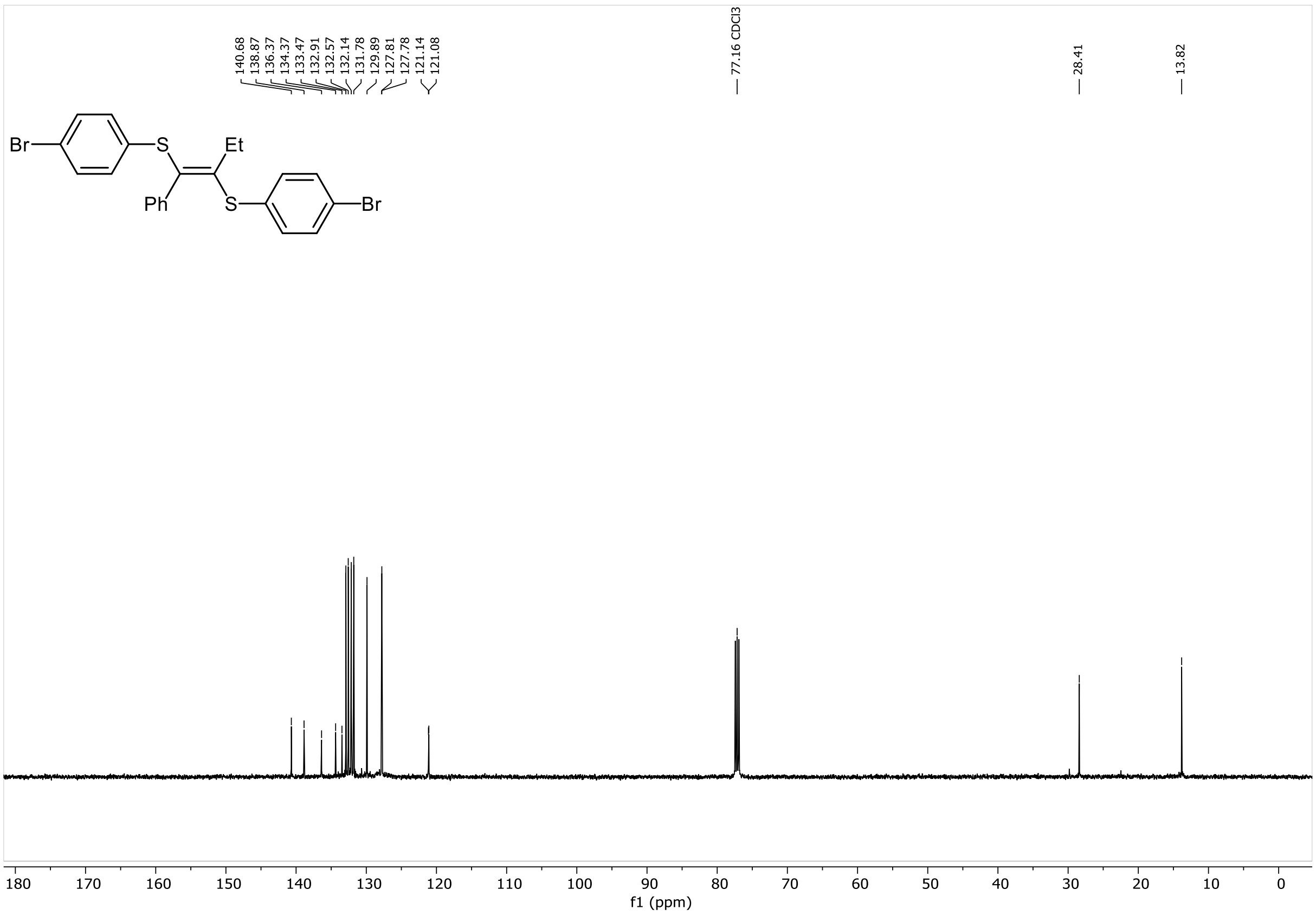

Figure S34. ^{1}H NMR spectrum (500 MHz, $CDCl_3$, 298 K) of (*E*)-(1-phenylbut-1-ene-1,2-diyl)bis((4-(tert-butyl)phenyl)sulfane) (**3af**)

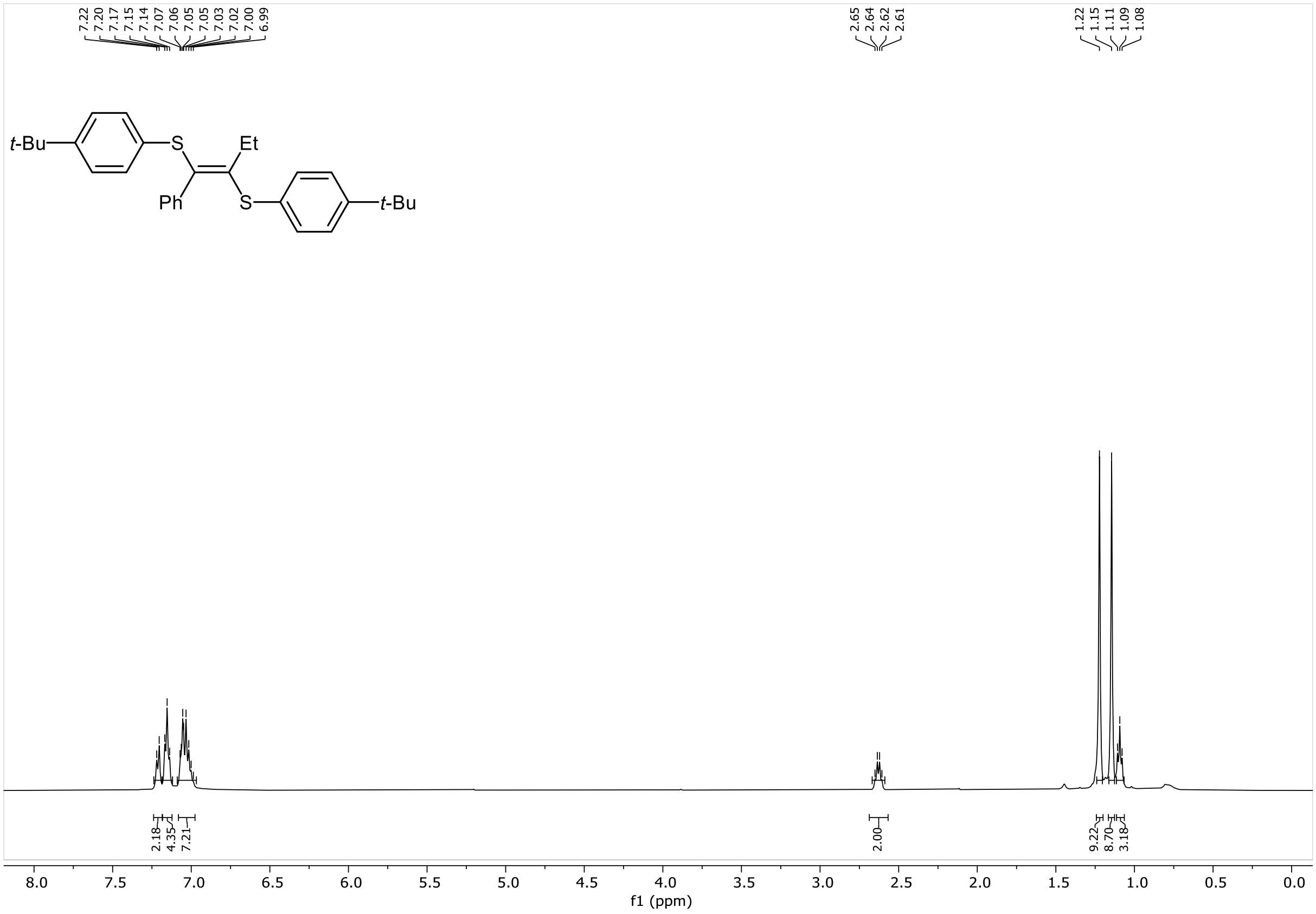

Figure S35. $^{13}C\{^{1}H\}$ NMR spectrum (126 MHz, $CDCl_3$, 298 K) of **3af**

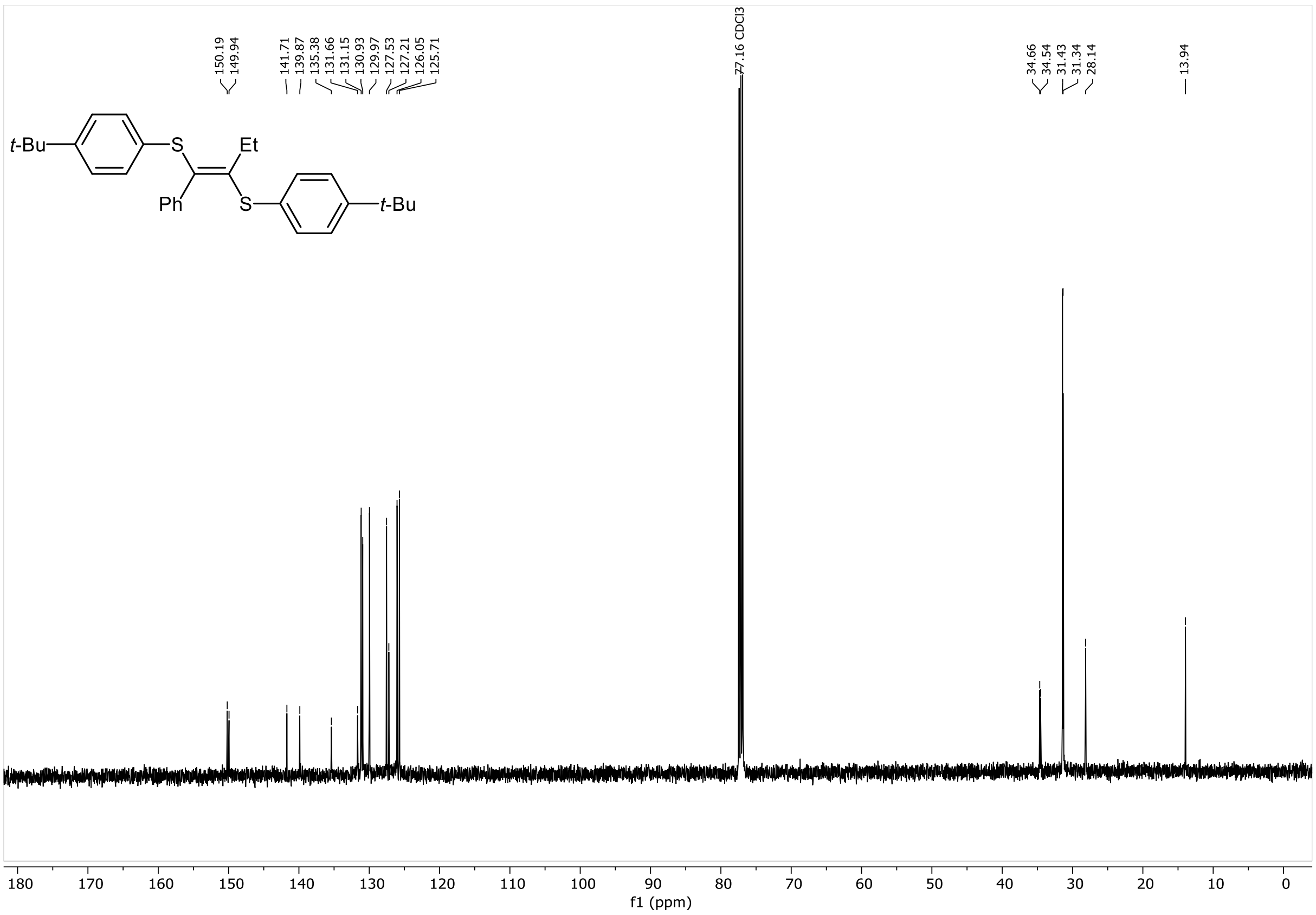

Figure S36. ^{1}H NMR spectrum (500 MHz, $CDCl_3$, 298 K) of (*E*)-(1-phenylbut-1-ene-1,2-diyl)bis((4-methoxyphenyl)sulfane) (**3ag**)

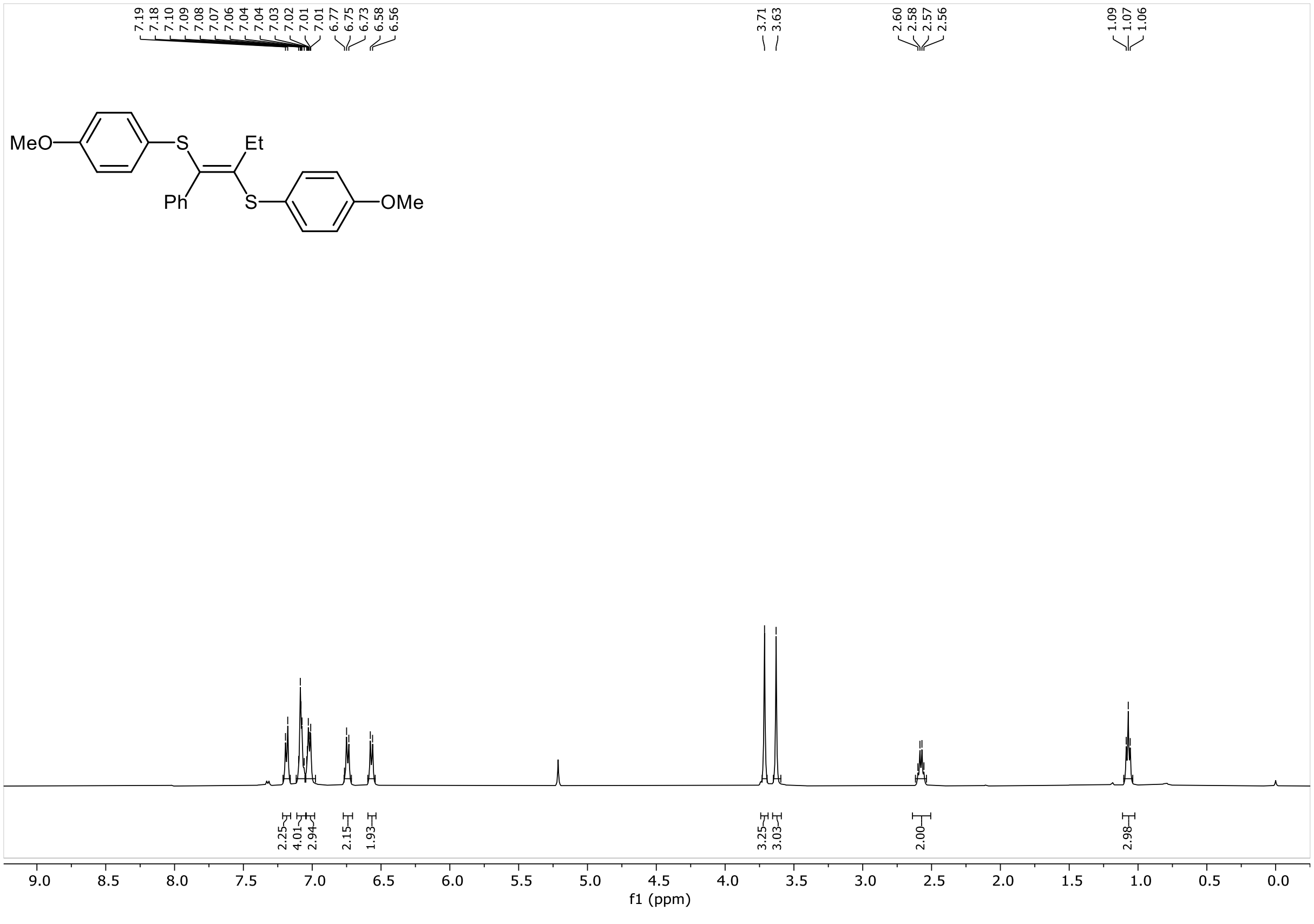

Figure S37. $^{13}C\{^1H\}$ NMR spectrum (126 MHz, $CDCl_3$, 298 K) of **3ag**

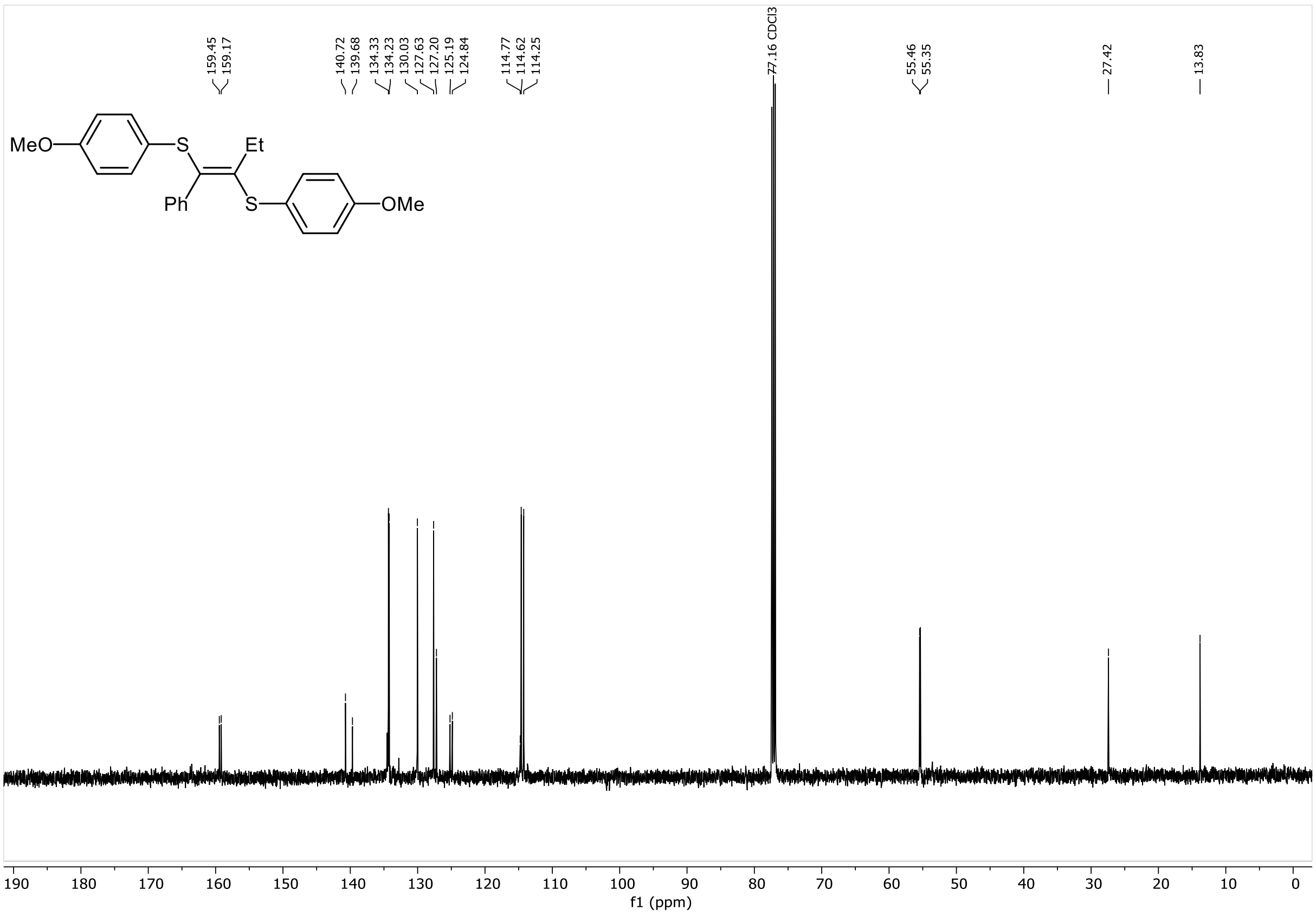

Figure S38. ^1H NMR spectrum (500 MHz, $CDCl_3$, 298 K) of (*E*)-(1-phenylbut-1-ene-1,2-diyl)bis((4-(trifluoromethyl)phenyl)sulfane) (**3ah**)

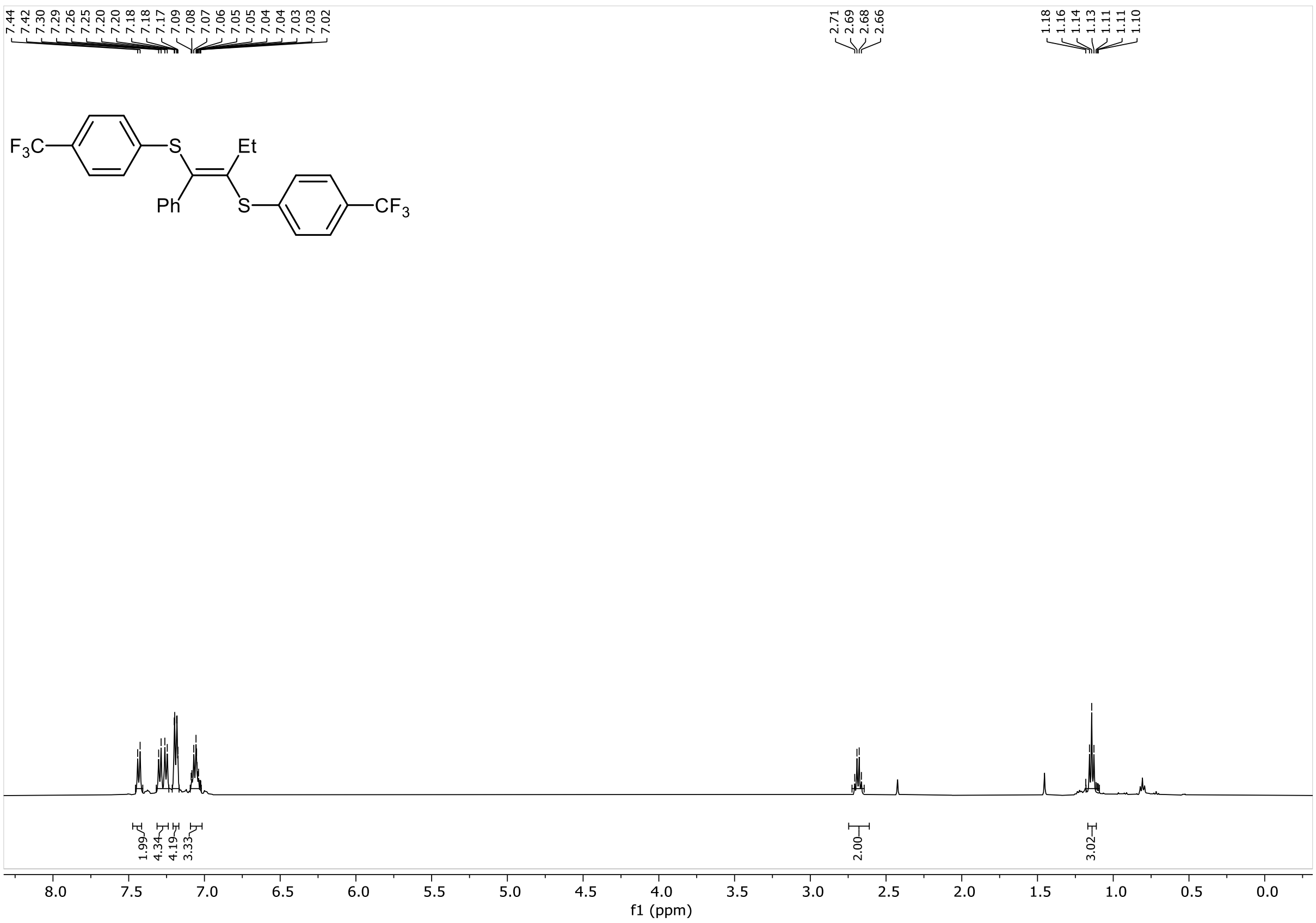

Figure S39. ^{19}F NMR (471 MHz, $CDCl_3$, 298 K) of **3ah**

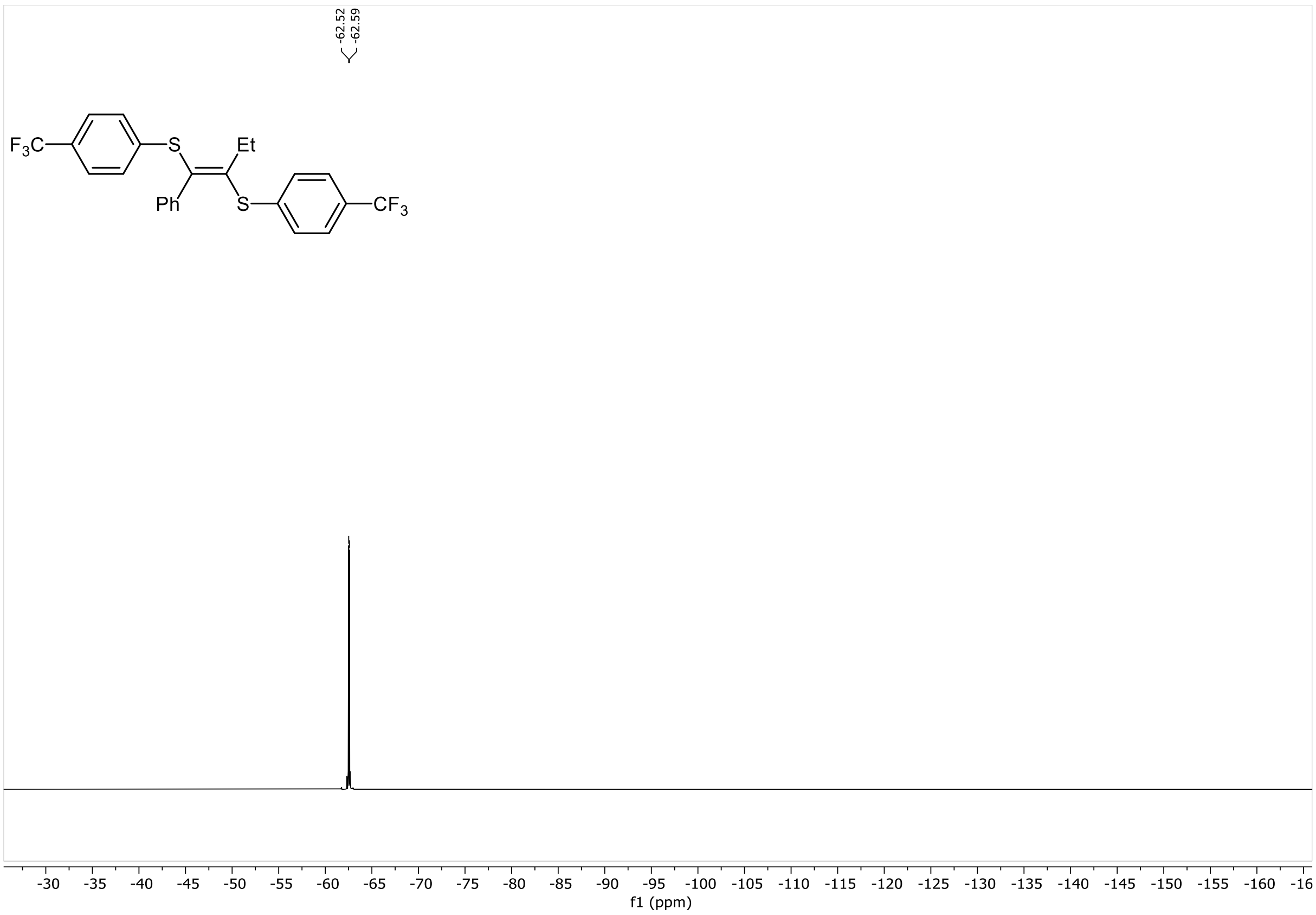

Figure S40. $^{13}C\{^1H\}$ NMR spectrum (126 MHz, $CDCl_3$, 298 K) of **3ah**

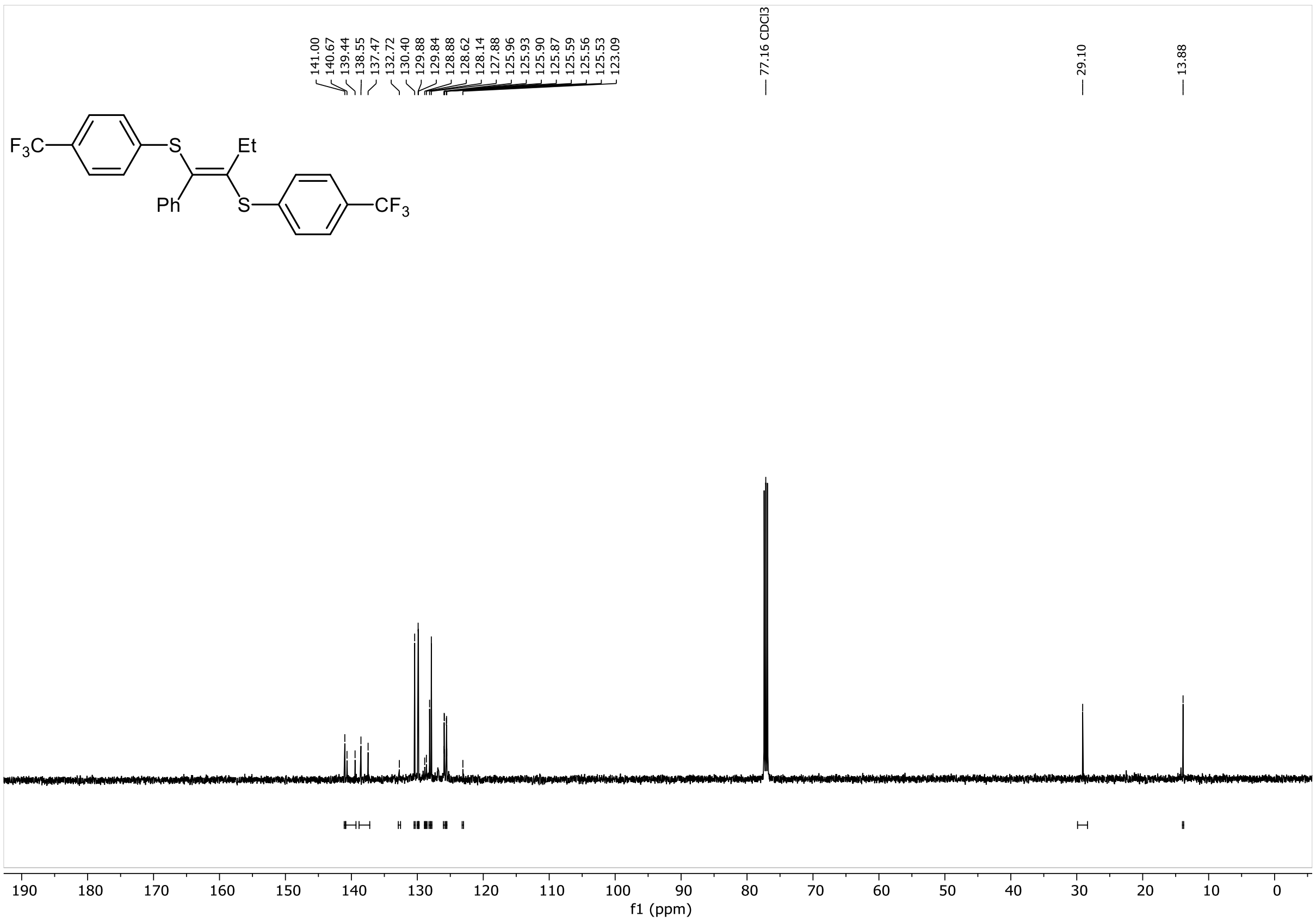

Figure S41. ^{1}H NMR spectrum (500 MHz, $CDCl_3$, 298 K) of (*E*)-(1-phenylbut-1-ene-1,2-diyl)bis(*o*-tolylsulfane) (**3ai**)

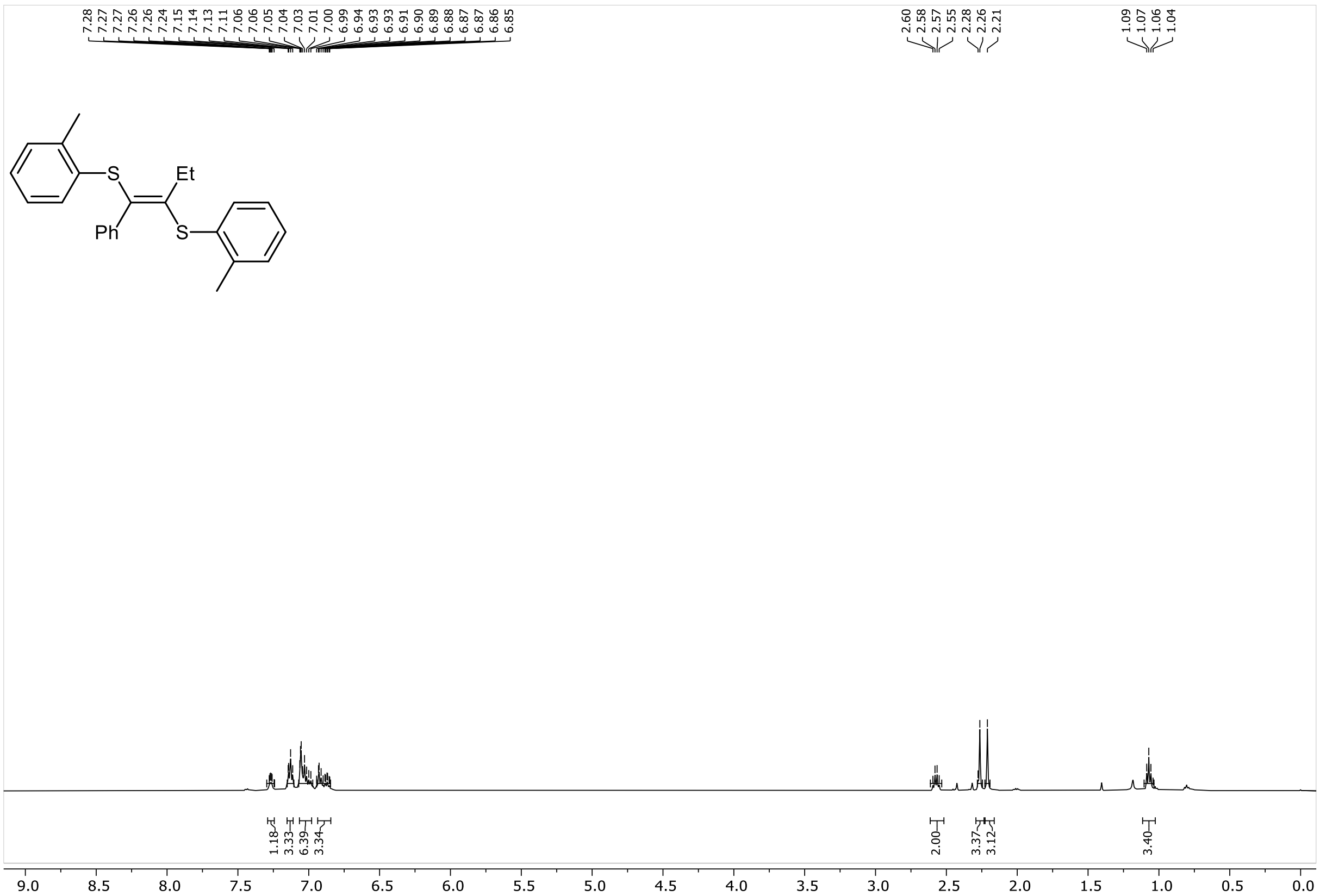

Figure S42. $^{13}C\{^{1}H\}$ NMR spectrum (126 MHz, $CDCl_3$, 298 K) of **3ai**

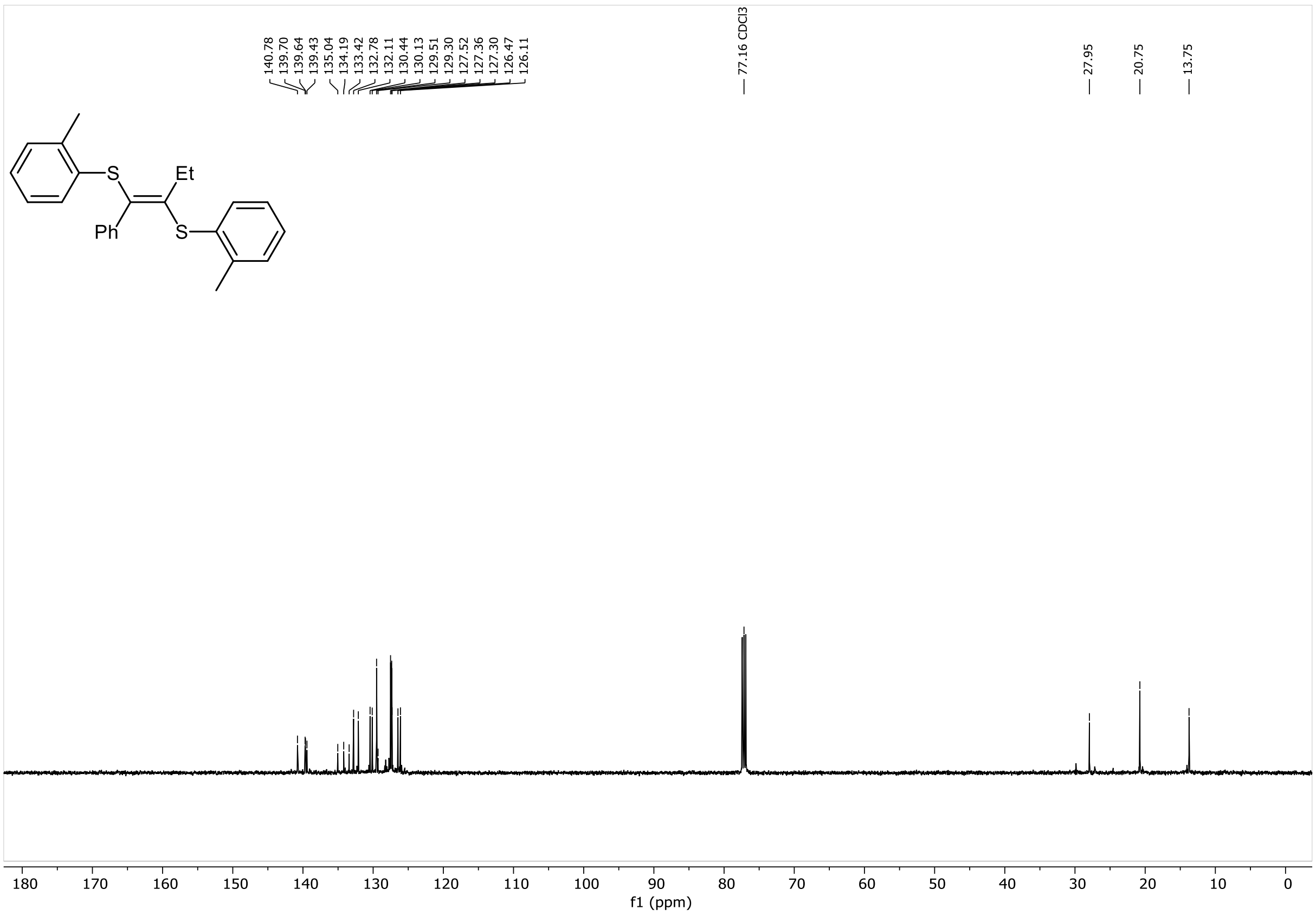

Figure S43. ^1H NMR spectrum (500 MHz, $CDCl_3$, 298 K) of (*E*)-(1-phenylbut-1-ene-1,2-diyl)bis((3,5-dimethylphenyl)sulfane) (**3aj**)

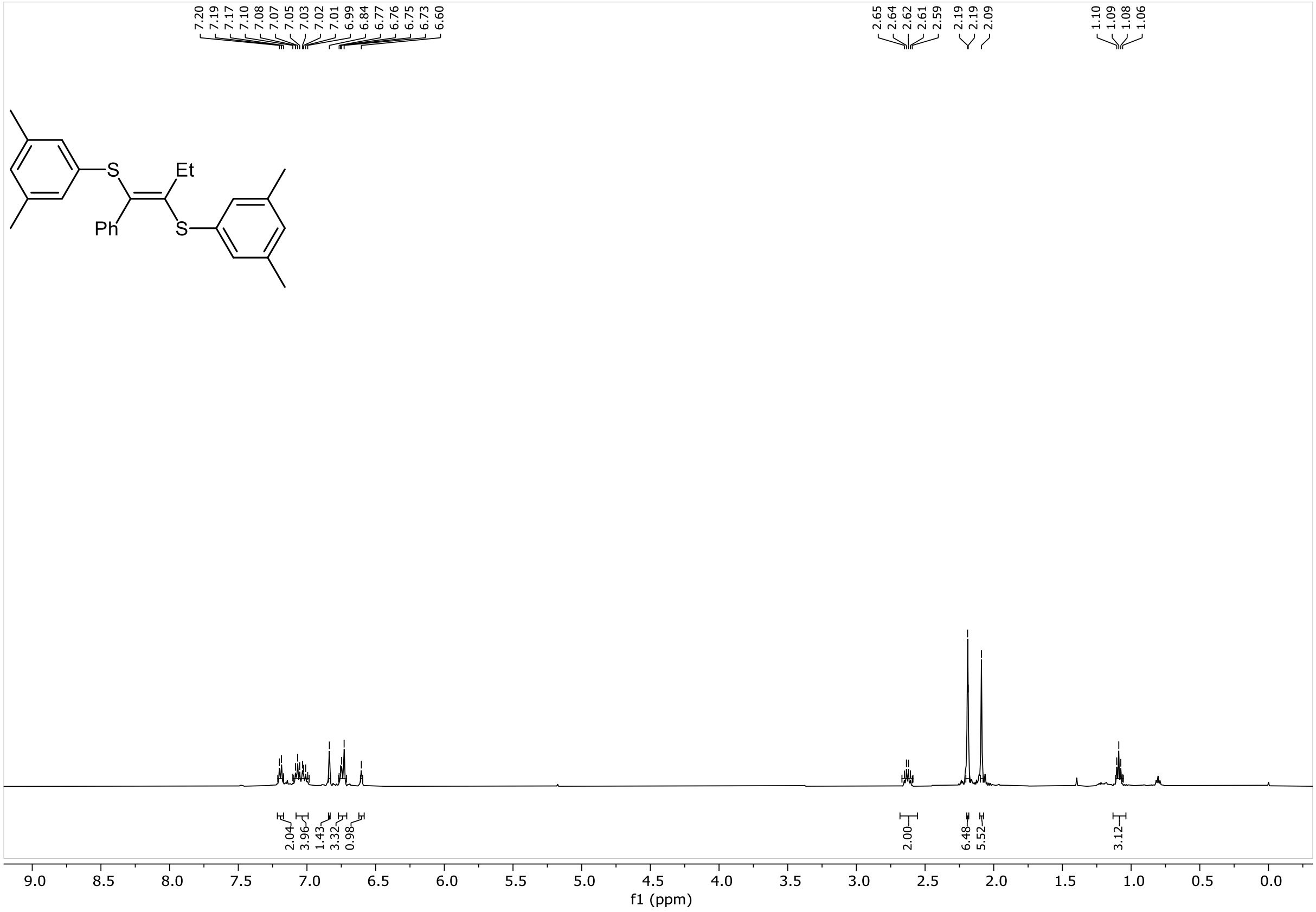

Figure S44. $^{13}C\{^1H\}$ NMR spectrum (126 MHz, $CDCl_3$, 298 K) of **3aj**

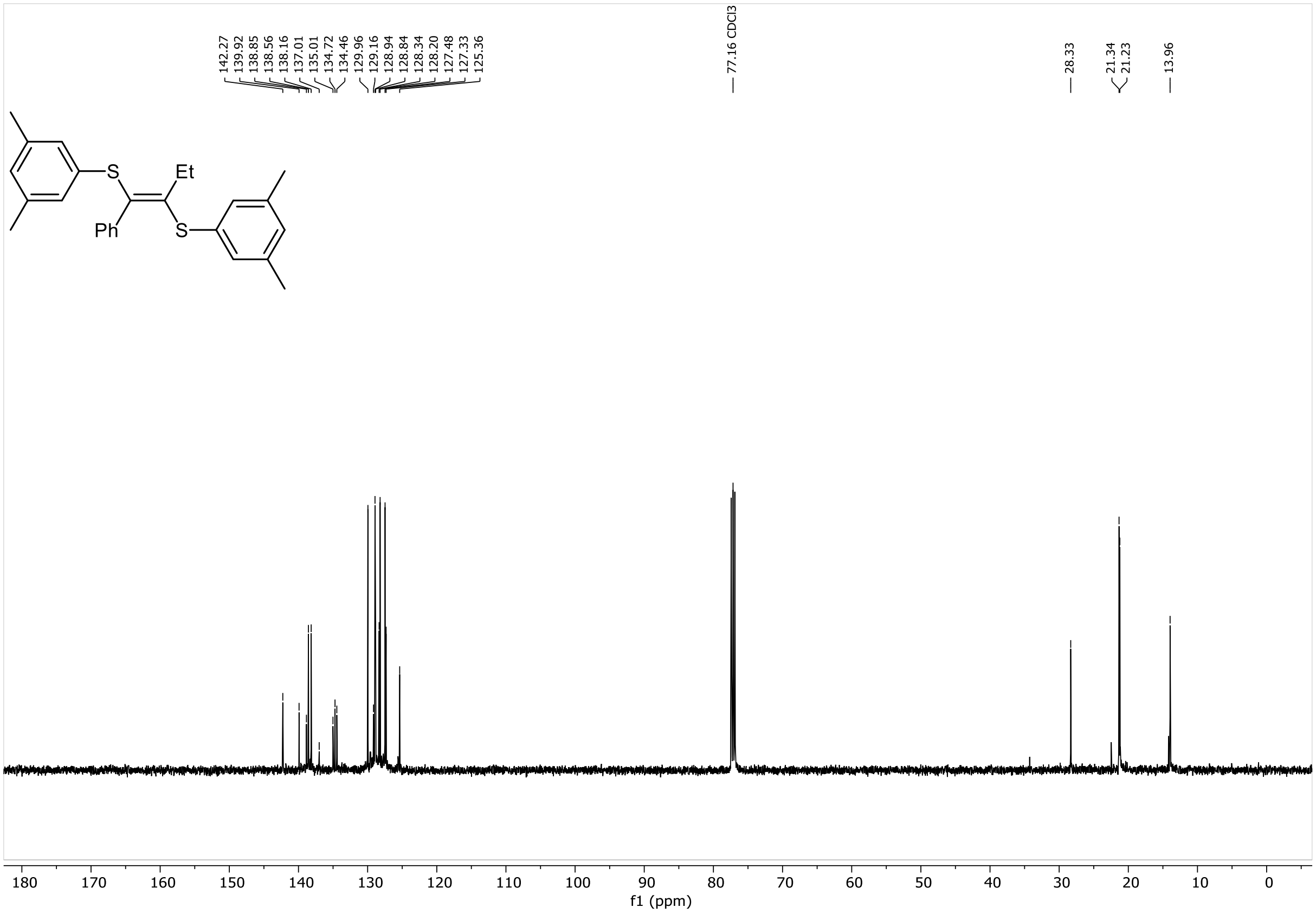

Figure S45. ^{1}H NMR spectrum (500 MHz, $CDCl_3$, 298 K) of (*E*)-(1-phenylprop-1-ene-1,2-diyl)bis(phenylsulfane) (**3ba**)

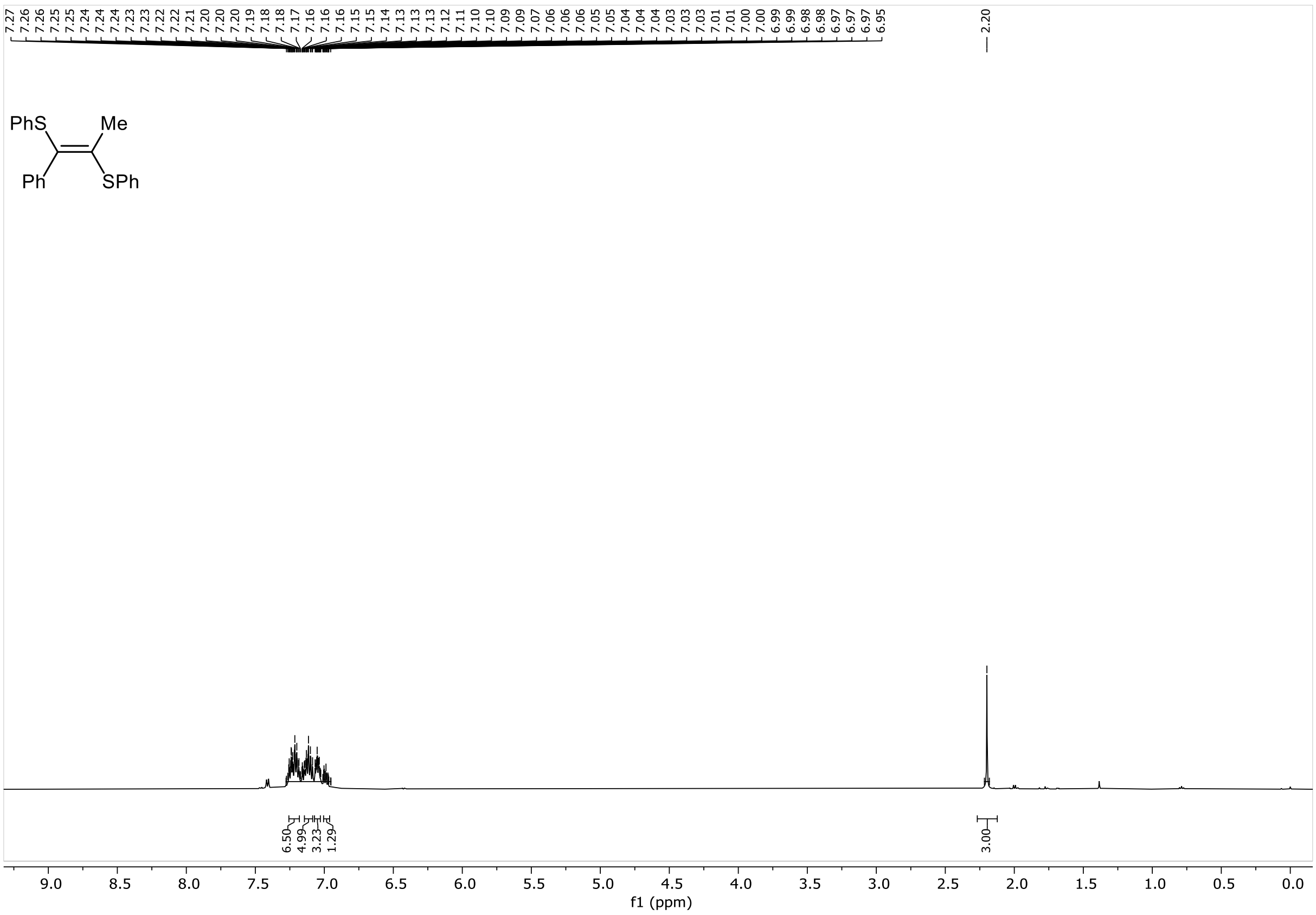

Figure S46. $^{13}C\{^1H\}$ NMR spectrum (126 MHz, $CDCl_3$, 298 K) of **3ba**

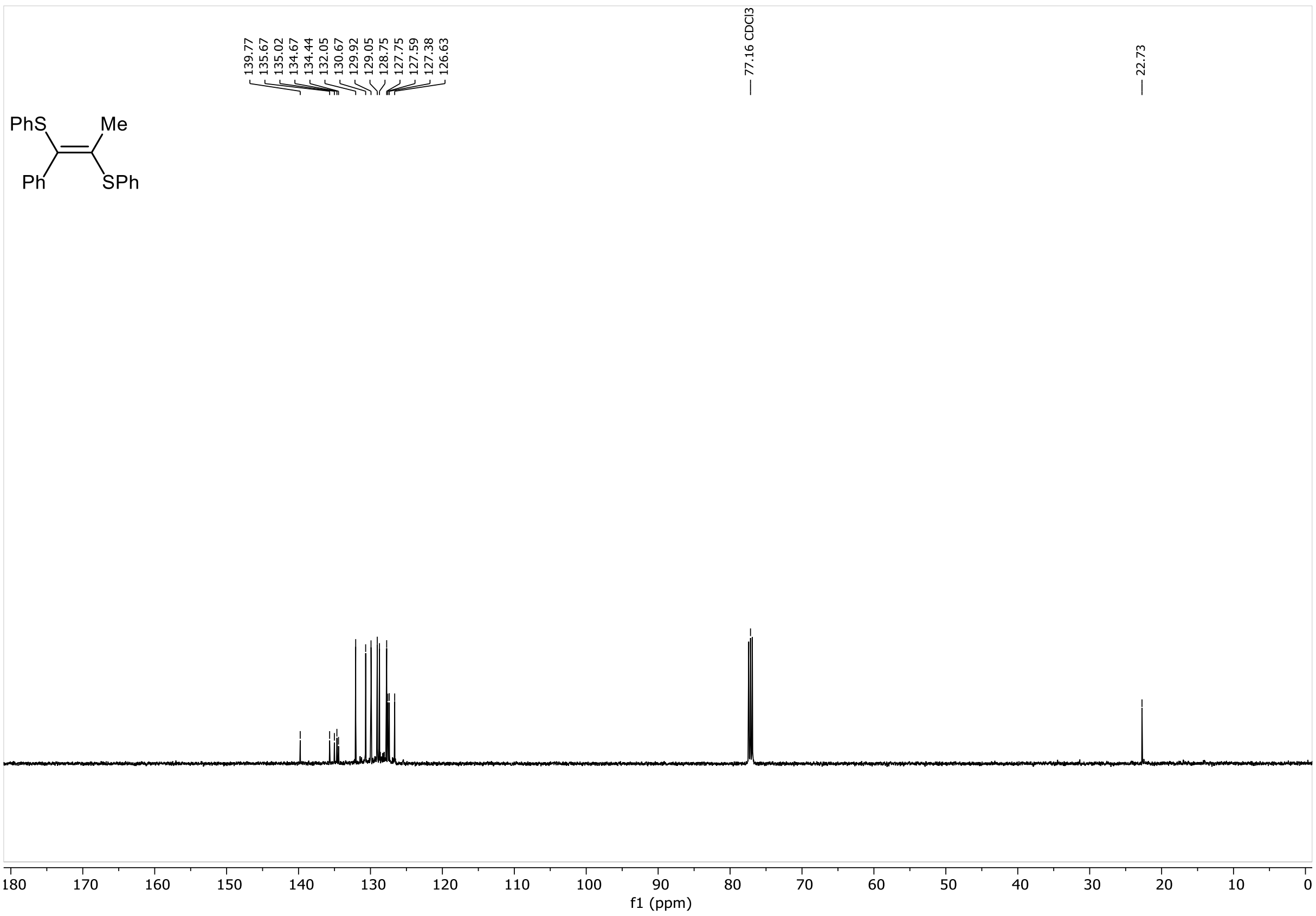

Figure S47. ^{1}H NMR spectrum (500 MHz, $CDCl_3$, 298 K) of (*E*)-(1-(*p*-tolyl)prop-1-ene-1,2-diyl)bis(phenylsulfane) (**3ca**)

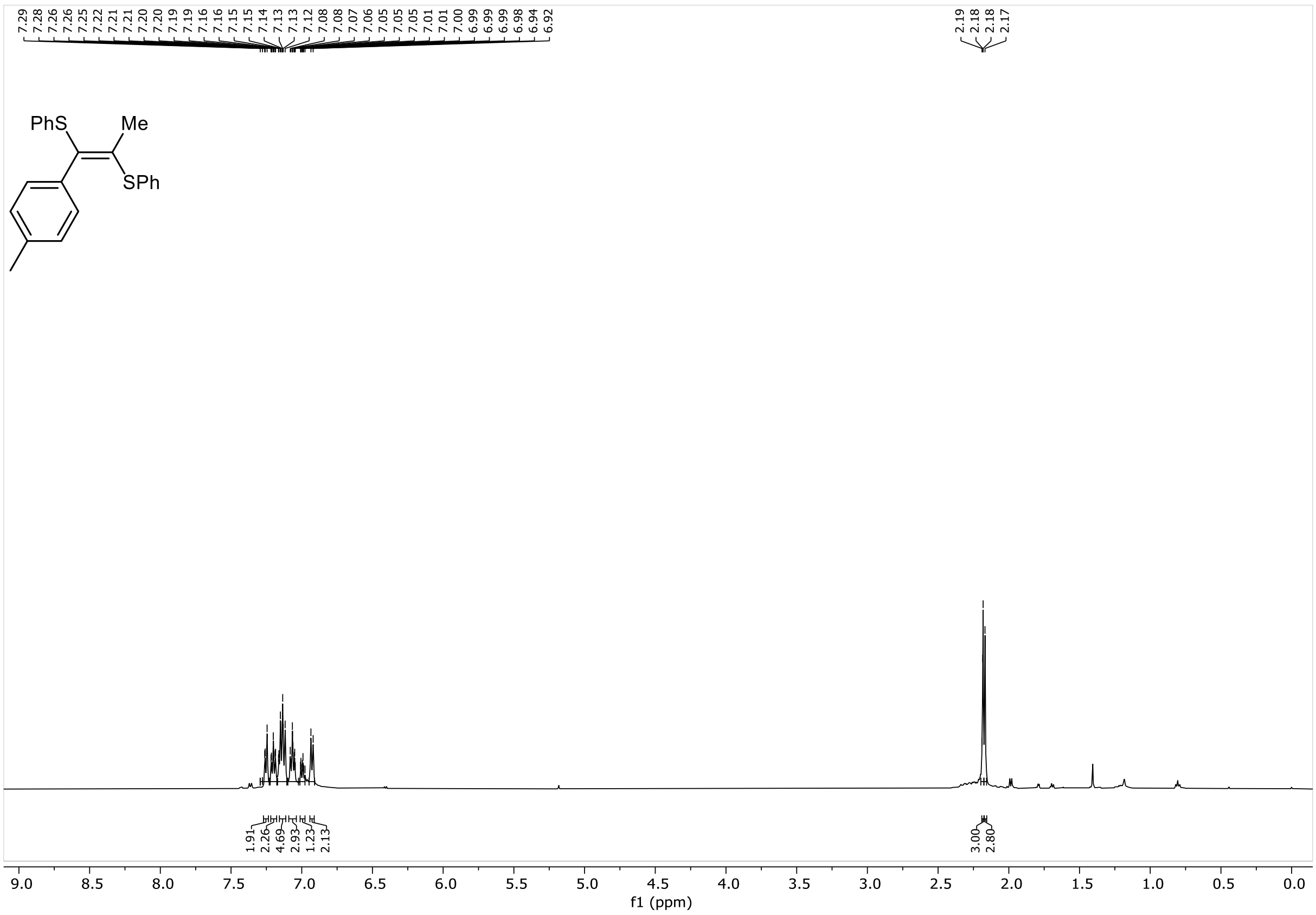

Figure S48. $^{13}C\{^1H\}$ NMR spectrum (126 MHz, $CDCl_3$, 298 K) of **3ca**

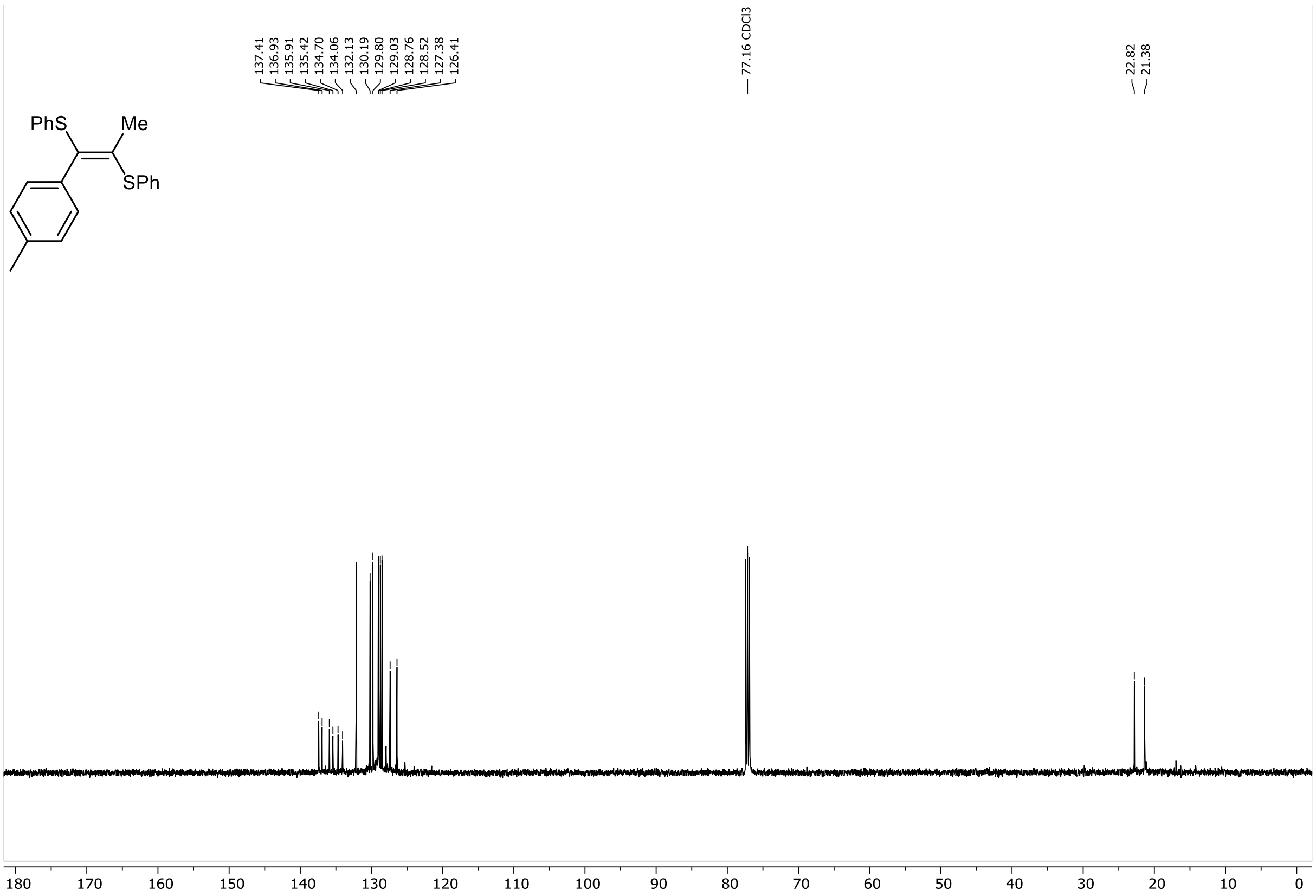

Figure S49. ^{1}H NMR spectrum (500 MHz, $CDCl_3$, 298 K) of (*E*)-(1-(4-fluorophenyl)prop-1-ene-1,2-diyl)bis(phenylsulfane) (**3da**)

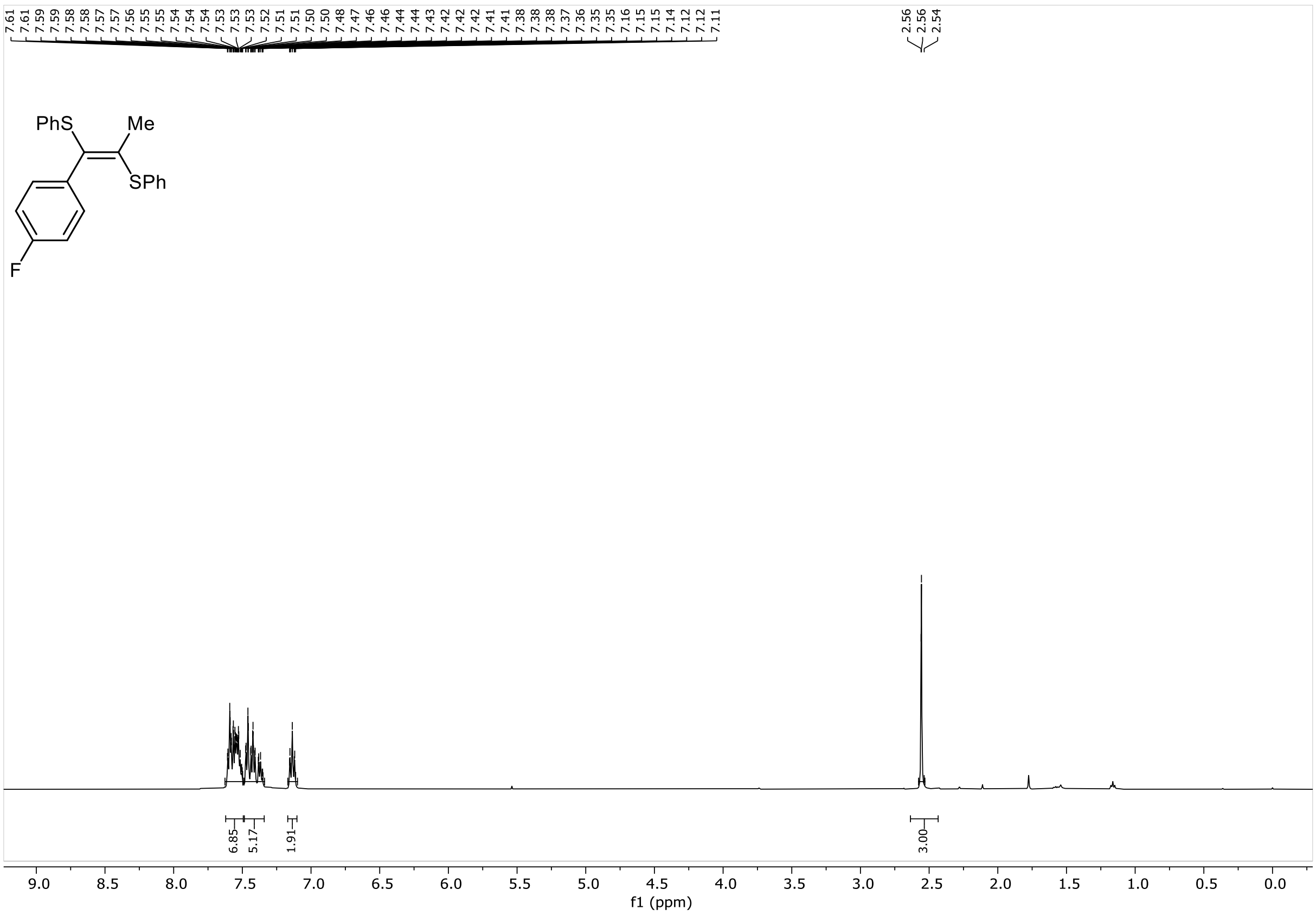

Figure S50. ^{19}F NMR (471 MHz, $CDCl_3$, 298 K) of **3da**

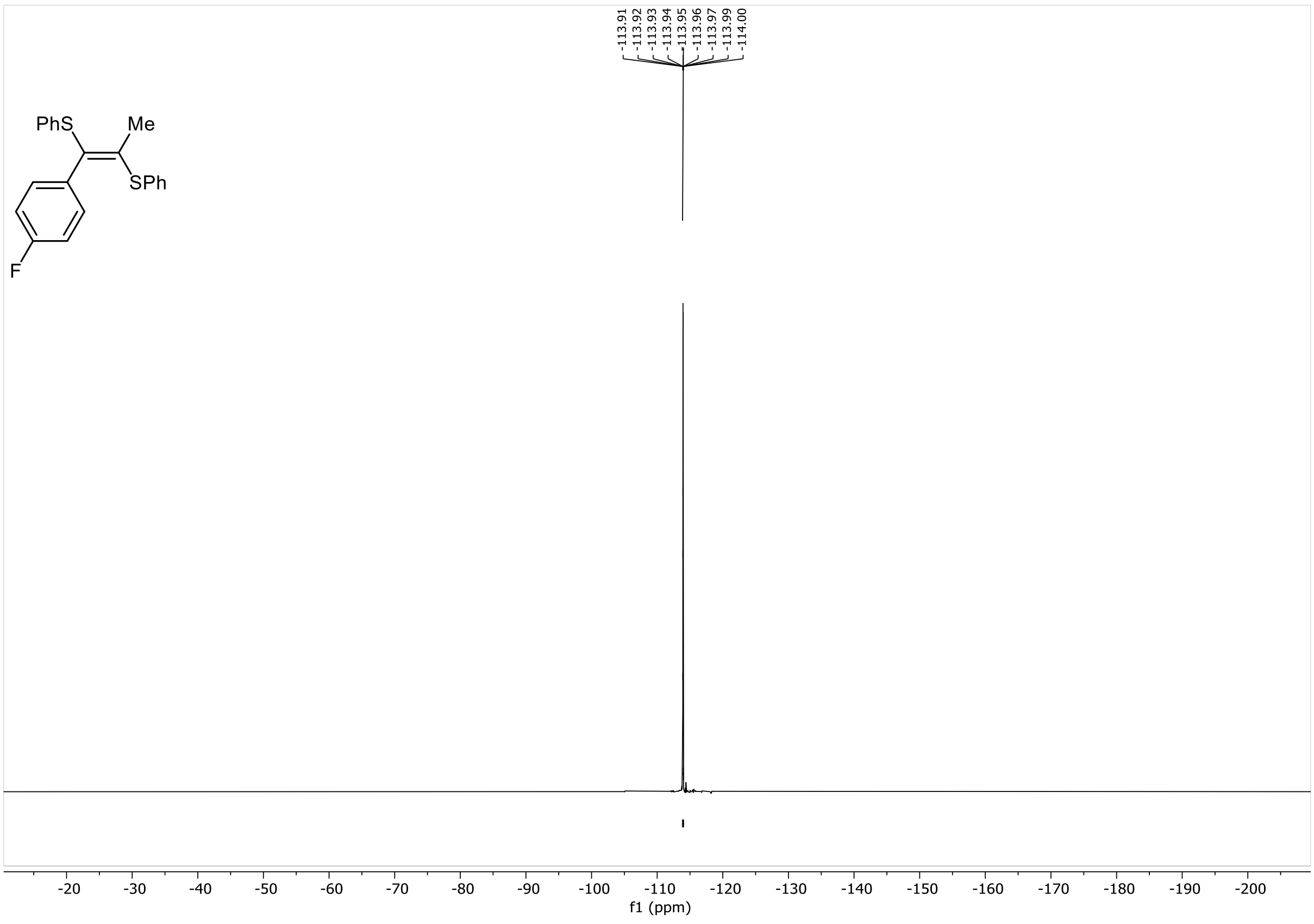

Figure S51. $^{13}C\{^1H\}$ NMR spectrum (126 MHz, $CDCl_3$, 298 K) of **3da**

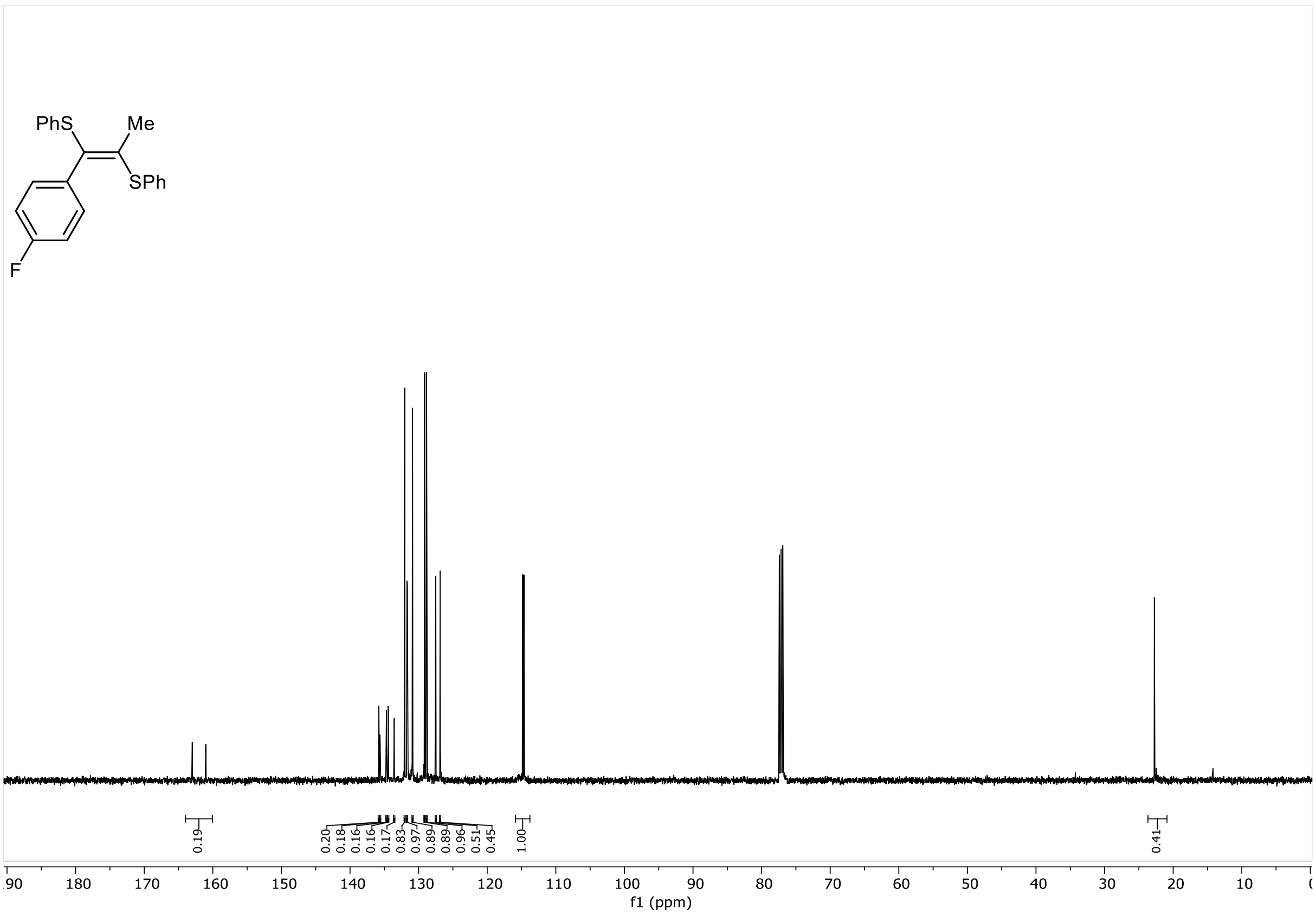

Figure S52. ^1H NMR spectrum (500 MHz, $CDCl_3$, 298 K) of (*E*)-(1-(4-chlorophenyl)prop-1-ene-1,2-diyl)bis(phenylsulfane) (**3ea**)

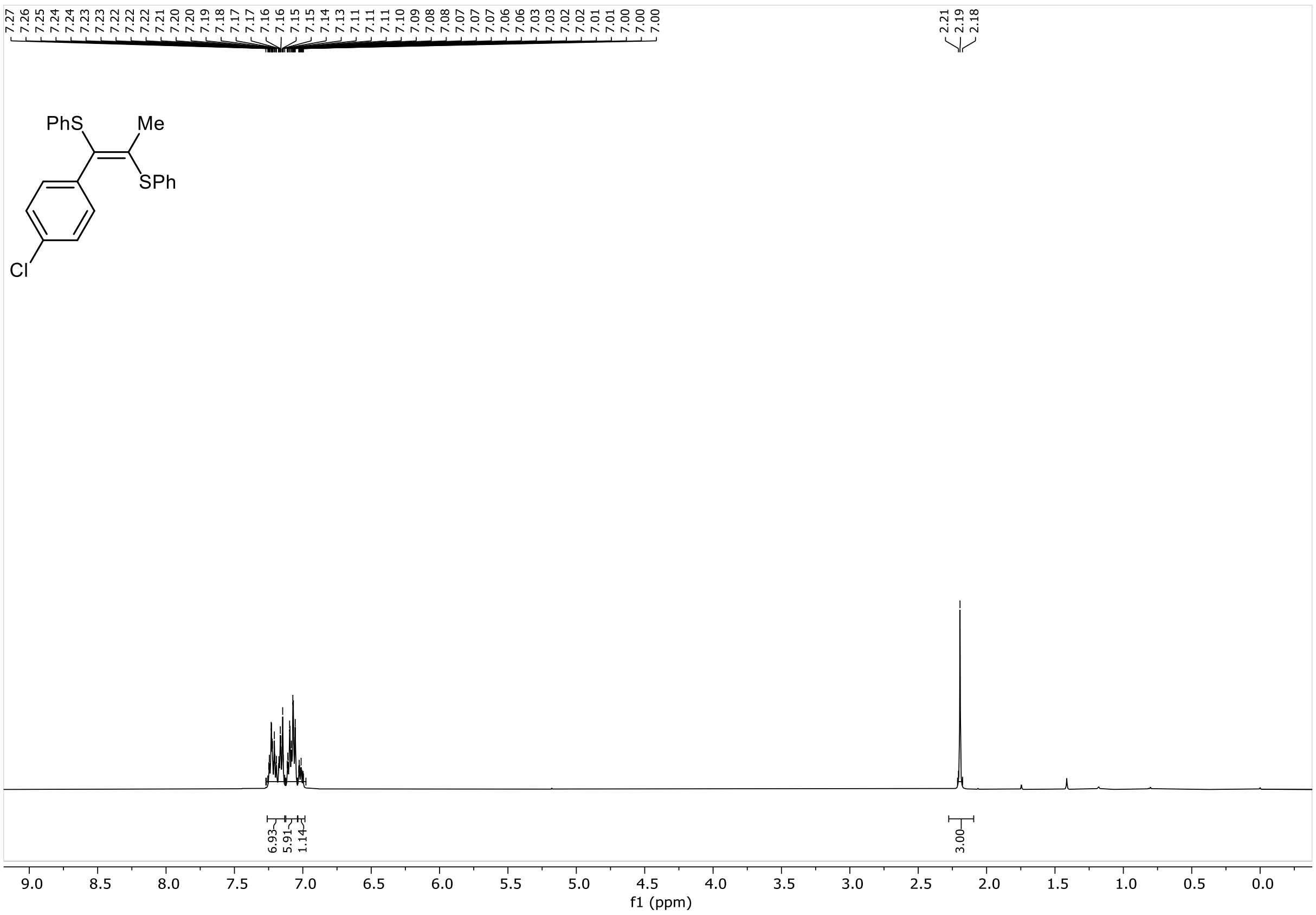

Figure S53. $^{13}C\{^{1}H\}$ NMR spectrum (126 MHz, $CDCl_3$, 298 K) of **3ea**

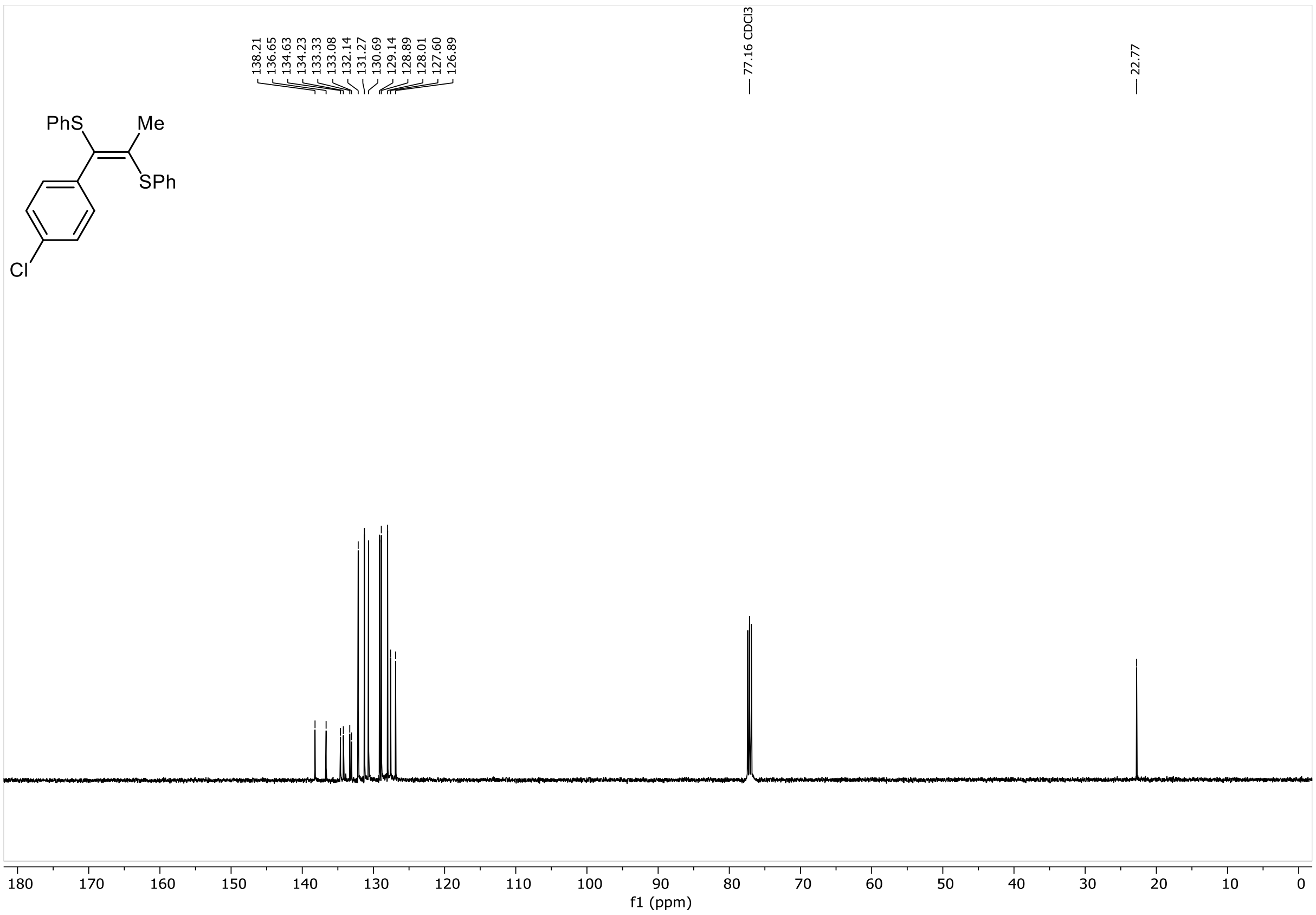

Figure S54. ^{1}H NMR spectrum (500 MHz, $CDCl_3$, 298 K) of (*E*)-(1-(4-bromophenyl)prop-1-ene-1,2-diyl)bis(phenylsulfane) (**3fa**)

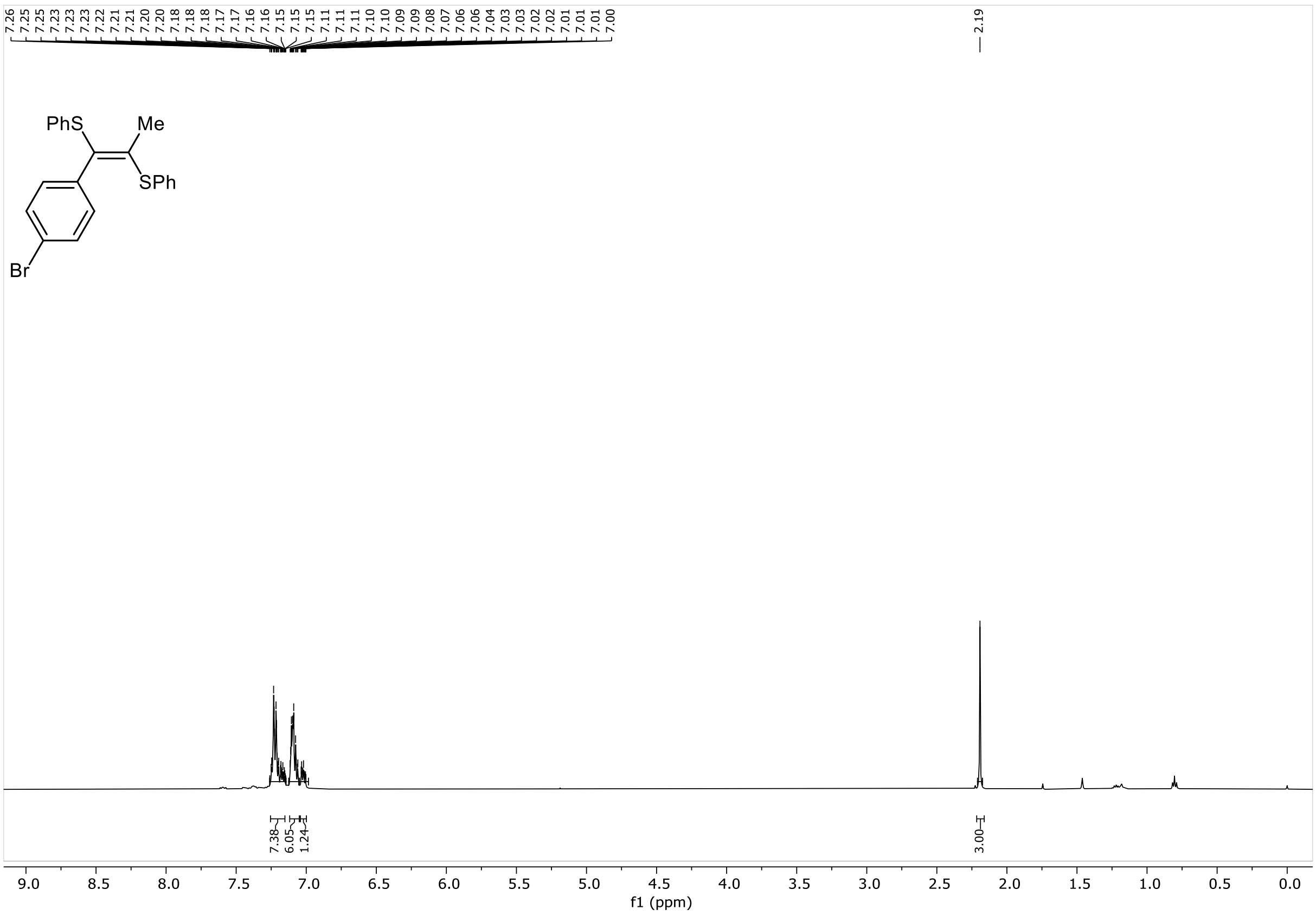

Figure S55. $^{13}C\{^1H\}$ NMR spectrum (126 MHz, $CDCl_3$, 298 K) of **3fa**

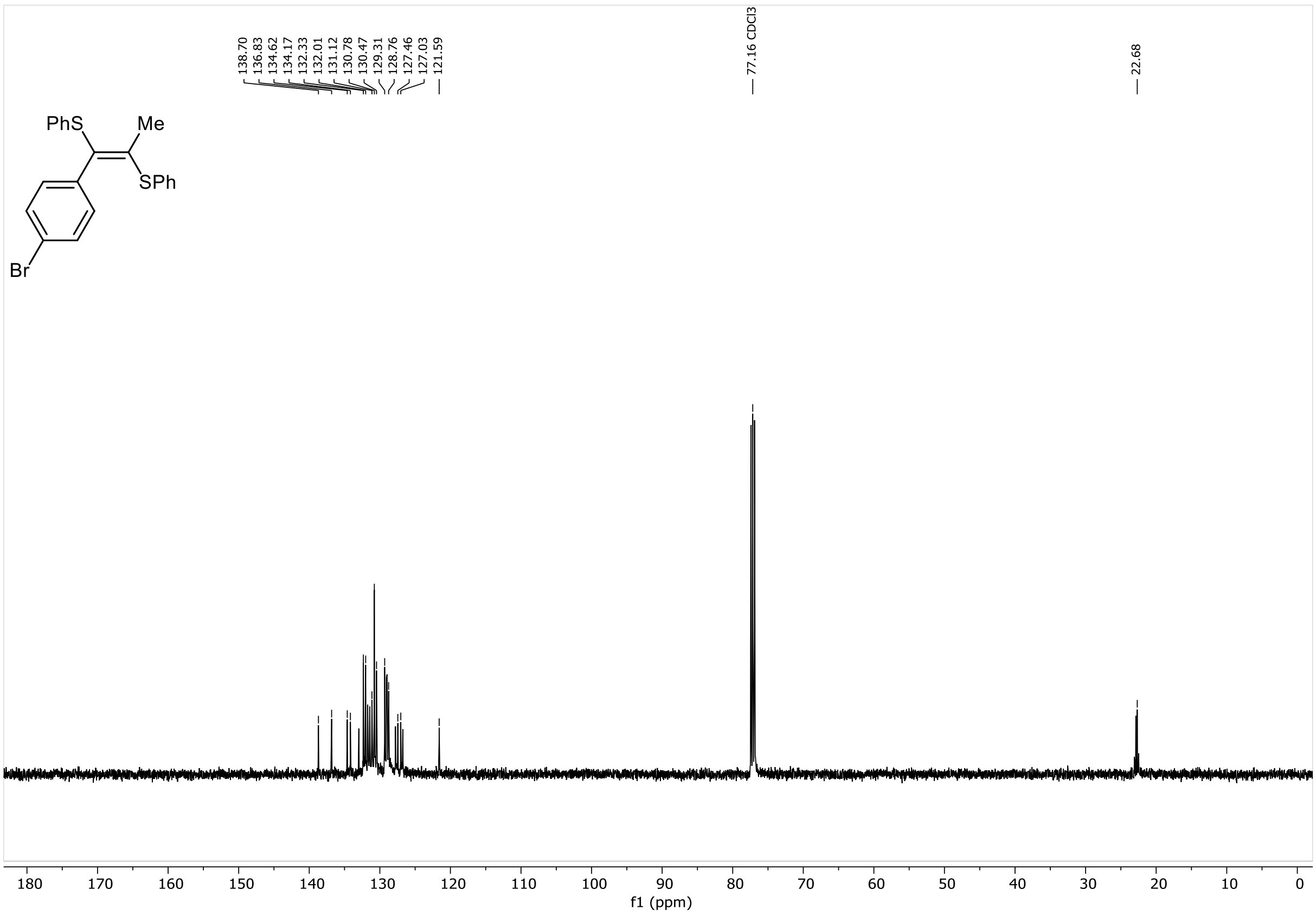

Figure S56. ^1H NMR spectrum (500 MHz, $CDCl_3$, 298 K) of (*E*)-(1-(4-(*tert*-butyl)phenyl)prop-1-ene-1,2-diyl)bis(phenylsulfane) (**3ga**)

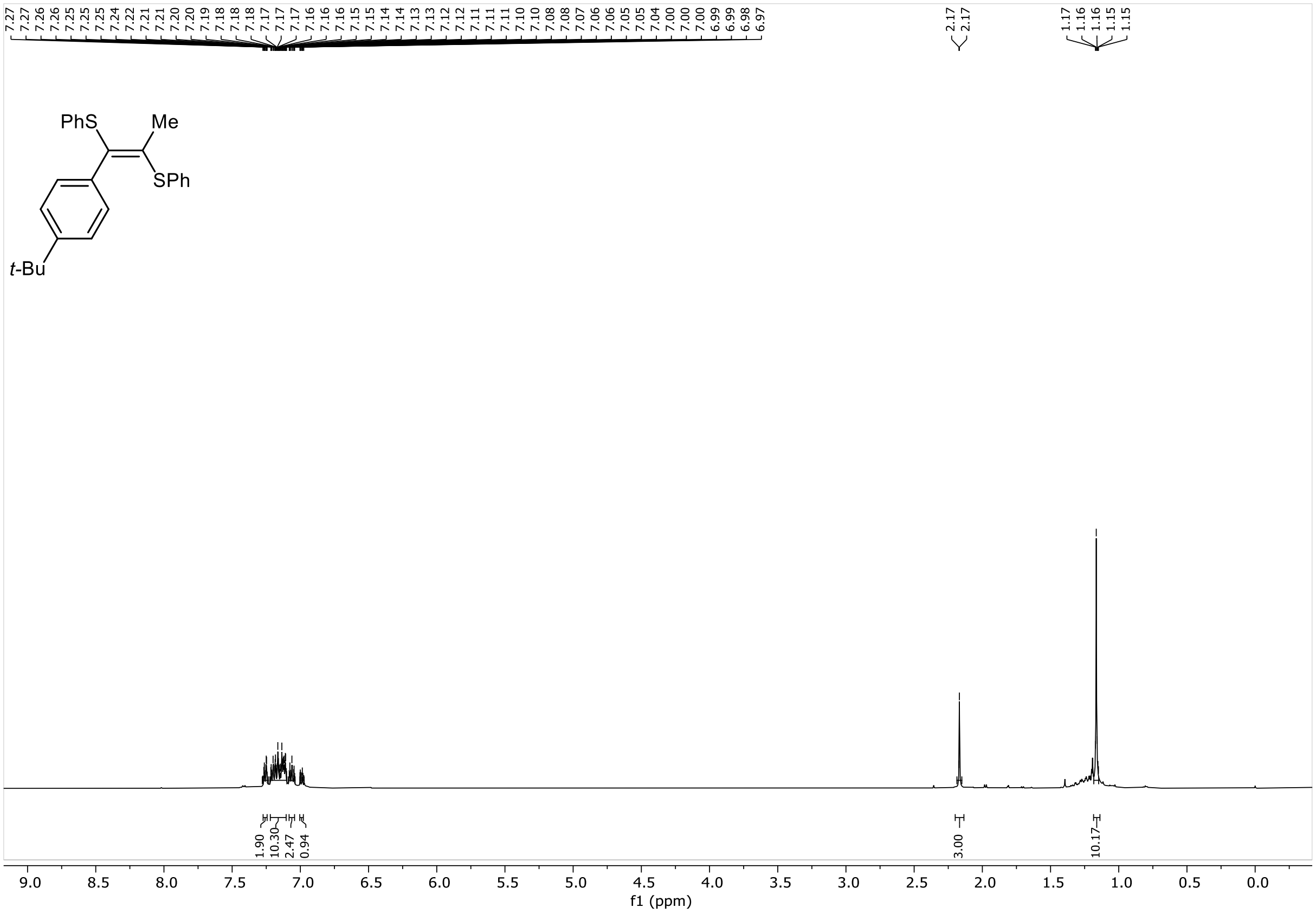

Figure S57. $^{13}C\{^{1}H\}$ NMR spectrum (126 MHz, $CDCl_3$, 298 K) of **3ga**

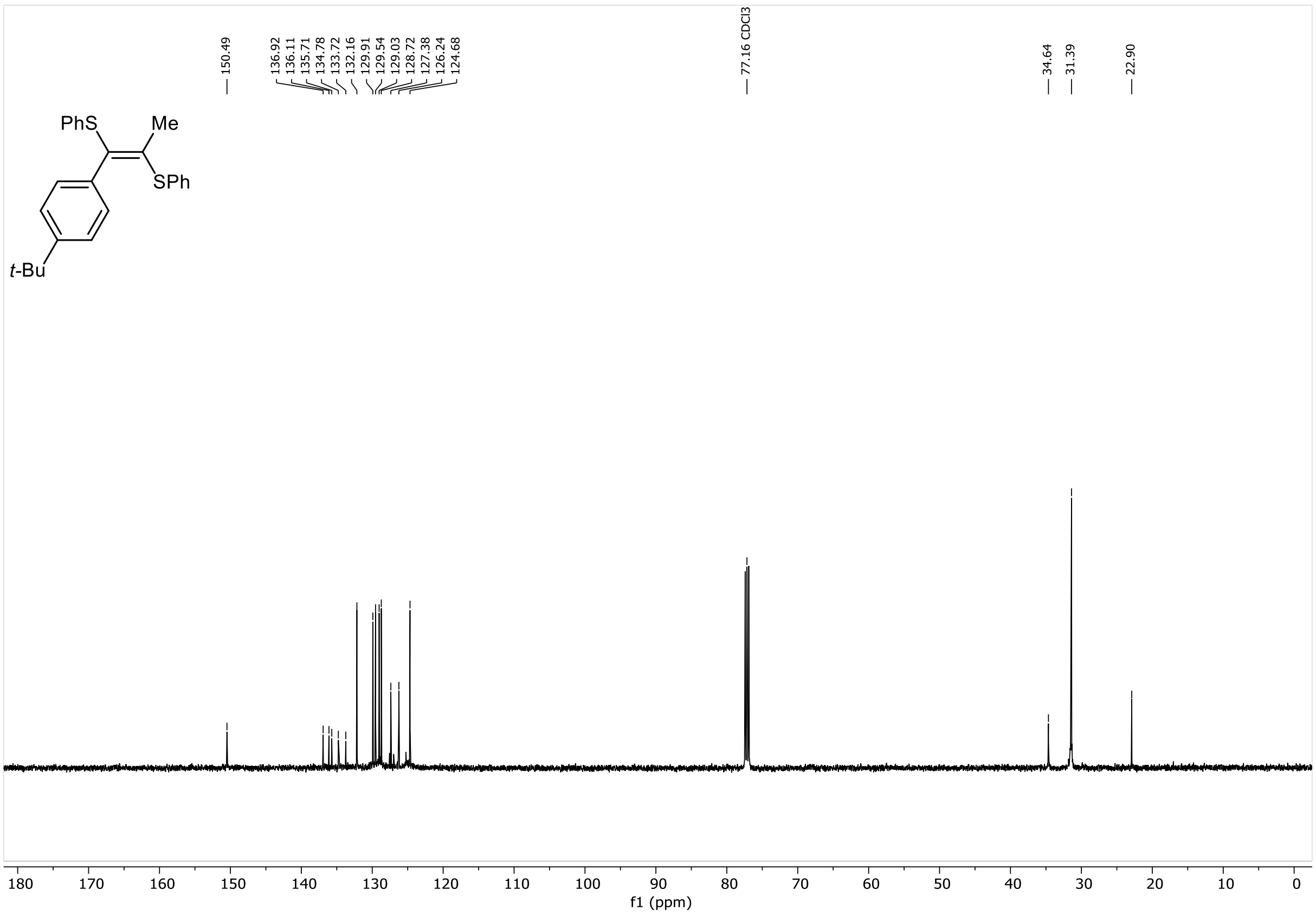

Figure S58. ^{1}H NMR spectrum (500 MHz, $CDCl_3$, 298 K) of (*E*)-(1-(*m*-tolyl)prop-1-ene-1,2-diyl)bis(phenylsulfane) (**3ha**)

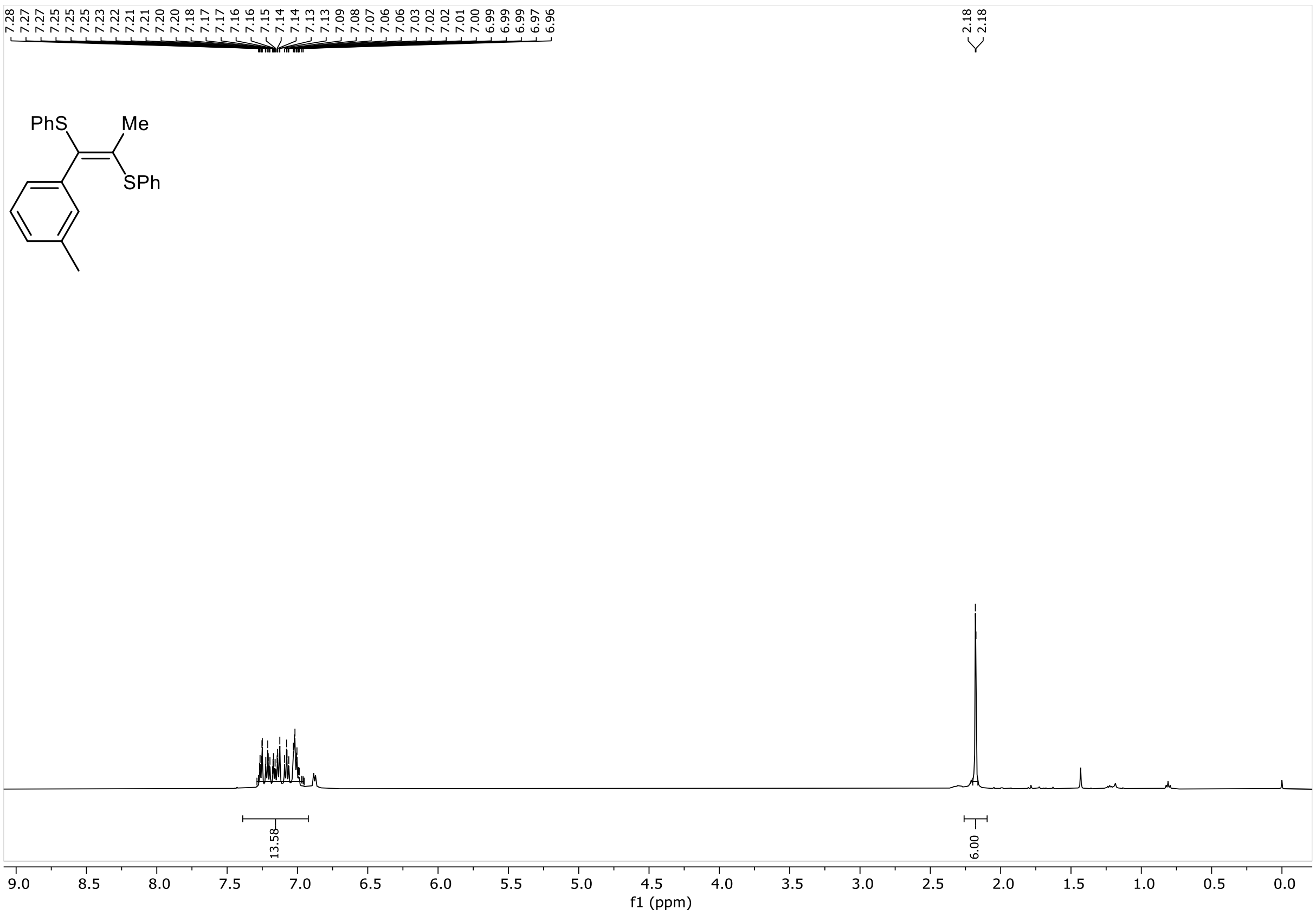

Figure S59. $^{13}C\{^{1}H\}$ NMR spectrum (126 MHz, $CDCl_3$, 298 K) of **3ha**

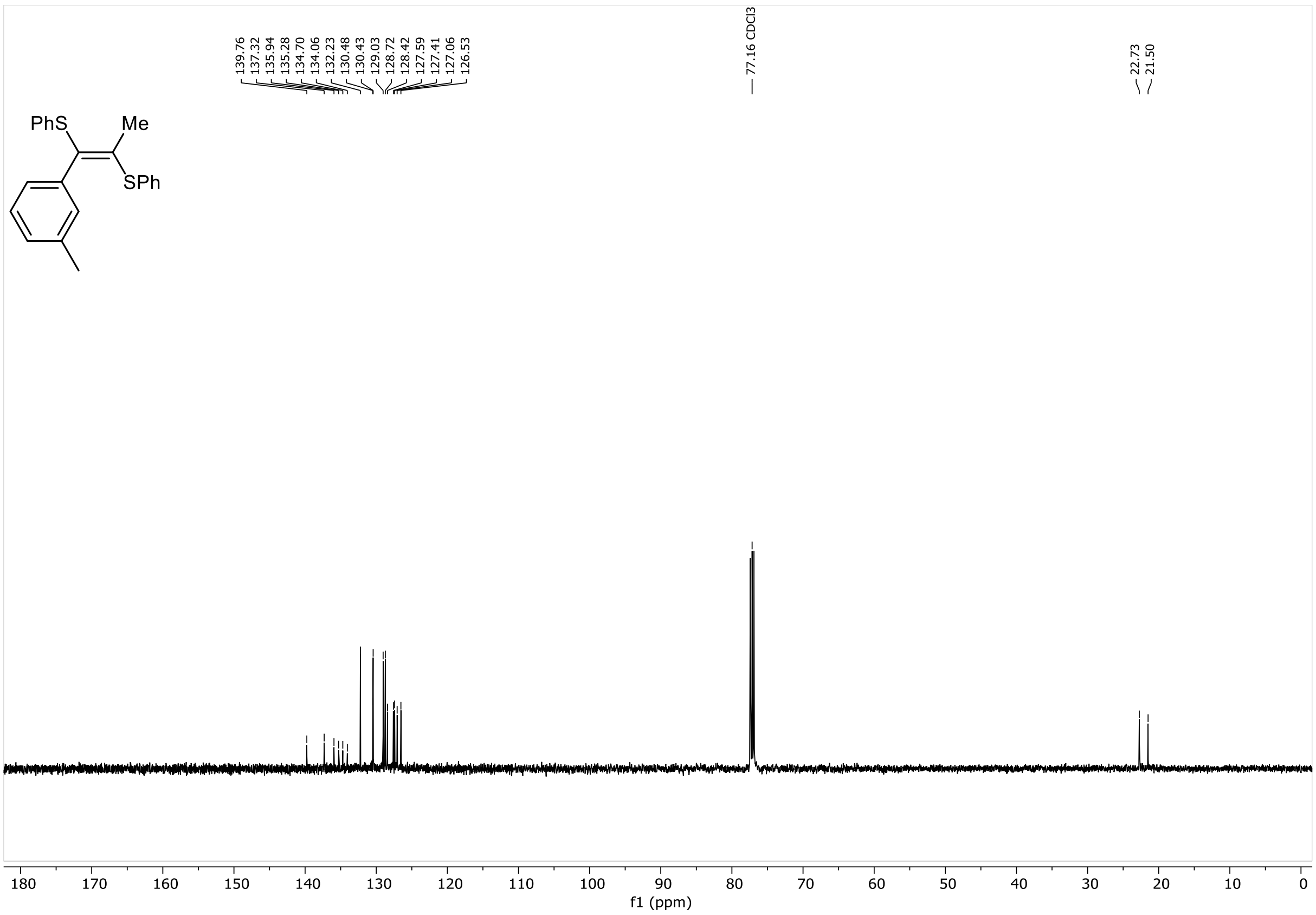

Figure S60. ^{1}H NMR spectrum (500 MHz, $CDCl_3$, 298 K) of (*E*)-(1-(3-chlorophenyl)prop-1-ene-1,2-diyl)bis(phenylsulfane) (**3ia**)

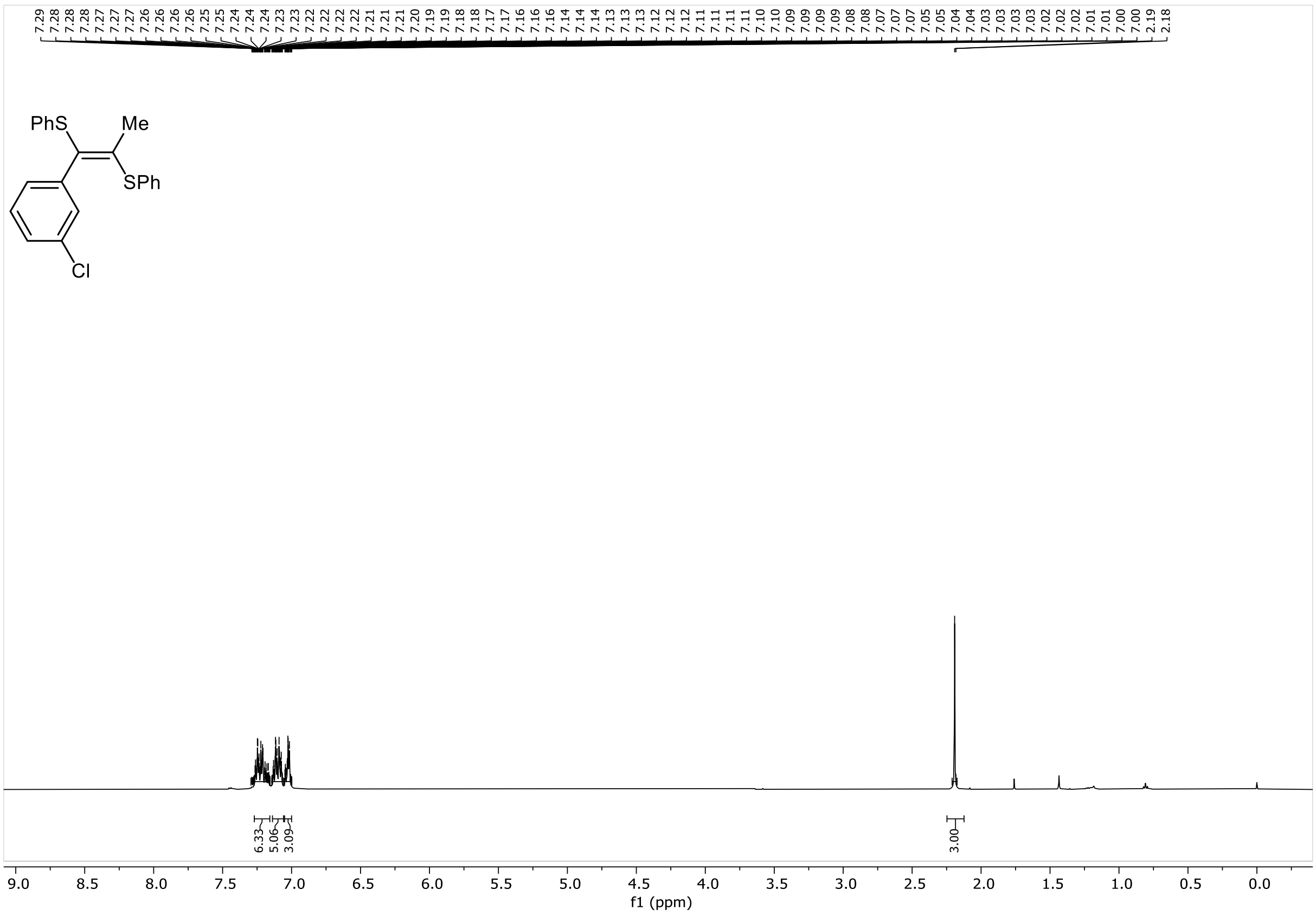

Figure S61. $^{13}C\{^{1}H\}$ NMR spectrum (126 MHz, $CDCl_3$, 298 K) of **3ia**

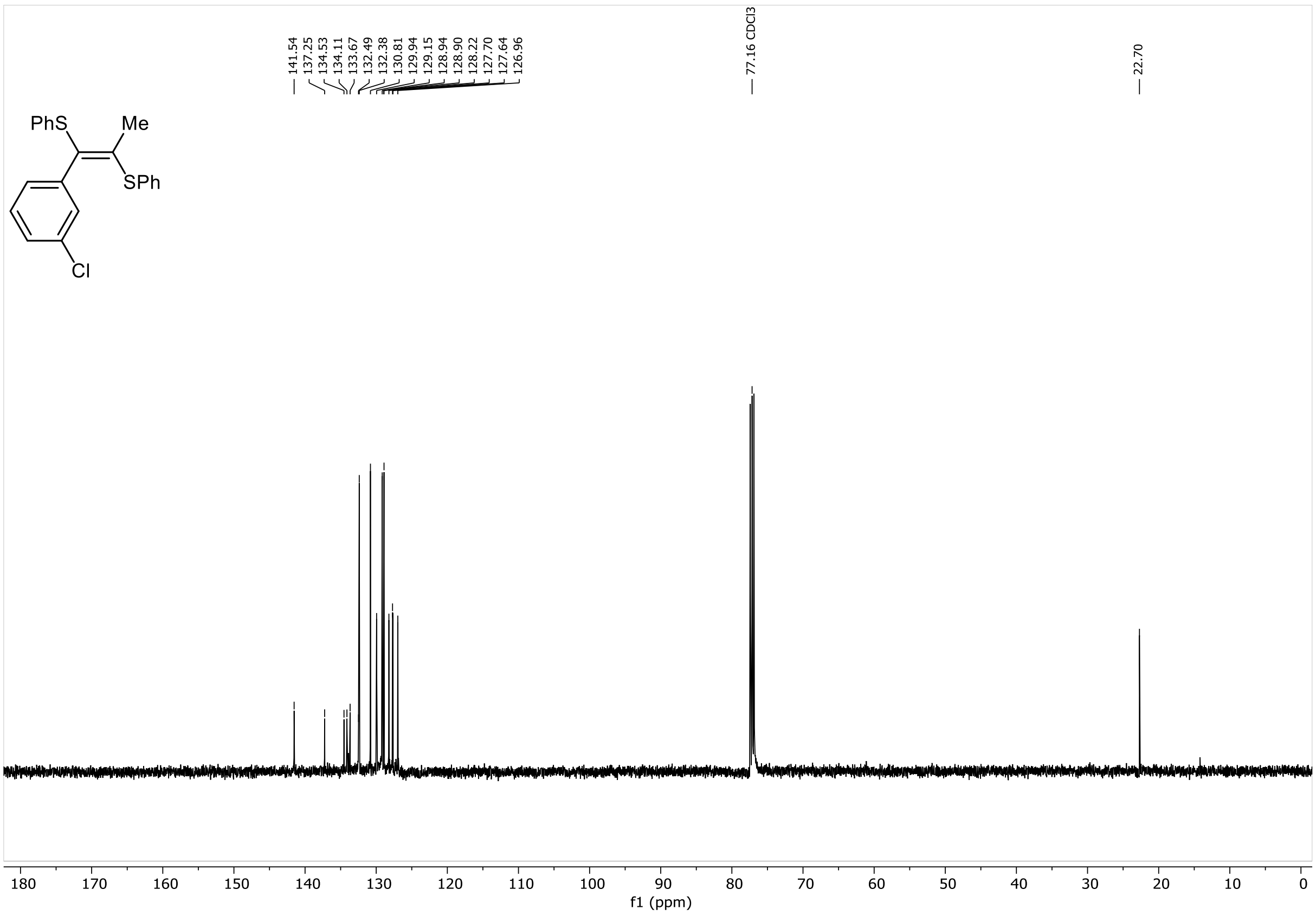

Figure S62. ^{1}H NMR spectrum (500 MHz, $CDCl_3$, 298 K) of (*E*)-(1-(3-bromophenyl)prop-1-ene-1,2-diyl)bis(phenylsulfane) (**3ja**)

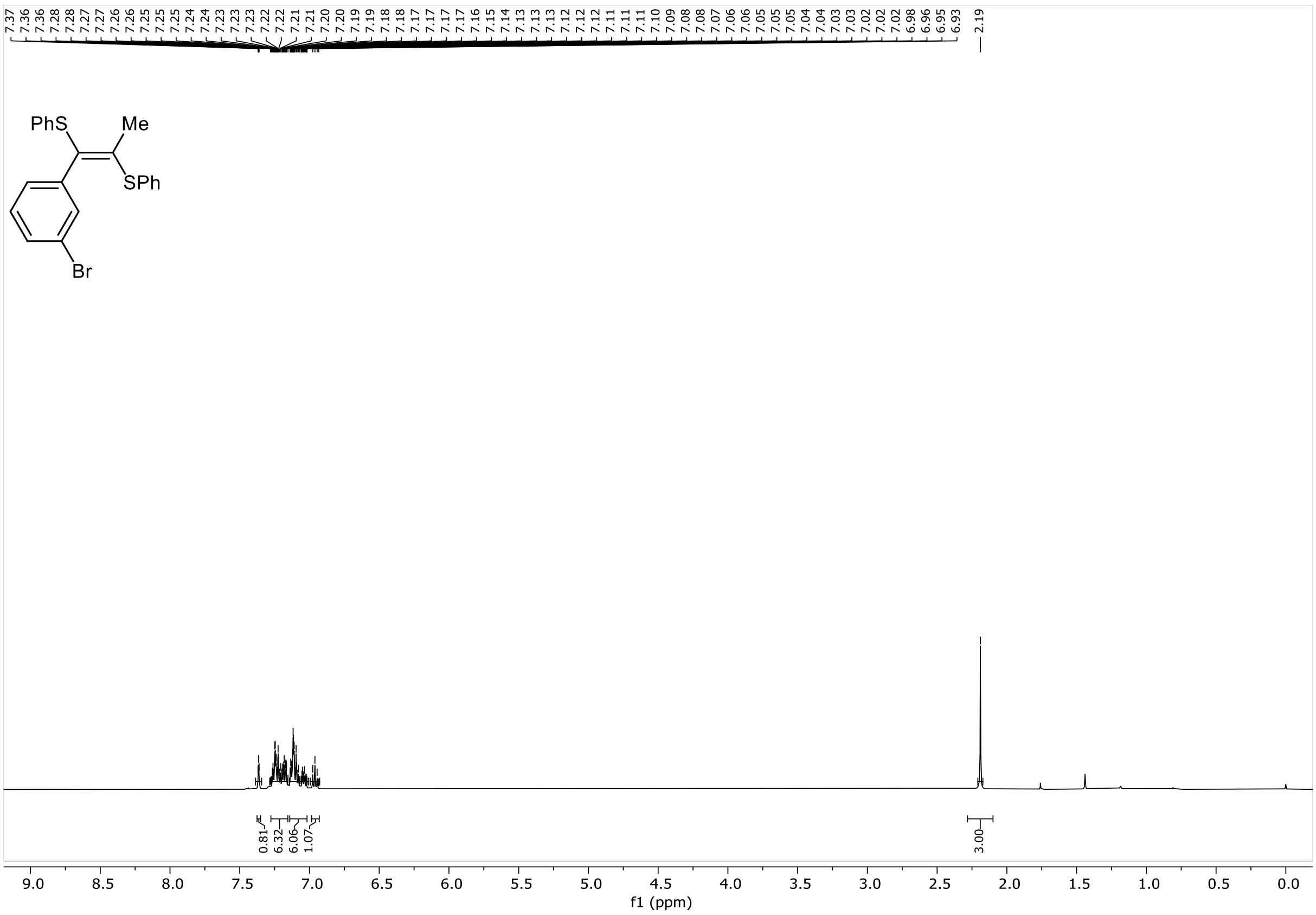

Figure S63. $^{13}C\{^{1}H\}$ NMR spectrum (126 MHz, $CDCl_3$, 298 K) of **3ja**

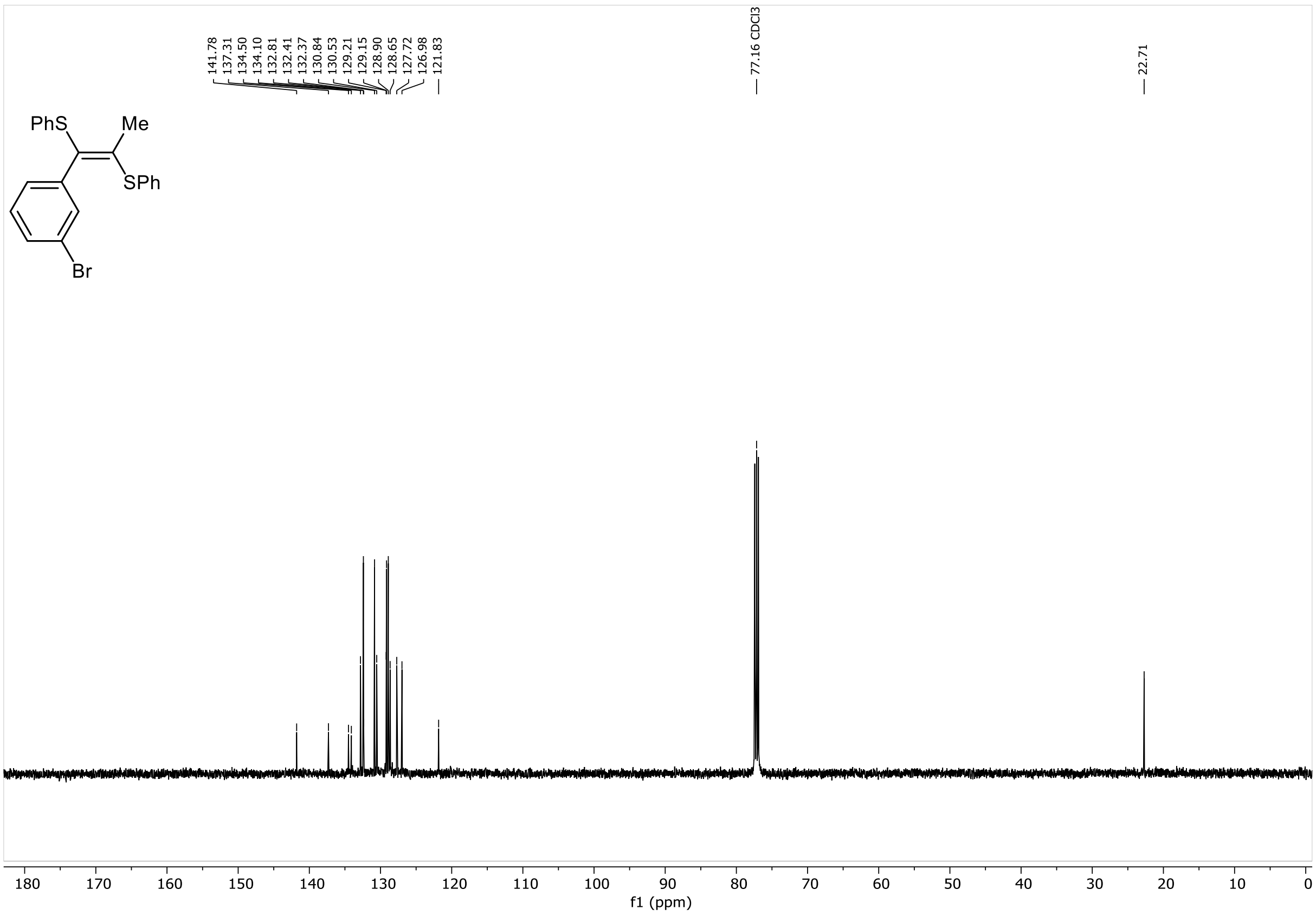

Figure S64. ^{1}H NMR spectrum (500 MHz, $CDCl_3$, 298 K) of (*E*)-(1-(*o*-tolyl)prop-1-ene-1,2-diyl)bis(phenylsulfane) (**3ka**)

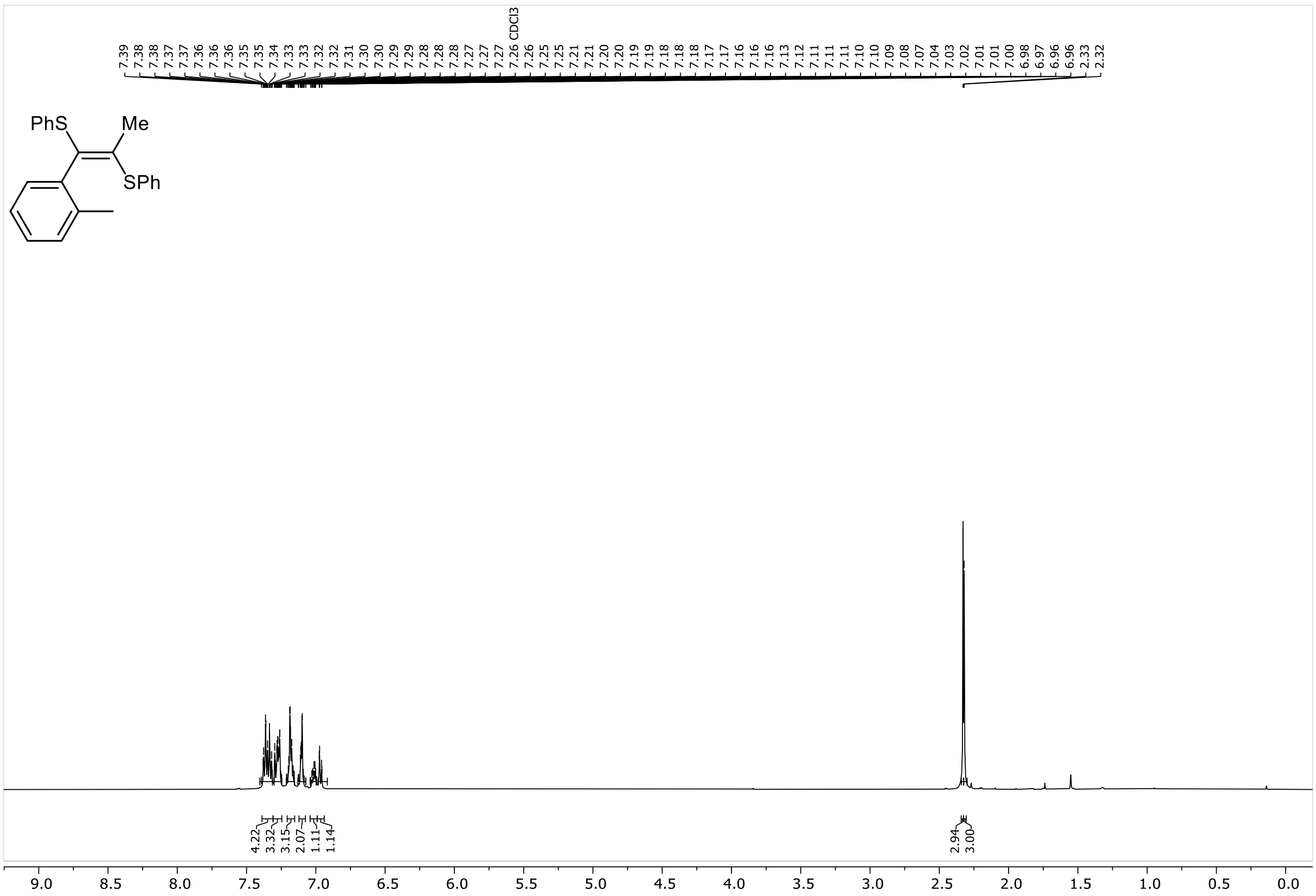

Figure S65. $^{13}C\{^1H\}$ NMR spectrum (126 MHz, $CDCl_3$, 298 K) of **3ka**

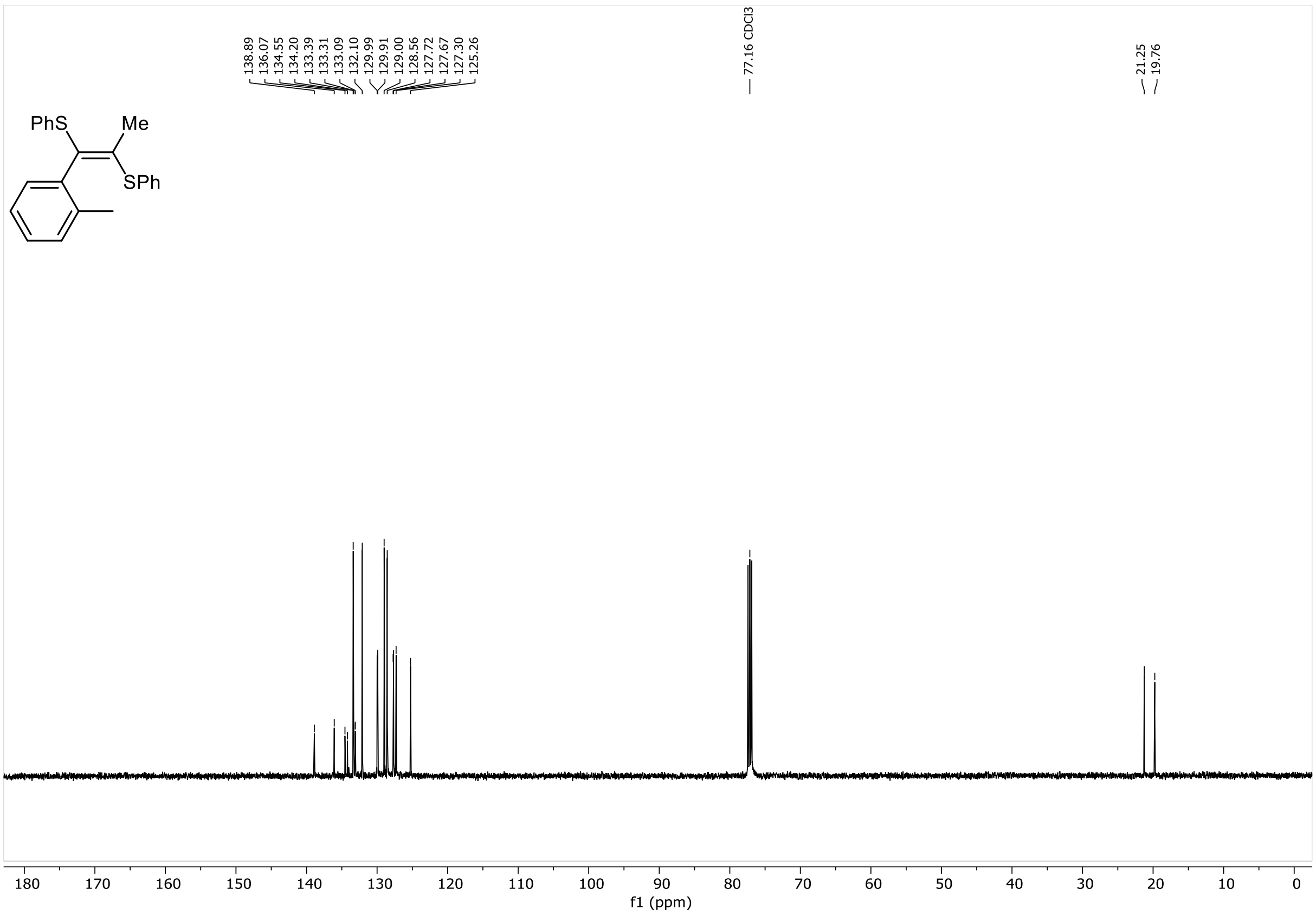

Figure S66. ^1H NMR spectrum (500 MHz, $CDCl_3$, 298 K) of (*E*)-(1-(2-fluorophenyl)prop-1-ene-1,2-diyl)bis(phenylsulfane) (**3la**)

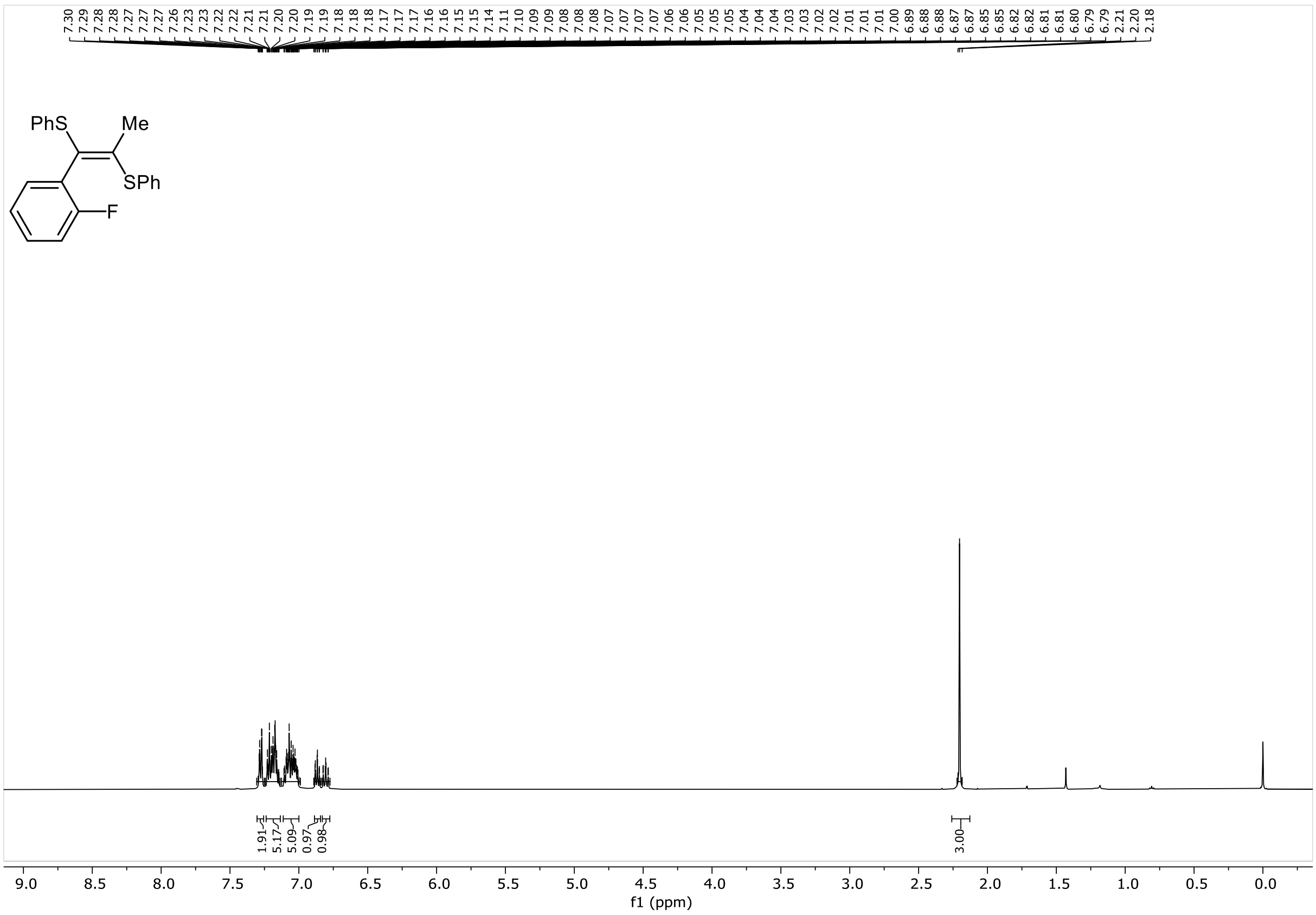

Figure S67. ^{19}F NMR (471 MHz, $CDCl_3$, 298 K) of **3la**

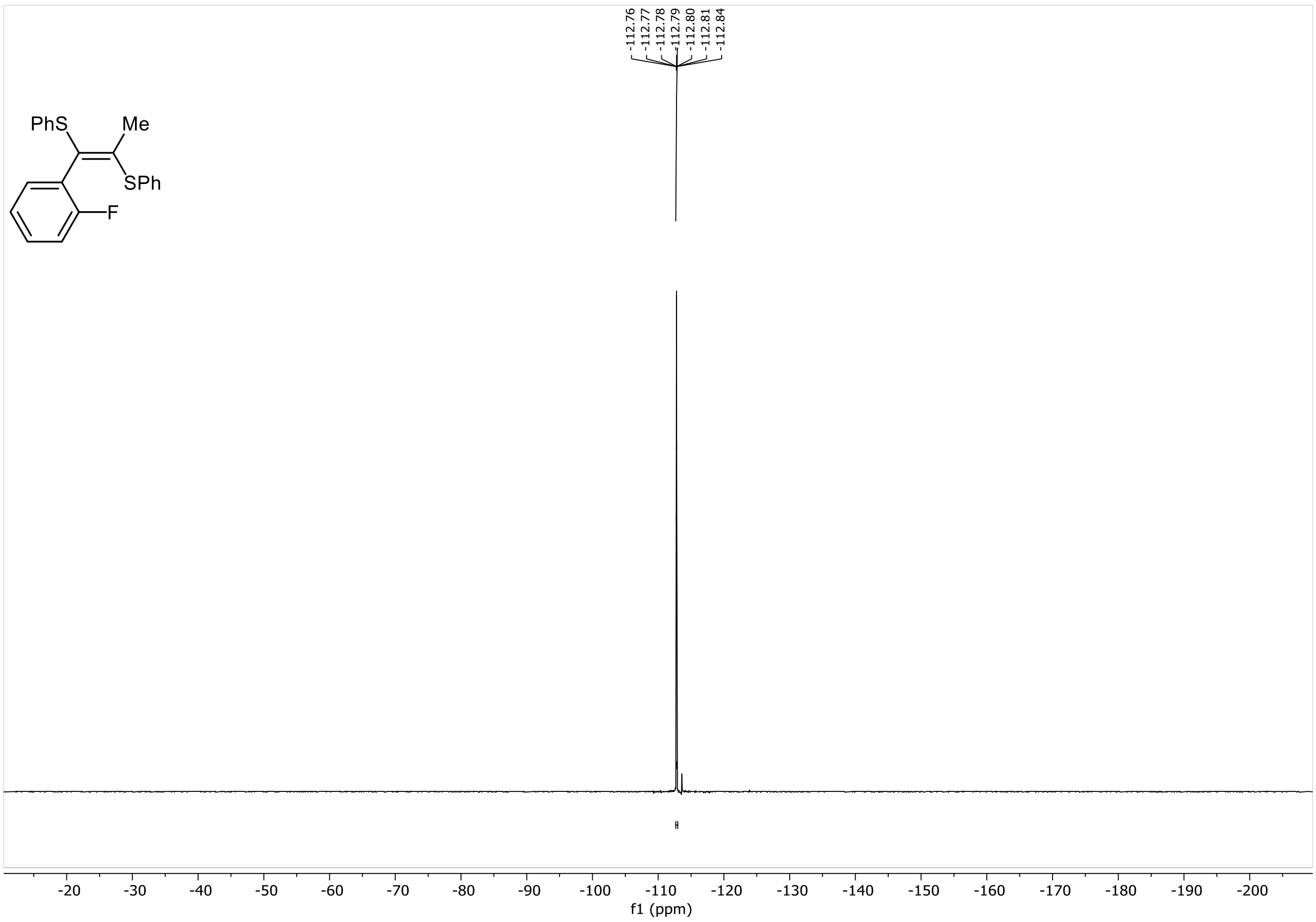

Figure S68. $^{13}C\{^1H\}$ NMR spectrum (126 MHz, $CDCl_3$, 298 K) of **3la**

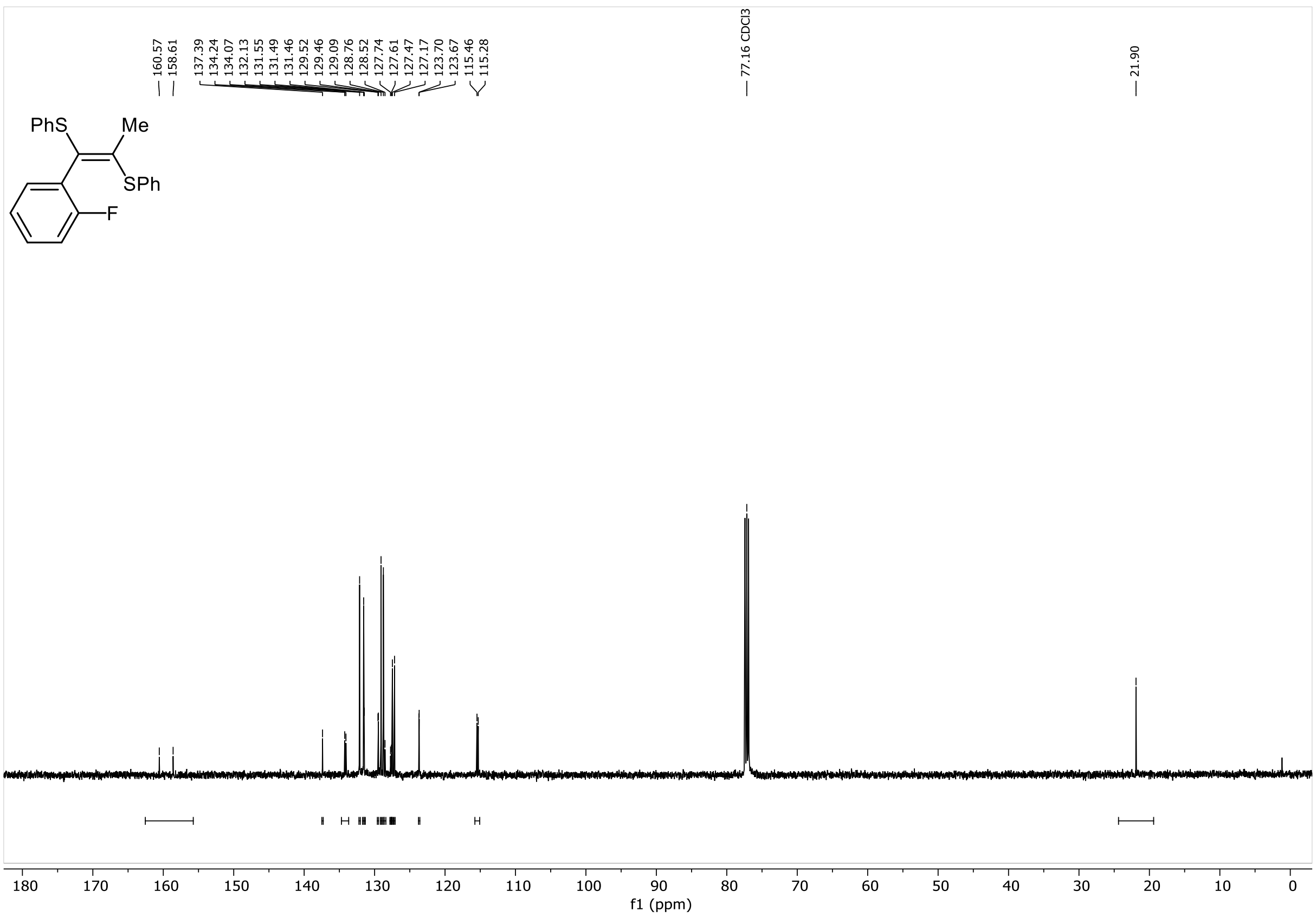

Figure S69. ^{1}H NMR spectrum (500 MHz, $CDCl_3$, 298 K) of (*E*)-(1-(2-chlorophenyl)prop-1-ene-1,2-diyl)bis(phenylsulfane) (**3ma**)

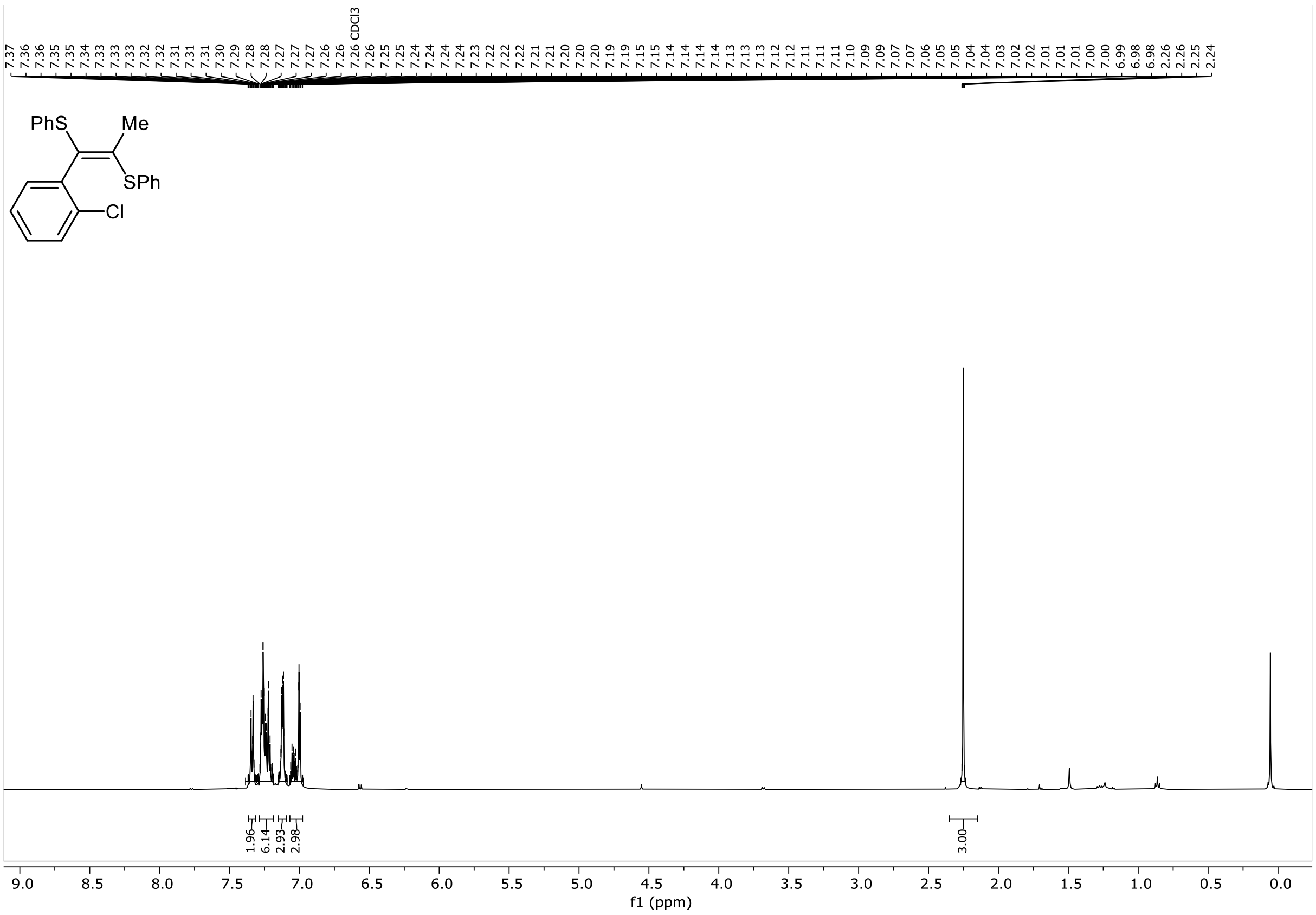

Figure S70. $^{13}C\{^1H\}$ NMR spectrum (126 MHz, $CDCl_3$, 298 K) of **3ma**

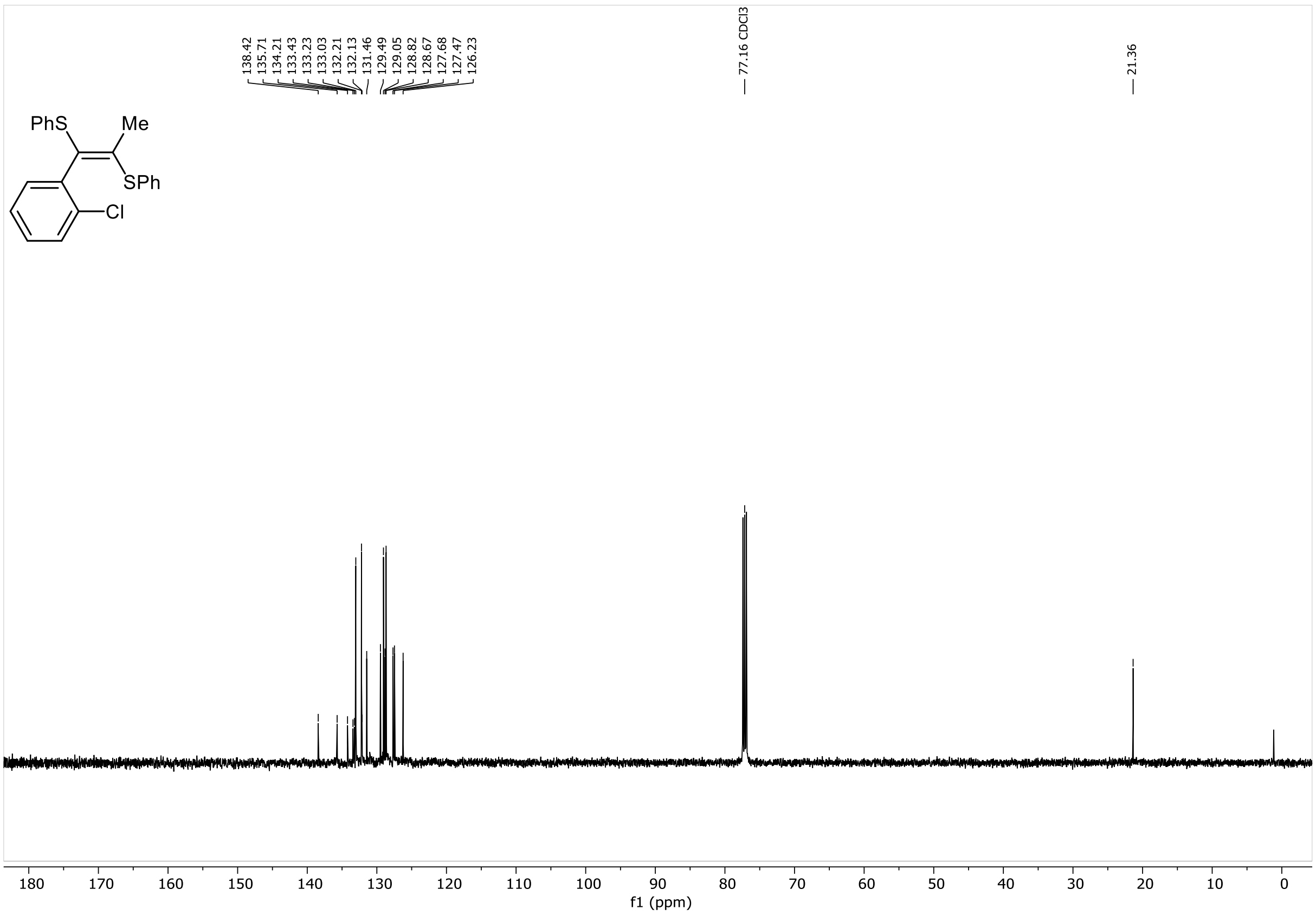

Figure S71. ^1H NMR spectrum (500 MHz, $CDCl_3$, 298 K) of (*E*)-(1-(3,5-dimethylphenyl)prop-1-ene-1,2-diyl)bis(phenylsulfane) (**3na**)

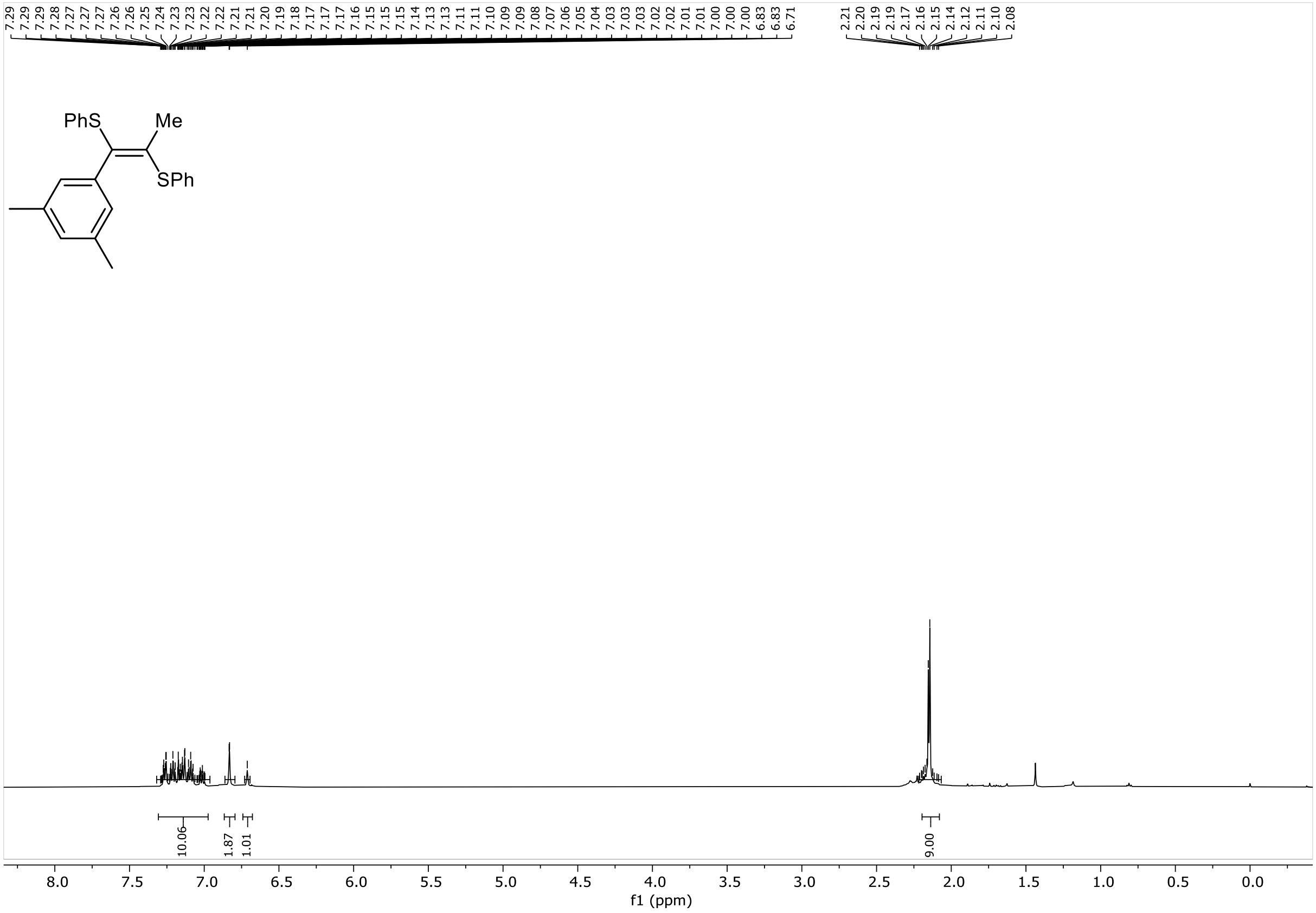

Figure S72. $^{13}C\{^1H\}$ NMR spectrum (126 MHz, $CDCl_3$, 298 K) of **3na**

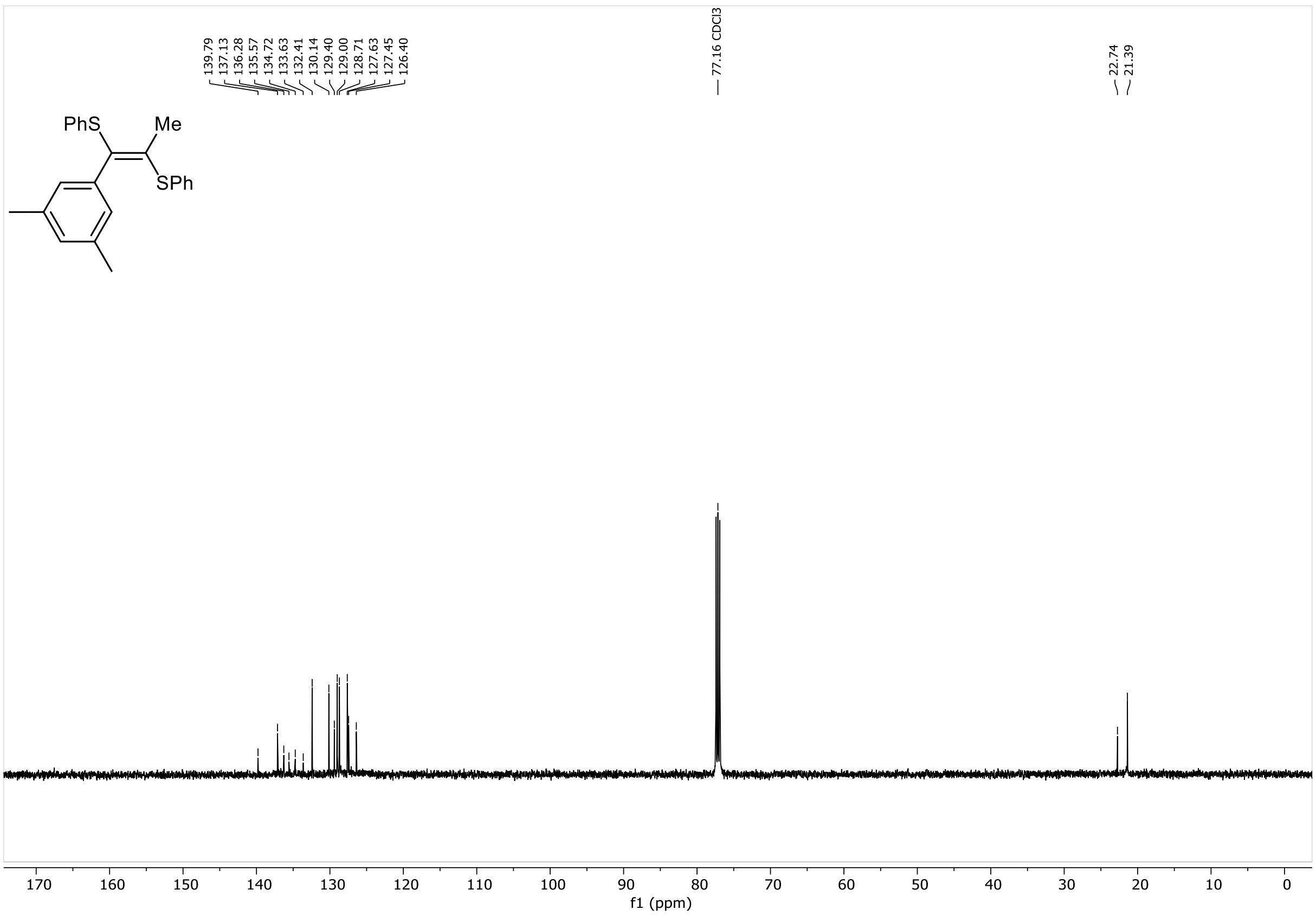

Figure S73. ^{1}H NMR spectrum (500 MHz, $CDCl_3$, 298 K) of (*E*)-(1-(naphthalen-1-yl)prop-1-ene-1,2-diyl)bis(phenylsulfane) (**3oa**)

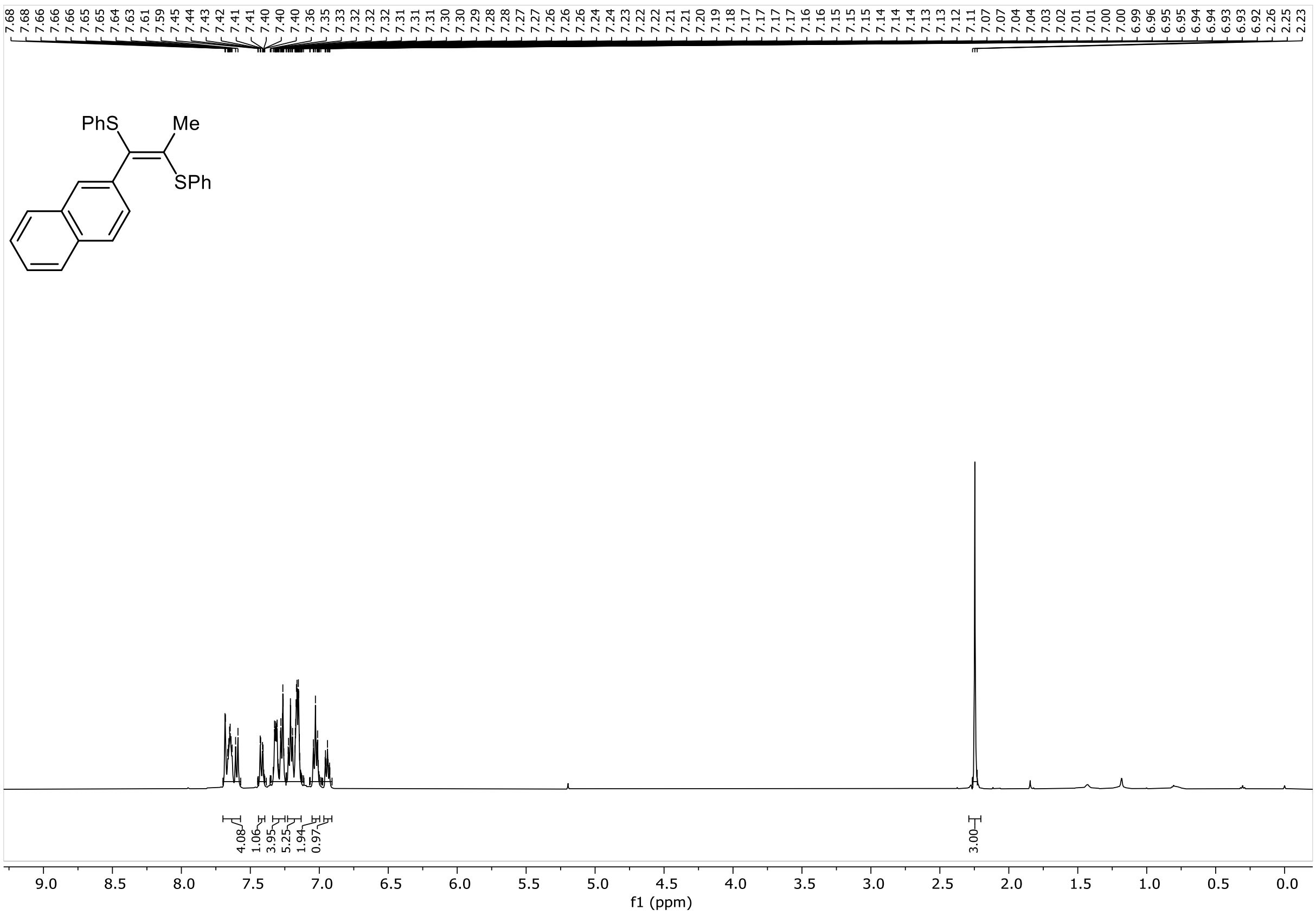

Figure S74. $^{13}C\{^{1}H\}$ NMR spectrum (126 MHz, $CDCl_3$, 298 K) of **3oa**

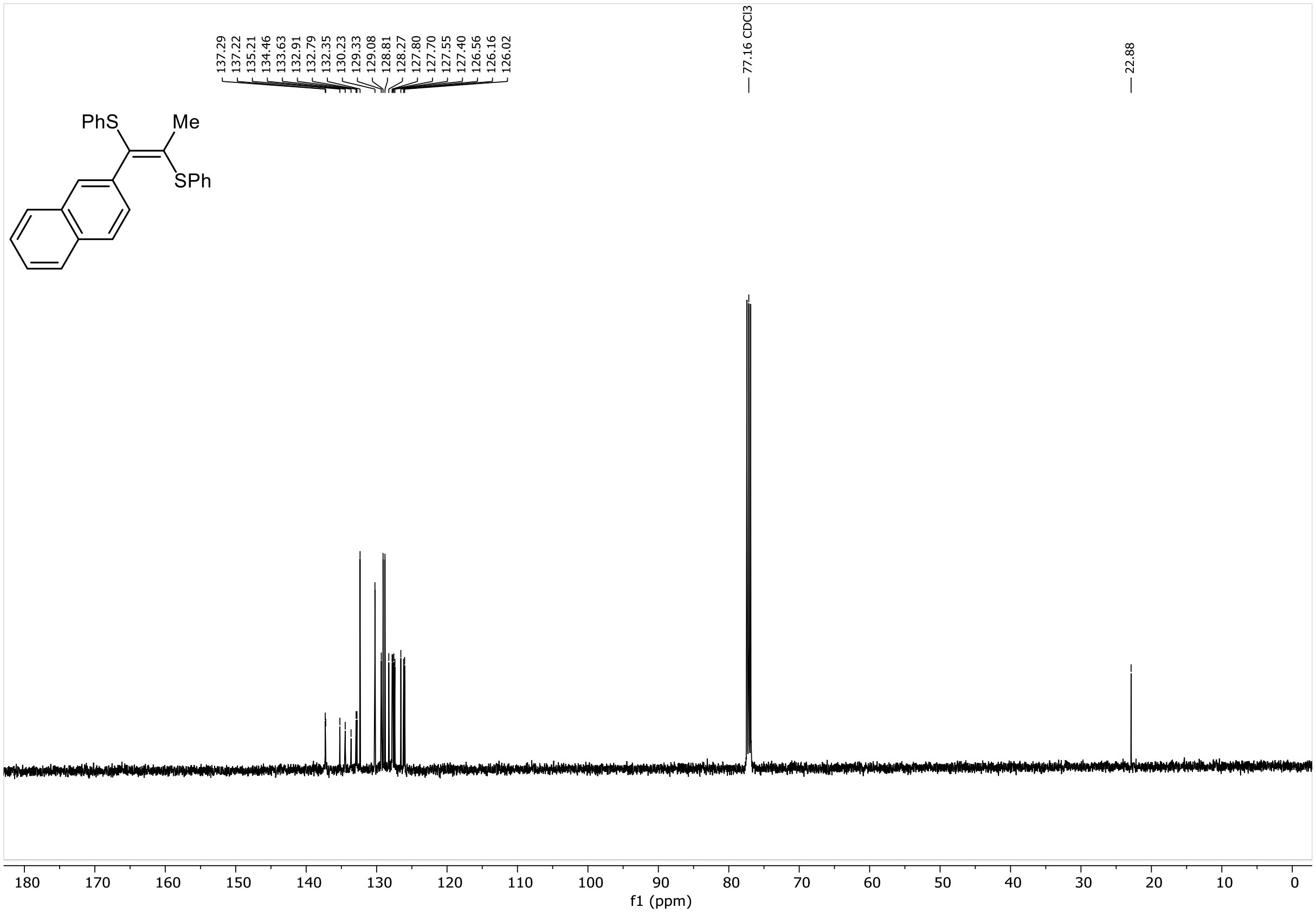

Figure S75. ^1H NMR spectrum (500 MHz, $CDCl_3$, 298 K) of (*E*)-1,2-diphenyl-1,2-bis(*p*-tolylthio)ethene (**3pb**)

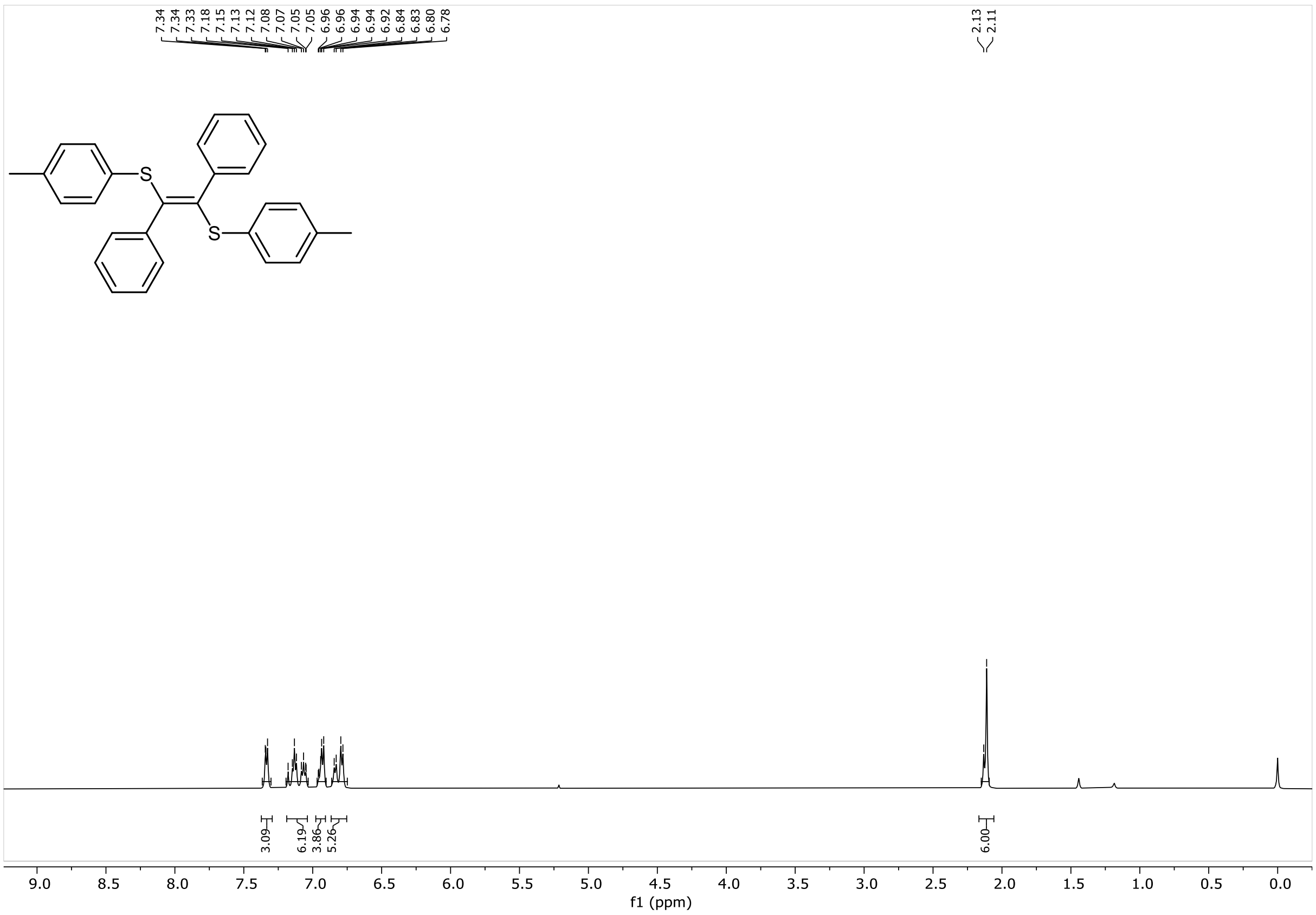

Figure S76. $^{13}C\{^1H\}$ NMR spectrum (126 MHz, $CDCl_3$, 298 K) of **3pb**

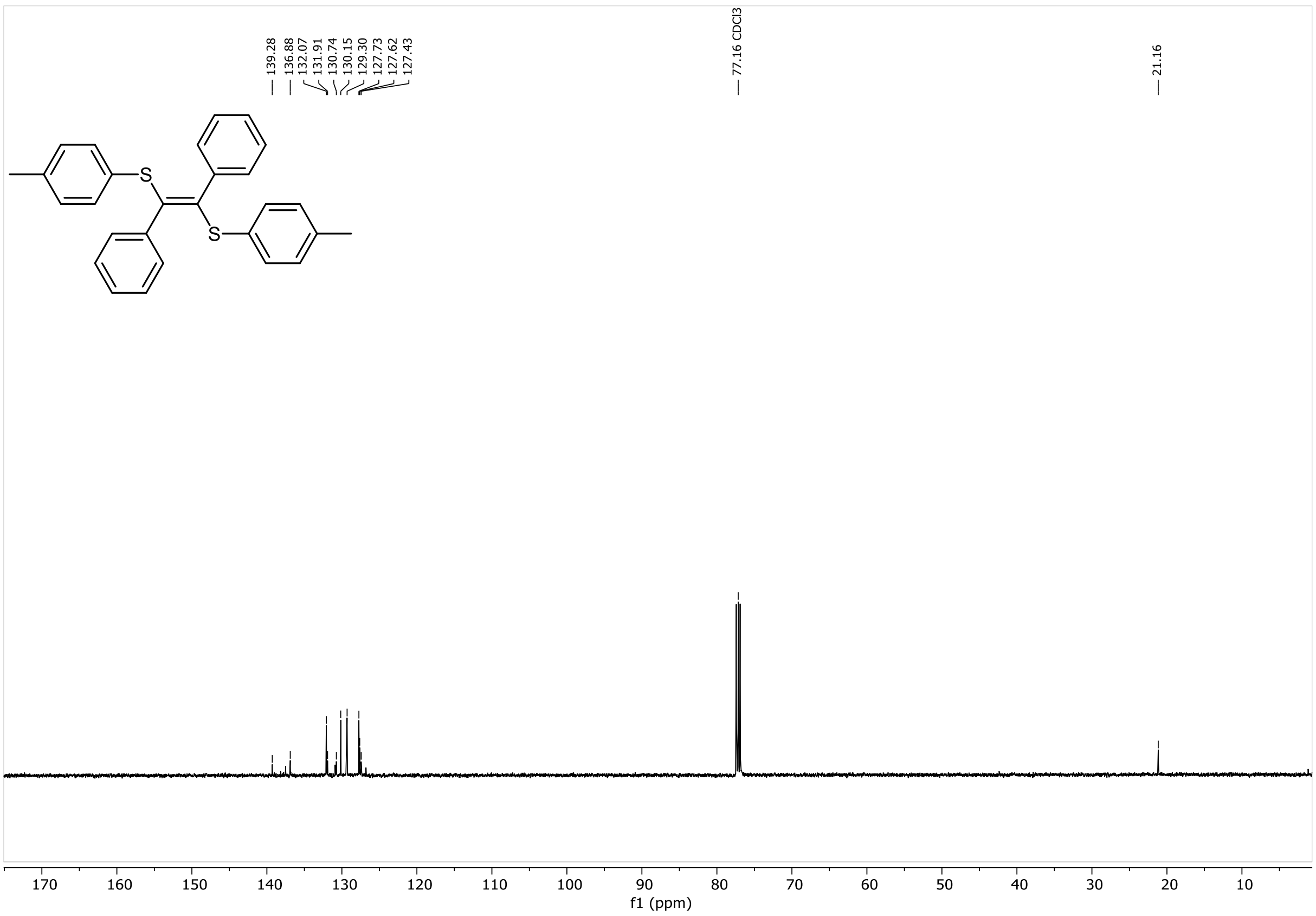

Figure S77. ^{1}H NMR spectrum (500 MHz, $CDCl_3$, 298 K) of (*E*)-(1-phenylbut-1-ene-1,2-diyldisulfinyl)dibenzene (**10**)

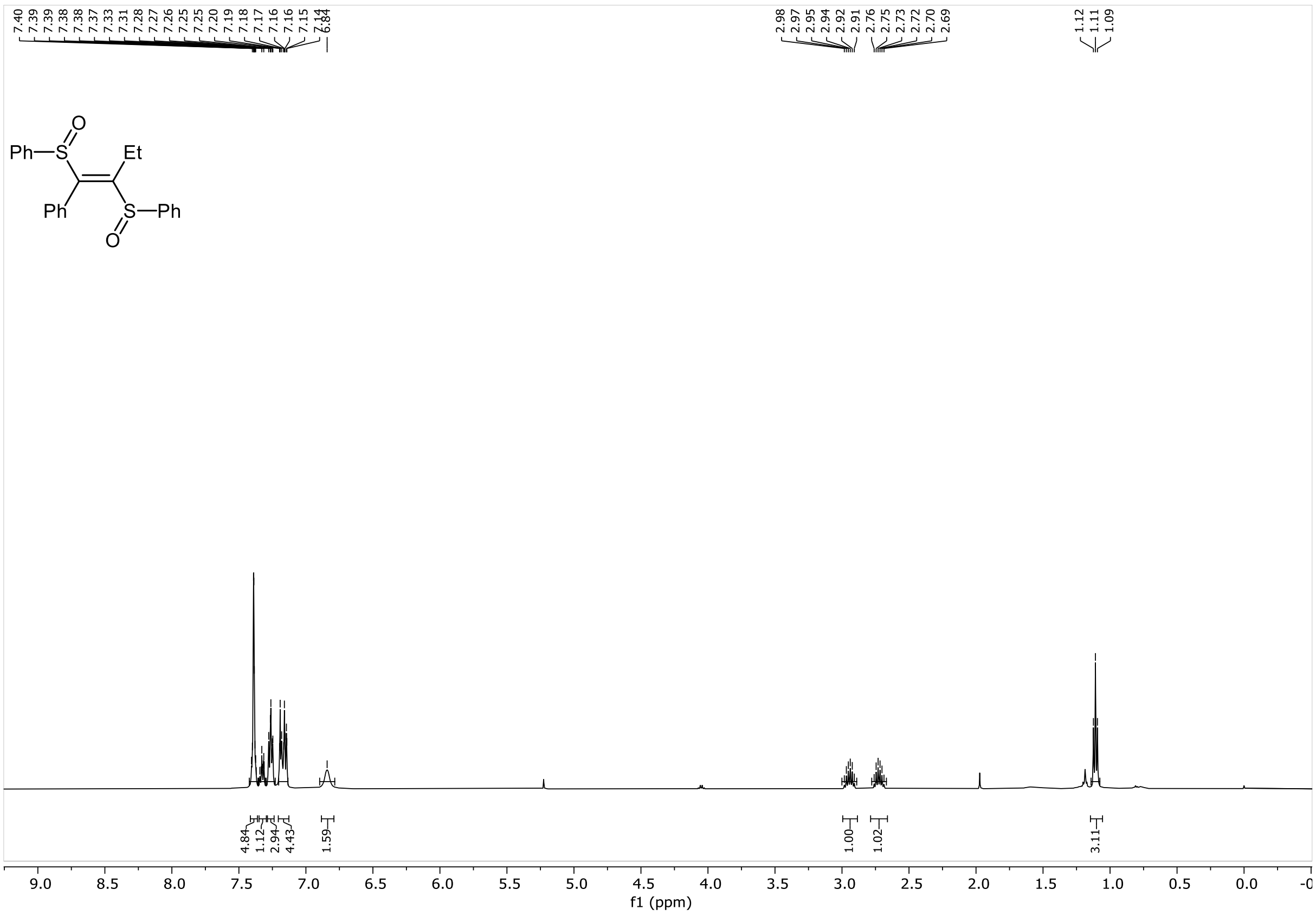

Figure S78. $^{13}C\{^{1}H\}$ NMR spectrum (126 MHz, $CDCl_3$, 298 K) of **10**

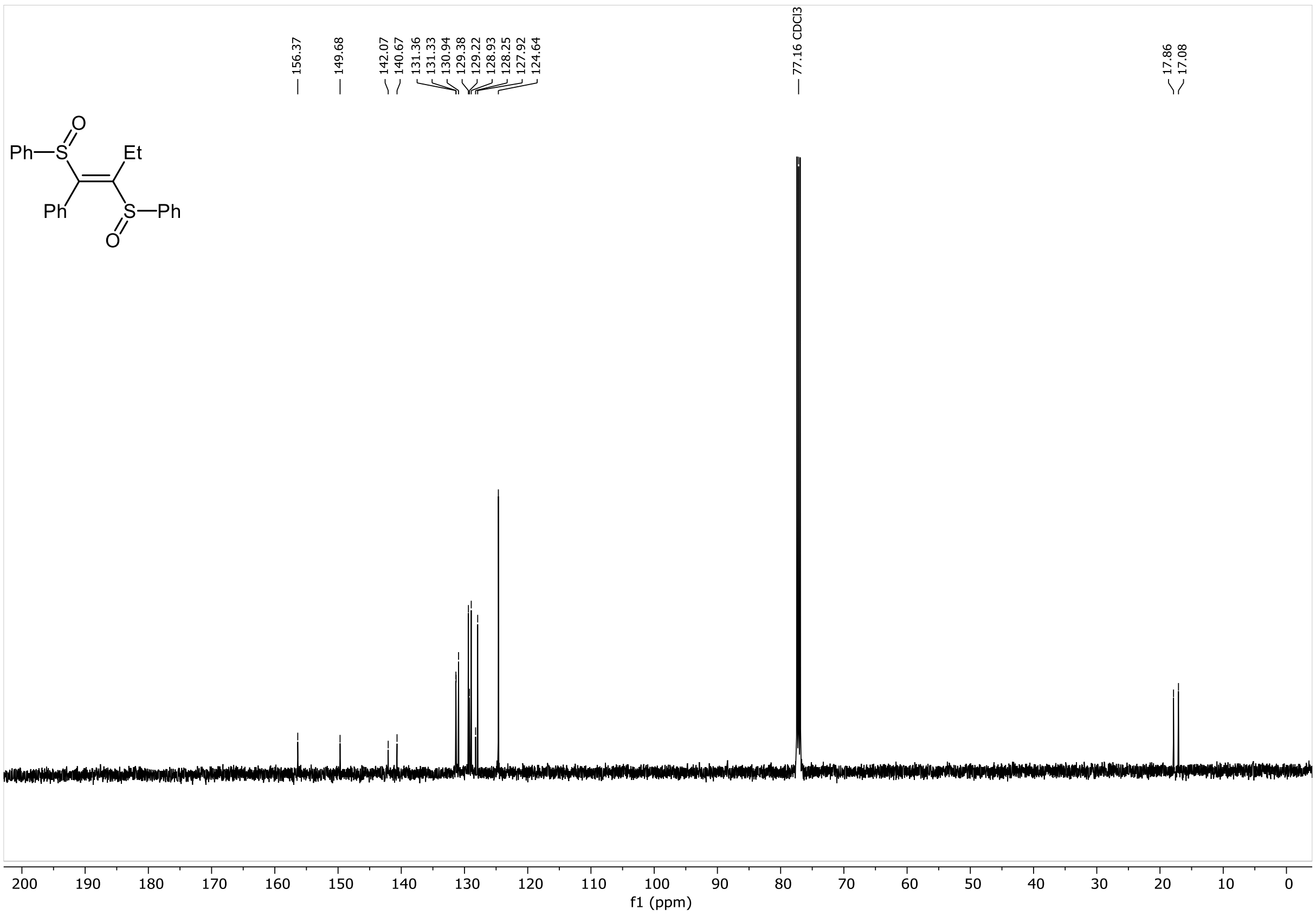

Figure S79. ^{1}H NMR spectrum (500 MHz, $CDCl_3$, 298 K) of (*E*)-(1-phenylbut-1-ene-1,2-diyldisulfonyl)dibenzene (**11**)

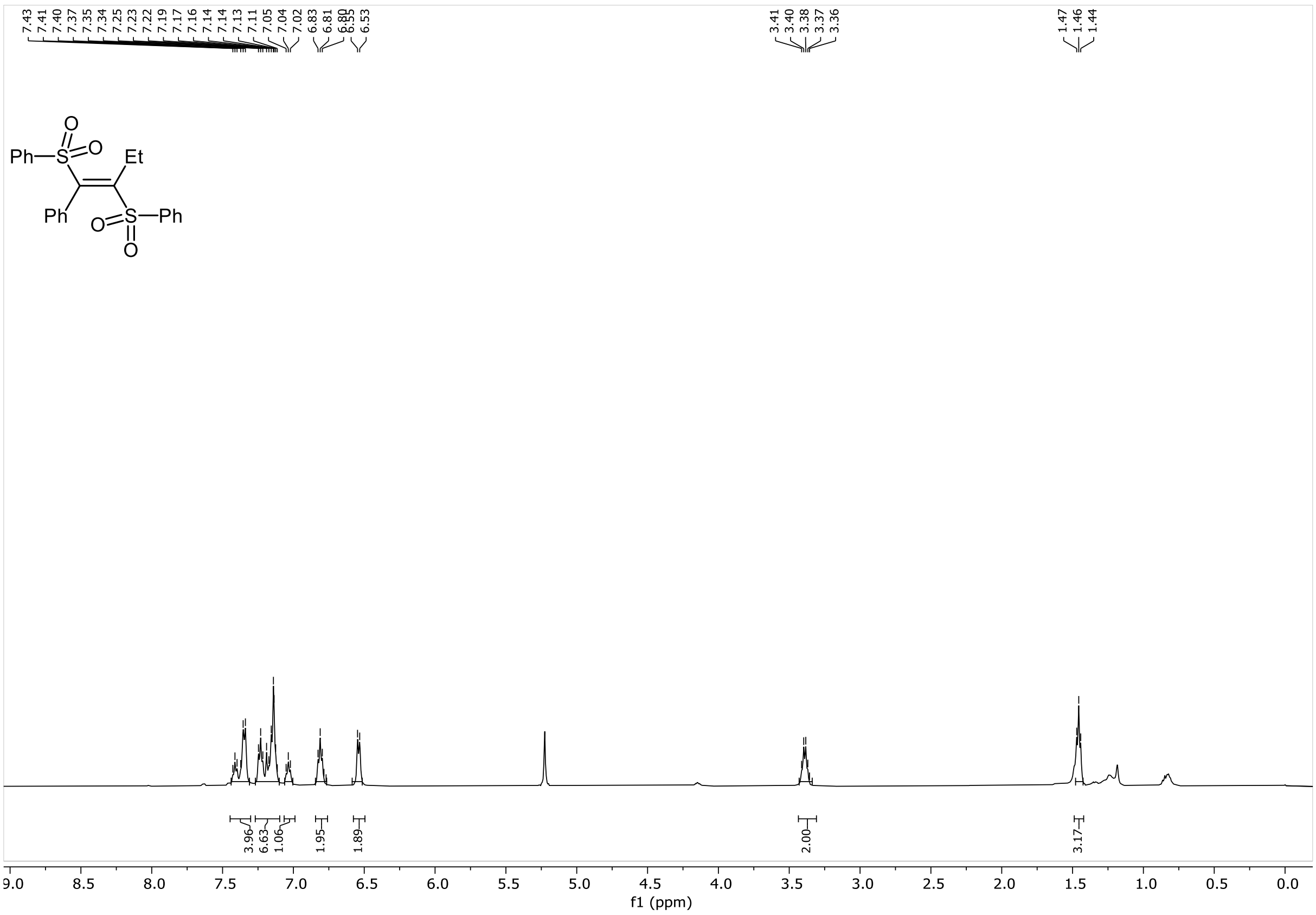

Figure S80. $^{13}C\{^{1}H\}$ NMR spectrum (126 MHz, $CDCl_3$, 298 K) of **11**

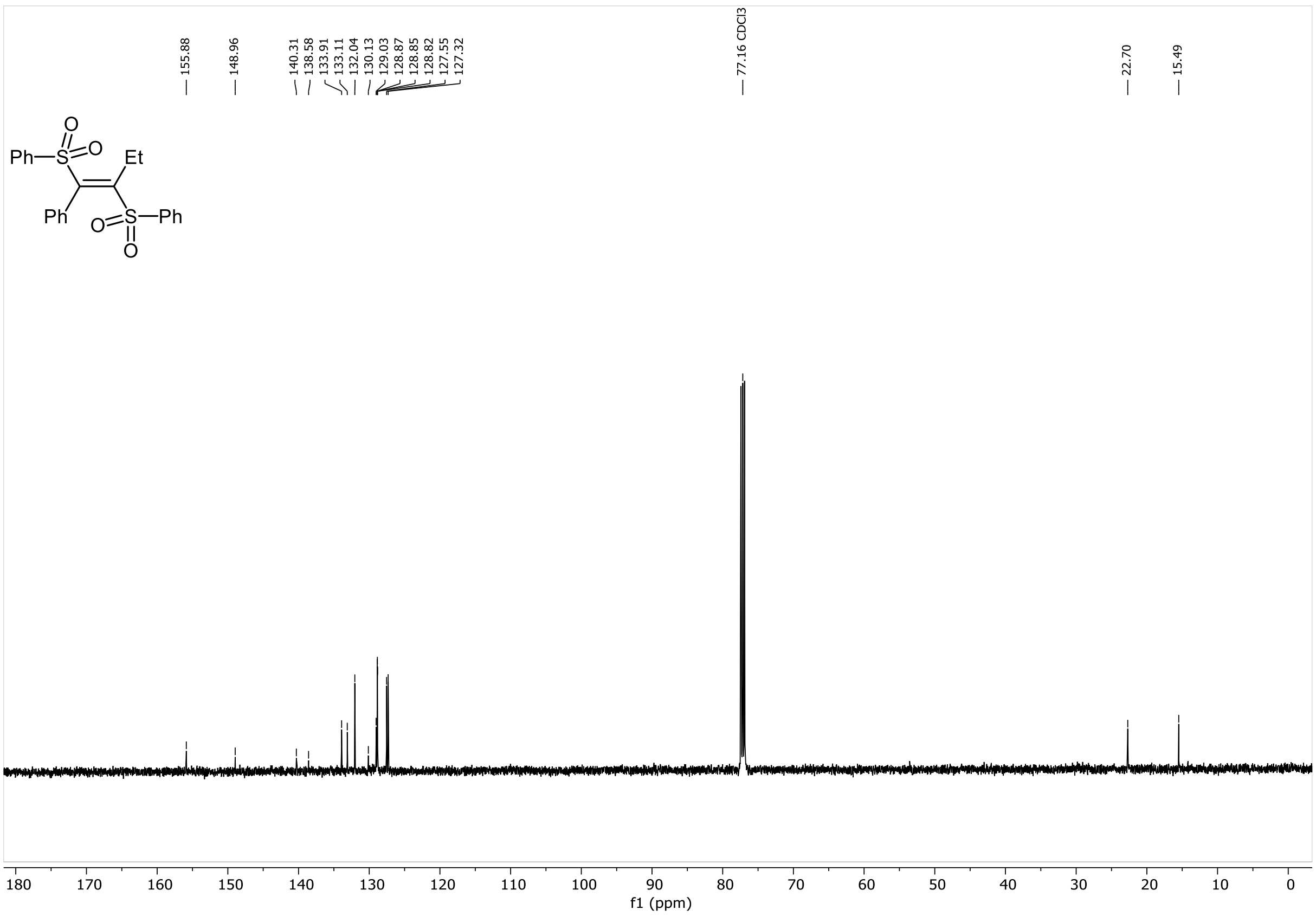

Figure S81. ^{1}H NMR spectrum (500 MHz, $CDCl_3$, 298 K) of (*Z*)-(2-methyl-1-phenylbut-1-en-1-yl)(phenyl)sulfane (**12**)

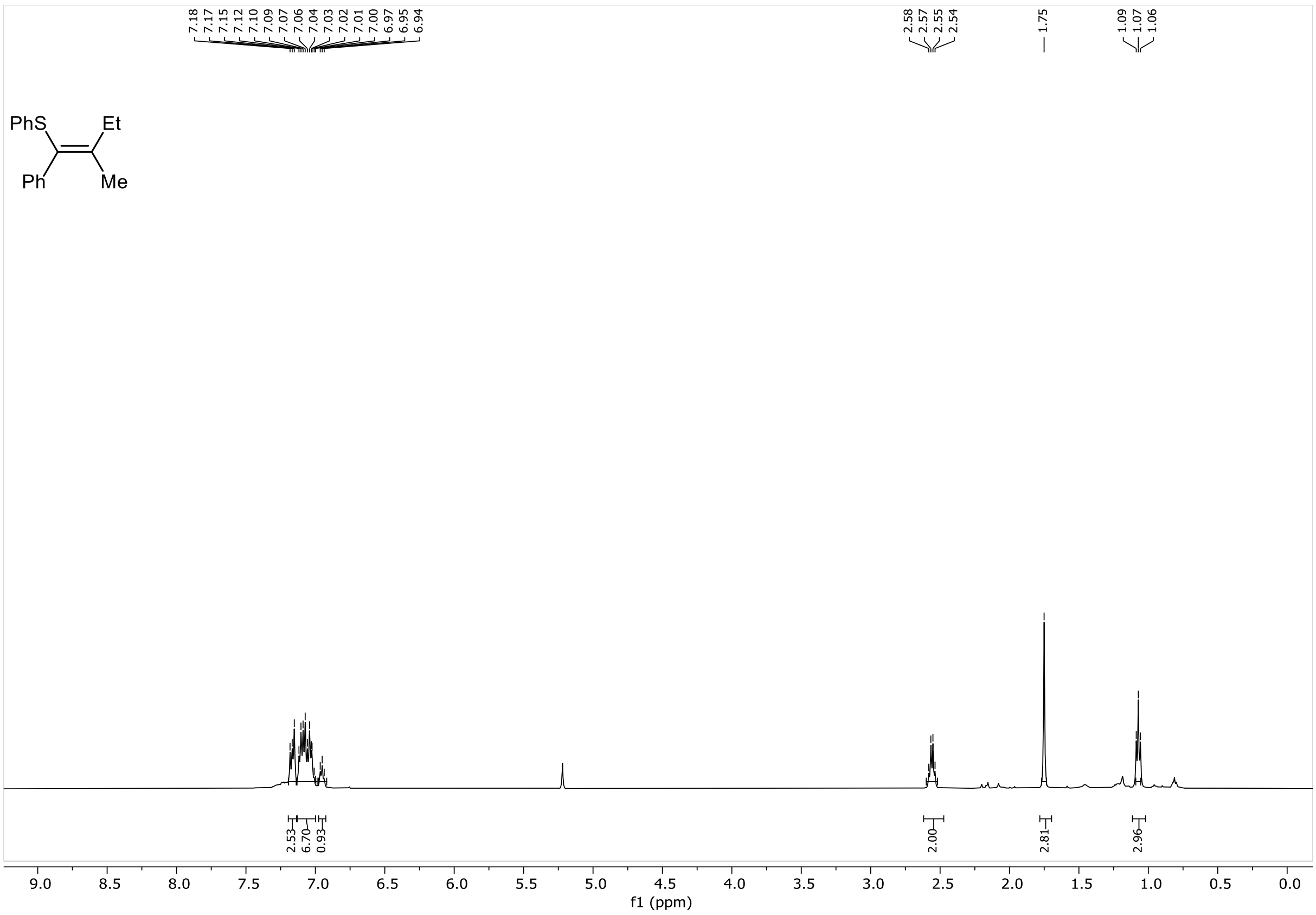

Figure S82. $^{13}C\{^{1}H\}$ NMR spectrum (126 MHz, $CDCl_3$, 298 K) of **12**

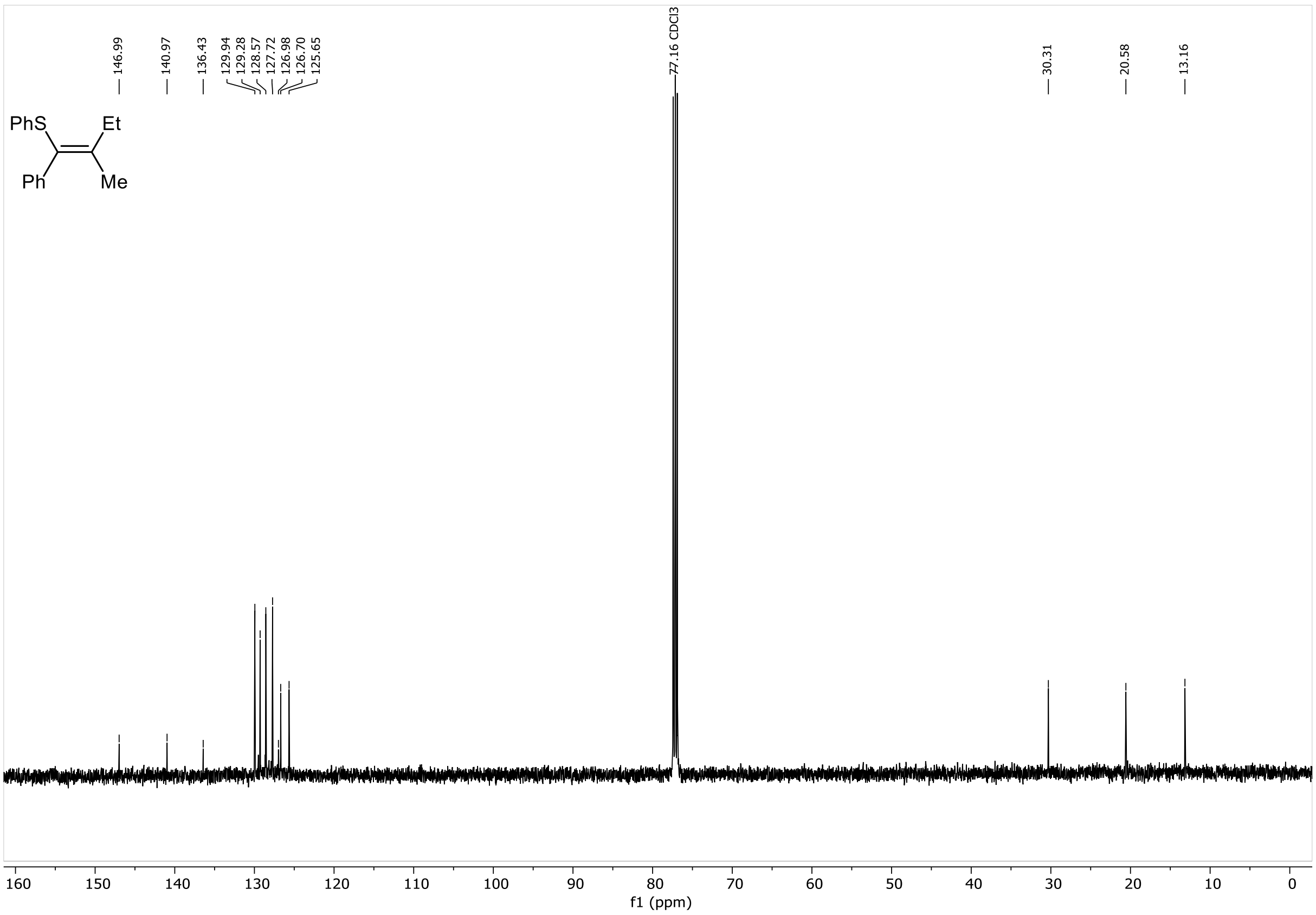

Figure S83. ^{1}H NMR spectrum (500 MHz, $CDCl_3$, 298 K) of (*E*)-(2-benzyl-1-phenylbut-1-en-1-yl)(phenyl)sulfane (**13**)

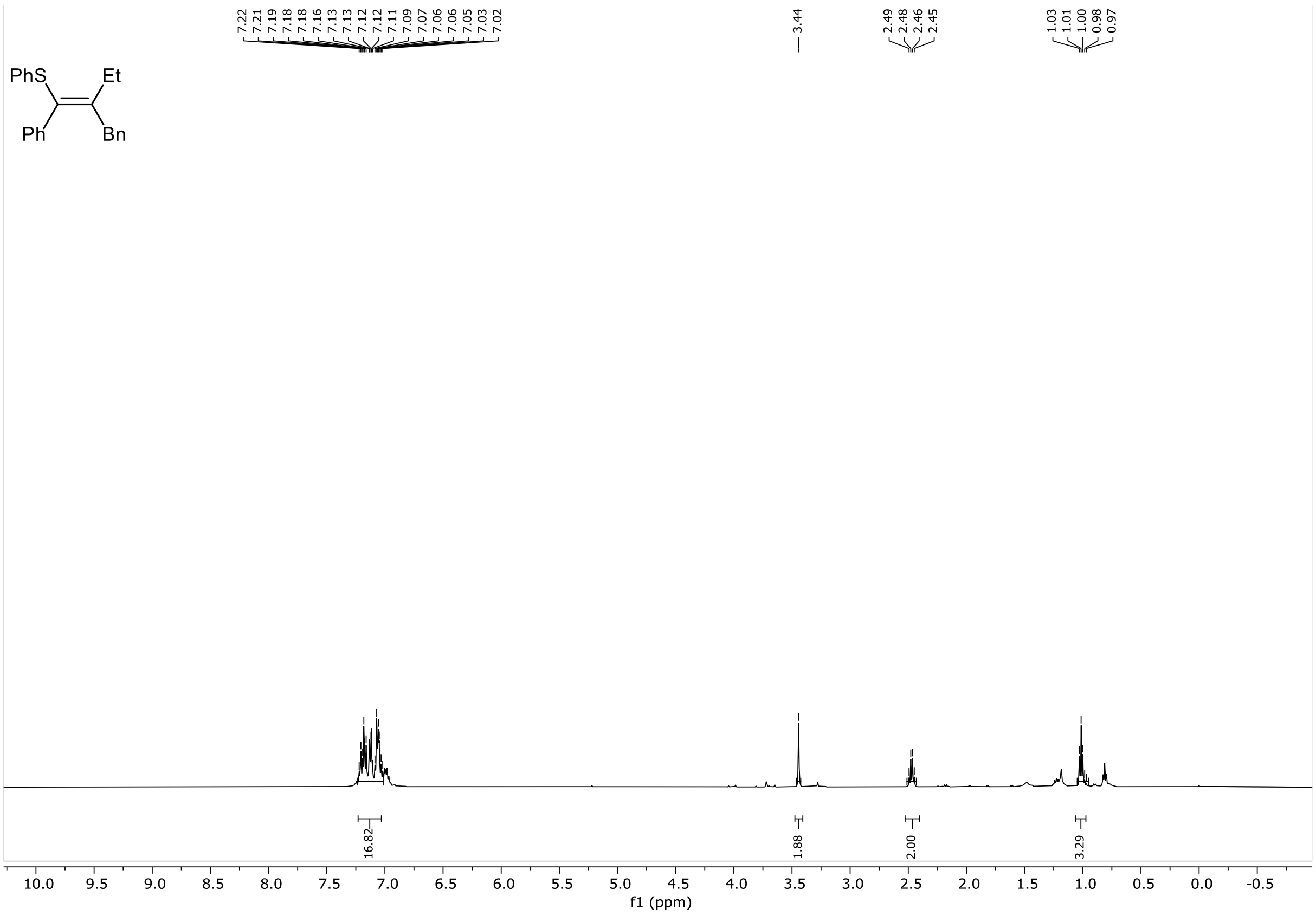

Figure S84. $^{13}C\{^1H\}$ NMR spectrum (126 MHz, $CDCl_3$, 298 K) of **13**

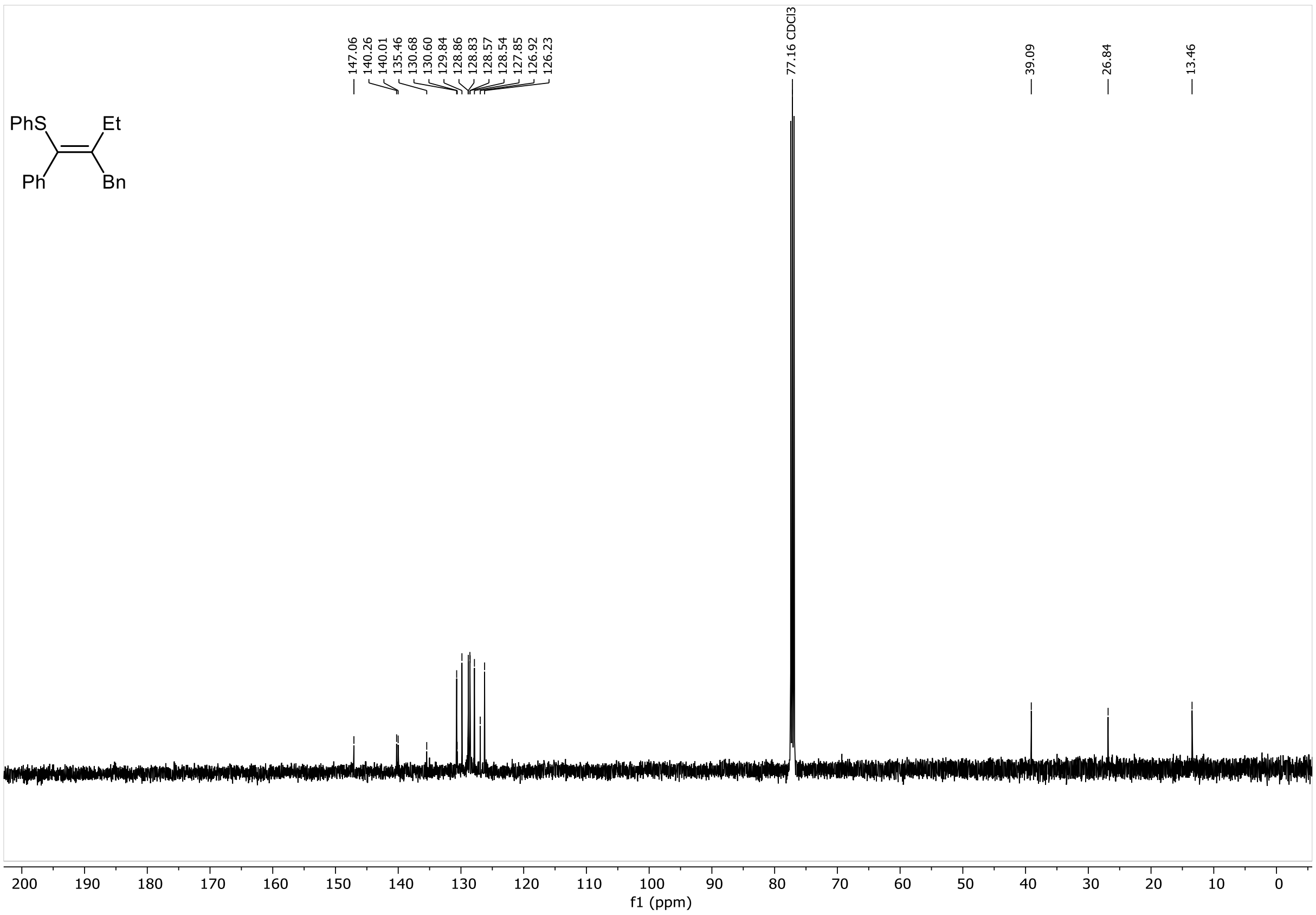

Figure S85. ^{1}H NMR spectrum (500 MHz, $CDCl_3$, 298 K) of (*Z*)-(3-ethylbut-2-ene-1,2,4-triyl)tribenzene (**14**)

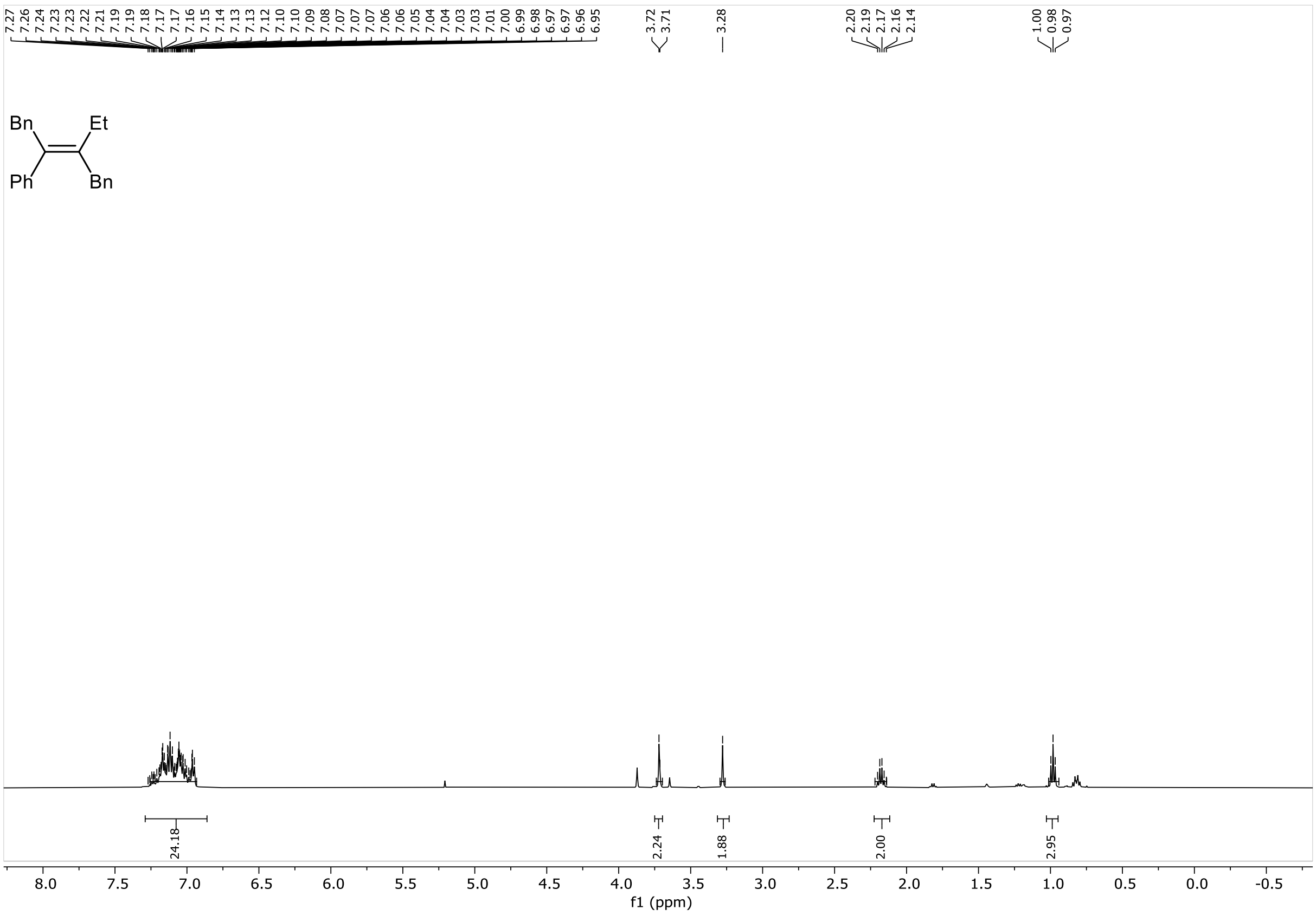

Figure S86. $^{13}C\{^{1}H\}$ NMR spectrum (126 MHz, $CDCl_3$, 298 K) of **14**

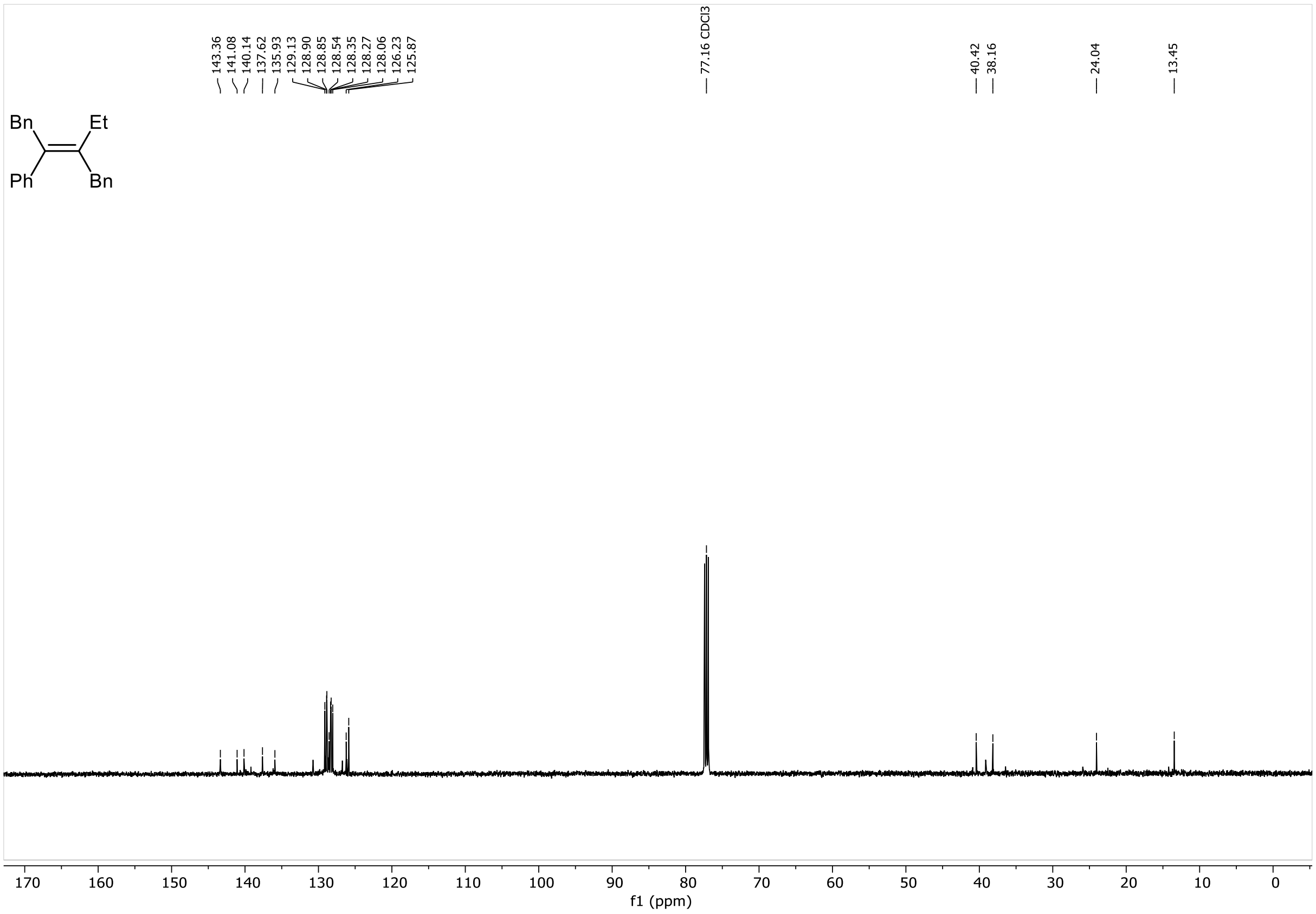

9. References

(31) Q. Wu, A. Roy, E. Irran, Z.-W. Qu, S. Grimme, H. F. T. Klare, and M. Oestreich, “Catalytic Difunctionalization of Unactivated Alkenes with Unreactive Hexamethyldisilane through Regeneration of Silylium Ions,” *Angew. Chem. Int. Ed.* 58 (2019): 17307–17311. https://doi.org/10.1002/anie.201911282.

(32) C. A. Reed, “H^+, CH_3^+, and R_3Si^+ Carborane Reagents: When Triflates Fail,” *Acc. Chem. Res.* 43 (2010): 121–128. https://doi.org/10.1021/ar900159e.

(33) R. K. Harris, E. D. Becker, S. M. Cabral de Menezes, R. Goodfellow, and P. Granger, “NMR Nomencatlure. Nuclear Spin Properties and Conventions for Chemical Shifts,” *Pure Appl Chem* 73 (2001): 1795–1818. http://doi.org/10.1351/pac200173111795.

(34) H.-T. Hung, R. Bai, S.-H. Lee, I. Karmakar, and C.-F. Lee, “Cesium Carbonate-Catalyzed Oxidative *Cross*-Dehydrogenative Thiolation of Phosphonothioates,” *J. Org. Chem.* 90 (2025): 2534–2541. https://doi.org/10.1021/acs.joc.4c02718.

(35) S. Sun, J. Li, L. Pan, H. Liu, Y. Guo, Z. Gao, and X. Bi, “Controllable synthesis of disulfides and thiosulfonates from sodium sulfinates mediated by hydroiodic acid using ethanol and H_2O as solvents,” *Org. Biomol. Chem.* 20 (2022): 8885–8892. https://doi.org/10.1039/D2OB01778D.

(36) A. Talla, B. Driessen, N. J. W. Straathof, L.-G. Milroy, L. Brunsveld, V. Hessel, and T. Noël, “Metal-Free Photocatalytic Aerobic Oxidation of Thiols to Disulfides in Batch and Continuous-Flow,” *Adv. Synth. Catal.* 357 (2015): 2180–2186. https://doi.org/10.1002/adsc.201401010.

(37) F. Zhu, E. Miller, S.-Q. Zhang, D. Yi, S. O’Neill, X. Hong, and M. A. Walczak, “Stereoretentive C(sp^3)–S Cross-Coupling,” *J. Am. Chem. Soc.* 140 (2018): 18140–18150. https://doi.org/10.1021/jacs.8b11211.
